# Effect of Physical Violence on Sexually Transmitted Infections and Treatment Seeking Behaviour among Female Sex Workers in Thane District, Maharashtra, India

**DOI:** 10.1371/journal.pone.0150347

**Published:** 2016-03-02

**Authors:** Ravi Prakash, Suneedh Manthri, Shaikh Tayyaba, Anna Joy, Sunil Saksena Raj, Devender Singh, Ashok Agarwal

**Affiliations:** HIV/AIDS Partnership for Impact through Prevention, Private Sector and Evidence-based Programming (PIPPSE) Project, Public Health Foundation of India, New Delhi, India; University of Maryland School of Medicine, UNITED STATES

## Abstract

**Background:**

Violence against sex workers can heighten their vulnerability to HIV and other sexually transmitted infections (STIs). Evidence suggests the risk of acquiring STI/HIV infections among female sex workers (FSWs) who have experienced violence to be almost three-times higher than FSWs, who have not experienced violence. Moreover, an experience of physical and sexual violence makes it difficult for them to negotiate safer sex with their partners and often act as a barrier to utilization of prevention services.

**Methods:**

This study utilizes data from 2785 FSWs aged 18 years and above who participated in a cross-sectional behavioural study conducted during 2013–14 in Thane district, Maharashtra. A probability-based two-stage cluster sampling method was used for data collection. This study assesses the effect of physical violence on self-reported STI symptoms (any STI and multiple STIs) and treatment seeking for the last STI symptom using propensity score matching method.

**Results:**

About 18% of sampled FSWs reported physical violence at the time of the survey. The likelihood of experiencing such violence was significantly higher among FSWs who solicited clients at public places, engaged in other economic activities apart from sex work, had savings, and reported high client volume per week. FSWs experiencing violence were also inconsistent condom users while engaging in sex with regular partners and clients. The average adjusted effect of violence clearly depicted an increase in the risk of any STI (11%, p<0.05) and multiple STIs (8%, p<0.10) and reduction in treatment seeking (10%, p<0.05).

**Conclusions:**

This study demonstrates a significant effect of physical violence on reporting of any STI symptom and treatment seeking. Findings call for the immediate inclusion of strategies aimed to address violence related challenges in HIV prevention program currently being provided at Thane district. Such strategies would further help in enhancing the access to tailored STI prevention and care services among FSWs in the district.

## Introduction

Globally about 1.5 million people died from HIV/AIDS-related causes, and nearly 2.1 million new infections detected by the end of the year 2013.[1] In India, about 310,000 deaths caused due to AIDS-related by the end of the year 2013. As the same year, around 2.1 million people were living with HIV and of them, 750,000 were women aged 15 and above living with HIV.[2] Most of the new HIV infections in India were transmitted through heterosexual route. Even though risk factors vary among core groups such as men having sex with men (MSM), female sex workers (FSWs), injecting drug users (IDUs), transgenders (TGs) and bridge population such as truckers and migrants, [3, 4] among all the population sub-groups, FSWs are considered as one of the core groups most vulnerable to HIV transmission due to various reasons including sex with multiple partners, inconsistent condom use, and higher infection rates.[3, 4] Moreover, the HIV prevalence in India is estimated to be more than 54 times higher among sex workers as compared to the general population.[5, 6]

Worldwide, sex work has been considered as a primordial and widespread profession among females and there is a presence of about 40 million FSWs, including an estimated 870,000 FSWs in India.[2, 7] India has become one of the most flourishing sex industries in the world and Mumbai, Delhi, Chennai, and Kolkata are being at the epicenter of this traffic.[3, 7] Despite of having such a large volume, sex workers have often found to be marginalized and experience discrimination and violence from their intimate partners (non-paying), regular and occasional clients, relatives, and gatekeepers.[8] In addition, young sex workers have often been found to be suffering from more psychological disturbances such as depression, anxiety, irritability, distrust, shame, rejection, low self-esteem and post-traumatic stress disorder as a result of violence perpetrated by different clients/partners.[9]

FSWs in India have experienced high levels of physical and sexual violence from clients, *madams* (brothel owners) and police.[10] Recent evidence from India suggests reporting of sexual violence among FSWs lies between 20% to 63%.[10] However, in a study from southern India, the reported prevalence of physical and sexual violence among FSWs ranged between 50% to 77%.[10] Evidence from in south and western India further suggests high level of sexual violence was perpetrated by partners of FSWs.[11–13] Moreover, FSWs experienced sexual violence were more likely to be vulnerable to both reproductive health problems and HIV risks.[11] Existing evidence among FSWs suggests that acts of physical and sexual violence are independently associated with a number of factors including inconsistent condom use, STI symptoms and infection, anal sex, multiple forced pregnancy terminations and suicidal attempts.[11, 14–17]

Violence against women can heighten their vulnerability to HIV and other sexually transmitted infections (STIs).[18–21] The risk of acquiring HIV infection among women who have experienced violence are found to be almost three times higher than the women who did not experience violence.[22, 23] Experience of violence among women, makes them difficult to negotiate with their intimate partners, clients, *madams* and *pimps* (broker) for safer sex, and thus leads to higher chances of STI/HIV among them.[24] Studies have also found that women living with HIV were more vulnerable to violence. Many a times act of such violence limit the ability of women to avail the HIV care and treatment services that they need.[25–28]

Maharashtra state, in western India, has a population of approximately 110 million and ranks among the top four state in India with regard to HIV epidemic severity with an estimated HIV/AIDS prevalence of 0.42% among general population (higher than the national average of 0.27%), rates among FSWs have reached 6.89% by the year 2012.[29] The District Thane, situated in the western part of Maharashtra state, is one of the districts in Maharashtra having high HIV prevalence among high-risk groups and a moderate prevalence among the general population. As per the eight round of HIV Sentinel Surveillance (HSS) data, the HIV epidemic trends for the district indicate high prevalence (more than 5%) among HRGs and a prevalence of less than one percent among generation population. Moreover, due to presence of a large network of clients of FSWs (58,972), FSWs (15,550), MSMs (4,827), TGs (2,020), IDUs (688), and migrants (934,285) in the district, there is a high potential for increase in the rates of STI/HIV transmission in the future.[29, 30] The Thane district has a population of 11.03 million, which is about one percent of the national population[31], high decadal population growth rate and is one of the districts with poorest sex ratio (880 females per thousand males) which is far below the national average (940 females per thousand males).[28]

The HIV/AIDS Partnership: Impact through Prevention, Private Sector and Evidence-based Programming (PIPPSE) project, supported by United States Agency for International Development (USAID), is working since 2012 to deliver targeted HIV prevention intervention programs under District Network Model (DNM) in Thane District with active support from Maharashtra State AIDS Control Society, Government of Maharashtra.[30, 32] FSWs in the district are often from lower castes, poor economic background, with a low level of education, having more children, and live with few economic alternatives for survival if they are deserted by their husbands or became widowed. From our earlier dialogues with sex workers, it emerged that violence is a crucial concern among FSWs in Thane district, and it has a potential role in limiting the sex workers from accessing various health related preventive and clinical services.[33] Moreover, violence experienced by sex workers was considered a priority issue to address along with the provision of sexual health education and other preventive HIV/AIDS services offered through the project. Although, evidence on linkages between the experience of violence and STIs are well documented in Indian settings and they are not particularly available in the context of Thane district. None of the previous studies has also attempted to evaluate the impact of violence on the prevalence of STIs and treatment seeking behaviour due methodological difficulties in the evaluation. For example, existing evidence are based on the cross-sectional data and do not have an appropriate control group. Moreover, FSWs, who experienced physical violence are usually different in a set of unobserved (perception, attitudes) as well as observed characteristics (background characteristics) from those who have not experienced violence that, sometimes, is difficult to consider in conventional regression analysis. Statistical methods such as propensity score matching allow overcoming these limitations to a large extent.

Considering potential adverse effects of physical violence on health status of FSWs, this paper aims at examining the net (true) effect of violence on the self-reported STIs and the treatment seeking behaviour among FSWs in Thane district. We investigate the extent to which the net difference observed in the outcome between treated and untreated groups of FSWs, i.e. those experienced violence versus those who did not, could be attributed to violence given that the potential covariates are matched appropriately. In the absence of information on sexual violence from the survey, only the effect of physical violence has been discussed in this paper.

## Methods and Materials

### Ethical statement

The data used for the study comes from a site assessment survey conducted to gain the details on risks and vulnerabilities of FSWs by their location and typology covered under each FSW TI NGOs implementing the HIV prevention intervention program in the Thane District. According to the targeted intervention strategy of National AIDS Control and Prevention (NACP) program in India, each NGO implementing the TI programme are mandated to conduct this assessment before rolling out the intervention. This assessment helps to the TI NGOs in validating the broad mapping estimate and help them in understanding the details on prevailing HIV related risks and vulnerabilities of high-risk population in their intervention geography. The assessment instruments and methodology followed the similar guideline as described in the National Operational Guidelines for Targeted Intervention. No separate ethical approval was sought. However, written informed consent was obtained from each participant before initiating any discussion. Confidentiality and anonymity were strictly maintained. Before the interview of the brothel and bar based study participants, permission was obtained from the brothel *madam* and bar owners after explaining the purpose of the study. FSWs were free to decline to participate in the study or not to answer questions they were not comfortable. No monetary incentive was provided to the respondents. FSWs in need of care were referred to the nearest health facility for the necessary treatment.

### Data, study setting, and sampling

The present study utilizes data from a cross-sectional behavioural site assessment carried out during 2013–14 in the Thane district. This study assesses factors that elevate the HIV-related risks and vulnerabilities among FSWs, identify their HIV-related needs and implementation gaps in the existing HIV interventions.

Any female, 18 years or older who sold sex in exchange for cash at least once in the last one month was as an eligible FSWs for this assessment study. A probability-based two-stage cluster sampling method was used. At the first-stage, selection of sites was done using conventional cluster sampling for stable populations and conventional time-location cluster sampling for the mobile population. Stable population includes FSWs based at home, brothels, lodges, and Dhaba’s (road-side eating or drinking establishments), and the mobile population includes street-based FSWs. The respondents were selected systematically within each site, at the second stage. The overall sample selection was proportionally allocated to estimated proportion of sex workers of each typology in the district. The methodology was almost on the similar line as used in earlier bio-behavioural studies conducted among HRGs in southern states of India. The detailed sampling strategy for the previous bio-behavioural studies described elsewhere [34–37].

In this survey a total of 3,200 FSWs approached and 2,785 completed the behavioural interview with a response rate of 87%. These FSWs belonged to 11 Targeted Intervention (TI) NGOs implementing HIV-prevention program and geographically spread across the Thane district. The FSWs were interviewed face-to-face using pretested structured questionnaire by the trained staff of the TI NGO, who were part of the local community, t in a private setting away from the solicitation site. Interviews were conducted either in Hindi or local language that respondents speak or understand.

### Outcome variables

We used three outcomes variables, self-reported any STI symptoms, self-reported multiple STI symptoms, and STI treatment seeking behaviour defined as:

#### Self-reported any STI symptoms

Since biological samples (blood and urine) were not collected to test for syphilis or chlamydia infection, we considered any self-reported STI symptoms as the first outcome variable measuring risk behaviour among FSWs. Participants were enquired about suffering from any STI symptoms in the 12 months preceding the survey. The response categories for the above question were: genital/vaginal discharge, genital sore/ulcer, lower abdominal pain, burning sensation while urination, swelling in groin area, itching in genital areas, and pain during sexual intercourse. FSWs who reported any STI symptoms in 12 months preceding the survey were considered as ‘reporting any STI symptoms’.

#### Self-reported multiple STI symptoms

Participants suffering from more than one STI symptoms in 12 months period preceding the survey were considered as ‘reporting multiple STI symptoms’. FSWs reporting multiple STI symptoms were considered as having higher risk profile than those who suffered from a single episode.

#### STI treatment seeking behavior

The third outcome variable of interest was treatment seeking behaviour for the last STI symptoms they had in 12 months preceding the survey. Participants sought treatment/medicine/advice for the last STI symptom from government or private hospitals/clinics, traditional healers, medical shops or through the help of NGO-run-clinics were categorized as treated for STIs whereas participants did not obtain any treatment, took home based remedy or the medicine that they had at home, and just consulted with their friends or relatives were clubbed into another group, i.e., not treated for STIs.

### Exposure variables

An experience of physical violence at the hands of intimate partners, regular clients or the occasional clients including the stranger, local *goons* (local criminal or violent person in the community), police, and others was the primary exposure variable of our interest. The questionnaire contained following question about violence: “In the past twelve months, have you ever been harassed or beaten up by regular partner/husband, clients, local leaders, police and local *goons*. Those who said yes were probed about the number of times each of these persons beat them. These two variables were used to compute an indicator of FSWs’ exposure to physical violence.

### Explanatory variables

We explored the extent of similarities and differences between FSWs, who experienced physical violence versus those who did not, with regard to a number of background variables likely to influence exposure and outcome variables of interest. Background variables fell into three broad categories: socio-demographic, economic, and sex work related characteristics. Socio-demographic and economic variables included age of respondents, marital status (ever married; never married), literacy status (literate; non-literate), current residence in the form of whether they belong to place of interview (localite) or not (non-localite), co-residents (spouse/family members; alone/with friend/co-workers), and alcohol use (yes; no). Two variables on economic status of respondents includes source of income other than sex work (yes; no), and any savings (yes; no).

In order to assess the differential in experience of violence by sex work-related characteristics, we included variables representing age at first sex (below 15 years; 15 or more years), duration of sex work (below 3 years; 3 or more years), place of solicitation (public places such as street; non-public places), weekly client volume (below 10 clients per week; 10 or more clients), have a regular partner such as husband/boyfriend/lover, and exposure to any HIV prevention intervention (yes; no). Other than these, two indicators on condom use, representing the sex work characteristics, were also included in the study. These were consistent condom use with regular partners and consistent condom use with regular/occasional clients.

### Analytical approach

The customized database was prepared for the study using the Census and Survey Processing System version 4.1 (CSPro; https://www.census.gov/population/international/software/cspro) software. All data were double entered to make it free of error at the data entry level. All statistical analyses were performed using the STATA version 12.0.[38]

We used descriptive analyses to describe the population and to estimate the prevalence of physical violence. We compared the experiences of FSWs, who reported physical violence by each of the explanatory indicators described above. Chi-square tests were performed to examine associations between each background characteristics and the experience of physical violence and the association between violence and STI related outcomes of interest. All tests were two-tailed, and associations were considered significant at p<0.05.

The study adopted the Propensity Score Matching (PSM) method to examine the net effect of violence on STI prevalence (self-reported) and treatment seeking behaviour among FSWs. This approach allows assessing the true effect of exposure on desired outcomes through cross-sectional survey data.[39–42] PSM method has recently being used to analyze the impact of programme intervention and other exposure variables of interest on desired outcomes. Due to the computing strengths and associated algorithms as well as matching approach adopted by the method.[39, 43] In recent past, selected studies have used this method to evaluate public health programs in India.[44–49]

PSM generates a set of “control” cases (e.g., FSWs who did not experience physical violence) corresponding to “treatment” cases (FSWs who experienced physical violence) by matching a set of observational characteristics of respondents in treatment and control groups- in the form of predicted probabilities (propensity score)- estimated through logistic regression analysis. The fundamental assumptions of this approach are: i). Conditional on the propensity score, assignment to the treatment and control groups can be taken to be random, and ii). For each value of the covariate, there is a definite probability of being falling into treatment or control group (assumption of common support condition). If these assumptions are satisfied then the differences in outcomes between treatment and control groups can be directly compared to estimate the effect of “treatment”, known as treatment effect of treated (ATT).[39, 46] Additionally, the average treatment effect on the untreated (ATU) was through comparing the differences in outcomes between controlled and matched treated group shows the effect of exposure to the controlled (unexposed) group. Average treatment effect (ATE) further combines the above two average effects (ATT and ATU) to measure the overall change in outcome indicators (self-reported STI prevalence and treatment seeking behaviour). Mostly due to exposure variable of interest (experience of physical violence) by weighing them with the proportion of respondents in treatment and control groups respectively.[43]

The radius caliper method with replacement was used for matching purpose. This matching approach uses all the possible comparison group members within the maximum distance from caliper to reduces the risk of using poor matches.[48] To improve the quality of matching the common support restriction (to exclude data from exposed with a higher propensity score than that of any unexposed person) was imposed. The region of common support was (0.0392, 0.8761) depicting a fairly good matching of cases based on selected characteristics of FSWs namely: age, marital status, literacy, residence, co-residents, source of income other than sex work, savings, alcohol use, and other sex work characteristics (except client volume and condom use). The statistical differences in the outcome measures among the FSWs belonging to “treatment” and “control” group, i.e. ATT, were measured by the bootstrap standard error (100 replications) of the estimates in regression analysis.[38–40, 43, 46] While explaining the results, we considered only those estimates significant at p<0.10 in the matching analysis.

## Results

**Fig 1:**
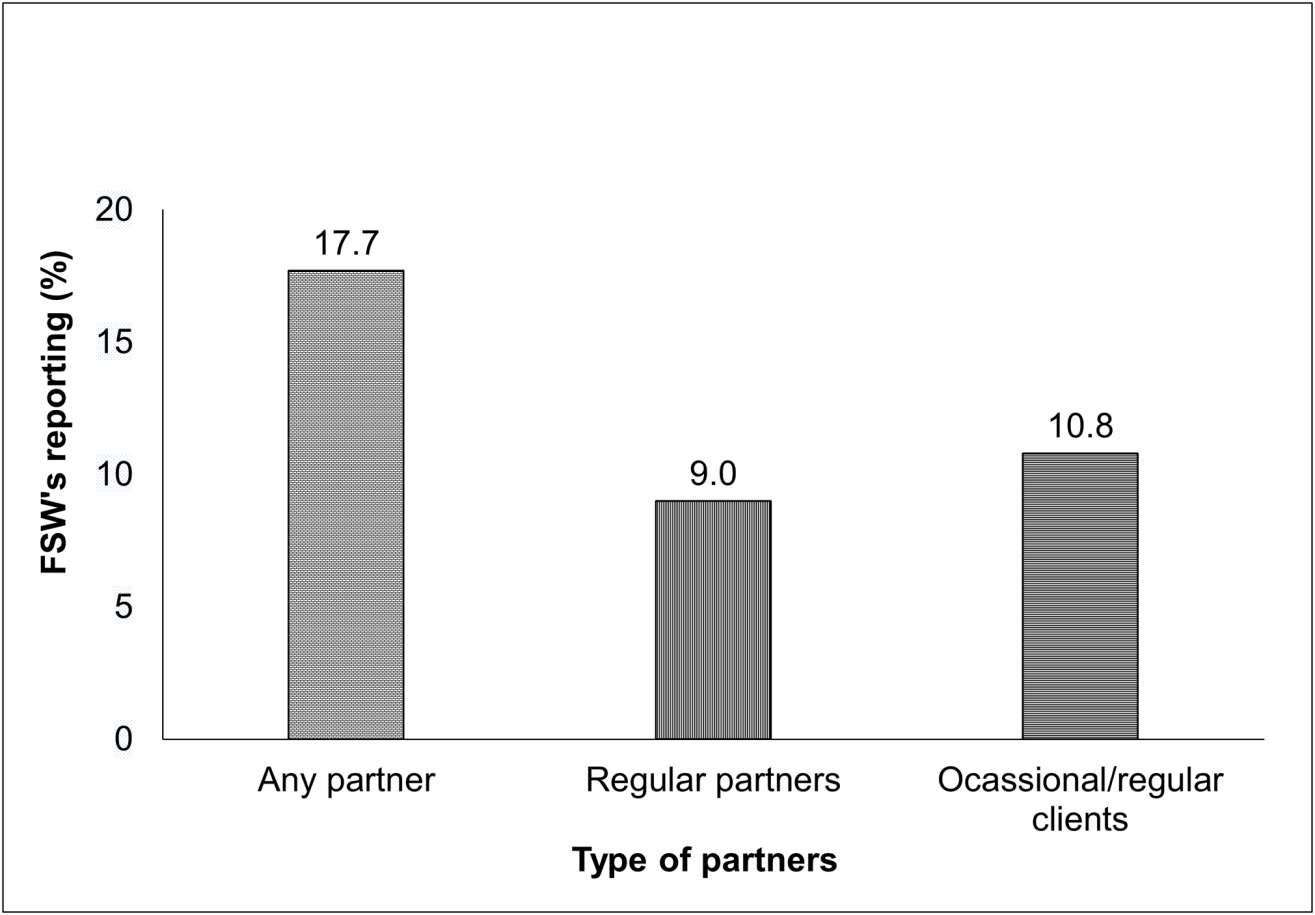
Percentage of FSWs experienced physical Violence by type of partners, Thane, 2014. Self-reported physical violence perpetrated by partners of FSWs in Thane district. Partners were classified into regular partners and occasional/regular clients for the purpose of this study. Regular partners are non-paying partners of the FSW such as husband or boy-friend or lover. Occasional or regular clients do pay for having sex with the FSW.

Of 2,785 FSWs interviewed, little less than one-fifth of them (18%) experienced physical violence perpetrated by their regular partners (husband/boyfriend/lover) or their regular/occasional clients (Fig 1). While nearly 11% FSWs experienced physical violence from occasional or regular clients, about nine percent reported violence perpetrated through their regular partners.

Differential in socio-demographic, economic and sex work related characteristics of FSWs by the experience of physical violence are presented in Table 1. Findings show that the characteristics of FSWs experiencing violence and those who did not experience violence were almost similar on many fronts. For example, women in both groups had mean age of about 30 years, about 86% were ever married, more than half of them were literate (52% vs. 57%, respectively), around 82% were migrants, i.e., belonged to a place outside Thane/Mumbai, were residing with spouse or any family members (about 74%), had experience of sex work trade of 3 or more years, and about 81% were exposed to ongoing HIV prevention programme in the district. The key characteristics of FSWs that were significantly different from the group of FSWs experiencing violence versus those who did not mostly dealt with the indicators of their economic status and those reflecting their sex work and behavioural characteristics. For instance, significantly higher proportion of women experiencing violence had other sources of income than sex work and had savings compared to the FSWs, who did not experience physical violence (47% vs. 32% and 19% vs. 12%, respectively; p<0.001). Moreover, the proportion of FSWs consuming alcohol were significantly higher in the group who experienced violence compared to their counterparts who did not report any physical violence at the time of survey (41% vs. 32%, respectively; p<0.001).

**Table 1:** Proportion of FSWs experienced physical violence by selected background characteristics (Thane, 2014).

Background and sexual behaviour variables	Total	Experienced physical violence (%)	Did not experience physical violence (%)	Significance
N	2,785	492 (17.7)	2,293 (82.3)	-
***A*. *Background characteristics***				
Mean age (SD)	30.2 (5.6)	30.6 (5.4)	30.1 (5.6)	
Marital status				
Ever married (%)	86.1	88.4	85.7	
Literacy				
Literate (%)	56.3	52.4	57.1	
Residence				
Non-localite (%)	82.3	80.5	82.6	
Co-residents				
Reside with spouse/family members (%)	73.6	76.4	73.0	
Source of income other than sex work (%)	36.8	47.2	34.6	***
Have savings (%)	13.2	18.7	12.0	***
Consume alcohol (%)	33.8	41.3	32.2	***
***B*. *Sex work characteristics***				
Age at first sex				
15 or more years (%)	85.7	90.2	84.4	**
Duration in sex work				
3 or more years (%)	91.1	93.4	90.5	
Place of solicitation				
Public places (%)	19.1	33.7	16.0	***
Weekly client volume				
10 or more clients (%)	16.8	22.3	15.6	**
Have regular partner (%)	55.5	69.5	52.5	***
Exposed to HIV intervention (%)	80.6	81.1	80.5	
Consistent condom use with regular partners’^#^ (%)	26.9	23.6	38.9	***
Consistent condom use with occasional/regular clients (%)	77.1	74.8	87.6	***

^#^ Among those who had a regular sexual partner.

** Differences by exposure to physical violence are statistically significant at p≤0.05.

*** Differences by exposure to physical violence are statistically significant at p≤0.001.

Differentials in sex work characteristics of FSWs by their violence experiences suggest that reporting of physical violence was comparatively higher among FSWs, who had entry into sex work at relatively later age (15 or above). In addition to that, significantly larger proportion of FSWs among those experienced physical violence were found to be soliciting for their clients in public places (34%), had 10 or more clients per week (22%), and reported having a non-paying regular partner (70%) than their respective counterparts who had not experienced physical violence either by their regular partners or occasional/regular clients. Corresponding estimates were 16% for place of solicitation and weekly client volume. About 53% FSWs who did not experience physical reported to have a regular partner at the time of survey (Table 1). Findings also depicted that the consistent condom use with the regular partners, and occasional/regular clients was significantly lower among FSWs reported physical violence than those who did not experience so.

The proportion of FSWs reported STIs and seeking behaviour by their experiences of violence is presented in Table 2. Findings of descriptive analysis suggest higher prevalence of at least one STI symptom or multiple STI symptoms and low level of uptake of treatment for the last STI symptom they had in 12 months period preceding the survey among FSWs who experienced violence. For instance, the prevalence of any STI was 50% vs. 41% (p<0.001) and multiple STIs were 36% and 29% (p<0.001) among FSWs experienced violence compared to their counterparts who did not experience any violence, respectively. Of the FSWs who reported any STI symptom in last 12 months, just 28% received treatment for the last STI treatment among the FSW experienced violence compared to 36% FSWs did not report any form of physical violence by their regular partner or clients. The difference in treatment seeking was statistically significant at 5% level of significance.

**Table 2:** Proportion of FSWs reported experience of STIs and treatment seeking behaviour by experience of violence (Thane, 2014).

Outcome variables	Total	Experienced physical violence (%)	Did not experience physical violence (%)	Significance
N	2,785	492 (17.7)	2,293 (82.3)	-
Reported any STI symptom in last 12 months				
No	59.3	50.0	61.3	
Yes	40.7	50.0	38.7	***
Reported multiple STI symptoms in last 12 months				
No	71.3	64.2	72.8	
Yes	28.7	35.8	27.2	***
Sought treatment for the last STI symptom^#^				
No	70.3	71.6	64.2	
Yes	29.7	28.4	35.8	**

^#^ Among those self-reported any STI symptoms.

** Differences in outcomes by exposure to physical violence are statistically significant at p≤0.05.

*** Differences in outcomes by exposure to physical violence are statistically significant at p≤0.001.

The study estimates the effect of physical violence on self-reported any STI symptom, multiple STI symptoms and treatment seeking behaviour among FSWs by the estimated difference in the outcomes, between the treated (exposed to physical violence) and the matched control (unexposed to physical violence) groups using matching analysis. As mentioned before, due to its’ computing strengths, this method allows to analyze the actual impact of the treatment on outcomes very precisely. The potential effect of violence on the group of FSWs who did not experience violence, i.e., if they would have experience such violence what would be the effect on the outcomes, is also presented. Table 3 shows that the average treatment effect (ATE) of violence on any symptom was 12.1%, on multiple STI symptoms was 9.3% and on treatment seeking behaviour was about 10% (negative). This means the experience of physical violence among FSWs has led to an increase the prevalence of any STI and multiple STIs while a decrease in the likelihood of obtaining treatment for the last STI symptom that they had.

**Table 3:** Estimated effect of violence on experience of STIs and treatment seeking: Propensity score matching analysis (Thane, 2014).

Outcome variables	Treated^$^ (%)	Matched controls^#^ (%)	ATT (%)	ATU (%)	ATE (%)	Significance^¥^
Reported any STI symptom in last 12 months	49.2	37.9	11.3	12.3	12.1	**
Reported multiple STI symptoms in last 12 months	33.7	26.2	7.5	9.8	9.3	*
Sought treatment for the last STI symptom^±^	24.2	34.5	(-)10.3	(-)9.9	(-)10.1	**

^±^ Among those self-reported any STI symptom.

^$^Treated (exposed to physical violence).

^#^Matched control (unexposed to physical violence).

ATT- Average treatment effect among treated; ATU- Average treatment effect among untreated; ATE- Average treatment effect.

^¥^ Significance level for ATT:

***p≤0.001;

**p≤0.05;

*p≤0.10.

The adjusted treatment effect of treated (ATT), i.e., effect of violence on the FSWs who experienced the same, demonstrated an increase in the risk of any STI by 11% (p<0.05), and on multiple STIs by 7.5% (p<0.10) whereas reduction in chances of obtaining the treatment by 10.3% (p<0.05). When we compared the average treatment effect on treated (ATT) and average treatment effect on untreated (ATU) (Table 3), considering the experience of physical violence as an exposure variable, we did not find differences between ATT and ATU except the fact that the magnitude of ATU was slightly higher than ATT. It suggests that the FSWs who did not experience physical violence, if they would have been exposed to any such violence, the chances of reporting STI would be at a higher level while seeking treatment for last STI at the lower level.

### Quality assessment of results obtained in Table 3

The critical step in using matching methods is to diagnose the quality of the resulting matched samples. The quality of results was assessed on the five parameters, namely, common support (% of variables discarded due to unavailability of matched sample), balancing test (comparison of significance of mean differences between unmatched and matched sample before and after treatment), identification of substantial overlap of the propensity score distributions in the two groups (treated and control) mostly presented through bar diagram, assessment of the covariate balance in the matched groups, and assessment of overall significance of the model by comparing the significance of pseudo- R2 between treated and untreated groups.[39, 45, 50–52]

Table 4 shows the post matching the common support and suggests that the number of dropped FSWs due to common support were minimal. For example, while comparing the FSWs did not experience violence with that experienced violence, of the 2,661 observations, 93 cases were excluded. While 82 cases were excluded from the untreated group, 11 cases were excluded from the treated group, resulting in a sample of 2568 observations. Thus, of the total matched sample, less than five percent (93/2,661) were discarded due to the unavailability of any matched sample on a set of defined characteristics. The second parameter of the quality assessment was the balancing test. Table 5 shows mean values of each variable before and after matching in both treated and untreated group, the level of bias before and after matching for each variable, and percentage reduction in bias after performing the matching. The differences between matched pairs were evaluated using the t-test. As seen in Table 1, before matching, the FSWs in treated (experienced physical violence) and control group (did not experience physical violence) were significantly different in terms of selected economic and sex work-related characteristics, however, results of balancing test clearly revealed that the mean differences of all the covariates became insignificant after performing the matching. The covariates were adequately balanced and, therefore, the matched sample of FSWs did not vary from each other in terms of their socio-demographic, economic and sex work related characteristics.

**Table 4:** Common Support.

Physical violence vs. no violence	Sample size		
Treatment assignment	Off support	On support	Total
Untreated	82	2,093	2.175
Treated	11	475	486
**Total**	**93**	**2,568**	**2,661**

**Table 5:** Balancing test: Post matching comparison of means in matched and unmatched groups and percentage of bias reduced.

Variable	Unmatched/Matched	Mean		%bias	% bias reduction	t-test	
		Treated	Control			t	p> | t |
Age (mean)	U	31.5	29.9	28.6		5.040	0.000
	M	31.3	31.4	-1.8	93.6	-0.260	0.794
Marital status (Ever married)	U	0.879	0.867	3.7		0.630	0.527
	M	0.879	0.871	2.3	36.7	0.330	0.745
Literacy (Literate)	U	0.526	0.523	0.8		0.140	0.892
	M	0.526	0.541	-3.1	-298.8	-0.430	0.666
Residence (Non-localite)	U	0.781	0.834	-13.5		-2.420	0.015
	M	0.781	0.789	-2.0	85.4	-0.260	0.794
Co-residents (Reside with spouse/family member)	U	0.771	0.731	9.3		1.600	0.111
	M	0.765	0.729	8.3	10.0	1.160	0.248
Other source of income	U	0.491	0.353	28.1		4.970	0.000
	M	0.479	0.492	-2.6	90.6	-0.360	0.720
Have savings	U	0.199	0.063	41.2		8.290	0.000
	M	0.180	0.186	-1.6	96.2	-0.190	0.853
Consume alcohol	U	0.418	0.360	12.0		2.110	0.035
	M	0.415	0.361	11.1	7.5	1.550	0.122
Age at fist sex (mean)	U	17.0	17.1	-0.6		-0.090	0.927
	M	17.0	17.1	-1.1	-87.7	-0.150	0.885
Duration in sex work (mean)	U	7.2	8.1	-17.3		-2.940	0.003
	M	7.3	7.3	-0.8	95.5	-0.120	0.906
Typology (Public places)	U	0.340	0.180	37.1		6.870	0.000
	M	0.332	0.345	-3.0	92.0	-0.380	0.705
Have regular partner	U	0.693	0.552	29.2		5.000	0.000
	M	0.686	0.642	9.1	68.8	1.290	0.197
Exposed to HIV intervention	U	0.791	0.858	-17.8		-3.240	0.001
	M	0.796	0.760	9.5	46.6	1.210	0.227

**Fig 2:**
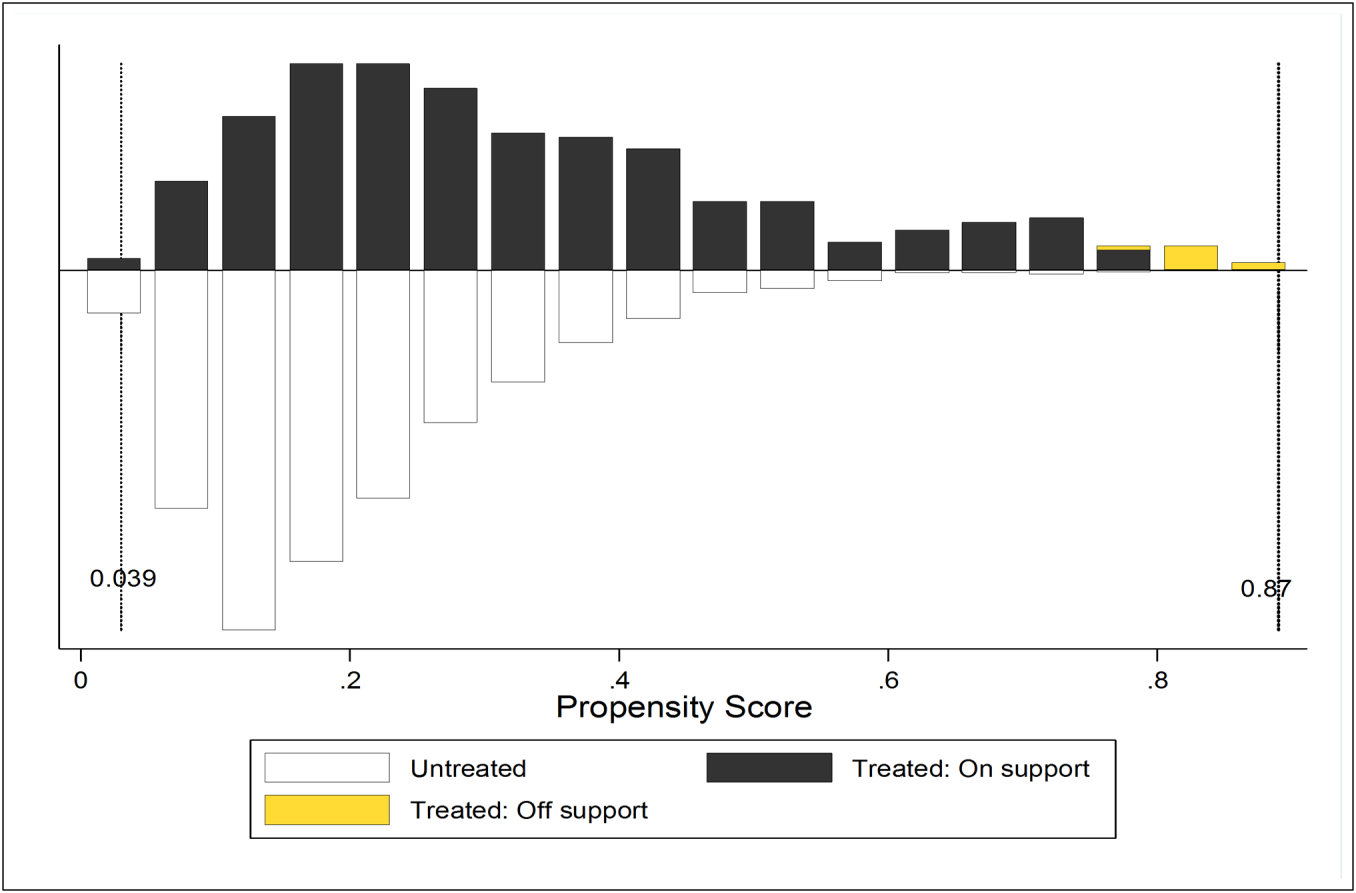
Predicted probability of experiencing physical violence: matched sample, Thane, 2014. The bar diagram demonstrates extent of overlap of the propensity score distributions in the two groups (treated and untreated FSWs), above and below the x-axis. The radius caliper method with replacement was used for matching purpose. This matching approach uses all the possible comparison group members within the maximum distance from caliper to reduce the risk of using poor matches. The region of common support was (0.039, 0.87) depicting a fairly good matching of cases based on selected characteristics of FSWs.

To demonstrate the extent of overlap of the propensity score distributions in the two groups, a bar diagram above and below the lines (x-axis) shows the propensity score for treated and untreated FSWs, respectively (Fig 2). This graph compares the quality of matching by analyzing the distribution of propensity of scores for FSWs experienced violence and those who did not experience so. As observed in Fig 2, the distribution of FSWs above and below the line (x-axis) was more or less identical after matching on propensity scores. The existence of substantial overlap between the characteristics of treated and untreated FSWs confirms the validity of the common support assumption. The overall quality of the model was assessed through pseudo-R^2^. [45] Table 6 shows that after the matching the pseudo-R^2^ had become insignificant, indicating the fact that there is no systematic difference in the distribution of covariates between treated and untreated groups.

**Table 6:** Significance of overall model.

Sample	Pseudo R^2^	LR chi^2^	p>chi^2^
Unmatched	0.085	156.94	0.000
Matched	0.007	7.82	0.855

## Discussion

This study aims to estimate the extent of violence faced by the FSWs and the differential in the characteristics of women experiencing violence as compared to those who did not report violence in Thane District. The study also attempts to comprehend the linkages between violence, self-reported STI symptoms, and treatment seeking behaviour by comparing the experiences of two groups of FSWs. Though, in the Indian context, evidence on this issue is fairly available, due to methodological limitations they often failed to estimate precisely the net effect of violence on desired STI-related outcomes. The robust matching approach was adopted to assess the impact of violence on STI and treatment seeking among FSWs in the intervention district (Thane) from where evidence on this issue is rarely available. Below sections present a detailed discussion of the study findings.

This study demonstrated an association between physical violence and demographic indicators for FSWs. The FSWs, who reported a source of income other than sex work and had savings, were more likely to report the experience of violence. A randomized control trial among FSWs in Mongolia demonstrated how high levels of financial responsibility and limited bargaining power increase the risk for physical violence, and HIV/STIs.[53] Study findings suggested that most of the FSWs were the primary financial providers for their households as sex work was often constituting the major source of household income. FSWs were using an array of earning strategies to fulfill the financial needs of them and other dependents.[46] Women who reported savings and parallel engagement in other work along with sex work were more likely to be facing the economic hardship. To secure the financial needs of the family, they often compromise from negotiating safer sex with partners in exchange for more money and elevate their risk for HIV and STIs.[46] Findings in Indian context do reveal economic insecurity and debt as contributing factors to the violence among FSWs. For example, FSWs who need funds to pay debts may be more likely to work in multiple riskier contexts that increase their vulnerability to violence. A study from Andhra Pradesh revealed that sex work had increased FSWs vulnerability to violence and risk of HIV by agreeing to have sex with more than one client at an unknown location to make more money.[54]

Our study findings elucidate that those FSWs who solicit the clients from public places were more vulnerable to experience physical violence than those of their counterparts (34 vs. 16, p≤0.001). Aastha programme, implemented in Thane during 2004–2009, also found that FSWs, who were soliciting sex with the clients at streets, were found to be more vulnerable to physical violence than the FSWs of other typologies. Street based FSWs mainly belonged to the lowest socio-economic strata and was at the mercy of local *goons*, *pimps* and the police, who were the main perpetrators of violence.[55] Moreover, physical violence among female sex workers was also found to be strongly and independently associated with consumption of alcohol. These results were in line with the previous research findings from Africa and China which showed a higher physical violence reported by FSWs who drink alcohol with their clients.[27, 56] Data from Thane district also suggests that physical violence significantly increases the chances of experiencing STI (any or multiple) by 8% to 11% and reduces the chances of seeking treatment for the STI-related symptoms by about 10% compared to their counterparts of almost similar characteristics.[28]

Research from India and South Africa demonstrated a strong relationship between intimate partner violence and women’s risk of contracting STI/HIV.[19, 57, 58] Even studies from many high- and low-income country settings have found that women who are HIV-positive are more likely to experience physical and sexual violence compared to HIV-negative women.[22, 23] Physical violence may increase women’s vulnerability to HIV and STIs, through direct and indirect pathways–for example: physical violence perpetrated by regular partners makes it difficult for women to refuse sexual intercourse or negotiate condom use [11, 23, 28, 59]; experience of physical and sexual abuse among FSWs at early ages may increase rates of high-risk sexual behaviour later in life, including chances of having multiple partners, less condom use, multiple partners, and experiencing subsequent violence.[28, 60] Fear of violence may prevent women from seeking HIV prevention and clinical services, including services for preventing the transmission of HIV to infants.[23, 58]

Our findings also corroborate with above results that found associations between being a victim of violence and non-condom use; which could be related to women’s inability to negotiate safe sex with clients or regular partners leading to increased chances of experiencing STI and HIV. [11, 61–63] There are higher chances of violence among the women having high client volume or their male partners being involved in sex with multiple partners.[60]

Table 7 shows the proportion of FSWs consistently using a condom with their regular partner or occasional/ regular clients and reporting the prevalence of STIs by their violence experiences. Findings clearly show that, given the condition that FSWs are using the condoms consistently with their regular partners/clients, chances of reporting one or multiple STI symptoms was significantly higher among the FSWs who experienced violence (50% and 36%, respectively) than those who did not experience any physical violence in 12 months period preceding the survey (reported prevalence of any STI and multiple STIs- 41% and 29%, respectively). Moreover, findings from Table 1 clearly depicted that FSWs experiencing violence were less consistent in condom use with their regular and occasional partner/client as compared to FSWs did not experience any physical violence; which were further in line with the studies observing association between perpetration of physical violence and inconsistent condom use with regular partners [11, 61] and occasional/regular clients.[64]

**Table 7:** Proportion of FSWs reported consistent condom use with regular partner/ clients and reported symptoms of any STI or multiple STIs by experience of violence (Thane, 2014).

Outcome variables	Any self-reported STI symptom (%)	Self-reported multiple STI symptoms	N
Experience of violence			
No	40.5	28.7	2128
Yes	50.0	35.6	486
Significance	***	**	2785

** Differences by exposure to physical violence, controlling for consistent condom use, are statistically significant at p≤0.05.

*** Differences by exposure to physical violence, controlling for consistent condom use, are statistically significant at p≤0.01.

We acknowledge that, although, we made considerable efforts to analyze the relationship between violence and STI related outcomes of interest for a major geographic region of India carrying a significant potential risk of HIV spread; our study does have certain limitations. For example, while an attempt was made to ensure, as far as possible in a cross-sectional study, appropriate temporal ordering, we acknowledge its limitations in asserting causality. There is a need for more longitudinal studies or, at least, cross-sectional studies that are better equipped to provide histories of both violence as well as STI-related outcomes. Second, there might be under-reporting of physical violence, especially those perpetrated by the regular partners. Besides these, the information on sexual violence was not collected in the study. Therefore, the study could not demonstrate the overall level of violence among the FSWs in Thane district. As sexual violence has a relatively larger direct effect on the prevalence of STI, mostly through forced sex.[23] The relationship between physical violence and prevalence of STI could be ascertained only through inconsistent condom use. Third, the survey did not include any concrete information to measure the empowerment level of FSWs in the study area. Analysis of the experience of violence, the level of empowerment and treatment seeking for the STI symptoms could have added more strength to the analysis. Moreover, the intensity of violence, in terms of number of times harassed or physically beaten, and its’ relationship with the prevalence of self-reported STIs and treatment seeking behaviour would have been an important indicator of analysis. However, this information was not included in the analysis as information on the frequency of violence in last twelve months was obtained from the FSWs at the time of the survey and thus responses on frequency might be affected by recall bias. Lastly, the outcomes on STI are based on the self-reported symptoms and are not based on clinically tested cases. Despite these limitations, our study, for the first time in the context of Thane district, provides scientific information on linkages between violence, the prevalence of STIs and treatment seeking behaviour among FSWs and hold an important implication for the program planning and implementation.

## Conclusion

Violence has immediate effects on women’s health, which in some cases, could even be fatal. Physical and mental health consequences can also have a sustained effect even after the violence has stopped.[65] The results of this study demonstrate that those FSWs who experienced physical violence are more likely to report STI symptoms and inconsistent condom use. Such violence also limited the opportunities for women to seek care for the last STI symptoms that they had. Violence related challenges in HIV prevention program must be addressed to reduce the risk of STIs and increase the access of tailored STI prevention and care services. To further elucidate the strength of the association between physical violence and prevalence of STI symptoms among FSWs, there is a need for high-quality follow-up studies conducted in different geographical regions of the India and elsewhere, and among FSWs of diverse racial/cultural backgrounds and varying levels of STI/HIV risks.

## Supporting Information

S1 Doc Operational Guideline under NACP III for Targeted Intervention Vol I (Core group), NACO. (PDF)

S1 Fig Percentage of FSWs who experienced physical violence by type of perpetrators, Thane, 2014. FSWs reported physical violence was perpetrated by various perpetrators in Thane district. The key perpetrators of physical violence were regular partners (husband or boy-friend or lover), occasional or regular clients including the stranger, local goons (local criminal or violent person in the community), police, and others in Thane district.
(TIF)

S1 Table Results of multivariate logistic regression analysis. (DOCX)
