# Supplementary material for: Effect of Physical Violence on Sexually Transmitted Infections and Treatment Seeking Behaviour among Female Sex Workers in Thane District, Maharashtra, India
Source: PLoS One. 2016 Mar 2;11(3):e0150347. doi: 10.1371/journal.pone.0150347 (PMC4774990; doi:10.1371/journal.pone.0150347)
Supplement: S1 Doc — (PDF) [file pone.0150347.s001.pdf]

Targeted Interventions Under NACP III

OPERATIONAL GUIDELINES

Volume I

CORE HIGH RISK GROUPS

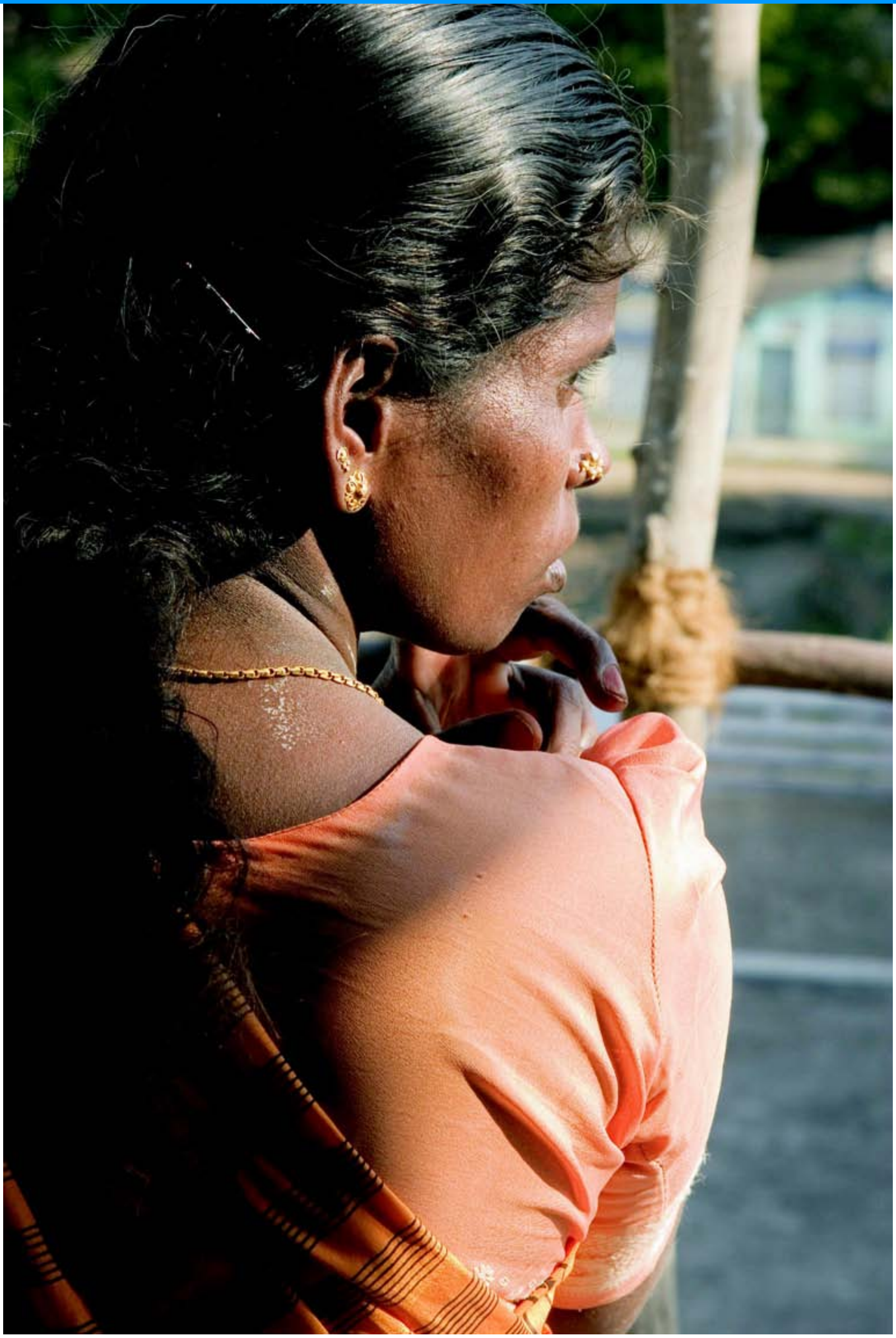

NACO

National AIDS Control Organization
Ministry of Health & Family Welfare
Government of India

Targeted Interventions
Under NACP III

OPERATIONAL GUIDELINES

Volume I

CORE HIGH RISK GROUPS

NACO

National AIDS Control Organization
Ministry of Health & Family Welfare
Government of India

© October 2007

National AIDS Control Organization
Government of India

FOREWORD

K.Sujatha Rao

Additional Secretary & Director General

National AIDS Control Organization, Ministry of Health & Family Welfare, Government of India

The prevention of new infections in high risk groups is a major thrust in National AIDS Control Programme III. The most effective means of controlling the spread of HIV in India is through the implementation of Targeted Interventions (TIs) amongst persons most vulnerable to HIV/AIDS, such as female sex workers (FSWs), men who have sex with men (MSM) and transgenders (TGs) and injecting drug users (IDU). In addition, the bridge populations of truckers and migrants also require focused interventions. Both NACO and the States place a high priority upon full coverage of the States' FSWs, MSMs/TGs, IDUs and migrants/truckers with TIs. In order to standardise the approach to scaling up coverage among these core groups and bridge populations and maintain a high level of quality, it is important to provide detailed information on various operational issues in TIs.

NACO has prepared these Operational Guidelines after a series of consultations with Technical Resource Groups (TRGs), representatives of civil society, Government, core groups, donors and other stakeholders. The guidelines describe the operational details of TI projects with various core high risk groups (Part 1) and bridge populations (Part 2). The guidelines also provide detailed information on issues related to programme management, services required in terms of human resources, infrastructure, linkages and monitoring and evaluation indicators for each programme area.

I take this opportunity to acknowledge the contribution made by the TRGs, the TI Team of NACO and the NACO Technical Support Unit (TSU) in preparing these guidelines. I would also like to acknowledge and thank the various agencies mentioned in the acknowledgments section for their valuable inputs.

We hope that these guidelines will help State AIDS Control Societies, potential partners (NGOs, CBOs, and networks), programme managers and other staff working in TI projects and TSUs to implement and manage TI projects more effectively.

Let the scale up challenge begin!

9th Floor, Chandralok Building, 36 Janpath, New Delhi-110001 Phone: 011-23325331 Fax: 011-23731746
E-mail: asdg@nacoindia.org

TABLE OF CONTENTS

Foreword iii

Acknowledgements vii

Introduction 1

Chapter 1
Introduction to Targeted Interventions for Core High Risk Groups under NACP III 3

Chapter 2
Operationalising Targeted Interventions for FSWs/MSMs/TGs: Guidelines for SACS, TSU and DAPCU 19

Chapter 3
Operationalising Targeted Interventions for FSWs/MSMs/TGs: Guidelines for NGOs 53

Chapter 4
Operationalising Targeted Interventions for IDUs: Guidelines for NGOs 93

Chapter 5
Development of CBOs and Community Led Responses 137

LIST OF ANNEXURES

Annexure	Title
**Annexure 1**	Broad Mapping
**Annexure 2**	Site Assessment
**Annexure 3**	Peer Educator Training
**Annexure 4**	Peer Progression
**Annexure 5**	Peer Led Outreach and Planning
**Annexure 6**	Dialogue-Based Interpersonal Communication By and With HRGs
**Annexure 7**	Crisis Response System
**Annexure 8**	Community Committees
**Annexure 9**	Power Analysis
**Annexure 10**	Programme Management
**Annexure 11**	MSM: Orientation, Identity and Vulnerability to HIV
**Annexure 12**	Excerpt from Infosem's 'Strategic Plan for Scaling Up Interventions for MSM and Transgender Populations in India'
**Annexure 13**	Modular Costing Framework for IDU TIs
**Annexure 14**	Staffing and Running a Drop-In Centre
**Annexure 15**	Universal Precautions and Post-exposure Prophylaxis
**Annexure 16**	Developing a BCC Strategy and IEC Materials for IDUs
**Annexure 17**	Application Form for Accreditation to Run OST Services
**Annexure 18**	Checklist for Scoring Proposals to Run OST Services
**Annexure 19**	Quality Assurance Protocol
**Annexure 20**	Indicators Scoring Sheet
**Annexure 21**	Site Selection Scoring Sheet

NACO Guidelines and Tools Referenced in these Guidelines

NGO/CBO Guidelines, March 2007

Guidelines on Financial and Procurement Systems for NGOs/CBOs, March 2007

STI Guidelines

ACKNOWLEDGEMENTS

Th e following organisations and individuals are acknowledged for their work which is quoted or used in adapted versions in the text of the Guidelines and the Annexures.

FSW/MSM/TG Guidelines (Chapters 1-3)

- Bill & Melinda Gates Foundation
- CARE
- Catalyst Management Services
- Family Health International (FHI)
- Humsafar Trust
- INFOSEM (*Strategic Plan for Scaling Up Internventions for MSM and TG Populations*, Naz Foundation International, Humsafar Trust, in consultation with MSM/TG CBOs and community leaders, supported by DfID)
- University of Manitoba/Karnataka Health Promotion Trust
- Lawyers Collective
- PATH
- RCSHA
- Sangama, Bangalore
- Tamil Nadu AIDS Initiative, Voluntary Health Services
- UNAIDS

IDU Guidelines (Chapter 4)

- Bill & Melinda Gates Foundation
- Dr. Suresh Kumar
- Dr. Samiran Panda
- Jimmy Dorabjee
- Dr Arup Chakraborty
- SHARAN Project Network
- UNODC, ROSA, New Delhi
- National Drug Dependence Treatment Centre, All India Institute of Medical Sciences (NDDTC, AIIMS)
- Society for Promotion of Youth and Masses (SPYM)
- NACP III Design Documents
- Society for Community Intervention and Research, (SCIR) Kolkata
- The NACO Costing Guidelines

- Australian International Health Institute
- Emmanuel Hospital Association
- SPYM
- Rusan Pharma Hand Book for Doctors
- DOH International
- The Centre for Harm Reduction (Macfarlane Burnet Institute),
- Mahesh Nathan and the West Bengal Project Support Unit (A unit of Mott MacDonald India)

CBO Development (Chapter 5)

- Dr. S. Jana, (West Bengal)
- Dr. Sushena Reza-Paul (Bangalore)
- Dr. Sundar Sundararaman (Chennai)

NACO Technical Support Unit for Targeted Interventions

- Aparajita Ramakrishnan, Team Leader, NACO Technical Support Unit (The Bill & Melinda Gates Foundation)
- Nandinee Bandyopadhyay, Targeted Interventions Team, NACO Technical Support Unit (PATH)
- Senthil K. Murugan, Targeted Interventions Team, NACO Technical Support Unit (University of Manitoba)
- Amit Shrivastav, Targeted Intervetnions Team, NACO Technical Support Unit (Family Health International)

NACO Technical Resource Group for FSW/MSM/TG

- **Chairperson**: Ms. Sujatha Rao, Additional Secretary & Director General, National AIDS Control Organization, Ministry of Health & Family Welfare
- **Co-Chair:** Dr. P. Krishnamurthy, Project Director, AIDS Prevention and Control Project (APAC-VHS)
- Ms. Sanghmitra Iyengar, SAMRAKSHA, Bangalore
- Dr. Smarajit Jana, NACO
- Mr. Ashok Row Kavi, Hamsafar Trust, Mumbai
- Mr. Arif, Naz Foundation International, Luknow
- Mr. Shiv Kumar, Director, SWASTI, Bangalore
- Mr. Ashok Alexander, Bill & Melinda Gates Foundation
- Dr. Sushena Reza-Paul, Director, Direct Intervention, KHPT, Mysore
- Dr. James Blanchard, IHAT, Rajasthan
- Mr. Alankar Malviya, CHARCA, UNDP
- Representative of Department of Rural Development, GOAP
- Mr. Manoj Gopalakrishnan, CEO, HLLFPPT, New Delhi

NACO Technical Resource Group for IDUs

- Chairperson: Dr. Rajat Ray, Professor & Chief National Drug Dependence & Treatment Centre Department of Psychiatry, All India Institute of Medical Sciences, New Delhi
- Dr. A. Jayanta Kumar President, Galaxy Club
- Dr. M. Suresh Kumar, SAHAI Trust
- Mr. L. Birenderajit Singh Secretary Social Awareness Service Orgnisation (SASO)
- Dr. Rajesh Kumar Director, Society for Promotion of Youth & Masses (SPYM)
- Mr. Mahesh Nathan, West Bengal State Management Agency
- Mr. Debashis Mukerjee Co-ordinatorThe Calcutta Samaritans P-14, CIT Road, Kolkata-700014
- Mr. Luke Samson, SHARAN, New Delhi
- Ms. Ashita Mittal, UNODC
- Mr. Sateyndra Prakash Director, National Institute for Social Defence(NISD) Ministry of Social Justice & Empowerment, New Delhi
- Dr. B. Langkham, Director, Emannuel Hospital Association
- Dr. Samiran Panda, Consultant- UNODC
- Ms. Kathleen Kay, Family Health International, New Delhi
- Professor (Dr.) B.M. Tripathi, Department of Psychiatry, National Drug Dependence & Treatment Centre, All India Institute of Medical Sciences, New Delhi
- Dr. Mohd. Shaukat, Joint Director (Blood Safety & IDU), National AIDS Control Organisation
- Mr. Rajesh Nair, National AIDS Control Organisation Ministry of Health & Family Welfare

INTRODUCTION

The purpose of these guidelines is to ensure the delivery of quality HIV prevention interventions through the Targeted Interventions (TIs) under the National AIDS Control Programme (NACP III) in India. The guidelines outline standardised operational procedures for implementing comprehensive HIV prevention services.

According to the framework of NACP III, prevention strategies will have a three-pronged approach:

1. **Core High Risk Groups (HRGs)**: There are three core HRGs — female sex workers (FSWs), high risk men who have sex with men and transgenders (MSM and TGs), and injecting drug users (IDUs). Through the TIs under NACP III these populations receive a comprehensive package of preventive services. State AIDS Control Societies (SACS) will be expected to saturate coverage of these groups before moving on to cover other groups.

2. **Bridge populations, with particular focus on clients of sex workers**: Clients receive a combination of services including condom promotion, referrals to clinical services for STI management and behaviour change communication (BCC). Specific strategies have been outlined to approach two major populations within the bridge population: **truckers** and **high risk migrants**.

3. **Other Vulnerable Populations**: Risk groups in rural areas, HIV affected children, youth 15-19 years old and women receive a package of services delivered through a more extensive mechanism – that of link workers. This is discussed in the section on link workers within the NACP III Project Implementation Plan (PIP) Chapter 5 and the NACO Operational Guidelines for Link Workers.

This document is an operational guideline for Targeted Interventions (TIs) with the three core HRGs (FSWs, MSM/TGs and IDUs) under NACP III (approach no. 1 above). Guidelines for bridge populations such as truckers and migrants are outlined in Volume Two of these Operational Guidelines.

CHAPTER 1

Introduction to Targeted Interventions for Core High Risk Groups Under NACP III

TABLE OF CONTENTS

1.1 RATIONALE FOR AND DEFINITION OF TARGETED INTERVENTIONS (TIs)

1.1.1 Female Sex Workers (FSWs)

1.1.2 Men Who Have Sex With Men (MSM) and Transgenders (TGs)

1.1.3 Injecting Drug Users (IDUs)

1.2 HIGH RISK GROUPS – DEFINITIONS AND TYPOLOGIES RELEVANT FOR INTERVENTIONS

1.2.1 Typologies of Female Sex Workers (FSWs)

1.2.2 Typologies of High Risk Men Who Have Sex with Men (MSM) and Transgenders (TGs)

1.2.3 Typologies of Injecting Drug Users (IDUs)

1.3 INTERVENTION PACKAGE FOR HIGH RISK GROUPS COVERED UNDER TIs

1.3.1 Outreach and Communication

1.3.2 Services

1.3.3 Creating an Enabling Environment

1.3.4 Community Mobilisation

1.4 RATIONALE FOR CBOs

1.5 TRAFFICKING AND NACP III

1.1 RATIONALE FOR AND DEFINITION OF TARGETED INTERVENTIONS (TIs)

It is estimated that more than 90% of HIV transmission in India is related to unprotected sexual intercourse or sharing of injecting equipment between an infected and an uninfected individual. Not everyone in the population has the same risk of acquiring or transmitting HIV. Much of the HIV transmission in India occurs within groups or networks of individuals who have higher levels of risk due to a higher number of sexual partners or the sharing of injection drug equipment.

These **core high risk groups** (HRGs) of individuals who are most at risk include:

- Female sex workers (FSWs)
- High risk men who have sex with men (MSM), and transgenders (TGs)
- Injecting drug users (IDUs)

Note: In Volume I, HRGs refers only to CORE HRGs (FSWs, MSM/TGs, IDUs), and not to truckers/migrants.

The broader transmission of HIV beyond these HRGs often occurs through their sexual partners, who also have lower-risk sexual partners in the "general" population. For example, a client of a sex worker might also have a wife or other partner who is at risk of acquiring HIV from her higher-risk partner. Individuals who have sexual partners in the highest-risk groups and other partners are called a "bridge population", because they form a transmission bridge from the HRG to the general population. This is illustrated in Figure 1.1, which shows how HRG members or HRGs have many sexual partnerships with different bridge population members, who in turn have at least one partner in the general population.

Given this pattern of epidemic transmission, it is most effective and efficient to target prevention towards HRG members to keep their HIV prevalence as low as possible, and to reduce transmission from them to the bridge population.

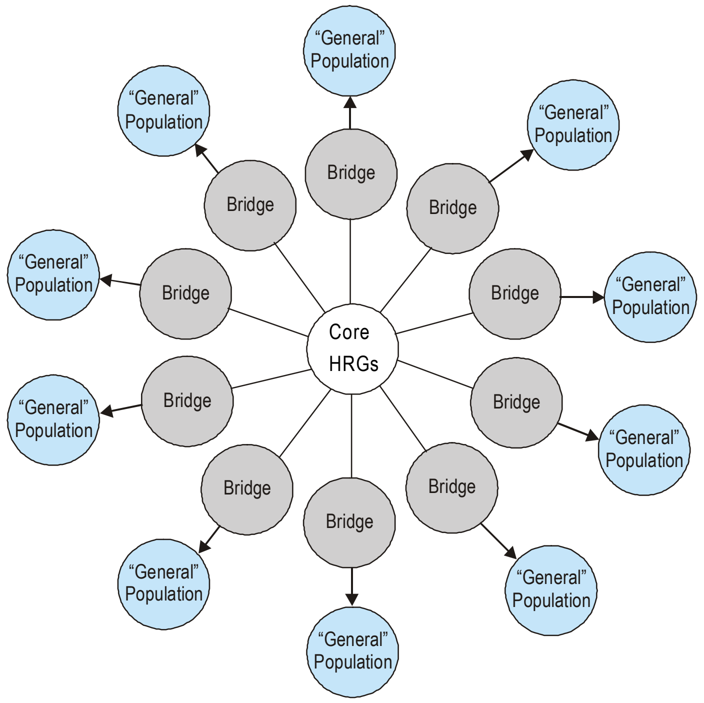

Figure 1.1 Illustration of an HIV Transmission Network

1.1.1 Female Sex Workers (FSWs)

FSWs have many sexual partners concurrently. Generally, full-time FSWs have at least one client per day, or at least 30 clients per month, and nearly 400 per year. Some FSWs have more clients than others, having several clients per day and 100 or more clients in a month. The higher risk of FSWs is reflected in a substantially higher prevalence of HIV among them than in the general population. In India, Sentinel Surveillance data has shown that HIV prevalence is generally 10-20% or more, which is more than ten times higher among FSWs than among pregnant women attending antenatal clinics.

The relative importance of FSWs as a HRG can be summarized by estimating the number of sexual contacts occurring between FSWs and clients. Within one year, 1,000 FSWs will have sexual contact with 300,000 to 1,000,000 clients. In contrast, 1,000 "high risk" men who have 6-12 sexual partners in a year will have a total of 6,000-12,000 sexual partners in a year. Since the HIV prevalence is much higher among FSWs, a higher proportion of their sexual partnerships could result in HIV transmission. As illustrated in Figure 1.2, the number of HIV positive sexual contacts for 1,000 FSWs is much greater than for the same number of high risk men. This demonstrates the strategic importance of focusing prevention programmes on FSWs.

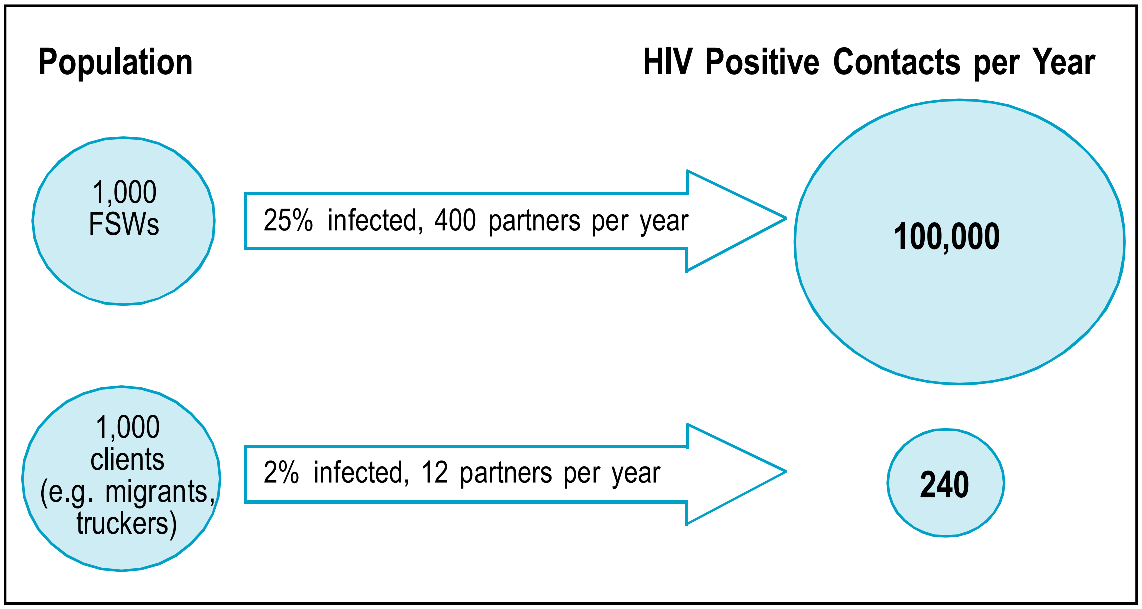

Figure 1.2 Number of HIV Positive Contacts for 1,000 FSWs and High Risk Men

1.1.2 Men Who Have Sex with Men (MSM) and Transgenders (TGs)

MSM/TGs are another important HRG who are highly vulnerable to HIV and are also a strategically important group for focusing HIV prevention programmes. It is important to know that not all MSM have many sexual partners, and are therefore not at a substantially increased risk for HIV compared to others. However, there are MSM sub-populations which do have high rates of partner change as well as high number of concurrent sexual partners, and those that often engage in anal sex with multiple partners are at particularly high risk, since HIV is more transmissible through anal sex than by other sexual practices. Members of the transgender population who have many male partners are also at high risk, since many of them engage in anal sex. Because many men who have sex with high-risk MSM and transgendered individuals also have other partners, both male and female, targeted interventions for these HRGs are strategically critical to controlling the HIV epidemic.

For more information see Annexure 11.

Tool

Annexure 11 ***MSM: Orientation, Identity and Vulnerability to HIV***

1.1.3 Injecting Drug Users (IDUs)

IDUs are a third HRG for which targeted interventions are of critical importance. HIV is highly transmissible through the sharing of needles and other injection equipment, so it can spread very rapidly within networks of IDUs who share injecting equipment with each other. Once HIV prevalence is high in the IDU population, it can expand quickly into their sexual networks. Some IDUs are also sex workers, which can quickly link HIV transmission in the IDU networks to transmission in the larger high-risk sexual networks.

It is important to recognise that, like sexual transmission of HIV, HIV is essentially preventable among IDUs and their sex partners too. Interventions that are implemented early (HIV prevalence <5% among IDUs) are most effective in halting the spread of the HIV epidemic among IDUs. HIV interventions targeting the majority of IDUs can stabilise and even reverse the escalating HIV epidemic among them. HIV positive IDUs receiving opioid substitution treatment (OST) not only helps them to avoid injecting but also to adhere to anti-retroviral treatment (ART) as well as other treatments.

In summary, the HIV transmission dynamics in India are such that unless effective targeted HIV prevention saturates the most at-risk HRGs of FSWs, MSM/TGs and IDUs, the epidemic will not be controlled. But the positive implication of this is that if HIV prevention is successful in these HRGs, the epidemic will be substantially curtailed.

1.2 HIGH RISK GROUPS – DEFINITIONS AND TYPOLOGIES RELEVANT FOR INTERVENTIONS

1.2.1 Typologies of Female Sex Workers (FSWs)

For the purpose of TIs, a female sex worker (FSW) is an adult woman who engages in consensual sex for money or payment in kind, as her *principal* means of livelihood.

In any given geography, sex workers are not a homogeneous group. Sex workers can be categorised into 6 main typologies, based on where they work and more specifically on where they recruit or solicit clients and not where they live or actually entertain the clients. The major typologies of FSW in India are described below. The accompanying chart shows the distribution of these typologies among sex workers in southern India.

1. **Street-based** sex workers are those who **solicit clients** on the street or in public places such as parks, railway stations, bus stands, markets, cinema halls. They may **live** in a brothel and may entertain their clients in a lodge, car, truck, hotel room, at the client's home, in a cinema or in a public place.
2. **Brothel-based** sex workers are those whose clients contact them in recognised brothels, that is buildings or residential homes where people from outside the sex trade know that sex workers live and work. This includes sex workers in Kamathipura in Bombay and Sonagachi in Calcutta, and also smaller scale brothels in Districts such as Sangli, Bagalkot and Guntur. Typically, a brothel is a place where a small group of sex workers is managed by a Madam (*gharwali*) or an agent. Usually the sex worker pays a part of her earnings to the *gharwali*.
3. **Lodge-based** sex workers are those who reside in what is known as a lodge (a small hotel) and their clients are contracted by the lodge owner, manager or any other employee of the lodge on the basis of sharing the profits. These sex workers do not publicly solicit for clients.
4. **Dhaba-based sex workers** are those who are based at dhabas (roadside resting places for truckers and other long-distance motorists) or road-side country motels. Like lodge-based sex workers, these sex workers do not publicly solicit clients, but rather are accessed by clients who come to these locations. In some cases, dhaba-based sex workers are also contracted by the dhaba owners and could move from dhaba to dhaba based on their contracts.

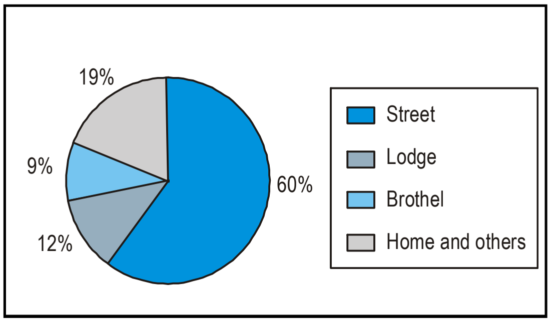

5. **Home-based** or **"secret"** sex workers operate usually from their homes, contacting their clients on the phone or through word of mouth or through middle-men (e.g. auto drivers). Generally, they are not known to be working as sex workers within their neighbouring areas. In fact, they could have an entirely different "public" identity – e.g. housewife, student. While many sex workers operate "secretly" given the level of harassment, violence and stigmatisation they experience from the police, the rowdies and the members of general public, for the purpose of TIs, the term "secret" sex worker refers to a specialised category of sex workers, as explained above. They are only "secret" or "anonymous" in terms of their identity in their immediate context (e.g. family, neighbourhood) – not in terms of accessibility to programmes or their clients.

> Sex workers can be categorised into six categories **based on where they *work* (i.e. recruit clients) and *not* where they live or actually entertain the clients**. Programmes that attempt to reach out to sex workers in their residences can be problematic, especially if the sex worker is "anonymous" at her home and practises sex work without the knowledge of her family.

6. **Highway-based sex workers** are those who recruit their clients from highways, usually from among long distance truck drivers.

There are other sex workers whose primary occupational identity may vary, but a large proportion of their occupation group, *but not all*, often engages in commercial sex regularly and in significant volumes. Bar girls, Tamasha artistes and Mujra dancers come under this category.

The categories used here are often overlapping and fluid. For example, a sex worker may be street-based for some time and then go into a contract with a lodge owner to become-lodge based. Or a brothel-based sex worker may move to another town or city temporarily and work as a street-based sex worker. **For the purposes of mapping and designing TIs we must categorise sex workers according to their primary identity and terms of engagement in the sex trade.**

> **Risk varies with typology**
> It is important to note that certain typologies (brothel- and lodge-/dhabha-based sex workers) tend to have higher client volumes than home-based sex workers, and they therefore have a higher risk profile, requiring special focus even within the category of female sex workers. New entrants into these categories also warrant special focus.

1.2.2 Typologies of High Risk Men who have Sex with Men (MSM) and Transgenders (TGs)

The term "men who have sex with men" (MSM) is used to denote all men who have sex with other men as a matter of preference or practice, regardless of their sexual identity or sexual orientation and irrespective of whether they also have sex with women or not. Coined by public health experts for the purpose of HIV/STI prevention, this epidemiological term focuses exclusively on sexual practice. This term *does not* refer to those men who might have had sex with other men as part of sexual experimentation or very occasionally depending on special circumstances. It should be noted that not all of those who engage in male-to-male sex do not necessarily identify themselves as homosexuals or even men.

There are several sub-groups among MSM. For the purposes of TIs, these groups are defined as below.

- **Hijras:** *Hijras* belong to a distinct socio-religious and cultural group, a "third gender" (apart from male and female). They dress in feminine attire (cross-dress) and are organised under seven main *gharanas* (clans). Among the *hijras* there are emasculated (castrated, *nirvan*) men, non-emasculated men (not castrated, *akva/akka)* and inter-sexed persons (hermaphrodites). While one sub-set of *hijras* is involved in blessing and gracing during births, marriages and ceremonies, another is involved in begging, and a third group is involved in sex work. For the purposes of TIs, *hijras* are covered under the term "transgenders" or TGs.
- **Kothis:** The term is used to describe males who show varying degrees of "femininity" (which may be situational), take the "female" role in their sexual relationships with other men, and are involved mainly – though often not exclusively – in receptive anal/oral sex with men. Some proportion of Kothis has bisexual behaviour and many may marry a woman. Self-identified hijras may also identify themselves as kothis. Many kothis assume the gender identity of a woman.
- **Double Deckers**: *Kothis* and *hijras* label those males who both insert and receive during penetrative sexual encounters (anal or oral sex) with other men as *Double Deckers*. These days, some proportion of such persons also self-identify as *Double Deckers*. Some equivalent terms used in different States are *Double*, *Dupli-Kothi* (West Bengal) and *Do-Paratha* (Maharashtra).
- **Panthis:** The term *panthi* is used by *kothis* and *hijras* to refer to a "masculine" insertive male partner or anyone who is masculine and seems to be a potential sexual (insertive) partner. Some equivalent terms used in different States to denote masculine insertive partners are *Gadiyo* (Gujarat), *Parikh* (West Bengal) and *Giriya* (Delhi).

Not all MSM/TGs are at equal risk

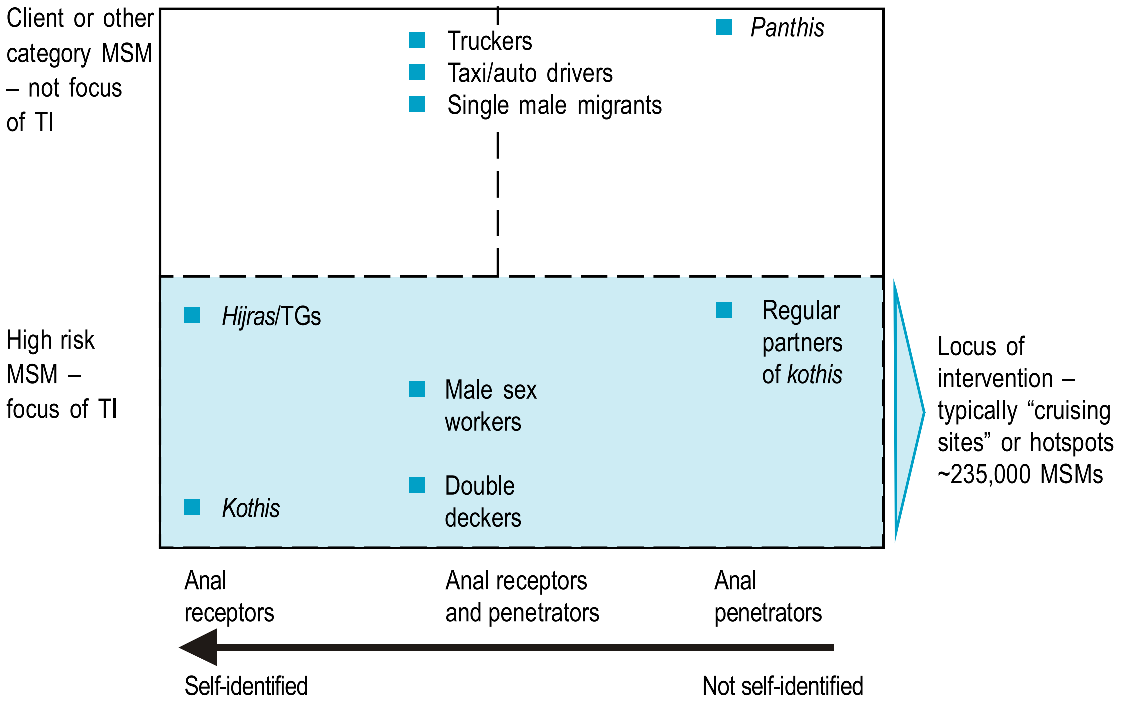

Do MSM have sex with women?

Various sexual behaviour studies have shown that MSM are also involved in sexual relationships with women.

- The national BSS study showed that **31% of MSM reported having sexual intercourse with a female partner** in the 6 months prior to the survey, and the mean number of female partners was 2.4.
- Data from Andhra Pradesh show that **65% of MSM had ever had sex with women, among which 76% was with their wife, 29% with FSW and 13% with wife as well as FSW.**
- Community studies from Mumbai confirm the above findings that the female partners of MSM were primarily their wife, but about **18% had more than one female partner.**

Among those who are defined as MSM, only some MSM are most at risk. In this document, the term MSM will refer **only** to those high-risk MSM/TGs who are included as HRGs in the TI efforts, i.e. those who may be self-identified and anal receptors with multiple sexual partners. **These groups are *hijras*, *kothis* and double deckers (not *panthis*).**

1.2.3 Typologies of Injecting Drug Users (IDUs)

IDUs are not injectors at all times in their injecting life-span. They may inject, then fall back into non-injecting (e.g. oral) drug use, or abstinence, and then return to injecting. Thus IDUs are defined as those who used any drugs through injecting routes **in the last three months.**

In addition to addressing IDUs, IDU programmes should ensure that they also address the **regular sexual partners of IDUs**, as many of them are likely to be infected, and some of them may be IDUs too.

It is equally important to remember that some IDUs might be sex workers or MSM, and some of them are also female.

1.3 INTERVENTION PACKAGE FOR HIGH RISK GROUPS COVERED UNDER TIs

Targeted interventions for HRGs offer a "package" of services which are detailed further in the operational guidelines. This package of services varies for each major HRG, but broadly follows the components outlined below.

1.3.1 Outreach and Communication

Peer-led, NGO-supported outreach and behaviour change communication.

a. Differentiated outreach based on risk and typology
b. Interpersonal behaviour change communication (IPC)

1.3.2 Services

Promotion of condoms, linkages to STI (sexually transmitted infection) services and health services with a strong referral and follow-up system.

a. Promotion/distribution of free condoms and other commodities (e.g. lubricants for MSM, needles/syringes for IDUs)
b. Provision of basic STI and health services (including abscess management and oral substitution therapy for IDUs and also oral/anal STI services for MSM/TGs)
c. Linkages to other health services (e.g. for TB) and voluntary counselling and testing centres (VCTC)
d. Provision of safe spaces (drop-in centres or DICs)

1.3.3 Creating an Enabling Environment

a. Advocacy with key stakeholders/power structures
b. Crisis management systems
c. Legal/rights education

1.3.4 Community Mobilisation

Building community ownership of the TI's objectives ("community" refers here to the HRGs: FSWs, high risk MSM and IDUs).

a. Collectivisation
b. Creation of a space for community events
c. Building capacity of FSW, MSM and IDU groups to assume ownership of the programme

1.4 RATIONALE FOR CBOs

The NACP III design aims to strengthen the processes of community-led and community-owned TIs (where "community" refers to HRGs). The rationale for this is based on several observations:

- When the community defines HIV prevention as part of its own agenda, uptake of services and commodities is higher than when services are "imposed" upon it.
- Community-led interventions leverage the existing organic bonding among community members so that individual HRG members take an interest in supporting their colleagues in accessing both information and services. This leads to rapid and saturated coverage of the FSW, MSM/TG and IDU communities.
- On many occasions, community based organisations (CBOs) are found to be most effective in scaling up HIV prevention programmes. The Sonagachi project started in 1992 and was subsequently handed over to the FSW CBO Durbar Mahila Samanwaya Samiti (DMSC) in 1999. Soon after that, this organisation was able to expand to 15 red light Districts in the State of West Bengal in a span of two years, increasing the coverage of the FSW population in the State to a level of 75%-80%.
- Community-led initiatives allow members of the community to enable HRGs to play the role of a pressure group as consumers to maintain and reinforce quality of services, leading to sustained demand for high-quality services.
- Sustainability of a programme depends among other things on the level of ownership by the community. For example, the FSW CBO in Bangladesh, Durjoy, even after the withdrawal of the donor's support, was able to sustain basic minimum services with its own organisational resource base.

Beyond HIV, there are several other examples of CBOs developing strong scaled programmes. Community mobilisation of poorer women through micro-credit has helped them to gain more control over their own lives (e.g. BRAC and Grameen Bank in Bangladesh, SEWA in India). These organisations have improved the quality of life of thousands of women, in addition to providing economic security to the family through the process of institutionalisation of community ownership building.

Thus, NACP III offers a set of guidelines specifically to address the strengthening of CBOs and building of new CBOs (both from scratch and from existing NGOs).

1.5 TRAFFICKING AND NACP III

Since its inception, the National AIDS Control Programme (NACP) has accorded priority to preventing and controlling HIV among populations at greater risk, which include inter alia sex workers. Targeted interventions (i.e. provision of risk reduction measures such as information, condoms, treatment for STIs) for high risk groups (FSWs, MSM, IDUs) will remain the mainstay of the response under NACP III. The programme recognises that stigma and marginalisation experienced by high risk groups amplify risks and limit their ability to protect themselves and others. Therefore, NACP III aims to empower high-risk groups to enable improved negotiation and health seeking. Creation of an enabling environment and community mobilisation are the key programmatic strategies to address such vulnerability.

NACP notes that structural determinants such as poverty, gender inequality and lack of viable opportunities compel many persons, particularly girls and young women, into commercial sex. Further, many are forced or fraudulently brought into sex work. NACO and its affiliate State AIDS Control Societies (SACS) cannot and will not support NGOs and CBOs which encourage the compelling of persons into sex work. NACO and SACS affirm the principle of voluntary entry and exit from sex work. NACO, in partnership with other Ministries, will seek to address fundamental conditions that contribute to involuntary entry into for sex work. Simultaneously, at project sites, targeted interventions will help institute community mechanisms to prevent involuntary sex work.

For persons in sex work, NACP will promote health and occupational safety by promoting use of condoms, providing access to STI and other treatment and encouraging voluntary HIV counselling and testing. NGOs implementing targeted interventions for sex workers and MSM will proactively assist persons opting out of sex work through collaborative arrangements with women's groups, Women's Commission and the Ministry of Women and Child Development. At the same time, NACP will not interfere with the rights of those choosing to remain in sex work. Targeted interventions will promote active involvement of sex workers in all aspects of project development, implementation and evaluation.

CHAPTER 2

Operationalising Targeted Interventions for Core High Risk Groups: Guidelines for SACS, TSU and DAPCU

TABLE OF CONTENTS

2.1 MAPPING HRGS – GEOGRAPHIC MAPPING, SIZE ESTIMATION AND SITE ASSESSMENT

2.1.1 Background
- A. Mapping in the context of HIV intervention
- B. Objectives
- C. Guiding principles
- D. Steps
- E Involvement of TIs

2.1.2 Organisations Involved in Mapping
- A. Mapping TRG and NHRGCs
- B. State or regional organisations
  - Role of organisations
  - Selection criteria
  - Selection and contracting procedure
- C. Summary chart

2.1.3 Methodologies of Mapping
- A. Methodology for review of available information
  - Objective
  - Who will do it?
  - Sources of information
  - Process
  - Expected outputs
- B. Methodology for broad mapping
  - Objective
  - Who will do it?
  - Sources of information
  - Process
  - Expected outputs
- C. Methodology for site assessment
  - Objective
  - Who will do it?
  - Sources of information
  - Process
  - Expected outputs

2.2 ANALYSING THE COVERAGE AND QUALITY OF CURRENT TIs AMONG HRGs

2.2.1 Analysing Existing TIs

2.2.2 Criteria for TI Allocation

2.2.3 TI Unit Size

2.2.4 Geographic Distribution of TIs

2.3 RECRUITMENT, CAPACITY BUILDING AND PROGRAMME MANAGEMENT

2.3.1 Recruiting NGOs/CBOs/Networks to Implement TIs

2.3.2 Capacity Building Plan for NGOs and CBOs Implementing TIs

2.3.3 Programme Monitoring

2.3.4 Programme Management

A. Objectives of programme management

B. Role of State AIDS Control Society (SACS)

C. Role of Technical Support Unit (TSU)

D. Role of Non-Governmental Organisations (NGOs)

E. Principles of CMIS for TIs

F. Timelines and key indicators

2.3.5 Financial Management

Steps in Mapping and NGO Selection

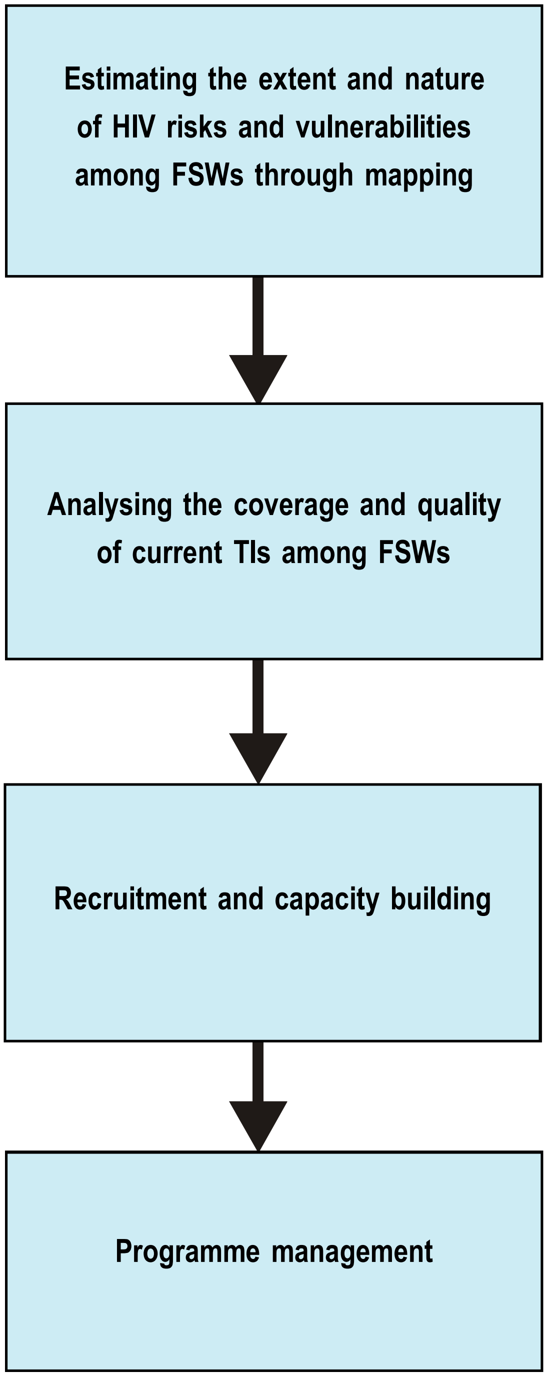

- Objectives of mapping/assessment
- Guiding principles
- Methodologies
- Organisations involved

- Analysing existing TIs
- Criteria for TI allocation
- TI unit size
- Geographic distribution of TIs

- Recruiting NGOs/CBOs/networks
- Capacity building for NGOs/CBOs

- Organisational roles
- Principles of CMIS
- Financial mangement

2.1 MAPPING HRGs – GEOGRAPHIC MAPPING, SIZE ESTIMATION AND SITE ASSESSMENT

2.1.1 Background

A. Mapping in the context of HIV intervention

Mapping, in the context of NACP III TIs (and of this document), refers to the following three exercises:

1. Review of secondary data
2. "Broad mapping" to estimate size, identify HRG typology and locations of risk
3. "Site assessment" to derive basic insights into factors that make HRGs particularly vulnerable to HIV, and to initiate interventions

HRGs will be "mapped" in each State in two distinct phases:

1. In the **first phase** mapping is to be carried out:
   - Where TIs addressing HRGs are in operation
   - Any other areas where TIs are not in operation but HRGs are known to be present in significant numbers
2. The **second phase** of mapping is implemented when SACS and TSUs identify major geographic areas in the State which have been left out of TI coverage. This could occur through a review of TI data against State geography. The objective of mapping in the second phase is to ensure that such gaps in coverage are "mopped up" through commissioning of new TIs or reconfiguration of existing TIs. Mapping in the second phase will follow the same methodologies as in the first phase.

These guidelines describe mapping in the **first phase**.

Key Terms

- A geographical area demarcated by a definite boundary (e.g. town, city, village) is referred to as a **"site"**.
- Areas within a site where there is significant concentration of HRGs are referred to as "**hotspots"**. Within hotspots, HRGs may solicit, cruise, and interact with other HRG members, or have sex or share injecting drugs.

The overarching goal of mapping HRGs is to **put appropriate and effective interventions in place**. Therefore it is important to remember:

1. Mapping must be **rapid –** based on its results the TIs have to be designed and services have to reach these populations urgently.
2. Those who are mapping HRGs must know **how to find them**; must be **credible** and **acceptable** to them; and most importantly, must be **respectful** towards the norms, practices and rights of HRGs. This is because many of these HRG members are hard to reach or hidden or physically scattered. The stigma, discrimination and violence they experience from mainstream society often make them even more inaccessible, as they are usually reluctant to share personal information with outsiders.
3. Methodologies must be **usable and HRG-friendly**.

B. Objectives

Identify or confirm locations within the States and Districts where TIs ought to be placed to reach those HRGs who are most vulnerable

Targeted interventions will address only those MSM and IDUs who are most at risk, namely kothis, hijras, double deckers and IDUs who share injecting equipment. Therefore only these subcategories of MSM and IDUs will be mapped and not the whole universe of MSM and IDUs.

Validate estimates of size

- Generate estimates of the size of HRGs in each site, by different categories
- Provide locations of hotspots where HIV risk activities predominantly take place
- Generate information to help understand the mobility patterns of HRGs within and outside the site
- Explore the HIV/STI risks that HRGs face and the vulnerability factors that exacerbate such risks
- Characterise the HRGs to facilitate subsequent programming
- Identify their HIV related needs, existing HIV interventions and key gaps

Begin the process of mobilising HRG groups for HIV/STI prevention

- Build awareness of HIV
- Increase knowledge about risk reduction strategies
- Increase knowledge about existing HIV/STI prevention interventions for HRGs
- Build social capital and solidarity amongst HRGs – a collective voice
- Explore safe and private spaces for HRGs to meet and work together
- Build a core group of HRG members from the site who will serve as an important resource for project implementation by recruiting and training local HRG members to implement mapping

C. Guiding principles

Pay attention to definitions of HRGs (including subcategories)

We must clearly define the HRGs we are mapping. Otherwise, those conducting the exercise will not know whom to count and whom to leave out. For example, for the purposes of the TI, who is a sex worker, and when is a sex worker classified as street-based? Who are MSM and who among them are kothis (or regular and casual partners of kothis or double deckers)? Whom do we define as an IDU?

Please refer to Chapter 1 for details on how different HRGs and their subcategories are defined under NACP III.

Use members of HRGs to map

- Experience shows that if HRGs themselves are recruited to conduct the mapping the **results will be closer to reality.** It is also important to recruit onto the mapping teams representatives from all subcategories of HRGs that are available in a site – since brothel-based sex workers, whose operations are restricted to a particular brothel in a town, may not know where street-based sex workers solicit their clients. A hijra may not necessarily be able to relate to a kothi. A city-based IDU may not know where IDUs hang out in more rural settings.
- Since mapping involves getting people to provide sensitive information about their sexual behaviours, their partners, locations, networks, etc., HRG members are more likely to share information without fear or prejudice if it is solicited by people from the same group, who are **acceptable** and **credible** to them.
- When HRG members do the mapping, the **process of mapping becomes an intervention in itself** – it mobilises local HRG communities to understand and address HIV risks and creates a demand for HIV services. So while mapping is going on the intervention simultaneously gets underway.

Of course, like all other researchers, the HRG members must be trained to do the mapping and will also require administrative and technical support during the entire mapping process. This additional support will be provided by non-HRG members in the mapping team.

Some critical criteria for selecting people who will do the mapping are that they be:

- True peers of and represent different categories of HRGs in the areas being mapped
- Acceptable and credible to HRGs
- Known in the site and know the site well
- Motivated to work with their peers on HIV/STI risk reduction
- Available to follow the entire mapping process from training, field level implementation, feedback and analysis of data, to dissemination. This means they must agree to take time off from their regular occupations for a considerable period of time at a stretch, for which they will be financially compensated.

Mapping implementers who are true peers of HRG participants will find the safest space and best time to facilitate mapping activities with them. They also have the necessary acceptability and credibility among HRGs to effectively mobilise them and to generate in-depth, accurate information for project design.

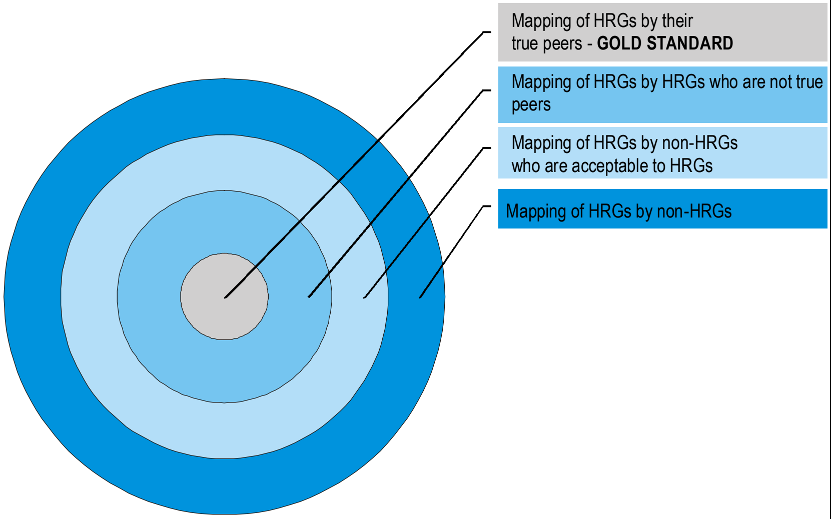

Effectiveness of mapping by HRGs

Gather information from multiple sources

Triangulation is a critical component of any mapping exercise. Information gathered from one source needs to be verified against information from other sources. In order to triangulate the data, it is important to have multiple sources of information for mapping:

1. **Primary Key Informants** are members of HRGs and their sexual partners. For example, sex workers and their clients, people who inject drugs and their sexual partners, a *kothi* and his *panthis*.
2. **Secondary Key Informants** are those who are part of or close to the HRG members' occupational or sexual lives or their addiction practices. For example, suppliers of injecting drugs and equipment, pimps, agents, brokers, madams, hotel workers, shopkeepers near risk sites, auto rickshaw drivers plying routes near sex sites.
3. **Tertiary Key Informants** are those with a good idea about the HRGs at a town/District/State level. For example, NGOs, government officials, pharmacy owners, local journalists.

It is advisable **not** to consult groups that are known to have adversarial relationships with the particular HRGs, such as rowdies and goondas, as this might jeopardise local HRG members' trust in the mapping exercise or cause them actual harm.

A rule of thumb

Although secondary or tertiary stakeholders often know something about HRGs, they never have as full or the same picture as HRGs themselves. Therefore, in any mapping exercise **more than 60% of respondents** – that is people who are consulted for seeking information – **must be from HRGs that are being mapped**.

Understand the limitations of the mapping process

Mapping is not formal research or ethnographic study. So the information it generates can (a) be limited to informing the design or review of HIV interventions; and (b) be site-specific and therefore not generalisable to other sites. Keep in mind that size estimates are just that – they are not an exact headcount of individual HRG members. **There is constant turnover/mobility of some HRGs – estimates arrived at from mapping must be regularly revised and updated through the course of TI implementation.**

Do no harm – be ethical

As mapping is an integral part of NACP III, it must be implemented in a way that reflects and reinforces the core values and approaches of NACP III, ensuring the well-being and protecting the rights and interests of HRGs. While mapping it is important to remember the secret, socially marginalised, and also formally criminalised status of most HRGs and the practices that they engage in. To protect HRG participants in mapping the following key guidelines are to be followed:

- Do not breach confidentiality of HRG members
- Seek the consent of HRG members before involving them in mapping
- Be prepared to handle negative consequences of mapping for the HRGs – have a harm redress plan
- Do not raise false expectations (e.g. promise services, jobs or remuneration)

Steps to ensure protection of HRGs during mapping

1. Access to HRGs may require going through various gatekeepers such as employers, brothel owners or suppliers. Mapping teams will hold discussions with gatekeepers and clarify the purpose of the mapping, e.g. size estimation, service providers to HRGs, places where HRG members operate and obtaining information to guide design of HIV interventions or improved implementation of ongoing projects. Gatekeepers will be made aware that all information gathered by the mapping team will be kept anonymous and confidential and will not be shared even with them.
2. Specific efforts will be taken to inform the NGOs working with the populations covered by the mapping, as well as community leaders, about the purpose, risks and benefits of the mapping.
3. The mapping is anonymous. No names or personal identifiers will be recorded. Mapping teams and others associated with mapping must ensure that mapping records are kept secure throughout the mapping process and after.
4. Mapping teams will have to take witnessed verbal consent from each participant before they involve her/him in the process. All mapping documents and information will be labelled in such a way that the participants remain anonymous. Prior to implementation of any mapping procedure or method, those who are implementing it will explain the mapping procedures in detail to potential participants, and answer all questions to the full satisfaction of the participants. The mapping team will emphasise that participation is voluntary and should participants decide not to participate or withdraw from the procedure at any time, their decision would not affect any services from the NGO or the clinic that they would normally receive.
5. Mapping teams, SACS, TSU and NACO will closely monitor the consent procedure through spot checks.
6. Discussions will be held between SACS, TSU, NACO, the mapping team and local NGO/ CBO staff and community leaders on potential use of information for programming when the mapping is complete or before any dissemination of mapping data.
7. Implementers of mapping will adopt stringent measures to ensure that participation in mapping does not expose HRG members to any risk or cause them any harm. However, it is also essential to spell out what specific steps would be taken to mitigate the harm that HRG participants *might* still be exposed to, despite such precautionary measures, and how the HRG participants will be supported by the implementers of mapping following such incidence of harm.

These steps are necessary not just to mitigate any harm caused materially (such as money to compensate for loss of work or other support such as legal aid, safe custody, etc.), but also to establish that NACP III respects the rights and entitlements of HRG participants and acknowledges that any harm to them ought to be substantively redressed.

D. Steps

Steps	Programmatic Objective	Where to be Implemented?	Output Expected	Time frame
1. **Review of available information**	To determine the sites where mapping will be carried out	At State level	List of sites to be mapped<br>■ All sites where there are TIs<br>■ Sites where there are no TIs but obvious and/or reported concentrations of HRGs such as cities/towns, trading centres, religious centres	2 Weeks
2. **Broad mapping and feedback and analysis of data**	To determine where to place the TIs (which District)	At site level	■ List of reported hotspots<br>■ Size estimation by subcategories<br>■ HIV/STI serives available for HRGs	4-12 weeks, depending on size of the State
3. **Site assessment including feedback, data analysis**	To determine the site-specific design of TIs	Within a site at hotspot level	■ Confirmed list of hotspots in the site<br>■ Fine-tuning of estimated numbers by subcategory in the site<br>■ Mobility pattern of HRGs<br>■ Availablity of HRGs<br>■ Risk profile of HRGs	6-12 weeks

The shaded area represents activities which are a part of TI processes.

E. Involvement of TIs

As far as possible NGOs and CBOs implementing TIs should be engaged in the process of mapping: they should understand the process and realise why such close participation of HRG members is critical for successful mapping. Only if they know how and why information is being gathered will they trust the information and use it for shaping their intervention design. In addition, involvement in the mapping process enables NGOs, CBOs or networks running TIs to fully understand the complex realities of the lives of HRG members. Witnessing the competence with which HRG members implement mapping also convinces NGOs of their full potential as partners in interventions.

While TI involvement in mapping is the ideal, TIs are likely to be in different stages of maturity, and coverage of HRGs by TIs may vary from State to State. As a rule of thumb:

1. Where a TI has been in operation for some time and has an extensive and effective intervention programme, the NGO/CBO running the TI should ideally be involved in all steps of mapping. However, it is recommended that HRG members who are not being paid or working with the existing TI (as peer educators, outreach workers or in any other paid or voluntary position) be selected to do the mapping. Of course, if the TI in question has mapped the HRGs they work

with **with substantive participation of HRGs themselves**, and the data they have is reliable and credible, there is no need to do mapping again in their operation site/s.

2. Where TIs have been commissioned but the intervention is new, mapping will be done as the first step of the intervention, and if necessary the design, location or composition of particular TIs will be reconfigured based on the mapping information.
3. In areas where HRG presence is reported but TIs are not yet in place, mapping needs to be carried out first, and TIs are then to be contracted depending on the numbers of HRG members present. Since it takes considerable time to advertise for, select and contract TIs, advertisements can be placed based on the information generated through the review, and TIs can be selected from those who apply, based on the information from Broad Mapping.

Steps	Involvement of TIs
1. Review of available information	Existing TIs to be consulted
2. Broad Mapping	Ideal but not imperative
3. Site Assessment	Should be closely involved
4. Feedback and data analysis	Should be closely involved
5. Report writing	Not necessary
6. Dissemination	Should be closely involved

Based on information from review, numbers of new TIs required can be estimated and accordingly Expressions of Interest can be advertised.

Based on information from Broad Mapping, organisations can be selected, and if possible within the time, contracted, to run TIs.

Selected organisation can be involved, and if already contracted can implement Site Assessment as the first step in intervention.

2.1.2 Organisations Involved in Mapping

A. Mapping TRG and NHRGCs

A Technical Resource Group or task force dedicated to mapping (Mapping TRG) at NACO level will provide technical oversight to the whole process of mapping under NACP III. This group of experts will be supported by a cadre of National HRG Consultants (NHRGCs), drawn from members of HRGs with previous experience of implementing mapping in different States.

The Mapping TRG and the NHRGCs have the following roles:

- Build capacity of NACO TI Project and Technical Officers, State AIDS Control and/or Prevention Societies and the Project Support Units (TSUs) in mapping approaches and mechanisms
- Identify and select State- or regional-level organisations that will be responsible for implementing mapping in each State in consultation with State AIDS Control and/or Prevention Societies
- Mapping TRG and NHRGCs orient and train selected State- or regional-level organisations in mapping approaches and methodology, including selection and recruitment of HRG members to implement mapping

- NHRGCs select HRG members to implement mapping in collaboration with selected State- or regional-level organisations
- Mapping TRG and NHRGCs train HRG members they recruit in implementing mapping
- NHRGCs provide hands-on technical and mentoring support to HRG mapping teams
- Provide technical assistance during the field implementation of mapping, feedback and analysis of information, report writing and dissemination
- Intervene in case of any technical dispute or other major crisis
- If necessary, the Mapping TRG will also carry out the review of available information in consultation with State AIDS Control and/or Prevention Societies

B. State or regional organisations

The broad mapping and site assessments are to be carried out at each State, and within the State at each site identified through the process of review of available information. These activities will be carried out by selected State or regional organisations (either an individual organisation or a group of two organisations, to fulfil the selection criteria given below).

Role of organisations

- To carry out review of available information in consultation with Mapping TRG and respective State AIDS Control and/or Prevention Societies
- To recruit, contract, train and remunerate HRG members to implement mapping in the State
- To provide administrative and technical oversight to implementation of broad mapping and site assessment (e.g. hiring field staff to support HRG mappers)
- To provide logistical and administrative support to HRG mapping teams during training, field implementation, feedback and analysis of data
- To take a lead in analysis of data in collaboration with HRG mapping teams
- To compile a State-level mapping report

Selection criteria

- Institutional capacity (in terms of human resources, breadth of experience, systems in place) to work across a particular State and to work simultaneously at multiple centres in the State
- Financial and administrative capacity to recruit, contract and remunerate HRG members for the duration of the mapping
- Commitment to and proven track record of working with HRGs or other marginalised and stigmatised populations
- Proven track record of delivering on time
- Capacity to implement, or amenability to learn to implement, participatory methods
- Experience of carrying out field-based research and handling data

- Capacity to compile reports to specifications
- Commitment to disseminating results of mapping
- Cost effectiveness

Selection and contracting procedure

1. The Mapping TRG will support the NACO TI team in selecting organisations to implement mapping in each State, using the selection criteria given above
2. A State or regional organisation or groups of organisations can be selected to implement mapping in a geographical region, provided they have the capacity to do so
3. One organisation will be independently contracted to implement mapping in a State or a region

C. Summary chart

Roles of Different Players in Mapping				
Step	Who leads it?			
	Implementation	Administrative and logistical support	Recruitment and mentoring	Technical oversight
1. Review of available information	Selected State- or regional-level or organisation in initial stages Mapping TRG	-	-	Mapping TRG with local SACS and TSU
2. Broad mapping	Trained HRG members from the site	Non-HRG field staff of selected State- or regional-level organisation	NHRGCs	Mapping TRG with local SACS and TSU
3. Feedback and data analysis/ report writing of broad mapping	Trained HRG members from the site Selected State- or regional-level organisation	Selected State- or regional-level organisation	NHRGCs	Mapping TRG with local SACS and TSU
4. Site assessment	Trained HRG members from the site	TI NGO/CBO, with support from State-level organisation	NHRGCs	Mapping TRG with local SACS and TSU

2.1.3 Methodologies of Mapping

A. Methodology for review of available information

Please note that this step will be skipped for mapping MSM, as a District-wise list of sites to be mapped has already been developed.

Objective

Programmatic	Mapping related
1. To determine the sites where mapping will be carried out in a particular State	2. To estimate the time that it will take to implement Broad Mapping in the State

Who will do it?

- Selected State- or regional-level organisation
- If those organisations are not yet selected, the Mapping TRG at the initial few States to kick-start the process

Sources of information

1. Secondary sources
   - Published surveys about HRGs in India, including National Survey, Sentinel Surveillance reports
   - Past mapping data for the State available with SACS
   - RCSHA reports, if relevant to the State
   - DFID-PMO documentation, if relevant to the State
   - Other site estimation reports from local and international NGOs including USAID, Avahan partners, if relevant to the State
2. Consultation with selected key informants
   - SACS, TSU (if in place), other government departments, local NGOs working with HRGs, HRG CBOs

Process

1. The selected State- or regional-level organisation or members of Mapping TRG and selected NACO TI Project and/or Technical Officers collect all existing secondary data available about numbers and location of HRGs in the State
2. Selected State- or regional-level organisation or members of Mapping TRG and selected NACO TI Project and/or Technical Officers visit the State to be mapped for 2 days

3. On Day 1 they will review the existing secondary data along with the SACS and PSU staff and compile a preliminary list of sites where there is significant concentration of HRGs (100 or more sex workers and 50 or more MSM or IDUs in a site)
   - This list will include all sites where HRGs are known or likely to be present in significant numbers, such as big towns, trading towns, religious centres, traditional sex work sites, existing TI sites, etc.
4. On Day 2 SACS will convene a meeting and ensure participation of:
   - SACS representatives (Project Director, NGO Advisor and other staff who have been working in the State for more the 1 year)
   - TSU staff
   - Representatives of other relevant government departments in the State who might have knowledge about numbers and distribution of HRGs in the State
   - At least one NGO each with experience of running interventions with sex workers, MSM and *hijras*, and IDUs
   - Representatives of local HRG CBOs or networks
5. At this meeting in-depth consultation will be held to finalise the list of sites to be mapped in the State from the preliminary list prepared from the review of existing data
6. The preliminary list will be reviewed at this meeting using the following checklist:
   - Which HRGs are reported to be present at the site?
   - Which subcategories of HRG are present at the site?
   - What is the reported number of members of each HRG subcategory reported to be present at the site?
   - What explains the estimated number of HRGs? (E.g. it is a trading centre and therefore there is a large turnover of likely clients of sex workers; there is a military camp in the town, therefore there is a concentration of likely sexual partners of kothis and hijras; existence of injecting sharing networks, mapping data suggesting the estimate is not more than 1 year old and is realistic and credible.)
7. At the suggestion of the participants, sites can be added to or deleted from the preliminary list. The following criteria are used to determine if a site will be included or excluded in the final list of sites to be mapped in the State:
   - The reported presence of one or more HRG is significant enough to warrant at least one TI at the site
   - The estimated number can be justified
   - If a TI has mapped the HRGs of the site **with substantive participation of HRGs themselves**, and the data they have is **reliable and credible**, the site will be excluded from the final list of sites to be mapped in the State

8. Once the final list has been compiled and verified at the meeting, the time needed to implement broad mapping of each site will be estimated site by site through in-depth consultation with the participants, keeping in mind the size of the site, transportation facilities within the site, law and order situation at the site.

Expected outputs

1. List of sites to be mapped in the State
2. Time estimate for broad mapping all the sites in the final list

B. Methodology for broad mapping

Objective

Programmatic	Mapping related
1. To determine the Districts and sites where TIs will be located on a priority basis	2. To develop a list of sites where site assessment will have to be carried out<br>3. To prepare a list of reported hotspots at each site where site assessment will be carried out<br>4. To estimate the time it would take to implement site assessment at each site

Who will do it?

Trained HRG members from the site, with administrative and logistics support of selected State- or regional-level organisation, mentored and supervised by NHRGCs and with technical oversight by the Mapping TRG and local SACS and PSU.

Sources of information

1. Primary Key Informants
2. Secondary Key Informants
3. Tertiary Key Informants

For detailed definitions of these informant groups, see Section 2.1.1.C above.

Process

1. The Mapping TRG assigns a team of NHRGCs to support the State or regional organisation responsible for implementing mapping in the State
2. The NHRGC team, in consultation with local SACS and TRU, selects local HRG field researchers to implement broad mapping

3. The State or regional organisation recruits and contracts the local HRG field researchers for stipulated days (for training, field implementation, feedback and data analysis)
4. The Mapping TRG and NHRGCs carry out a Training of Trainers for trainers from the State or regional organisation
5. The State or regional organisation trains the local HRG field researchers
6. The local HRG field researchers implement broad mapping at every site selected in the State. **(See Annexure 1, *Broad Mapping*.)**
7. During field implementation by local HRG field researchers, the NHRGC team assigned to the State mentors them and provides technical supportive supervision, and the State or regional mapping organisation staff provide administrative and logistics support
8. The local HRG field researchers and the NHRGC team reconvene for a feedback and data analysis workshop facilitated by the State or regional mapping organisation
9. The State or regional mapping organisation prepares a list of Districts where TIs are to be implemented on a basis of priority, based on the data analysis and in consultation with the local SACS and TSU.

Expected outputs

1. A list of Districts and sites where TIs will have to be located on a priority basis
2. List of reported hotspots at each site
3. Size estimate by subcategories
4. HIV/STI services available for HRGs

C. Methodology for site assessment

Objective

Programmatic	Mapping related
1. To determine the site-specific design of TIs<br>2. To initiate interventions<br>3. To contact at least 50% of the broad mapping denominator at least once<br>4. To build rapport of TI with the HRG community<br>5. To identify potential peers	6. Not applicable

Who will do it?

Trained HRG members from the site, selected for the purpose with administrative and logistics support of the TI NGO/CBO who will operate in that site, with technical support from the selected State- or regional-level organisation, mentored and supervised by NHRGCs and with technical oversight by the Mapping TRG and local SACS and PSU.

Sources of information

Members of HRGs and their sexual partners at each hotspot. For example, sex workers and their clients, people who inject drugs and their sexual partners, a kothi and his panthis.

Process

1. The NHRGCs involved in the broad mapping of the State are available to support TI NGOs/CBOs in conducting the site assessments. It is critical to ensure continuity between the broad mapping and site assessment steps.
2. TI NGO/CBO (with support from NHRGCs as needed) selects local HRG field researchers as community guides to implement site assessment. For details on this selection process, see Section 3.2.1.C below. The HRG community guides from the site who had implemented broad mapping in the State will be included, but depending on the size of the site in question, more HRG community guides may have to be selected.
3. The TI NGO/CBO recruits and contracts the local HRG field researchers for stipulated days (for training, field implementation, feedback and data analysis)
4. The TI NGO/CBO trains the local HRG field researchers to implement site assessment and the TI staff to support them
5. The local HRG field researchers implement site assessment at the selected site. **(See Annexure 2, *Site Assessment*.)**
6. The local TI NGO/CBO community guides and the NHRGC team reconvenes for feedback and data analysis workshop facilitated by the TI NGO/CBO

Expected outputs

1. Details on risks/vulnerabilities by typology and location for HRG members
2. Validation of broad mapping size and location estimates
3. Initial rapport with at least 50% of the broad mapped HRG denominator
4. Identification of potential peers (among the community guides and other HRGs mapped)

Tools

Annexure 1 ***Broad Mapping***

Annexure 2 ***Site Assessment***

2.2 ANALYSING THE COVERAGE AND QUALITY OF CURRENT TIs AMONG HRGs

2.2.1 Analysing Existing TIs

In the State the first priority should be to complete the mapping/assessment (described in Section 2.1 above) for size estimation of HRGs by category.

Based on mapping and size estimation data, a set of analyses can help define the scope and scale of needed TI coverage in the State:

- There should be a physical map of the entire State describing mapping data for each location and site
- The data should include all the detail information collected during mapping (e.g. locations of sex workers, typology, numbers/concentrations by region)
- The map should also include information of existing TIs and their coverage

This geographic picture of the State will highlight gaps in TI coverage. Based on these gaps, TIs can be configured or supplemented as per the criteria below.

2.2.2 Criteria for TI Allocation

TIs should be allocated, or where they already exist, an analysis of whether they are able to saturate coverage of the existing HRGs should be conducted, based on the following criteria:

- Locations where there are no interventions
  - Large pockets of HRGs
  - Smaller pockets of HRGs
- Locations where HIV prevalence rate is higher than other Districts
- Locations where sizeable number of HRG exist with some TIs but not covering 100% of the HRG
- Locations where TIs exist but coverage (outreach to HRGs on a monthly basis of >80%) is low
- To achieve economic efficiency (TI units of 800-1,500 HRG members, with the possibility of one NGO covering multiple TI units, or one TI unit covering multiple HRG groups)
- To achieve 80% coverage of HRGs

2.2.3 TI Unit Size

Evidence shows that for interventions among HRGs to be cost-effective and impact-efficient, each TI unit should aim to provide services to **800-1,500** HRG members (150-350 for IDUs, and in select cases, up to 1,000 where there are concentrations of IDUs)

If a particular NGO or CBO is already working with a larger population of HRGs, and has the necessary capacity to provide the comprehensive package of services to them, it can be assigned more than one TI unit, depending on the actual size of the population it works with.

Similarly, even in areas where new TIs are to be started, **an NGO/CBO can be assigned more than one TI unit, each covering 800-1,500 FSWs/MSM, provided there are such numbers in a particular geography and the NGO/CBO has the appropriate capacity.** For IDUs, the unit size may be smaller-between 150 and 1,000.

In some areas, the size of the sex work population may well be smaller than 800. In such instances the TI can address a cross-HRG composite group, that is, a combination of FSWs, MSM and transgenders and/or IDUs so that the total population size addressed by the TI unit is 800-1,500.

Thus there are only two possible types of HRG TIs under NACP III:

1. **TIs for a single core group** – e.g. FSW-only TIs, or MSM-only TIs, or IDU-only TIs
2. **Core composite TIs for multiple core groups**: e.g. TIs for FSWs and MSMs in a given geographic area

Composite TIs for HRGs can be composed only of core HRG members, not bridge populations like truckers or migrants.

2.2.4 Geographic Distribution of TIs

Based on HIV surveillance data, epidemiological profile, risk and vulnerability, NACO has classified the 609 Districts in the country into 4 categories: A, B, C and D.

Description	Category	Number of Districts
In District in any time in any of the sites in the last 3 years	A	**156**
In all the sites during last 3 years associated with more than 5% prevalence in any HRG group (STI/FSW/MSM/IDU)	B	**39**
Less than 1% in ANC prevalence in all sites during last 3 years with less than 5% in all STI clinic attendees or any HRG with known hot spots (migrants, truckers, large aggregation of factory workers, tourists, etc.)	C	**296**
Less than 1% in ANC prevalence in all sites during last 3 years with less than 5% in all STI clinic attendees or any HRG or no/poor HIV data with no known hotspots	D	**118**

Given the variations in risk among Districts, Categories A, B and C will receive high priority in scaling up. In category D Districts and parts of category C Districts, focus will be on awareness-raising strategies for vulnerability reduction, risk reduction, promotion of protective behaviours and data-gathering on the extent of the high risk groups for initiation of TIs.

Scale-up will likely occur in a few stages, based on concentration of HRGs. These stages are usually in the following order of geographical distribution:

- Initially in large cities/towns
- Then peri-urban areas
- Finally in rural areas (to be covered by Link Worker Scheme)

2.3 RECRUITMENT, CAPACITY BUILDING AND PROGRAMME MANAGEMENT

2.3.1 Recruiting NGOs/CBOs/Networks to Implement TIs

Through NACP I and II, the focus has been on implementing TIs through NGOs. NACP III aims to implement through NGOs and CBOs

To bring about a systematic and transparent process for identification, field appraisal, selection, funding and monitoring of suitable NGOs, CBOs and networks, NACO has developed *NBO/CBO Guidelines* and *Guidelines on Financial and Procurement Systems for NGOs/CBOs*. These guidelines:

- Delineate the process involved in calling for applications, partner identification, appraisal and contracting, capacity building of partners, monitoring and evaluation
- Explain the steps in each stage and outline the process
- Enable SACS/TSUs to establish procedures for the various stages by adapting them to specific contexts

Each SACS is to recruit suitable NGOs, CBOs or networks following the processes laid out in the guidelines to implement the numbers of TI units required to saturate coverage of the HRGs mapped and estimated in the State. Interventions implemented and led by HRGs themselves lead to faster and more effective and extensive coverage than NGO-led interventions. In order to achieve this comparative advantage, CBOs and HRGs require high-quality capacity building. For examples, refer to Chapter 5.

There are four possible types of interventions under NACP III:

1. Funding of existing or new NGOs
2. Funding of existing CBOs
3. Funding of *de novo* CBOs
4. Funding of NGOs with capacity building to help them transition to CBO-led model of intervention, with NGOs continuing to play a role in support and technical assistance

Note on CBO selection and transition guidelines

For details on transitioning to CBOs, refer to Chapter 5. The CBO guidelines outline the process of CBO formation and development, either as offshoots of NGOs, or *de novo* (from scratch). It is critical to note that CBO formation takes time, and the percentage of funding expected to go to CBOs may vary based on the stage of existing interventions in States. For example, States with longstanding interventions and existing CBOs may be able to develop CBOs which could be funded before States without long-standing interventions. **Chapters 2 and 3 focus on the NGO-led model of intervention.**

To implement and operationalise the TIs and ensure the quality of their services, the capacities of SACS/ TSU and DAPCU, as well as the NGOs, CBOs or networks that will run the TIs, must be strengthened.

Tools

NACO ***NGO/CBO Guidelines***

2.3.2 Capacity Building Plan for NGOs and CBOs Implementing TIs

Note: The budget for capacity building will be earmarked under the SACS budget. If there is a TSU in the State, SACS will release TI training budget to the TSU on an annual basis (based upon spending against a regular "impress" or "indent").

TI component	Capacity building on	Objective	For whom	Types of training	When	By whom
**Outreach/ Peer Education**	■ Rationale and design of TIs under NACPIII<br>■ Roles and responsibilities of Peer Educators and Outreach Workers<br>■ Roles of other TI staff in outreach<br>■ Sexual and Reproductive Health<br>■ Basics of STI<br>■ Basics of HIV<br>■ Gender<br>■ Sex and sexuality<br>■ Values and attitudes about HRGs and HIV<br>■ Structual contexts of HIRGs<br>■ Community mobilisation and its role in Tis	To build capacity to support and manage PEs and ORWs	Project Coordinator, Counsellors	2-day orientation training	By the 2nd month of intervention by the TI	TSU with support from State-level capacity building consultants or organisations, including trained trainers from HRGs
		To build capacity to implement	Peer Educators (PEs) and Outreach Workers (ORWs)	8-day training including at least 50% of the time spent on field practice; 2 days classroom training + 4 days mentored fieldwork practice in own sites (spread over 1 week) + 2 days classroom training	By the 4th month of intervention by the TI	

TI component	Capacity building on	Objective	For whom	Types of training	When	By whom
**Outreach planninng and management**	Site Assessment	To build capacity to plan outreach and support and manage PEs and ORWs	Project Coordinator, Counsellors	1-day orientation	By 5th month	TSU with support from State/regional mapping organisation, including trained trainers from HRGs
		To build capacity to implement outreach plan (some PEs and ORWs will already be trained in this during mapping)	PEs and ORWs	4-day training including 2-day mentored field practice		
**Communica-tions**	Dialogue-based interpersonal communication (IPC)	To build capacity to support and manage PEs and ORWs	Project Coordinator, Counsellors	1-day orientation	In the 4th month	TSU with support from State/regional mapping organisation, including trained trainers from HRGs
		To build capacity to implement dialogue-based IPC	PEs and ORWs	4-day training including 2-day field practice	In the 4th month	
**STI**	STI Management	To strengthen capacity of TI doctors in clinical STI management	TI doctors	4-day training	In the 4th month	TSU
**Condom programming**	Planning condom programming	To build the capacity of Tis to estimate requirements, stock, distribute and monitor distribution and usage of condoms	Project Coordinator, TI accountants, PEs and ORWs			TSU with support from external consultants

TI component	Capacity building on	Objective	For whom	Types of training	When	By whom
**Counselling**	■ Nature and purpose of counselling<br>■ Counselling skills<br>■ Sex and sexuality<br>■ Understanding HRG issues and rights<br>■ HIV/STI prevention counselling<br>■ Counselling sex workers, MSM and IDUs	To strengthen HIV counselling skills	TI counsellors	6-day residential training	In the 4th month	State or regional counselling training institute accredited by NACO
	■ Sex and sexuality<br>■ Understanding HRG issues and rights<br>■ HIV/STI prevention counselling<br>■ Counselling sex workers, MSM and IDUs	To build capacity to work with HRGs	TI doctors	2-day training	In the 4th month	
**Programme management**	■ Importance of programme management<br>■ Intervention planning<br>■ Quality assurance<br>■ Supportive supervision<br>■ The role of a manager	To strengthen management capacity	Project Coordinators	3-day training	In the 2nd month	TSU with support from external consultants
**Programme vision and design**	Understanding best practices in focused prevention among HRGs	To strengthen capacity to implement TI creatively and effectively	Project Coordinators, selected PEs and ORWs	Exposure visits to learning site	In the 2nd month	SACS to coordinate

TI component	Capacity building on	Objective	For whom	Types of training	When	By whom
**MIS**	■ Understanding the structure of the MIS system<br>■ Indicators<br>■ Field level formats<br>■ Peer Cards<br>■ Data Collection<br>■ Reporting and documentation	To build capacity in monitoring and documentation	■ Project Coordinators<br>■ TI accountants	4-day Training	In the 4th month	TSU with support from external consultants
			■ ORWs<br>■ PEs	2-day Training	In the 5th month	
**Finance management**	■ Account keeping<br>■ Accounting software<br>■ Finance Management<br>■ Statutory Issues	To strengthen finance skills and systems	TI accountants	3-day Training	In the 2nd month	TSU with support from external consultants

TI component	Capacity building on	Objective	For whom	Types of training	When	By whom
**Community Mobilisation**	■ Group dynamics and group cohesion<br>■ Ownership of TIs<br>■ Rights of HRGs<br>■ Understanding power dynamics in lives of HRGs<br>■ Specific risk and vulnerability factors of HRGs<br>■ Attitude and values towards sex, drugs and autonomy<br>■ Legal frameworks affecting HRGs and how to address them	To strengthen capacity to mobilise HRGs for HIV prevention and protection and promotion of their rights	PEs and ORWs	4-day residential training	In the 7th month	Trained trainers from HRGs
	■ How it works in reality			Exposure visits to strong CBOs	In the 10th month	SACS to coordinate
Enabling Environment	Advocacy	To strengthen capacity for planning and implementing advocacy	■ Project Coordinators<br>■ PEs<br>■ ORWs	3-Day training	In the 10th month	Trained trainers from HRGs with support from TSU
	Crisis management	To strengthen capacity to mitigate crisis	PEs and ORWs +orienation for Project Coordinator	■ PEs<br>■ ORWs	In the 7th month	Trained trainers from HRGs with support from TSU

2.3.3 Programme Monitoring

The project life-cycle of the TI follows a few phases of scale-up, which should be reflected in the monitoring and management of these TIs:

1. **Scaling coverage**
   - Mapping of HRGs and defining where interventions need to be launched
   - Commissioning TIs to ensure saturated coverage of HRGs at the State level
2. **Scaling infrastructure** (0-3 months)
   - Improving infrastructure with respect to clinics and DIC (Safe Places)
3. **Scaling intensity of service delivery** (3-12 months)
   - Ensuring regular outreach contacts with >80% of the population on a monthly basis
   - Ensuring regular STI uptake for the population on a monthly basis
   - Ensuring condom availability and accessibility
   - Creation of an enabling environment – crisis response, power structure mapping and analysis
   - Strengthening community initiatives – formation of community committees, seeding collectives, etc.
4. **Scaling quality of service delivery** (9-18 months)
   - Improving service delivery
   - Strengthening monitoring and evaluation of TI
   - Improving linkages with DAPCU and other local administration
   - Strengthening fund utilisation
   - Strengthening referrals to TB units and other OI/VCTC/ART referrals
   - Building CBO systems

The process of monitoring happens at three different levels:

- National level by NACO
- State level by SACS & TSU
- TI level by NGO implementing the project

Programme monitoring of State performance should assess the performance of the TIs based on the life cycle mentioned above.

- SACS/TSU should be assessed on all four phases
- NGOs/CBOs/TIs should be assessed on phases 2-4

2.3.4 Programme Management

A. Objectives of programme management

- To improve quality and management of TI
- To effectively deliver project services to the HRG
- To increase the coverage of, and uptake of services by, the HRG
- To provide training and hand-holding wherever required
- To identify and effectively fill gaps in TI implementation
- To set up efficient administrative and management systems to support these operations

B. Role of State AIDS Control Society (SACS)

The overall responsibility of implementing NACP III in the State belongs to the SACS. SACS plans, monitors and manages TIs through partner organisations. SACS ensures adequate resources to accomplish goals and it will ensure the minimum quality of interventions. SACS provides support and necessary mentoring to achieve its objectives. It reviews and monitors all partner organisations to identify gaps in TIs and address them.

C. Role of Technical Support Unit (TSU)

The TSU oversees the implementation of TIs in the respective State along with SACS. The TSU follows NACP III guidelines developed by NACO and facilitates its implementation along with partner organisations. The TSU facilitates the designing, planning, implementation and monitoring of targeted interventions in the States along with SACS, and provides management and technical support to the SACS.

Being in the field

The key to successful programme management of TIs is field-level presence: TSU project officers should spend at least three weeks in a month visiting TIs to provide hands-on capacity building and problem solving support in three key programme areas: STI, M&E and outreach/ community mobilisation.

The TSU makes supportive visits to partner organisations and ensures that coaching and mentoring to NGOs and TI staff are available. It participates in periodic reviews of all partner organisations and provides necessary inputs. TSU staff includes project officers who visit TIs on a regular basis to assess quality of STI services, outreach and M&E.

D. Role of Non-Governmental Organisations (NGOs)

NGOs implement TIs in their respective project areas and achieve objectives laid out by the project plan. The implementation of TIs follows the guidelines of NACP III. All NGOs report to SACS/TSU

and can seek support wherever required. Each NGO prepares a project implementation plan along with its respective SACS/TSU. NGOs will liaise with DAPCU, local health authorities and other NGOs while implementing TI. They will work towards forming a CBO of HRGs and strengthen it so as to transfer their project to the CBO at the end of year five.

E. Principles of CMIS for TIs

As a result of the scale of TIs and the importance of information gathering, analysis and use by the project, NACO has developed a Computerised Management Information System (CMIS). The meaning of CMIS and its uses should be understood clearly by the community, partner NGOs/CBOs and SACS/TSU. CMIS:

- **is not** a means to find faults in the implementation process
- **is not** gathering of information to be used only for research purposes
- **is not** gathering of quantitative information only
- **is** diagnostic, i.e. to identify opportunity gaps in the project implementation
- **is** supportive, i.e. to help bridge opportunity gaps for optimum implementation of the project
- **is** participatory, i.e. the community, NGOs/CBOs and SACS/TSU are equal partners in monitoring

F. Timelines and key indicators

Programme management occurs at the levels of the SACS, TSU, JAT, and TI/NGO. Teams from each of these groups play a role in monitoring project progress against indicators.

The attached Annexure 10, *Programme Management,* lays out the inputs, outputs, timelines, and monitoring guidelines for each of the programme areas.

An example of the programme management framework – Master Plan for TIs – is outlined below. This is for the programme component of BCC. Each other programme area (e.g., mapping, STIs, condoms, community mobilisation, peer engagement) has its own table like the one below.

Programme Component	Behaviour Change Communication / Interpersonal Communication			
Pre-requisites	1. TI coverage area and denominator fixed<br>2. TSU contracted and fully staffed<br>3. NGO contracted and funded as per NACO guidelines<br>4. NGO outreach staff (esp. project coordinator, outreach workers, advocacy officer) recruited to cover intervention area as per staffing guidelines<br>5. Site validation process completed<br>6. Peer educators from HRG recruited to cover all sites as per peer selection guidelines			
Input	1. Annexure 6a, *Dialogue Based Interpersonal Communication (IPC) By and With HRGs*			
Output	1. IPC packages for risk reduction			
Activities		Primary responsibility		
		SACS	TSU	NGO
Adapt IPC and BCC toolkits for local use				
Train NGO staff and peer educators on IPC methods - especially the value of analytical thinking and problem solving among community members to arrive at local solutions to HIV/AIDS risk and vulnerability issues				
Train NGO staff and peer educators on strategic planning for BCC message development				
Review NGO-developed BCC materials and NACO/SACS materials for message consistency/message reinforcement				
Conduct IPC capacity standards jointly with NGO staff and peer educators every six months to assess quality of IPC and identify areas for improvement				

Tools

Annexure 6a ***Dialogue-Based Interpersonal Communication By and With HRGs***

Annexure 10 ***Programme Management***

2.3.5 Financial Management

Available funds should be used in accordance with plans and proposals given to SACS/NACO/TSU. Proper accounting systems should be in place and all the necessary records should be maintained for internal/external auditing. For details, see the NACO *NGO/CBO Guidelines.*

Tool

NACO ***NGO/CBO Guidelines***

NACO ***Guidelines on Financial and Procurement Systems for NGOs/CBOs***

NACO ***STI Guidelines***

CHAPTER 3

Operationalising Targeted Interventions for FSWs/MSM/TGs: Guidelines for NGOs

TABLE OF CONTENTS

3.1 INTRODUCTION: NEW INTERVENTIONS

3.2 PHASE 1 OF INTERVENTION: START-UP

3.2.1 Step 1: Recruitment and Training of Staff (other than Peer Educators)

A. Hiring outreach workers and other TI staff

B. Capacity building of TI staff

C. Hiring and capacity building of community guides

3.2.2 Step 2: Site Assessment

3.2.3 Step 3: Establishment of Basic Services

A. Tips for planning services

B. Safe spaces: Drop-in centres (DICs)

3.3 PHASE 2 OF INTERVENTION: FROM PEER EDUCATOR RECRUITMENT TO SCALE-UP

3.3.1 Step 4: Peer Educator Selection and Training

A. What is a peer educator (PE)?

B. Why peer education?

C. Role of the PE

D. PE selection criteria

E. Process of PE selection/recruitment

Informal approach to selecting potential PEs

Formal selection process

F. Capacity building plan for PEs

G. Review and Rotation of PEs

H. PE progression pathways

3.3.2 Step 5: Scaling Up Services

A. STI and other clinical services

Planning and mode of service delivery

STI management strategy and implementation approaches for FSWs/MSM/TGs

B. Condom programming

The basics of free condom programming for FSWs/MSM/TGs

The basics of social marketing (SM) of condoms

Condom stocking/reporting

Special studies to assess condom use

C. Communication for behaviour change

3.3.3 Step 6: Outreach Planning
3.3.4 Step 7: Community Mobilisation
A. Community Committees
B. Collectivisation and CBO development
3.3.5 Step 8: Creating an Enabling Environment
A. What vulnerabilities do FSWs/MSM/TGs face?
B. Stakeholder/Power analysis
C. Crisis response system
D. Police advocacy
3.3.6 Step 9: Linkages with Other HIV Prevention/Care Programmes through DAPCUs
A. Role of DAPCU in TIs
B. Role of the DAPCU in enabling sevices linkages
3.4 PROGRAMME MANAGEMENT
3.4.1 Hiring and Training Staff
A. TI staff positions
B. Assessing attitudes and expectations
C. Establishing roles and responsibilities
3.4.2 CMIS Indicators
3.5 COSTING GUIDELINES
3.6 EXISTING INTERVENTIONS

3.1 INTRODUCTION: NEW INTERVENTIONS

These guidelines are designed for NGOs/CBOs starting a new targeted intervention (TI) to female sex workers (FSWs), men who have sex with men (MSM) and transgenders (TGs) or scaling up an existing intervention. Where an intervention already exists, the process can be modified as discussed briefly in Section 3.6. For a detailed list of existing MSM TIs, see Annexure 12.

The guidelines assume that a desk review and/or broad mapping of existing TIs on the ground has been completed by an external agency, and that the number and location of sites, and an estimate of FSWs/MSM/TGs by typology, are available.

**Tool**	
**Annexure 12**	***Excerpt from Infosem's 'Strategic Plan for Scaling Up Interventions for MSM and Transgender Populations in India'***

Steps in Starting and Scaling Up Targeted Interventions (TIs)

1. Recruitment and training of staff
2. Site assessment
3. Establishment of basic services
4. Peer Educator (PE) selection and training
5. Scaling up services
6. Outreach planning
7. Community mobilisation
8. Creating an enabling environment
9. Linkages with other services

3.2 PHASE 1 OF INTERVENTION: START-UP

Phase 1 of the Targeted Intervention comprises three major steps: staff recruitment (except for Peer Educators) and training, site assessment, and establishment of basic services.

3.2.1 Step 1: Recruitment and Training of Staff (other than Peer Educators)

A. Hiring outreach workers and other TI staff

NACO staffing guidelines for TIs stipulate that one outreach worker should be hired per 250 HRG members. The NGO should plan its own selection methods, e.g. group discussion, written examination and interview. Peer educators from other existing projects or CBO members can be part of the interview process. Outreach workers should have the following profiles:

- Non-judgmental attitude and willingness to work with FSWs/MSM/TGs. A good understanding of the community mobilisation process is also a plus.
- Previous experience of working with the same or any other HRG is desirable but not essential
- FSWs/MSM/TGs or their children should be given equal opportunity and priority if they meet the defined guidelines (e.g. educational and/or other qualifications, reporting skills)
- Strong facilitation skills
- Knowledge of local languages

B. Capacity building of TI staff

Trainings should be conducted for the new staff on the following:

- Basic induction on HIV/AIDS and understanding the FSW/MSM/TG community and the dynamics of sex work
- Skills in identifying and building rapport with FSWs/MSM/TGs and methodology of site validation

C. Hiring and capacity building of community guides

Any intervention for high risk behaviours requires the active involvement of members of the community from the beginning. Guides selected from the community can help the field team gain access to the vulnerable group, identify locations, help to estimate the size of the group, collect data for the initial survey and assist the investigator throughout the assessment. This also establishes as a norm the involvement of the community in making decisions for all activities concerning them.

Guides' compensation can be used from the "Peer educator" line item within the TI budget.

Potential guides can be identified quickly among the following groups: risk assessment contacts who were from the community; referrals from peer educators from other existing FSW/MSM/TG TI projects; and recommendations from project staff with site experience. Select guides who meet the following criteria:

- Available for the programme in terms of time
- Keen to work in the programme
- Representative of and accepted by the community
- Representative of multiple "social networks" from different locations/sites
- Knowledgeable about the local context and setting

Based on the staff induction training package, conduct a simple training for guides on HIV, the intervention and the process of site validation.

If a broad mapping was conducted with the assistance of NHRGCs, these NHRGCs can be leveraged by the NGO/CBO to identify potential local HRGs or community guides.

Why not hire Peer Educators immediately?
When a project starts, the TI staff may not know all the existing social and sexual networks. Selecting peer educators before understanding these networks can result in a skewed representation of the community. For example, an FSW programme hired 10 peers only to find that their total contacts did not exceed 100 FSWs. Upon investigation, they learned that all 10 peers were from the same social network and had overlapping contacts. This limited the extent of the intervention. Thus it is important to select peers from different social and geographic networks (at the 1:60 NACO recommended ratio).

3.2.2 Step 2: Site Assessment

The methodology of site assessment is referred to in Section 2.1.3.C above, and described in detail in **Annexure 2, *Site Assessment.*** The assessment is conducted by trained members of the local HRG group, who conduct a series of interactive exercises with members of their community, using visual tools (drawings and maps) to solicit information.

The objectives of the site assessment are to determine the site-specific design of TIs through:

- Validation of broad mapping size and location estimates
- Contact with at least 50% of the broad mapping denominator at least once
- Gaining details on risks/vulnerabilities by typology and location for HRG members
- Initiating interventions

Apart from the quantitative information gained in the assessment, there are qualitative outcomes:

- Establish contact with community – the site validation helps the project to meet at least 50% of the estimated population in a given location on a one-to-one or group basis
- Generate interest and curiosity about the project

- Dispel myths about the intervention before it even begins, and communicate correctly the project's scope and plans, avoiding false promises
- Identify potential peer educators for future hiring

Tool
Annexure 2 *Site Assessment*

3.2.3 Step 3: Establishment of Basic Services

In order for the community to have faith in the project and see early signs of benefit from it, basic prevention services (as per TI guidelines for FSWs/MSM/TGs) should be in place as early as possible. The basic services that can be established quickly are:

- Referral systems for treatment of STIs
- Availability of free condoms (and lubricants) through the project/staff/guides
- Setting up of a drop-in centre (DIC, also known as a safe space)

A. Tips for planning services

It is important to get the FSW/MSM/TG community involved in the planning of all basic services. The FSW/MSM/TG community will be able to indicate what types of services they need beyond the project-driven ones. Use the following approach:

- Talk to the community in a group setting and make a list of all required/requested services
- Differentiate between services the project can offer and those for which linkages/referrals need to be established
- Explore with the community how project-driven services (condom promotion and STI services) can be incorporated

B. Safe spaces: Drop-in centres (DICs)

"Safe spaces" are critical in the early phase of service delivery, especially for street-based populations.

- Public sites such as streets, parks, etc. do not allow much contact time for outreach workers or peers, so the creation of DICs as safe spaces is important
- At DICs, FSW/MSM/TGs can interact with each other, rest, seek advice, share information, approach someone in case of a crisis, or pick up condoms
- Other popular DIC activities are teaching self-defence, literacy classes and rotational savings schemes trainings
- Counselling and/or STI services can be provided at the DIC through counsellor and/or doctor visits on certain days/times. Referral to satellite services such as de-addiction, crisis response, social welfare schemes and services can also be provided through the DIC.
- The DIC should ideally be located close to the sex work sites or hotspots. The choice of the centre location will be dictated by availability and the preference of the community as to whether the centre should stand out or be relatively anonymous.

3.3 Phase 2 of Intervention: From Peer Educator Recruitment to Scale-Up

3.3.1 Step 4: Peer Educator Selection and Training

A. What is a peer educator (PE)?

A peer educator (PE) is a person from the HRG who works with her/his colleagues to influence attitude and behaviour change. PEs are responsible for providing information on HIV/STIs and harm reduction, and promoting condom use among colleagues/peers, which ultimately results in building peer pressure for behaviour change. They can also distribute condoms, lubricants, needles and syringes. They also provide basic data for monitoring the project. A PE is paid an honorarium as per NGO/CBO costing guidelines for her/his contribution to the TI project.

The PE to FSW/MSM/TG ratio is set at 1:60 – one PE for 60 FSWs/MSM/TGs.

B. Why peer education?

Peer education enables members of a given group to effect change among other members of the same group. It is considered to be one of the most effective and sustainable tools for changing group behaviour. Peer educators play an important role in TI implementation as they can:

- Help to build trust and establish credibility with the vulnerable group
- Provide a vital two-way link between the project staff and the community
- Provide important information about the vulnerable group to other stakeholders and the wider community
- Reach a large number of people effectively
- Provide a link between the service and the community (for instance, by introducing people or accompanying them to the service facility)

C. Role of the PE

- Conducting outreach: this includes identifying new FSW/MSM/TGs as well as maintaining regular contact with her/his own network of 60 FSWs/MSM/TGs. This might entail contacts on a weekly or bi-weekly basis within any given month.
- Able to meet all her/his contacts minimum once in 15 days
- Providing dialogue-based IPC to FSWs/MSM/TGs
- Encouraging service and commodity uptake - motivate FSW/MSM/TGs to come to DIC, distribute condoms, make referrals for sick FSWs/MSM/TGs
- Advocacy with the known power structure
- Training of new PEs from within the project and outside
- Maintaining the DIC
- Generating demand for Welfare Programmes and facilitating identification of beneficiaries

- Regular visit to condom service centres to gather information and to improve service
- Building skills of priority groups in understanding and assessing high risk behaviour, and in condom use, condom negotiation, identification of STIs, etc.
- Attending review meetings
- Preparing and presenting the daily reports to ORWs
- Report preparation for activities implemented
- Attending all trainings, workshops and seminars

Key programme focus areas

A good peer educator puts a great deal of effort into maintaining her/his social network. When new entrants into sex work enter her geographic/peer network, a FSW PE should be able to identify them and introduce them to services as soon as possible.

A PE should also be able to identify and segment her/his portfolio to identify and serve those FSWs/MSM/TGs with the highest risk profile (high volume, low condom use, new and young FSWs/MSM/TGs, those with a high volume of anal sex transactions).

D. PE selection criteria

- Available for the programme in terms of time
- Committed to the goals and objectives of the programme
- Representative of, and accepted by, the FSW/MSM/TG community
    - Representative of multiple "social networks" from different locations/sites
    - Representative in terms of age of their social network
- Knowledgeable about the local context and setting
- Sensitive to the values of the community, and able to maintain confidentiality
- Values accountability to her/his FSW/MSM/TG community and not just to the programme
- Tolerant and respectful of others' ideas and behaviours
- Good listening, communication, and inter-personal skills
- Demonstrates self-confidence and shows potential for leadership
- Potential to be a strong role model for the behaviour she/he seeks to promote with others
- Willing to learn and experiment in the field
- Committed to being accessible to her/his peers in times of crisis

E. Process of PE selection/recruitment

Informal approach to selecting potential PEs

a. Treat community guides as potential PEs during qualitative and quantitative surveys or when getting to know the community (see Section 3.2.1.C above)
b. Give priority to existing guides and key informants if they are suitable to join training for peer education. Ask them if they are willing to work as PEs.
c. Explain why you want to work with them
d. Tell them how much time they will need to spend working as peer educators

e. Explain the method of selecting PEs (i.e. training, assessment, etc.)
f. Work out a possible strategy for a peer outreach cycle in collaboration with selected peers

Formal selection process

The formal selection process should be clear and transparent to all FSWs/MSM/TGs in the area. The peer selection process should be well publicised within FSW/MSM/TG networks so that all those potentially interested in being peers can be considered for selection.

a. Conduct basic interviews to rank the candidates based on the criteria listed in Section 3.3.1.D above
b. Conduct a Contact Mapping exercise, facilitated by ORWs, to determine the size of the potential candidate's social network and whether she/he is well networked within her/his community (**for details, see Section II of Annexure 5, *Peer-led Outreach and Planning***).
c. Consolidate the lists from all peers to assess the overall contacts. Discuss with them to understand the duplication of contacts. If there is duplication, discuss who knows the duplicated FSWs/MSM/TGs better.
d. Ask each potential peer to bring her/his contacts to the project office. Organise a meeting with them to assess her/his contacts/rapport with the group.
e. Discuss with the group and find out whether they will accept/nominate her/him as a PE
f. Discuss and establish systems for monitoring the PE's performance by the community as well. Community members should able to contact the project if they have any issues related to the PE
g. Select the PEs based on the above consultations

F. Capacity building plan for PEs

As with other staff, PEs require support and training from the programme/NGO in several key areas:

- Sex and sexuality
- Sexual and reproductive health
- STI and peer role in STI management
- Basics of HIV/AIDS
- Condom promotion
- Negotiation skills
- Self esteem
- Care for PLWHA
- Peer-led monitoring
- Advocacy
- Community mobilisation

For details, see Annexure 3, *Peer Educator Training*.

G. Review and Rotation of PEs

Every six months, the performance of PEs should be reviewed against indicators spelled out in Section 3.4.2 below. Since all key components of the TI are led by PEs, this review is critical to keep track of quality of the intervention.

PEs should be selected for a period of 12 to 18 months. The peer selection process described above should be repeated after 12 to 18 months to ensure that the PEs in the network are "active" peers, and not PEs whose social networks have eroded/changed. This method also provides opportunities for more FSWs/MSM/TGs to participate and for developing second-line leadership.

H. PE progression pathways

Providing clear progression pathways for PEs is critical. The table below indicates the types of growth and positions PEs can attain. It should be noted that the progression pathways and positions shown are indicative only and not watertight compartments, and they may vary according to realities on the ground.

Growth Progression	1st Stage (Initial)	2nd Stage (Growth)	3rd Stage (Growth)	4th Stage (Mature)
Horizontal growth within project	■ Community member<br>■ Peer volunteer	■ Active member<br>■ Community guide<br>■ Peer Educator	■ Peer Educator<br>■ Core Committee member<br>■ Peer guide	■ Coordinator of committees<br>■ Advisory group member<br>■ Peer mentor
Vertical growth within project	■ Community Volunteer<br>■ Peer volunteer	■ Peer Educator<br>■ Peer co-worker	■ Sub-Committee member<br>■ Team member	■ Coordinator of Core committee<br>■ Team member and leader
Across boundaries	■ At project/ programme level<br>■ Participant as community member	■ Between projects<br>■ Peer Educator<br>■ Core Committee member<br>■ Community consultant	■ At programme level<br>■ Sub-Committee Member<br>■ Advisor-community development	■ At programme level<br>■ Coordinator<br>■ Program Mentor<br>■ Advisory group member

For details on vertical growth within the project, see Annexure 4, *Peer Progression*.

Tool

Annexure 3 ***Peer Educator Training***

Annexure 4 ***Peer Progression***

Annexure 5 ***Peer Led Outreach and Planning***

3.3.2 Step 5: Scaling Up Services

A. STI and other clinical services

Planning and mode of service delivery

Planning for STI services should be done with the FSW/MSM/TG community. It is important to gather the following information:

- Preferred list of physicians
- List of current barriers to accessing STI services
- Ways in which STI services can be made accessible and acceptable to FSWs/MSM/TGs in terms of location, operating hours, etc.
- Best mode of delivering STI services, e.g.:
  - **Intervention site based clinic:** This ensures confidentiality, less marginalisation and better quality of care. Easy to follow up but difficult to sustain.
  - **Referral to the public sector:** Services can be free but lack confidentiality, quality of services cannot be predicted and marginalisation of FSWs/MSM/TGs is unavoidable
  - **Referral to the private sector:** This ensures confidentiality, and services can be sustained, but quality and costs are difficult to predict

Once this information is gathered, health care services can be established through the preferred mode of service delivery. Special attention should be paid to ensuring **community-friendly STI service delivery** options:

- Clinicians with the right attitude towards the community
- Availability of services as per the needs of the community, e.g. late-night access
- Accessibility of services at optimal location (i.e. not too far from the major sex work sites, not requiring an auto ride)
- Basic infrastructure facility (facilities should be maintained at the standards stipulated by the NACO STI guidelines)
- Confidentiality between the clinic team and the community needs to be maintained

Effective prevention and treatment of STIs among FSWs/MSM/TGs requires attention to both symptomatic and asymptomatic infections. The prevention and treatment of STIs in FSWs/MSM/TGs at NGO clinics should have the following two components:

- **Management of symptomatic infections –** using NACO syndromic management flowcharts and laboratory diagnoses where available
- **Screening and management of asymptomatic infections –** quarterly history taking, physical examination and simple laboratory diagnostics (where available):
  - Treatment for asymptomatic gonococcal and chlamydial infections at the first visit and repeated every six months
  - Semi-annual serologic screening for syphilis

The packages of STI/STI services to be provided are (see *NACO STI Guidelines*):

- Health promotion and STI prevention activities, such as promoting correct and consistent use of male condoms (and female condoms where available) and water-based lubricants and other safe sexual practices
- Provision of free male condoms (and female condoms if available) and lubricants
- Immediate diagnosis and clinical management of STIs
- Provision of STI medicines and directly observed therapy for single dose regimes
- Health education and counselling for treatment compliance, correct and consistent use of condoms and regular partner treatment
- Periodic check-ups, syphilis screening and treatment of asymptomatic infections
- Partner management programmes (i.e. contact referral)
- Follow-up services
- Counselling support for seropositive persons
- Prophylaxis and treatment of simple Opportunistic Infections (OIs)
- Referral links to VCTC, HIV care and support and other relevant services
- Strong linkages with outreach activities targeted at FSWs/MSM/TGs and their regular partners
- STI surveillance as requested

As per the NACO STI procurement guidelines, all STI drugs are to be procured by SACS/NACO from GMP providers.

STI management strategy and implementation approaches for FSWs/MSM/TGs

Management Strategy

Technical Strategy	Role of NACO	Role of SACS/TSU/SSC
- Accessible and acceptable TI static and outreach clinics<br>- Adequate clinical services to provide effective STI services for FSWs/MSM/TGs (syndromic management of symptomatic STIs, regular screening and treatment of asymptomatic STIs for FSWs/MSM/TGs)<br>- Syndromic management of male clients<br>- Counselling on HIV risk reduction and informed choice on HIV testing<br>- Utilisation of strengthened strategic government facilities for STI services, HIV testing and treatment, TB treatment – upgrading government facilities	- Develop Clinical Operational Guidelines and Standards on:<br>- STI management<br>- STI and HIV counselling<br>- Syphilis screening and laboratory quality assurance<br>- Establishing referral network<br>- Capacity building of SACS/TSU:<br>- Training<br>- Regular technical support<br>- Develop tools for clinic service monitoring (STI services, counselling) including process, outcomes and quality of services<br>- Monitor process and outcomes – clinical services<br>- Evaluate effectiveness of STI services	- Facilitate implementation of the clinical operational guidelines and standards<br>- Capacity building of NGOs<br>- Training<br>- Adequate technical staff to provide regular technical support (defined as quarterly field visits to each TI to assess quality of STI services - see below for details)<br>- Develop referral network for HIV testing, treatment and care<br>- Monitor STI referral services

Implementation Approach

Technical Area	Implementation Details	Lead	Timeline and Frequency
Effective and quality provision of STI services that are acceptable and accessible: syndromic treatment of FSWs/MSM/TGs, regular screening (speculum and proctoscopic exam wherever necessary) and treatment of asymptomatic STIs including syphilis screening, STI services coordinated with outreach, ensuring condom promotion and community involvement	• TI-owned clinic established where cost-effective (>1,000 FSWs/MSM/TGs/site or high risk)<br>• TI-owned outreach clinics (fixed day, fixed site) established to reach smaller number and most at-risk FSWs/MSM/TGs<br>• For smaller groups of FSWs/MSM/TGs (<200), establish linkages with strengthened STI government facilities or trained preferred service providers (private practitioners)<br>• Adequate and quality STI services, STI/HIV counselling for FSWs/MSM/TGs<br>• Adoption of the NACO operational guidelines for STI management<br>• Ensure involvement of community members in clinic operations, including hiring and training of FSWs/MSM/TGs in clinic operations and management and quality monitoring<br>• Community members take ownership of the clinic – NGO supports community members to establish and design clinical services and to plan, manage and monitor them<br>• Adequate number of qualified, trained and supervised staff (MBBS physician) and counsellors to provide monthly STI screening and clinical services<br>• Adequate resources and commodities to provide free STI drugs, condoms and to implement operational guidelines and establish referral network<br>• Regular coordination of clinic staff and outreach/peer education	TSU/SACS/ NGO	• Clinic established within 6 months of NACP III<br>• Outreach clinic established within 12 months<br>• Linkages with Government facilities established by 18 months<br>• SCM by 1st year<br>• Asymptomatic treatment by 2nd year<br>• Universal regular STI check-ups by 3rd year<br>• Community ownership of clinic: ongoing
**Referral Network** for HIV prevention and care continuum	• Identification of referral organisations with the community, documentation and follow-up of referrals, organise meetings on referral mechanisms<br>• Establishment of formal referral mechanism for quality HIV testing and counselling. HIV testing and counselling referral facility should be sensitive to FSWs/MSM/TG special issues and have a strong referral mechanism to HIV treatment, care and support and other related services. If referral mechanism is not present, clinic to establish its own.<br>• Establishment of formal referral mechanisms for management of complex OIs, TB and ART, including follow-up management<br>• Establishment of linkages to community care and support and self-help groups<br>• Clinic maintains a referral directory, documents referrals and ensures follow-up	TSU/SACS<br><br>NGO<br><br>NGO	• Clinic with established referral linkages by 9 months<br>• Full referral network functional by 3rd year

Technical Area	Implementation Details	Lead	Timeline and Frequency
**Broader referral systems** for additional services as necessary (TB management, STI complications, medical care, social support, legal support, IDU services)	n Establishment of other referral linkages based on community-identified needs and available services in the community<br>n Clinic to maintain referral directory of other services, document referral and ensure follow-up of referral services	NGO	n Referral directory developed within 6 months of establishing clinic<br>n Referral mechanism established
**Clinic based laboratories or links to laboratories for syphilis screening**	n Establish clinic serologic testing for syphilis every 6 months and treatment of reactive cases for (>2,000 SW) or establish linkages with a laboratory with appropriate laboratory quality assurance systems.<br>n Establish laboratory quality assurance systems for clinic based laboratories and ensure quality of referral laboratories	NGO<br><br>TSU/SACS/SST	n Universal serologic screening for syphilis every 6 months by 3rd year
**Systems and staff in place to implement STI services and quality monitoring** based on STI/RTI technical guidelines and STI operational guidelines by NACO	n TSU/SACS/SST with adequate number of trained technical staff to provide capacity building support and conduct regular supportive supervision and monitoring of the TI clinics. (1 STI technical support person for 20-30 clinics, depending on the geographic spread.) Monitoring key areas on clinic operations, staff clinical knowledge, skills and performance, coordination of outreach programme, community involvement, client satisfaction and response, clinical management of STIs, infection control and waste management, drug and supply management, education and counselling, ethical standards, confidentiality, referral systems, monitoring, evaluation and reporting	TSUs/SACS/SST	n Staff in place by the time clinics are established<br>n Technical supervisors to conduct quarterly visits to all STI clinics
	n Paper-based clinic activity recording and reporting established, functional and utilised to improve clinical services<br>n Paper-based clinic reporting regularly entered into the computerised management information systems (CMIS) to generate information on clinic activities and STI outcomes	NGO and TSU/SST/SACS	n Paper-based system developed when clinic established<br>n CMIS operational by 2nd year
	n Systems in place to monitor and track overtime quality of clinical services in all clinics	TSU/SST	n Annual clinic audit to track level of quality service provision
	n Random visits to STI clinics to monitor clinic functions/ performance	NACO/SACS	n 5% of clinics half-yearly

B. Condom programming

Ensuring availability, accessibility and correct and consistent usage of condoms by HRGs is a core imperative of NACP III.

The two broad strategies for condom promotion for FSWs/MSMs/TGs	
**Primary strategy** Free supply of condoms to FSWs through TIs by NGOs/CBOs	**Secondary (optional) strategy** Social marketing of condoms (mainly for clients of core groups, but sometimes also to core groups) by NGOs/CBOs in collaboration with Social Marketing Organisations (SMOs)

Condoms should always be available for free. If and when demand for socially marketed condoms arises in these groups, appropriate mechanisms must be in place to ensure that the free and socially marketed supplies do not overlap.

Free condoms for FSWs/MSM/TGs (and also for MSM) will be designed to meet their specific needs. Prior experience shows that both FSWs/MSM have expressed need for condoms with extra lubrication and length, and MSMs in particular express interest in free condoms of extra thickness.

The basics of free condom programming for FSWs/MSM/TGs

- Ensuring availability alone is not enough – distribution does not ensure usage
- Ensuring accessibility is not enough – access does not ensure usage
- **The goal is increased correct and consistent <u>usage</u> of condoms by FSWs/MSM/TGs**

Address barriers to condom usage – It is important to understand various aspects related to condom usage among the FSW/MSM/TG population at the site level before initiating condom programming. Considerations may include:

- The barriers to condom usage, e.g. alcohol intake, "difficult clients"
- Misconceptions and myths regarding condom usage, e.g. not required for anal sex
- Condom availability in the area
- Condom accessibility – are condoms available at the point of sex (or does FSW/MSM/TG have to travel to procure the condom) and at the time of sex (often in the evening/at night)?
- Creating demand for condoms (see guidelines for Condom Social Marketing)

Assessing the condom requirement at any given site of intervention is critical in order to ensure condoms are not being "dumped" or stock-outs are not occurring. Ultimately, condom availability depends on the risk profile of the individual site and cannot be averaged/aggregated at the State level.

The following formula can be used to calculate condom requirement for a FSW at a given site:

$$D = (S \times I \times N) - C$$

where

- **D** is the condom requirement
- **S** is the number of FSWs operating in the area
- **I** is the number of sex acts per day
- **N** is the number of days that a sex worker is "active" in a given month
- **C** is the number of condoms brought by clients from other sources

S, I and N can be determined through the processes of site assessment and outreach planning. **C** can be determined by local SMOs, through special surveys of FSWs. If such surveys have not yet been carried out, the NGO/CBO can estimate the proportion of condoms brought by the clients by polling a random sample of FSWs.

Establish distribution channels – Key channels for ensuring condom distribution to FSWs include:

- **Direct distribution** – Condoms given directly to FSWs are more likely to be used and less likely to be wasted
  - Distribution by PEs and ORWs in the field
  - At the DIC
  - At the STI clinic
- **Indirect distribution** – Locations should be chosen carefully to minimise wastage or the chance of the condoms being sold
  - Condom outlets (e.g. public toilets, petty shops, tea shops, lodges)
  - Condom stockists from the sex circuit (e.g. lodges, bars, brothel madams, brokers, auto drivers)

Monitoring condoms occurs at three levels:

- **Monitoring distribution/availability** – This can be done at the PE level to ensure that the all high-risk acts are being covered by distribution channels. Availability of condoms at hotspots, especially beyond 9:00 p.m., should be measured by State by an independent research firm. The target is to ensure over 80% availability.
- **Monitoring accessibility** – This can be done in a variety of ways, including condom depot monitoring and individual tracking through PEs **(see Annexure 5, *Peer Led Outreach and Planning*).**
- **Monitoring usage** – This can be done through PEs, used condom depot (counting used condoms at sex work sites and matching with estimated sex acts), peer counsellors at the clinic.

The basics of social marketing (SM) of condoms

Condom social marketing follows two paths vis-à-vis FSWs/MSM/TGs:

a. NGO/CBO enables availability of socially marketed condoms at hotspots

SM aims to make sure that different brands of condoms (preferred choices) are available at/near pick-up points/places of sex (hotspots), including bars and lodges where sex work takes place. The SMO should prioritise all hotspots in towns with over 50 FSWs/MSM/TGs. Only following this should the SMO target urban and semi-urban areas where there are fewer FSWs/MSM/TGs. If the client or FSW/MSM/TG wants a different brand, it should be available within 5 minutes' walk from the place of solicitation/place of sex. NACO/SACS are collaborating with SMOs to promote SM of condoms. It is suggested that NGOs/CBOs coordinate/collaborate with the SMO within their project area to ensure that condoms are being stocked at hotspots. The role of ensuring that condoms are available at hotspots lies with SMOs. NGOs/CBOs engaged in TIs can enable this by:

- Identifying locations/areas where availability of condoms should be ensured and passing the information to the concerned SMO
- Providing feedback to SMO on a regular basis regarding availability of condoms and incidents of stock-outs in the intervention area
- Sharing information with SMO on newly identified sex work locations and new hotspots as and when identified
- Creating awareness of the availability of condoms among FSWs/MSM/TGs and clients
- Meeting periodically with SMO to share the field realities and for further improvement

b. CBO/collective sells socially marketed condoms to HRGs

In select cases, where established demand from the community requires it, NGOs/CBOs may decide to provide socially marketed condoms to FSWs/MSM/TGs to supplement an SMO's marketing efforts. It is anticipated that 70%-90% of condoms for FSWs/MSM/TGs will be available for free, and only in select locations (10%-30% of FSWs/MSM/TGs) will condoms be socially marketed.

The following points must be kept in mind when involved in SM of condoms:

Dos	Don'ts
- SM to FSWs/MSM/TGs should be implemented only if strong demand from the FSWs/MSM/TGs arises, and only if the willingness to pay for condoms is expressed by a large subset of the population<br>- Even if socially marketed condoms are being made available to the FSW/MSM/TG population, the free supply should not be pulled from the market – those FSWs who cannot afford them should always have access to free condoms<br>- CBOs should be given preference for SM, rather than NGOs. Profits or subsidies from from SM should be retained by CBOs as development/seed money<br>- SM will only be introduced after careful examination of number of potential sites<br>- Condom gap analysis (as per the section above) should be conducted. The free supply should be mapped against this, and only the gap should be filled by SM.<br>- SMO must deputise its own team to stock and verify the condom availability to the CBOs<br>- A separate cadre of FSWs/MSM/TGs should be employed to "sell" these condoms<br>- SMOs must build the capacity of CBOs or collectives and FSWs/MSM/TGs before launching SM	- SM must never be mandatory for NGOs/CBOs – providing condoms for free to FSWs/MSM/TGs is NACO's policy<br>- Staff (e.g. PEs and ORWs) who distribute free condoms during outreach should not be employed to distribute socially marketed condoms, to avoid creating confusion among the FSW/MSM/TG population<br>- SM and brand promotional activities (e.g. street theatre to promote condoms) should be handled by the SMO, and not by NGOs or PEs<br>- The accounting of SM money should not be mixed up with the TI budget but be handled independently by an external agency<br>- Fixing of SM targets for NGOs by SACS, or targets for FSWs/ MSM/ TGs by NGOs, should be avoided, as it creates a disincentive to ensuring free condom supply to those who most need it<br>- Performance rating of NGO field staff or FSWs/MSM/TGs should not be based on SM performance

Condom stocking/reporting

- Each implementing NGO should make sure they have an adequate stock of condoms. Re-ordering is recommended when there is 3 months' stock in hand.
- NGOs should have adequate storage space for condoms. Care should be taken that they do not get damaged in storage or during transit to outlets.
- Documentation of condom supplies should be ensured. TI partners should be able to provide data on where, when and how many condoms are supplied.
- When assessing condom requirements, one should factor in the condoms required for condom demonstrations and trainings.

Special studies to assess condom use

- Special studies can be carried out regularly to assess the changes taking place among FSWs/ MSM/TGs in knowledge, attitude, and practice with focus on negotiation skills about condom use.
- Condom programming should be assessed as part of the annual review/evaluation and appropriate redesigning done accordingly.

Condom breakage during anal sex and the importance of lubricants/lubrication

A common complaint by HRGs is "breakage of condoms". There are several possible reasons for breakage:

1. Poor quality of condoms
2. Condoms used after the expiry date
3. Incorrect use of condoms
4. Poor lubrication and use of incorrect lubricants

It is important to communicate that reasons 3 and 4 can be avoided by emphasising condom demonstrations and education on use of correct lubricants – **water-based, not oil-based.**

- Evidence suggests that most MSM use saliva as a lubricant. This is not optimal since saliva dries rapidly, becoming sticky, which thus can increase the level of friction and result in increased damage to the anus.
- Other forms of lubrication that are used include vaseline, ghee, butter or some other oil-based product – these oil-based lubricants can damage the condom (by damaging the latex)

C. Communication for behaviour change

The evolving communication strategies of NACP I and II have contributed to a significant increase in awareness about HIV infection, but this has not been matched by corresponding behaviour changes regarding safe sexual practices and optimal utilisation of services.

One of the key gaps identified is in the area of helping FSW/MSM/TG groups put HIV/STI prevention messages into practice in their own very local or individual contexts.

A two-pronged approach must be adopted to create behaviour change **(see Annexure 6, *Dialogue-Based Interpersonal Communication By and With HRGs*)**

Continue to communicate messages to:

- Create awareness about the importance of using condoms for every penetrative sexual act, vaginal and anal, with clients or with regular partners
- Create awareness about utilising the services available for STIs, including the importance of regular screening, as well as other services like (ICTC, PPTCT, ART, partner notification)
- Create demand for services, e.g. condoms, STI services, other health services

Move beyond messages to encourage analytical thinking and problem-solving among individual and small groups of FSWs/MSM/TGs, so that they can arrive at and act on locally appropriate solutions to overcome their barriers to HIV/STI risk reduction, through peer facilitated, dialogue-based interpersonal communication (IPC).

Tools
NACO *STI Guidelines*
Annexure 5 *Outreach Planning and Management*
Annexure 6 *Dialogue-Based Interpersonal Communication By and With* HRGs

3.3.3 Step 6: Outreach Planning

The objective of outreach planning is to enable outreach to 80%-100% of the available FSW/MSM/TG population on a regular basis, in order to have maximum coverage and impact on HIV prevention. Outreach planning led by PEs is also a process for their empowerment which increases ownership of the project by the community and peers.

The elements of outreach planning serve the following purposes:

Objective	Quantifier	Tool
**Improve quality of outreach**	Reach all contacts at least once	• Spot analysis<br>• Contact mapping<br>• Geographic and social networks
	Reach all contacts regularly	• Sex work typology-wise outreach planning<br>• Site load mapping<br>• Seasonal calendar<br>• Force field analysis
**Improve service levels**	STI clinic attendance, condom distribution	• Preference ranking<br>• Peer map for condom distribution<br>• Condom accessibility and availability mapping
**Build PE capacity to monitor her/his own performance**	Monitors own performance and fills gaps proactively	• Peer Education card<br>• Peer calendar
**Continuously improve programming**	Uptake of services	• Opportunity gaps analysis

Annexure 5, ***Peer Led Outreach and Planning***, **provides details on implementing these processes.**

Tool
**Annexure 5** ***Peer Led Outreach and Planning***

3.3.4 Step 7: Community Mobilisation

This includes building community-led service delivery and building community based organisations (CBOs). Creating community norms is important to sustain behaviour change among individuals in any community. Community mobilisation in an HIV/AIDS programme context mainly aims for collective actions and also to influence norms within the community for safe sexual behaviour and to address other structural barriers. A community mobilisation process should provide opportunity to each and every community member in the project area to participate in collective decision-making on various issues that affect the community, by establishing successful democratic processes. It also should provide an opportunity to everyone to become the selected or elected leader or representative in various organisational/social forums.

The following table summarizes the ways in which NGOs can enable the process of community mobilisation and CBO formation.

Steps in Community Mobilisation within an NGO-led Intervention Context	Major Activities
**Increasing peer engagement and FSW/MSM/TG involvement in service delivery**	■ Ensuring peer-led (rather than ORW-led) outreach<br>■ Sharing of programme budget by NGO with peers and community members<br>■ Formation of community committees (see below)<br>■ Ensuring community-friendly services, e.g. FSWs/MSM/TGs involved in selection of doctors or counsellors
**Networking within the community – moving beyond peers and building "community affinity"**	■ Conducting community meetings; specific activities can be developed to bring FSWs/MSM/TGs together in small groups initially, e.g.:<br>• Monthly meetings held by each PE with her/his contacts (60 as per guideline)<br>• Every quarter all PEs may bring their contacts together for a one-day event<br>• Involvement of FSWs/MSM/TGs in crisis response and management **(see Annexure 7, *Crisis Response System*)**<br>■ Legal support and literacy support for FSWs/MSM/TGs
**Increasing community ownership of the programme**	■ Ensuring peer progression (see Section 3.3.1.H above and **Annexure 4, *Peer Progression***)
**Improving governance/initiating CBOs**	■ Increasing membership of community groups or collectives through democratic processes<br>■ Capacity building of community groups, e.g. literacy, financial management

A. Community Committees

Community Committees (CCs) are a model for empowerment of HRGs as well as a key tool for effective provision of services. As such, they should be formed in close consultation with members of the community, and the structures, roles and responsibilities of the committees and their members should be evolved by the NGO/CBO jointly with the community members.

For an overview of CCs and the process for their formation and maintenance, see Annexure 8, *Community Committees*.

B. Collectivisation and CBO development

Community mobilisation processes should be aimed at developing formal democratic community structures. The processes for transitioning and building CBOs are outlined in Chapter 5.

**Tools**	
**Annexure 4**	***Peer Progression***
**Annexure 7**	***Crisis Response System***
**Annexure 8**	***Community Committees***

3.3.5 Step 8: Creating an Enabling Environment

Providing services, supplying condoms and raising awareness may not by themselves result in sustained behaviour change. TIs must also address barriers to change and work towards creating an enabling environment that ensures the right conditions for change among individuals and the community. It is critical to advocate with policy makers, law enforcers and opinion makers to ensure a supportive environment for intervention.

A. What vulnerabilities do FSWs/MSM/TGs face?

FSW/MSM/TG vulnerability can be broadly divided into two categories:

1. Vulnerability within the sex circuit, e.g. violence, exploitation by clients, harassment by police, etc.
2. Broader socio-economic vulnerabilities, e.g. poverty, illiteracy, lack of savings

Both these vulnerabilities need to be addressed in order to enable FSWs/MSM/TGs to negotiate safer sex.

B. Stakeholder/Power analysis

The most important step in creating an enabling environment is a careful analysis of the power structures in which FSWs/MSM/TGs are involved. This analysis must be peer-led in order to be effective. It has to identify, and strategise to address, the various stakeholders who influence FSWs/MSM/TGs, whether directly or indirectly, positively or negatively. These are the people whose support can help to create an enabling environment for the TI.

The following diagram depicts some of the possible different stakeholders for a TI with FSWs.

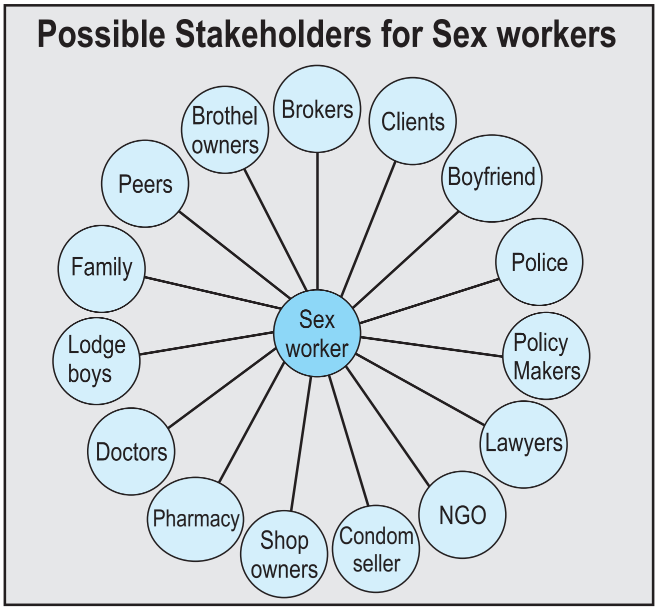

A stakeholder analysis could use the following table, which indicates the steps in the process leading to a strengthened enabling environment. It will be seen that specific strategies have to be designed for each stakeholder to solicit positive support from them for the TI.

Possible Stakeholders	Power/ Influence	Expected role in intervention	Planned strategy/ Activities	Expected Output
**Peers**				
**Brothel owners**				
**Brokers**				
**Clients**				
**Boyfriend**				
**Police**				
**Policy makers**				
**Lawyers**				
**NGOs**				
**Condom sellers**				
**Shop owners**				
**Pharmacy**				
**Doctors/Health care Providers**				
**Lodge owners/Boys**				
**Family**				
**Others**				

For a detailed tool on power analysis, see Annexure 9, *Power Analysis*.

C. Crisis response system

Harassment and violence towards FSWs/MSM/TGs are common and this causes a significant barrier to the HIV/AIDS outreach work of the project. When the obstacle of violence and harassment is removed through timely and proper crisis management and regular sensitisation and advocacy programmes, it creates an environment that is conducive to the FSW/MSM/TG, building up their self-esteem, which in turn helps them to focus more on their health specifically in relation to STIs and HIV/AIDS.

As part of a TI, crisis response interventions increase outreach to members of the HRG, thereby strengthening the NGO's or CBO's relationship with them and gaining their trust. Crisis response also facilitates the establishment of a good rapport between field workers and members of the HRG, which helps communication about prevention and treatments of STIs.

Essential ingredients of effective crisis management include:

- Trained and committed staff members who are willing to be "on call" 24 hours a day and to respond immediately when a crisis happens
- Effective communication mechanisms (i.e. crisis phones)
- Availability of information about crisis response to community members
- Experienced and committed lawyers who are willing to provide assistance 24 hours a day
- Networking, alliance-building, and sensitisation work with local stakeholders (especially the HRG) through regular meetings and education as appropriate. This includes community-level legal literacy sessions.
- Close alliances with other civil society organisations, activists and local media contacts who can advocate on behalf of the community when necessary
- Reflections on crisis management cases to improve and build internal capacities

See also Annexure 7, *Crisis Response System*.

D. Police advocacy

Police advocacy is usually a critical component of efforts to create an enabling environment for FSWs/MSM/TGs. It is critical to seek support of the police since their support or hindrance directly and indirectly influences the lives (and therefore risk behaviours) of FSWs/MSM/TGs.

The process for police advocacy should follow the following steps:

1. Start from the top if possible, with the SACS/TSU approaching the DGP, ADG (law and order), ADG (training), etc. to explain the HIV situation in the State, why we need to work with FSWs/MSM/TGs and what concrete support we need from the police. The goal is to get facilitating directives from the top police officers at the State level to the District SPs and city Police commissioners to support HIV interventions with FSWs/MSM/TGs, and from the training wing of the police to enable the project

to conduct police sensitisations. Facilitating directives can secure District police support along the following lines:

- Nodal officers from the District police should be designated by the DGP/ADG (training) for each District to be invited to the Traning of trainers (TOT) and coordinate sensitisation training at the District level
- Ensure signing of ID cards of project staff and personnel by SP
- If a FSW carries a condom, this should not be considered as a reason to arrest her
- Take proactive action against perpetrators of violence against FSWs/MSM/TGs (e.g. domestic partners, rowdies)
- Immediate action on complaints about violence against FSWs/MSM/TGs
- Ensure humane and friendly attitudes and treatment if a FSW/MSM/TG is arrested/brought to the police station
- Follow human rights laws/guidelines with FSWs/MSM/TGs
- Provide necessary support to the project's crisis response system
- Be part of the DAPCU and provide necessary support

2. Police sensitisation at the District and town levels is done through a step-by-step approach:
   a. First at the State level through a training of trainers (TOT) and then at the District level at the concerned police stations
   b. Involve police proactively in the training so that it does not appear to be just an NGO-led effort, e.g. inviting senior police officers to the training for the inaugural session, involving trained police officers as resource persons for the training
   c. Prepare a multi-disciplinary team of trainers for each District, comprising the trained police officer/nodal officer, NGO staff, PEs, lawyers, etc. through the TOT. Interaction between the police and NGO staff and PEs is very useful in developing mutual understanding.
   d. The trained multi-disciplinary team to conduct District-level training at identified police stations. It is important to try to focus the training on police stations which are in hotspots, especially in big cities where there are many police stations
   e. The training must cover general issues of FSWs/MSM/TGs apart from HIV related ones
   f. Advocate simultaneously at the PS, subdivision and District levels for tangible support of the HIV programme, e.g. ID cards for the PEs if possible (or recognition of ID cards issued by NGOs), request not to arrest FSWs for carrying condoms, support for PEs' fieldwork, etc.
3. The entire police advocacy should be backed up by the NGO-supported and community-led crisis intervention team, including legal support.
4. The DAPCU can also be involved in this effort if the direct approach with the police does not work.

Tools
Annexure 7 ***Crisis Response System***
Annexure 9 ***Power Analysis***

3.3.6 Step 9: Linkages with Other HIV Prevention/Care Programmes through DAPCUs

TIs should not operate in a stand-alone manner. NACO/SACS are implementing various programmes in the fields of HIV prevention and care at the District level. The District AIDS Prevention and Control Unit (DAPCU) is a nodal agency at this level. DAPCU will be an independent body functioning in every District. It will consist of:

- District health officials
- NGO representation
- Representatives from FSW/MSM/TG communities

A. Role of DAPCU in TIs

The objective of DAPCU is to enhance all HIV related activities in the District and increase service delivery to FSWs/MSM/TGs. DAPCU will provide active support to all TIs in the District. All NGOs implementing TIs in the District will share their key indicators with DAPCU. DAPCU will be a part of a Joint Assessment Team (JAT) supportive visit to all TIs. DAPCU will provide District-level insight to all SACS every month and help SACS in formulating strategies for the District. DAPCU will coordinate between SACS and NGOs in implementing TIs in the District.

All the feedback given by DAPCU to SACS and TIs will be documented and will be used in implementing programme related activities in the District. SACS and all TIs will incorporate feedback given by DAPCU into their activities.

DAPCU should enable intakes/referrals from TIs to other HIV activities in the District, e.g. VCTC, DOTS, ART.

B. Role of DAPCU in enabling service linkages

DAPCU operates within the District Health Society, sharing the administrative and financial structures of the National Rural Health Mission (NRHM). While the Unit reports to and works through the Chief Medical Officer of the District for medical interventions, it is also responsible for non-health related activities such as Adolescent Education Programmes, supportive supervision of TIs, M&E and mainstreaming. These activities will be carried out through the office of the District Collector or the Zilla Panchayat.

DAPCU can be leveraged to provide the following services to FSWs/MSM/TGs and their families:

- Ration card
- Voter identity card
- Domicile certificate

- Admission to schools for children
- Health facilities without stigma and discrimination
- DAPCU will network with District administration, police, local leaders and community groups to address the issue of harassment of FSWs/MSM/TGs

The category-wise District-level staffing structure proposed under NACP III is:

Staff	Categories of Districts			
	A	B	C	D
District Programme Officer (HIV/AIDS)	1	1	1	1
Assistant-cum-accountant	2	2	1	1
M&E Assistant	1	1	1	
Support Staff	1	1	1	1
Additional Supervisors for NGO and Care & Support Programmes	2	2	-	-

3.4 PROGRAMME MANAGEMENT

3.4.1 Hiring and Training Staff

A. TI Staff Positions

As per the NACO HR policy guidelines, TIs have the following staff:

- Project Coordinator
- Counsellor
- Accountant
- Office Support Staff
- Doctor (part-time)
- Outreach Worker
- Peer Educator

B. Assessing attitudes and expectations

Appropriate staff recruitment in terms of attitude, knowledge and experience is essential for a successful project. Working with issues of sex and sexuality dictates that the members of the staff be comfortable with their own gender and sexuality. Sensitivity and understanding towards the targeted population is also essential. Staff recruitment should be balanced with respect to gender and should include professional personnel as well as community persons.

C. Establishing roles and responsibilities

Establishing clear roles and responsibilities will not only minimise confusion but can also add efficiency to outputs. The underlying component to establishing roles and responsibilities is flexibility. Staff should not grow accustomed to routine job duties and should be flexible when needed. As the project grows and intervention processes become sophisticated, staff should be expected to perform varying duties while multi-tasking. As an employer it is important to clearly communicate changing roles and responsibilities to your employees. The staff should be aware of what they are expected to do each time new duties are assigned. Furthermore, staff should be clear on the reporting line, i.e., knowing who will hold them accountable.

For details on staffing, roles, procedures etc., see the *NACO TI HR Policy.*

**Tool**
**NACO *TI HR Policy***

3.4.2 CMIS Indicators

Tools

Area	Indicators	Definition	Frequency of Reporting
**Peer Engagement**	Ratio of HRGs to peer educators	A key measure of adequate resources for peer engagement in outreach. Derived by dividing the size estimation of HRGs into the number of active, paid peer educators.	Quarterly
	Proportion of outreach contacts made by peers	Key measure of extent of peers leading outreach activities. Derived by dividing the number of individuals contacted through peers during the month by the number of individuals contacted during the month	Monthly
	Proportion of peers receiving STI consultations during the month	Key measure of peers as role models. Derived by dividing the number of peers receiving STI consultations during the month by the number of active, paid peer educators.	Monthly
	Proportion of peers receiving STI consultations who underwent internal/speculum exams	Key measure of peers as role models and early adopters of prevention behaviour. Derived by dividing the number of peers receiving STI consultations who undergo an internal exam with the number of peers receiving an STI consultation.	Monthly
	Proportion of peers receiving STI consultations	Key measure of peers as role models and early adopters of prevention behaviours. Derived by dividing the number of peers receiving at least one STI consultation during the quarter, by the number of active, paid peer educators with the programme at the end of the quarter.	Quarterly

Area	Indicators	Definition	Frequency of Reporting
**Service Uptake**	Denominator: Number of individual HRGs mapped (as per broad mapping estimate)	Total estimate of individual HRGs mapped in a specific geographical coverage area. Methods of size estimation studies include: mapping, PSA, capture and recapture methods. The standardised methodology used to conduct an estimation must be articulated by the group doing the size estimation, and the updated figure should be entered along with target group, source, month, and year of study.	One-time
	Proportion of denominator who are being contacted monthly	Measure of proportion of mapped high risk groups who are being contacted by the programme (through outreach) - the expectation is that all High Risk Group members should be contacted at least once a month. Derived by dividing the number of individuals contacted during the month by the denominator (the number of individual HRGs mapped by the project as per a broad mapping estimate).	Monthly
	Proportion of monthly risky sexual acts covered by free condom distribution through peers and depots	Key measure for determining coverage of risky sexual acts through free condom distribution, and if free condom distribution matches up with estimated need. Derived by dividing the number of condoms distributed through free condom distribution by the number of estimated monthly sex acts with clients. If there is a particularly high wastage factor in a particular area due to double usage and breakage of condoms, then the demand can be adjusted accordingly.	Monthly
	Proportion of denominator who have ever attended a programme, referral or outreach clinic	Key measure of broad coverage of STI services for HRGs. Derived by dividing the number of high risk group individuals who have visited all types of clinic (programme, referral and outreach) at least once from the beginning of the programme establishment, by the denominator (number of high risk group individuals who were mapped as per a broad mapping estimate).	Cumulative

Area	Indicators	Definition	Frequency of Reporting
**Service Uptake (continued)**	Proportion of denominator who come to the clinic every month	Key measure of monthly coverage of STI services for HRGs. Derived by dividing the number of high risk group individuals who have visited all types of clinic (programme, referral and outreach) at least once during the specified month, by the denominator (number of high risk group individuals who were mapped as per a broad mapping estimate).	Monthly
	Proportion of HRGs who come for STI check ups during the quarter	Measure of health seeking behaviour of community with respect to STI care. Derived by dividing the number of high risk group population individuals who received an STI consultation during the quarter by the denominator (number of high risk group individuals who were mapped as per a broad mapping estimate).	Quarterly
	Proportion of HRGs who come for STI check ups during the quarter who were treated (as % of those who came during the quarter)	Derived by dividing the number of high risk group population individuals who received an STI consultation during the quarter and received treatment by the number of individuals who received an STI consulation during the quarter.	Quarterly
	Proportion of monthly clinic visitors who are "repeat" (vs. first time)	Derived by dividing the number of clinic visitors who are repeat visitors (not making their first visit) during the month by the total number of clinic visitors during the month (sum of first-time and repeat visitors)	Monthly
	Proportion of individuals with repeat STI symptoms who visit with symptom duration of less than 7 days	Individuals making repeat visit for STI symptom (not first-time STI symptom visit) who report symptom duration as <7 days. This is a key indicator for assessing treatment seeking behaviour in the HRG population.	Monthly
	Proportion of HRGs receiving STI consultations who underwent internal exams	Key measure of adoption of prevention behaviour. Derived from dividing the number of high risk group individuals receiving STI consultations who undergo an internal exam by the denominator (number of high risk group individuals who were mapped as per a broad mapping estimate).	Monthly

Area	Indicators	Definition	Frequency of Reporting
**Enabling Environment**	Number of reported incidents of rights violations against HRGs	Rights violations include any incident that violates Indian law where one or more community members are subject to extortion, abuse, violence or unlawful arrest by police or goondas. This does not include incidents where the police might have acted as per provisions of Indian law. Tracking should be done regularly through peers and consolidated by the NGO in a separate register and the NGO should determine, in consultation with the community, if the reported incident is a rights violation before reporting it here.	Monthly
	Proportion of reported incidents of rights violations or violence addressed within 24 hours	Derived by dividing the number of reported incidents that are addressed within 24 hours by the total number of reported incidents of rights violations or violence by. Addressal of cases means that peers and/or NGO staff should meet with affected community members and the concerned police officials within 24 hours to register a complaint and arrange for appropriate legal help; in case of rights violations by goondas a desired action is to get a police case registered within 24 hours.	Monthly
	Proportion of denominator referred to VCTC	Derived by dividing the number of individuals referred to voluntary counselling and testing centres (VCTCs), by the denominator (number of high risk group individuals who were mapped as per a broad mapping estimate).	Monthly
	Proportion of denominator referred to ART	Derived by dividing the number of individuals referred for provision of antiretroviral therapy (ART), by the denominator (number of high risk group individuals who were mapped as per a broad mapping estimate).	Monthly

Area	Indicators	Definition	Frequency of Reporting
**Enabling Environment (continued)**	Proportion of denominator referred to DOTS	Derived by dividing the number of individuals who were referred to TB DOTS centres by the denominator (number of high risk group individuals who were mapped as per a broad mapping estimate).	Monthly
	Number of HRGs who have been assisted by TI to access any government service (e.g. ration card, voter ID card, BPL card, school admission, housing, etc.)	Approved government ID cards include ration card, voter ID card, or PAN cards. This is only meant to report cases where the project has directly facilitated the issuance of the ID card - where individuals have more than one ID card, please count as one. This indicator is to be monitored for increases over time.	Monthly
**Community Mobilisation**	Number of community groups or SHGs formed	The number of groups primarily organised to address issues important to the community (e.g. violence, financial security, education, advocacy, welfare, cultural arts, etc). Includes Self Help Groups, Community Based Organisations, and other community committees.	Cumulative
	Number of members who are part of SHGs or community groups	Includes membership of high risk group individuals in various groups that are primarily organised to address issues important to the community (e.g. violence, financial security, education, advocacy, welfare, cultural arts, etc). Individuals who are members of multiple groups should be counted only once.	Monthly
	Proportion of denominator who are part of SHGs/ community groups	This is a gross indicator for community participation across the entire high risk group denominator. Derived by dividing the number of high risk group individuals who are members of various groups by the denominator (number of high risk group individuals who were mapped as per a broad mapping estimate).	Monthly
	Number of meetings/ events held for >50 HRGs	The number of meetings or events held in one month for more than 50 high risk group individuals.	Monthly

Tools
NACO ***TI HR Policy***

3.5 COSTING GUIDELINES

The costing of TIs should follow the NACO Costing Guidelines. An excerpt of the current set of NACO Costing Guidelines is included here for FSW and MSM/TG TIs only – IDU costing is included as an annexure to this document (in Chapter 4, and Annexure 13, Modular Costing Framework for TIs).

If NACO issues new costing guidelines for FSW/MSM and IDU TIs in the future, they should supersede the guidelines included in this section.

Module A	PROGRAMME MANAGEMENT Unit		Cost Fixed	Cost Annualised	Calculation	
	A1	Recruitment cost	5,000		one-time	one-time
	A2	Salary Project Manager	8,000	96,000	pm	fixed
	A3	Salary Accountant	5,000	60,000	pm	fixed
	A4	Travel cost admin purposes	800	9,600	pm	fixed
	A5	Rent	4,000	48,000	pm	fixed
	A6	Office expenses	52,200	52,200	pa	fixed
**Module B**		**OFFICE SET-UP**				
	B1	Office infrastructure	20,000		one-time	one-time
	B2	Computer peripherals	40,000		one-time	one-time
**Module C**		**PROGRAMME DELIVERY**				
	C1	Salary Outreach Worker	5,000		pm	variable
	C2	Peer Educator	1,500		pm	variable
	C3	Counsellor	6,500		pm	variable
	C4	BCC development	10,000	10,000	bulk cost	fixed
	C5	Travel cost Programme	500		pm *OW	variable
	C6	Community Mobilisation:				
		GD/FGD	6,000	6,000	Per annum	fixed
		Community events	7,500	7,500	per annum	fixed
	C7	Enabling Environment:				
		Advocacy	15,000	15,000	per annum	fixed
		Networking	15,000	15,000	per annum	fixed
		Meeting immediate needs	15,000	15,000	per annum	fixed
	C8	Training of Peers	7,500	7,500	per annum	fixed
	C9	Training of Volunteers	5,000	5,000	per annum	fixed
	C10	Monitoring & Evaluation:				
		Baseline Needs Assesment	20,000		per annum	one time
		Programme planning for next year	5,000	5,000	per annum	fixed
	C11	PLWA Support	10,000	10,000	per annum	fixed

Module D	THEME SPECIFIC COSTS		Unit Cost	Fixed Cost Annualised	Calculation	
	D1	Lubricants	1,250		per 100	variable
	D2	IDU:				
		Detoxification	2,000		per case	variable
		Sub./detox	7,500		per case	variable
		Abscess MGT	600		per case	variable
		Needle syringes	750		per case	variable
		Service-Nurse	5,000	60,000	pm	fixed
	D3	Non-Core Groups:				
		Peer Educator	1,500		pm	variable
		Outreach Worker	5,000		pm	variable
		Incentives-ext. stakeholders	2,000	2,000	pa/per centre	fixed
**Module E**	**DESIRABLE COSTS**					
	E1	Hon. PD	40,000	40,000	pa	fixed
	E2	Insurance to staff	500	3,500	pa/per staff	fixed
	E3	Audio Equipment	20,000	20,000	one-time	fixed
	E4	AMC	6,000	6,000	pa	fixed
	E5	STD drugs	125		per case	variable
	E6	Salary Doctor	6,000	72,000	pm/part time	fixed
	E7	Health Camps	5,000	5,000	pa	fixed
	E8	Condom procurement	10,000	10,000	pa	fixed
	E9	Social Marketing	20,000	20,000	pa	fixed
	E10	Drop-in centres	30,000	30,000	pa	fixed
	E11	SHG formation	10,000	10,000	pa	fixed
	E12	SHG seed money	5,000	5,000	pa	fixed
	E13	Documentation	2,000	2,000	pa	fixed
	E14	Hon.GIPA	6,000	6,000	pa	fixed

A ready reckoner based on these guidelines has been created. This is a template only and should be customised to local intervention requirements.

Template for Costs of TIs Based on Different Population Coverage Sizes
The following costs have been arrived at taking into account the project costs for 12 months of all activities including those under desirable costs (which may not be required for smaller TIs). Judicious review of the project plan depending upon local situation should be made and the budget should be developed considering the needs of the programme. However, in the case of Core Composite TIs with Core groups (FSWs, MSM, IDUs), the cost calculation should be made taking into consideration the total population (core groups) proposed for coverage instead of the individual numbers of each population for coverage in the TI.

Cost in Comparision to Coverage of FSWs/MSM/IDUs

		Number of FSW/MSM/IDUs covered by TI			
**Category**		**400**	**600**	**800**	**1,000**
**FSW**	Rs in Lakhs	10.6	12.8	15.5	18
**MSM**	Rs in Lakhs	10.6	13	16	18.5
**IDU**	Rs in Lakhs	16.26	20.58	24.98	29
	Note: A sum of Rs Lakhs 0.85 is to be reduced from budget if infrastructure is already provided; baseline may also not be necessary				

Tool
Annexure 13 ***Modular Costing Framework for TIs***

3.6 EXISTING INTERVENTIONS

These guidelines have focused on **new** TIs through NGOs.

The steps for improving scale and scope of **existing** TIs will be slightly different.

For existing TIs, it is suggested that a formal review be carried out by an **Annual TI Evaluation Team** for stocktaking, and where necessary to redesign the TI to achieve maximum output according to NACP III. For details on this review, see **the NACO Annual Evaluation Checklist.**

The review focuses on the level and quality of the following key components and provides inputs into redesigning the programme accordingly:

1. Outreach
2. Programme service delivery coverage/gaps
3. Capacity of the TI team
4. Community mobilisation
5. Programme management systems
6. Financial system

Based on the satisfactory performance of the review, the extension of the TI in that particular project area can be decided upon.

The various tools and techniques suggested in the guidelines can be used to redesign the existing TIs to improve their overall quality.

CHAPTER 4

Operationalising Targeted Interventions for IDUs: Guidelines for NGOs

TABLE OF CONTENTS

4.1 INTRODUCTION

4.1.1 Who is the Audience for these Guidelines?

4.1.2 Operational Definitions under NACP III

A. Harm reduction

B. Injecting Drug User (IDU)

C. Targeted interventions (TIs)

D. Community outreach based interventions

E. Detoxification

F. Needle/Syringe Exchange Programmes (NSEP)

G. Substitution

H. Abscess management

4.1.3 Strategies for IDU Intervention

A. Harm reduction strategy

B. Commodities and services provided through the TI

C. Linkages provided through the TI

4.2 PHASE 1 OF INTERVENTION: START-UP

4.2.1 Accessing and Establishing Services for IDUs

A. Rapid situation and response assessment

Operating principles

Steps in rapid situation and response assessment

B. Population size related issues for TIs

4.2.2 Step 1: Community Outreach

A Who performs the community outreach?

B. Outreach Worker and Peer educator ratios

C. Conducting outreach

D. Outreach sites

E. Operating hours

F. Materials for outreach workers

G. Staff supervision

4.2.3 Step 2: Drop-In Centre (DIC)

A. What is a Drop-In Centre?

B. What does a DIC provide?

C. Setting up a DIC

- Location
- Infrastructure
- At the entrance/registration counter
- Medical room
- Medical equipment
- Risk reduction materials

D. Staffing

- Training and technical assistance

4.3 PHASE 2 OF INTERVENTION: SCALE-UP

4.3.1 Step 3: Needle/Syringe Exchange Programmes (NSEPs)

A. Objectives of NSEP
B. Delivery options
C. Operational hours
D. Provision of injecting equipment
E. Types of NSEP
F. Disposal of used needles and syringes

4.3.2 Step 4: Primary Health Care

A. Operating principles
B. Procedures
C. Infection control procedures

4.3.3 Step 5: Behaviour Change Communication (BCC)

A. Group education
B. Counselling

- Requirements for counselling
- Staffing

4.3.4 Step 6: STI Management

4.3.5 Step 7: Condom Promotion

4.3.6 Step 8: Referral Networks

A. Linking services and addressing the multiple needs of IDUs

- Accompanied referrals
- Referral networks
- DOTS services

4.3.7 Step 9: Creating an Enabling Environment

A Advocacy with law enforcement
B. Working with the community
C. Raising public awareness

4.4 OPIOID SUBSTITUTION THERAPY (OST)

4.4.1 Guiding Principles for Designing OST Programmes

A. Minimising Risk

4.4.2 Guiding Principles for Establishing OST Clinics

4.4.3 Minimum Standards for OST Clinics

A. Location

B. Infrastructure/requirements

C. Staffing

4.4.4 Accreditation

A. Facilities where OST can be provided based on accreditation

B. Process of accreditation

C. National Accreditation Committee

4.4.5 Implementation

A. Eligibility criteria for admission to OST

B. Initiating OST

C. Administering OST

D. Follow-up of OST clients

E. Record maintenance for OST

4.5 PROGRAMME MANAGEMENT

4.5.1 Recording Information Using CMIS

A. The CMIS protocol

4.5.2 Monitoring and Evaluation

A MIS system for monitoring

B. Indicators for IDU programmes

4.1 INTRODUCTION

The purpose of these guidelines is to ensure the delivery of quality HIV prevention interventions to injecting drug users (IDUs) and their sexual partners in India. The guidelines outline standardised operating procedures for implementing comprehensive HIV prevention services for IDUs and their sexual partners.

4.1.1 Who is the Audience for these Guidelines?

These guidelines have been developed with the following audience in mind:

- State AIDS Control Society Project Directors
- State AIDS Control Society NGO Advisors
- Organisations implementing harm reduction programmes
- Programme managers
- Harm reduction workers
- Health professionals/social workers working with IDUs and their sexual partners

It is recommended that all organisations using these guidelines consider each of the proposed elements in the context of the organisation's current environment.

4.1.2 Operational Definitions under NACP III

A. Harm reduction

Harm reduction is a framework in which effective HIV prevention can be carried out among IDUs and their sexual partners. Harm reduction means that there is an emphasis on short-term pragmatic goals over long-term idealistic ones. Harm reduction aims to prevent the transmission of HIV by reducing the harm associated with high risk behaviours such as sharing needles, syringes and other equipment for preparing and injecting drugs, and unsafe sexual behaviours. Needle and syringe exchange and oral substitution therapy are integral parts of the spectrum of harm reduction services: drug use counselling, needle and syringe exchange, oral substitution, primary health care, detoxification and rehabilitation, leading finally to abstinence from drug use.

B. Injecting Drug User (IDU)

IDUs are those who used any drugs through injecting routes in the last three months.

C. Targeted interventions (TIs)

Targeted Interventions (TIs) are prevention interventions that specifically address HRGs who are at risk of acquiring or transmitting HIV infection. The central purpose of TIs among IDUs and their sexual partners is to prevent transmission of HIV:

- Providing the essential means and services that IDUs and their sexual partners need to practise safe behaviours (injecting as well as sexual) to reduce transmission of HIV
- Creating an enabling environment, which not only does not place obstacles in the way of safer behaviours but also proactively supports the practice of safer behaviours
- Ensuring that the IDU community (including their sexual partners) as a whole are empowered to decide for themselves and are able to lobby and advocate for what they need

D. Community outreach based interventions

Community outreach interventions aim to cover hard-to-reach populations vulnerable to HIV by providing credible risk reduction information and the means for change to safer behaviour, and referring IDUs to drug dependence treatment, VCTC and other services. Reaching out to the sexual partners of IDUs should constitute an important element of this package – in particular women who are sexual partners of male IDUs.

E. Detoxification

Detoxification refers to the treatment of withdrawal from an opioid or sedative/hypnotic over a short period of time by the use of the same drug or a similar drug that alleviates the distress in decreasing doses. The objective of detoxification is to facilitate the patient's transition to a "drug free" state.

F. Needle/syringe exchange programmes (NSEP)

Sharing of injecting equipment places IDUs at high risk of contracting blood-borne viruses such as HIV, Hepatitis B and C. The primary purpose of the needle syringe exchange programme is to give IDUs the means to use a new needle and syringe every time they inject, in order to reduce transmission of these blood-borne viruses and thus infection rates for the community as a whole.

G. Substitution

Drug substitution means replacing the drugs an IDU is taking with another or similar drug. It may also mean using the same drug but taking it in a different way, for example, sublingual buprenorphine/ methdadone to replace injection of the drug.

H. Abscess management

Ulcer/abscess management is another key component of harm reduction strategy. An abscess is a pocket of pus that forms in an infected area, made of dead tissue, germs and white blood cells. Drug injectors often get abscesses on their arms or legs, mostly at injection sites, due to unclean injection practices or when they miss their veins and inject non-injectable pharmaceutical substances like spasmoproxyvon, which is an irritant to the soft tissues and causes damage. If the abscess is left untreated, further liquefaction of the tissue causes formation of more pus, often leading to gangrene. If the abscess is left untreated, the bacteria can enter the blood stream, causing a complication called sepsis. Abscesses that discharge spontaneously and are not dressed properly may attract flies that lay eggs followed by maggot formation in an individual who may neglect their own health.

4.1.3 Strategies for IDU Intervention

A. Harm reduction strategy

Harm reduction is suggested as the key strategy for intervention among IDUs and their sexual partners, especially to reduce the risk of acquiring and transmitting HIV. This is primarily done through a needle/syringe exchange programme (NSEP) and substitution therapy to bring about behaviour change from sharing of contaminated injection equipment to safer injecting and from injecting, to oral substitution and subsequently drug use treatment (detoxification and rehabilitation are provided through linkages with drug de-addiction centres). For safer sexual behaviour, condoms are promoted and outreach to sexual partners of IDUs is established. Self-care and life skill development among women who have male partners injecting drugs constitute an important intervention approach in this regard. There are three tiers of harm reduction. While NACP II focused primarily on Tiers 1 and 3, NACP III also focuses on Tier 2 – oral substitution therapy (OST).

The services provided by the TIs and linkages to other services are outlined in Figure 4.1 below.

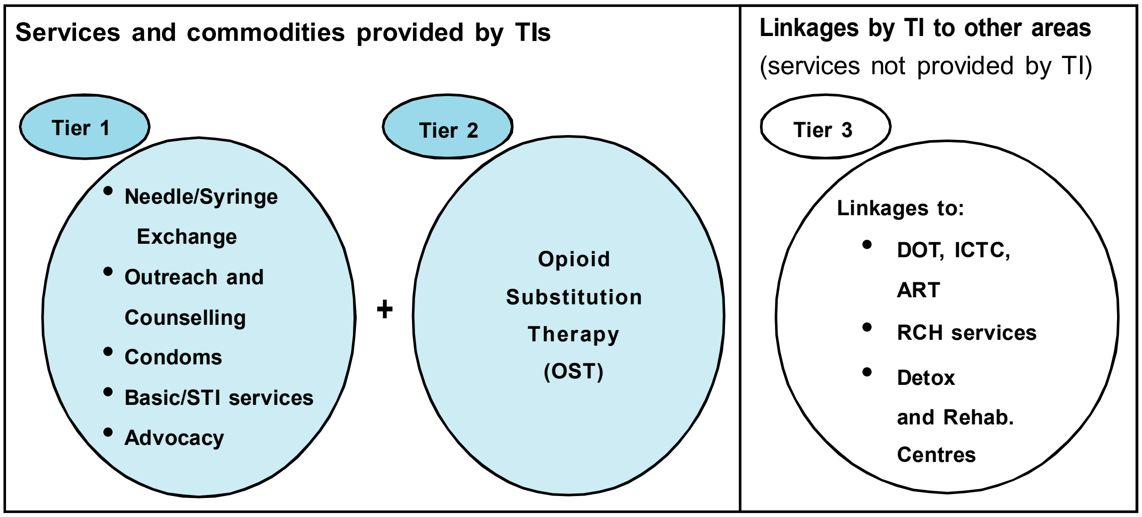

Figure 4.1 Tiers of Harm Reduction

TIER 1: OUTREACH	
■ Needle/syringe exchange programme (NSEP)<br>■ Outreach, Information and Education Communication (IEC), Behaviour Change Communication (BCC)<br>■ Free distribution and social marketing of condoms<br>■ HIV counselling for IDUs and their sexual partners<br>■ Primary health care (STI treatment, abscess management)	■ Needle/syringe exchange initiatives<br>■ Strengthening of BCC efforts for prevention of drug abuse, harm reduction measures and safe sex practices by developing IEC materials specific to IDUs, provision of training for NGOs, sub-group-specific materials (e.g. for women IDUs), region-specific IEC (language/dialect)<br>■ Free distribution and social marketing strategies to be implemented simultaneously for condom promotion<br>■ Primary health care, particularly syndromic STI treatment and abscess management

TIER 2:
OPIOID SUBSTITUTION THERAPY (OST)
(See Section 4.4)

TIER 3:
REFERRALS, LINKAGES WITH OTHER SERVICES, AND ADVOCACY FOR AN ENABLING ENVIRONMENT

- Linkages with key health services
- DOTS
  - OI management
  - VCTC
  - ART
  - PPTCT
  - PLHA networks for home based care and support
- Linkages with the MSJE supported centres and other private detoxification and rehabilitation centres.
- Other linkages and referrals
  - Pyschiatric services within government settings and NGOs
  - Referrals to and linkages with information sharing, networking, referrals; maintain follow-ups to ensure a continuum of care

(Tier 3 continued on next page)

TIER 3 (continued): REFERRALS, LINKAGES WITH OTHER SERVICES, AND ADVOCACY FOR AN ENABLING ENVIRONMENT
● Referrals from other TIs to address substance abuse issues among FSWs, Truckers and MSM<br>● Short-stay/Half way Homes and Night Shelters for availing safe environment<br>● Advocacy and creating an enabling environment<br>■ Advocacy with government ministries and agencies, e.g. Department of Social Justice and Empowerment, Police Administration, Paramilitary, Army, Human Rights Commission including local level PRI<br>■ Development of a State-level coordination committee involving stakeholders, public health experts, corporate representatives, State government departments and other agencies operating in drug related areas<br>■ Development of the capacity of TIs in harm reduction by utilising regional harm reduction resource centres for capacity development of implementing NGOs<br>■ Operational research (e.g. focusing on female IDUs, BCC strategies)

B. Commodities and services provided through the TI

Commodities:

- **Needle/syringe exchange programme** (NSEP) to cover 80% of the IDU population
- **Free condoms** (to 100% of population)
- **Opioid substitution therapy (OST)** to at least 20% of the population

Services:

- **Community outreach** through peer educators supported by outreach workers engaging in communication with IDUs to reduce risk/vulnerability and provide requisite risk reduction materials
- **Women outreach workers** to reach out to women who have male injecting partners and provide them with self-care information and life skills and help them access reproductive health services
- **Primary health care** for abscess and wound management, STI treatment
- **Drop-in Centres** (DICs)
- **HIV prevention counselling**

Structural Interventions:

- **Basic advocacy**
- **Community mobilisation**

C. Linkages provided through the TI

These services are not provided by the TI, but rather by other departments (e.g. MSJE, ICTC)

Linkages with other HIV services:

- TB referrals to DOTS
- ICTC linkages (VCTC, PPTCT)
- ART linkages and Hepatitis C management
- OI management
- Existing support groups (NGOs/CBOs)

Linkages with other key health services provided

- Drug treatment (de-addiction and rehabilitation through MSJE)
- Reproductive health services for drug using women and women who have male injecting partners
- Psychosocial support and counselling
- Linkages with other departments
- Vocational training/income generation efforts
- Social and legal support services
- Access to other government department services (e.g. BPL, nutritional supplements)

Steps in Starting and Scaling Up Targeted Interventions to IDUs

1. Community Outreach
↓
2. Drop-In Centre
↓
3. Needle/Syringe Exchange Programmes
↓
4. Primary Health Care
↓
5. Behaviour Change Communication
↓
6. STI Management
↓
7. Condom Promotion
↓
8. Referral Networks
↓
9. Creating an Enabling Environment

4.2 PHASE 1 OF INTERVENTION: START-UP

4.2.1 Accessing and Establishing Services for IDUs

Before implementing services for IDUs and their sex partners it is important to understand the patterns of injecting drug use, risk behaviours and adverse consequences as well as the existing capacity and resources in the geographical location where it is proposed to implement the targeted intervention.

A. Rapid situation and response assessment

Key objective: In order to design and develop appropriate interventions, conduct situation and response assessment of IDUs and their sexual partners in a specified geographical location.

Operating principles

- Rapid – assessment to be done in a short span of time (3 months)
- Community participation – involvement of both current and ex-users to reach out to IDU networks
- Involvement of women to reach out to women having male partners who use drugs
- Multiple methods employed – both qualitative data (observation, in-depth interviews, focus group discussions) and quantitative data (survey) collected
- Data collected from multiple stakeholders (IDUs, sexual partners of IDUs, service providers, law enforcement and policy makers)
- Triangulation of the data for analysis
- Response developed based on the findings

Steps in rapid situation and response assessment

- Planning the assessment
- Team formation and training
- Fieldwork – collecting existing information; mapping the areas with high prevalence of drug and injecting drug use; qualitative and quantitative data
- Analysis of the assessment findings by the assessment team
- Designing and developing an action plan based on the assessment findings
- Once the assessment is completed, an intervention should be initiated based on the assessed scale of injecting drug use problems

For a detailed methodology on site and rapid assessments, see Chapter 2

B. Population size related issues for TIs

Based on analysis of cost per unit, TIs are recommended for a group only when the population size of that particular group is a minimum of 150 and a maximum of 300-350 in a District. In select locations, dispersed IDU populations exist, and these may be harder to reach. Where there are such small and dispersed groups of IDUs, access to services can be ensured in one of two ways:

- A TI can be assigned to an NGO working in this area to address IDUs mapped in the area. One TI can cover potentially several areas within the District to reach a minimum threshold of 150 IDUs.
- These dispersed IDUs can be reached out to by existing de-addiction centres (e.g. MSJE), with strong referrals to VCTC and DOTS.

Tool	
**Annexure 13**	***Modular Costing Framework for TIs***

4.2.2 Step 1: Community Outreach

Key objective: Reaching IDUs and their regular sexual partners with the prime objective of preventing the transmission of HIV and other blood-borne viruses by reducing needle sharing and sexual risk behaviours.

A. Who conducts the community outreach?

Community outreach should be conducted by peer educators (PEs) and outreach workers (ORWs) who should preferably be a mixture of both current injectors and ex-injectors. The key to peer education's influence on behaviour change is peers talking among themselves and consequently determining a course of action to resolve the problem.

B. Outreach worker and peer educator ratios

- Gender balance among the staff (women PEs to reach out to women IDUs and the female sexual partners of male IDUs)
- One PE to reach out to 40 IDUs
- One outreach worker for every 4 PEs
  - One female outreach worker for every 200 female partners of IDUs
- Working in pairs is to be encouraged

C. Conducting outreach

The approach to outreach is that women outreach workers and peers should reach out to female IDUs and the female sexual partners of male IDUs, and male outreach workers to male IDUs.

- Identify locations with high drug use/drug dealing
- Actively engage and involve peers and women outreach workers to ensure access to sexual partners of male IDUs
- Provide relevant, credible education
- Increase access to needles, syringes and condoms
- Increase access to drug treatment services including opioid substitution
- Increase access to counselling, VCTC, sexual health and other social, legal and health services
- Keep client confidence and be non-judgmental

The minimum amount of information that PEs should try to communicate during a contact is:

- Discussion of risk behaviours
- Explanation of ways to reduce risk
- Giving written information and materials
- Offer of referral information
- Ensure necessary administrative and legal permissions are obtained for conducting needle and syringe exchange
- Repeat outreach contacts as per pre-designed field visits based on identified "gaps" in outreach **(See Annexure 5, *Peer Led Outreach and Planning*)**

D. Outreach sites

- Drop-in centres (see Section 4.2.3 below for details)
- Mobile outreach units – e.g. mobile vans from local DHO which have been seconded to the IDU project by the local DAPCU to provide clinical access for IDUs in remote areas
- Primary health care facilities
- Places where IDUs and sexual partner/s congregate
- Spaces near locations where other medical and social support services are located

E. Operating hours

- Outreach should happen at times when IDUs congregate (e.g. to buy drugs). If this includes evening hours, appropriate measures should be taken to ensure safety of outreach and peer education staff.

F. Materials for outreach workers

- Identity card and photocopy of the legal permission from appropriate authority which allows carrying out of needle and syringe exchange programme

- Outreach kit to contain the following:
  - IEC Materials
  - Needle 26" and 24"
  - Syringe 2ml and 5ml
  - Disposable gloves
  - Puncture-proof container to receive used and returned syringes and needles
  - Condoms
  - Penis model
  - Scissors
  - Betadine Ointment and Lotion
  - Spirit
  - Water
  - Sterilised Gauze
  - Bandage

G. Staff supervision

Supportive supervision provides workers with the opportunity to actively review their work practices and seek advice, structure and direction from a more experienced worker. The aim is to support and develop workers in their role.

Debriefing is a supportive process which offers staff an informal opportunity to express their feelings, thoughts and reactions about an unpleasant, negative or difficult work experience to a colleague or supervisor. Debriefing:

- Promotes teamwork and trust amongst team members
- Promotes skill sharing and an opportunity to review work practice
- Should be immediate, informal and low key. This is important to prevent staff burnout

Tool
Annexure 5 ***Peer Led Outreach and Planning***

4.2.3 Step 2: Drop-In Centre (DIC)

Key Objective: To provide services through user-friendly centres/clinics geographically accessible to IDUs.

A. What is a Drop-In Centre?

A DIC is a doorway for IDUs and their sexual partner/s to a welcoming and caring environment. It is a hub for all services which an IDU can access as per his/her need and convenience. The centre

acts as the one point from which all prevention and treatment efforts are coordinated. DICs are of two kinds:

i. DICs for primary-tier (level 1) services like outreach, NSEP, abscess management, STI, BCC, networking and referrals
ii. DICs which qualify based on essential OST standards and can function as NSEP and OST DICs

B. What does a DIC provide?

- **Outreach** – outreach workers and peer educators will reach out to IDUs and their sexual partner(s) in their own environment on daily basis to build rapport and refer them back to the DIC
- **Needle/Syringe Exchange Programme (NSEP)** – IDUs can exchange their used needles for clean new ones at the DIC or at fixed outlets
- **IEC dissemination** – continued education through leaflets/pamphlets on STIs/HIV, access to other IDUs and PEs for one-to-one contact
- **Psychosocial support** – counsellor available to address issues on behavioural change and VCTC
- **Ulcer/abscess management** – treatment, diagnosis and management of abscesses by nurse/ doctors/field staff
- **STI treatment** – syndromic treatment as per guidelines
- **Condom programming** – promoting correct use of condoms and access to free condoms
- **Referrals** to VCTC, DOTS

In select cases, where the DIC meets the strict criteria outlined in the OST guidelines, a DIC can offer OST to its clients.

C. Setting up a DIC

Location

- The DIC should be located with easy access to congregation points of drug users
- The TI should have information about the services available in the surrounding areas, and extensive social mapping of the intervention sites should be done to identify the location of IDUs and community resources
- The "Three A's" must be kept in mind:
  - Availability (menu of services under one roof)
  - Accessibility (in terms of location and timings)
  - Affordability (cost to reach the DIC)

Infrastructure

- Sufficient space, i.e. at least three to four rooms, one large (for group meetings) while the others (for counselling, primary health care) may be smaller
- The centre should be properly ventilated, well-lit and clean
- Toilet

- Running water/soap should be available
- Basic furniture
- If possible, a TV and some light recreational reading materials could be provided

At the entrance/registration counter

- Table and chair
- One PE or ORW
- Registration book
- Accessible box of condoms
- Needles and syringes for exchange programme
- Puncture-proof bucket for used injecting paraphernalia, with disinfectant

Medical room

All commodities utilised in this setting should be disposable:

- Large room
- Table and chair for nurse and doctor
- Stool for patient to sit
- Stool for abscess management
- Steriliser
- Patient examination table
- Needle crusher
- Gloves
- Storage space for drugs, e.g. cupboard for OST, STI drugs and other material
- Waste disposal container

Medical equipment

- Stethoscope
- BP apparatus - Sphygmomanometer
- Thermometer
- Torch
- Tongue depressor
- Weighing scales
- Kidney trays
- Disposable gloves and masks
- Hydrogen peroxide solution
- Savlon solution
- Solvent ether spirit

- Povidone iodine solution
- Freshly prepared eusol
- Freshly prepared 1% Na hypochlorite solution
- Cheatles forceps in savlon solution
- Drums with sterile gauze, gamjee and bandages
- Sterile packets of catgut, ethylon, prolene, silk, etc.
- Autoclaved linen
- Sticking plaster
- 2% xylocaine without adrenaline
- Suture cutting scissors
- Post exposure prophylaxis (PEP) drugs with visibly displayed instructions
- Storage bins for hazardous waste
- Emergency lights
- Waste management/disposal equipment

Risk reduction materials

- Condoms
- Sterile needles and syringes

D. Staffing

- Doctor (General Physician) – Full-time (based on local circumstances, part-time doctors may be permitted with SACS approval)
- Nurse/counsellor – Full-time
- Outreach worker/peer educator – Full-time
- Female outreach workers for female partners of male IDUs – Full-time
- Office support as required

(See also Annexure 14, *Staffing and Running a Drop-In Centre*)

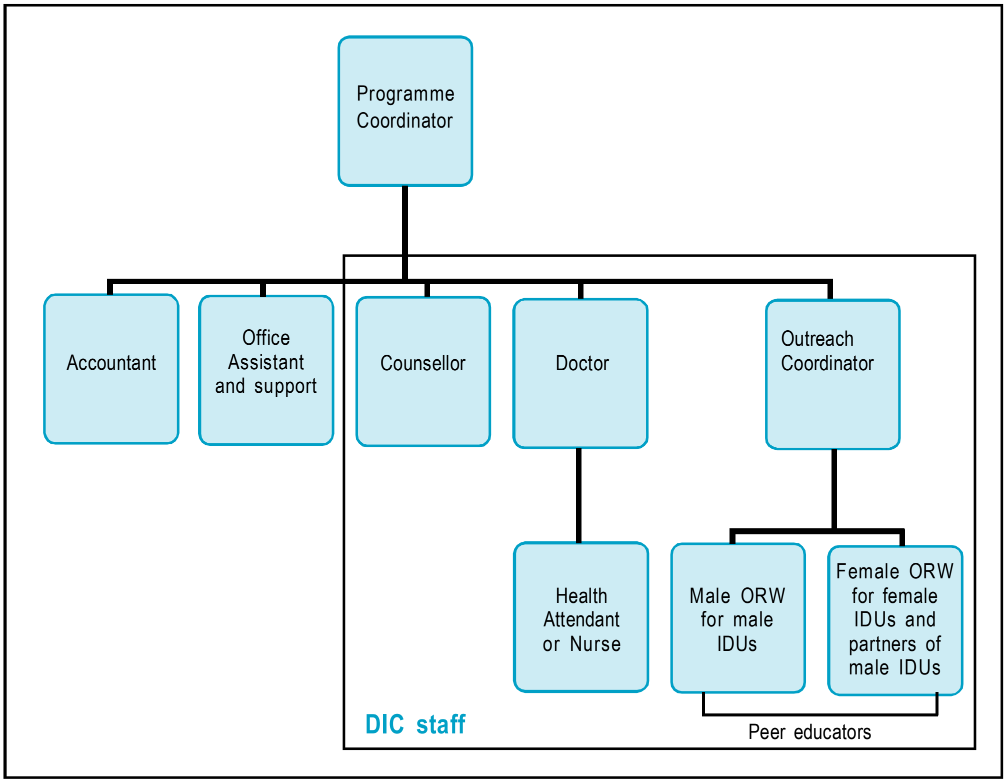

Figure 4.2 Staff structure for outreach and DIC support

Training and technical assistance

Training sessions are important to assist clinicians in making appropriate treatment decisions. However, patients look to non-technical staff to corroborate information given by physicians and paramedical staff. Further, patients expect the same accepting attitude from all staff members. Thus, all staff members need training in both medical and socio-cultural matters. Written educational/training materials for staff, such as national and regional treatment guidelines, should be made available and frequently updated. Close collaboration and coordination are needed between the primary care facility and the specialised HIV/AIDS care and treatment centre at the District/province level. Knowledgeable and sensitive health workers and others (ORWs and PEs) are needed to support vulnerable people living with HIV/AIDS and to maintain harm reduction activities. These workers and peers require ongoing training. All staff members require comprehensive training in various aspects relating to IDUs. These include:

- Safer injecting and sex practices
- Medical conditions associated with injecting drug use
- Medical conditions associated with HIV infection
- Managing drug dependency issues
- Infection control and universal precautions

- Procedures to be followed in case of medical emergencies (e.g., drug overdose)
- HIV treatment counselling

(Further guidelines on running a DIC are contained in Annexure 14.)

**Tool**	
**Annexure 14**	***Staffing and Running a Drop-In Centre***

4.3 PHASE 2 OF INTERVENTION: SCALE-UP

4.3.1 Step 3: Needle/Syringe Exchange Programmes (NSEP)

Key objective: To facilitate safe injecting practices by providing clean injecting equipment and ensuring safe disposal options for used equipment. In addition, education and information on safer injecting practices to help prevent transmission of HIV and minimise the potential health consequences of injecting is offered. **The goal of NSEP is to ensure that every injecting act is covered with a safe needle/syringe.**

A. Objectives of NSEP

- To distribute sterile injecting equipment to IDUs
- To remove used injecting equipment and paraphernalia from circulation
- To distribute other equipment related to injecting drug use:
    - Spoons, alcohol swabs, sterile water
    - Other materials such as condoms
- To establish point of contact with IDUs for dissemination of IEC materials
- To establish contact points for:
    - Counselling, VCTC
    - Primary health care
    - Referrals to other services
    - Engagement with drug treatment services

B. Delivery options

- DICs operate NSEP
- Needles and syringes can be delivered to IDUs by ORWs and PEs
- Satellite distribution of needles/syringes through secondary distributors (who have undergone thorough training on NSEP and counselling)

C. Operational hours

The service should:

- Operate seven days a week (if community outreach services are provided on alternate days at any particular site, adequate supply of syringes/needles should be handed over to cover for interim injecting episodes)
- Be open at times when IDUs most need the service
- Take into consideration how often and when IDUs in the area inject
- Keep in mind that it is easier to increase operating hours over time than to decrease them

D. Provision of injection equipment

Provide syringes and needles that are required by IDUs (e.g. 24" or 26" needles, as required by the users). On average, one syringe and one or two needles per day per person.

E. Types of NSEP

- **Fixed Site:** Specific place where IDUs come to collect and dispose of injecting equipment
- **Mobile Service:** Use of van or bus, often with regular route and stopping at several locations; ORWs and PEs travel the streets distributing clean injecting equipment and collecting used equipment for safe disposal.

F. Disposal of used needles and syringes

- Loose needles and syringes being returned to the NSEP should be placed by the client directly into a needles and syringes disposal container
- Any returned needle and syringe must be disposed of in the sharps bins, even if the client says they are new or unused
- The needle and syringe disposal containers should never be overfilled
- All needle and syringe disposal containers are to be stored at the DIC in a safe place for transferring to an approved medical waste service

4.3.2 Step 4: Primary Health Care

Key objective: To reduce morbidity and mortality among all IDUs and their sexual partners by early identification and treatment of infections and other drug use related illnesses.

A. Operating principles

- Provide essential health care universally accessible to IDUs and their sexual partners and families
- Care to IDUs and their sexual partners and other members of their family in the community in an acceptable and affordable way and with their full participation
- Should be delivered by health care providers who understand the health priorities of the communities they serve and have the confidence and trust of their clients
- Referral and linkages with other health agencies for treatment of conditions that cannot be treated on-site

B. Procedures

There should be specific guidelines and procedures regarding the operation of the primary health care services. All staff must be trained to follow these policies. These should include:

- Specific measures taken to ensure client confidentiality:
  - A client's right to accept or refuse treatment should be respected. Follow-up of clients who did not attend an appointment should not occur without the express consent of the client.
  - If the primary health care provider is to be involved in the daily dosing of patients with TB, DOTS/RNTCP guidelines should be followed and consent should be obtained prior to commencement of treatment
- Access should also be extended to sexual partners and family members, as well as the local community
- Addressing TB prevention and risk assessment for all staff of the DIC
- Occupational health and safety issues, especially related to the disposal of used injecting equipment
- Procedures in the event of an accidental needle stick injury, especially post-exposure prophylaxis (PEP) for HIV
- Immunisation of staff against hepatitis B (HBV) and tetanus is essential
- A clear statement that staff must not be involved in the procurement or distribution of illicit substances to, or from, clients
- Performance management and supervision of staff (in relation to HRGs, objective and general function of DIC)
- Clear guidelines on grievance procedures for both staff and patients to address conflict should it arise
- It is essential that all staff are offered, and have access to, regular in-service education and training

C. Infection control procedures

Simple infection control measures can reduce the risk of transmission of blood-borne pathogens through exposure to blood or body fluids among patients and health care workers. Under the "universal precaution" principle, blood and body fluids from all persons should be considered as infected with HIV regardless of the known or supposed status of the person.

See Annexure 15, *Universal Precautions and Post-Exposure Prophylaxis (PEP)*

**Tool**
**Annexure 15** ***Universal Precautions and Post-Exposure Prophylaxis (PEP)***

4.3.3 Step 5: Behaviour Change Communication (BCC)

Behaviour Change Communication is an interactive process with communities to develop tailored messages and approaches using a variety of communication channels to develop positive behaviours, promote and sustain individual, community and societal behaviour change and maintain appropriate behaviours.

- BCC with **IDUs** generally means attempting to persuade them to change their behaviour or to practice safe injecting behaviour
- BCC with **sexual partners of IDUs** means assisting them in acquiring knowledge on self care and acquiring life skills so that they can negotiate safer sex practices with their partners who inject drugs and also promptly seek reproductive health services as required

(For more information, see Annexure 16, *Developing a BCC Strategy and IEC Materials for IDUs*.)

A. Group education

Group education relies on the notion that IDUs form social networks, which can have a positive effect on their capacity to maintain safer behaviours. Women whose partners inject drugs also benefit from group sessions as these provide opportunities to learn from and be motivated by each other. Using the group education technique offers several benefits:

- The sharing of information within the group, thus respecting the knowledge, experience and skills IDUs and their sexual partner(s) already have
- Acknowledging that IDUs and their sexual partner(s) are a diverse group requiring education in a range of formats and styles
- Providing information in a way that enables users to pass it on
- Sharing power between IDUs and their sexual partner(s), rather than a formal programme which places some IDUs in a position of authority and knowledge over other users
- Women who have partners who inject drugs can reduce their own risk through a process of empowerment and negotiation skill building

B. Counselling

Counselling is a confidential dialogue between the client and a service provider (counsellor). This involves the assessment of risk behaviour, mental status, identifying problems together with the client and assisting them to take informed decisions about their future course of action. This should be done in a private place so that the information passed on to the counsellor can be kept confidential. The counselling can be individual or family counselling. A counselling session typically lasts thirty minutes to one hour. Repeat or follow-up counselling is important to reinforce information and to support and maintain behaviour change.

Counselling at the DIC must focus on HIV and be in accordance with the National Training Module developed for Counselling.

Counsellors should also make referrals to TB, VCTC, STI management, Hepatitis B and C services as required.

Requirements for Counselling

- Separate room to ensure confidentiality
- Table and two chairs for counsellor and clients
- Relevant IEC materials on display and free condoms for distribution

Staffing

- Counsellors with experience working with IDUs
- Female counsellors for women in drug use and partners of men in drug use

**Tool**	
**Annexure 16**	***Developing a BCC Strategy and IEC Materials for IDUs***

4.3.4 Step 6: STI Management

A TI should provide IDUs and their sexual partners with low-cost, good quality syndromic management of STIs at each intervention site. The provision of STI services for IDUs and their sexual partners follows NACO's guidelines for STI management **(similar to those for FSWs/MSM/TGs – see Chapter 3, Section 3.3.2.A)**.

4.3.5 Step 7: Condom Promotion

Ensuring availability, accessibility and correct and consistent usage of condoms by HRGs is a core imperative of NACP III. Free condoms for IDUs and their partners will be sourced to meet their expressed needs, e.g. condoms with extra lubrication and length.

The two broad strategies for condom promotion for IDUs

Primary strategy
Free supply of condoms to IDUs and their sex partners through TIs by NGOs/CBOs

Secondary (optional) strategy
Social marketing of condoms (for IDUs and their sex partners who are able to purchase condoms) by NGOs/CBOs in collaboration with Social Marketing Organisations (SMOs)

Condoms should always be available to IDUs and their sex partners for free. If and when demand for socially marketed condoms arises in these groups, appropriate mechanisms must be in place to ensure that the free and socially marketed supplies do not overlap.

- Ensuring availability alone is not enough – distribution does not ensure usage
- Ensuring accessibility is not enough – access does not ensure usage
- **The goal is increased correct and consistent USE of condoms by IDUs**.

For details on condom programming, see Chapter 3, Section 3.3.2.B.

4.3.6 Step 8: Referral Networks

Key objective: To ensure that IDUs and their sexual partners have access to the existing medical, social support and legal services.

A. Linking services and addressing the multiple needs of IDUs

A drug injector's life is complex and affected by multiple adverse social and health consequences. The IDU and his sexual partner may require many things: primary health care, shelter, drug abuse treatment, food, HIV counselling, employment opportunities, Hepatitis B and Hepatitis C and antiretroviral treatment, and recreational opportunities. Many agencies offer these services, and coordination between the various agencies ensures that IDUs and their sexual partner(s) are able to access them. It is important to link the various agencies offering help and provide coordinated services to IDUs.

Accompanied referrals

Accompanied referrals in which the ORW/PE accompanies the IDU or his/her sexual partner to the various services improves the relationship and trust between the IDUs or sexual partner and the ORW/PE.

Referral networks

It is important to network with all available services – medical, welfare and legal – that are relevant for IDUs and their sexual partner(s).

After providing necessary information, outreach workers may offer printed referral information on drug treatment, HIV testing, pre- and post-test counselling services, NSEP and other medical and social services. The objective is to provide IDUs with a specific agency and resource person for necessary information and support. Additionally, the information should include hours of operation, cost implication, eligibility requirements and an endorsement of the agency and the services it offers.

Linkages, if required, to MSJE-supported detoxification and rehabilitation centres can also be developed along the same lines as mentioned above.

DOTS services

IDUs are highly vulnerable to HIV infection as well as tuberculosis because of their low immunity and poor living conditions. VCTC and even outreach work are opportunities for screening and referral to DOTS services.

4.3.7 Step 9: Creating an Enabling Environment

A. Advocacy with law enforcement

Working with law enforcement agencies and gaining their support for harm reduction services is essential to avoid the police targeting IDUs or the staff working with them.

Key strategies include:

- Involving senior-level police officers in the planning and development of harm reduction services
- Letter of support from them for harm reduction services
- Education for police officers working in areas with known drug use/peddling
- Using supportive police officers in advocacy work with other police officials
- Periodic visits to local police stations
- Organising HIV awareness meetings with the police and the community

B. Working with the community

Involving the community in designing services for IDUs and their sexual partners is critical. It is important to consult with local community leaders and other health services in the area. When selecting the appropriate community stakeholders it is important to consider: Who are the supporters? Who are the opponents? Who are the decision makers? Who are undecided? Once these members have been selected, ask them to assist and participate in wider community consultation.

C. Raising public awareness

In order to reduce the stigma that is associated with injecting drug use and associated HIV, awareness programmes are necessary for the general population. They should be educated about the potential benefits of harm reduction programmes targeting IDUs and their sexual partners. It should be emphasised that harm reduction activities do not promote sex and drug use or drug injecting among those who do not use drugs, nor do they condone drug use by IDUs.

4.4 OPIOID SUBSTITUTION THERAPY (OST)

Agencies (public/private) implementing OST interventions under NACO must be accredited by NACO as per the guidelines below, and must have the statutory licenses/permissions required by law.

Key objective: To improve the quality of the life of IDUs by stabilising them and to transition them from the injecting mode of drug administration to non-injecting, thus preventing HIV and other blood- borne viruses. OST is a medical intervention in a clinic setting.

4.4.1 Guiding Principles for Designing OST Programmes

Issues of treatment safety must be addressed to minimise risk and avoid any potential harm from the treatment. Treatment should produce measurable benefit. Appropriate regulations exist at the national level to minimise the unintended consequences of treatment. Treatment must always be appropriate to the needs of the individual. Clients will be given a voice in the running of the treatment facility and be included in the planning and delivery process. In addition, the clinics will be run well and economically and remain responsive to the needs of their client population.

Quality of care will further be ensured if doctors routinely provide clear information to individual clients so that they fully understand treatment options. Confidence of clients will be enhanced by ensuring confidentiality, obtaining informed consent for VCT or HIV testing and for the disclosure of any information to a third party. Finally, all clients will be regularly monitored and evaluated to determine whether there has been any change of circumstances or change in health status which impacts the individual's progress and treatment services.

A. Minimising risk

- The National OST programme will be closely monitored and delivered by qualified personnel. This will minimise spillage into the black market
- Employing the DOT approach will ensure a close supervision of prescribing and dispensing of opioid substitution drugs, thus preventing overdoses or the spillage of drugs into the black market
- Patients will be educated and informed about the dangers of mixing prescribed drugs with other "street" drugs and will be made aware of the risks of additional unsanctioned drug use
- Methadone and buprenorphine must be transported and stored safely

4.4.2 Guiding Principles for Establishing OST Clinics

There are certain principles to be kept in mind when delivering OST:

- **Opioid Substitution Therapy is a medical intervention**, requiring medical assessment and ongoing medical supervision. Thus, for OST, health care professionals drive and lead the partnership with NGOs and CBOs. Roles for each player will be clearly outlined. The proportion of opioid users to be covered by the substitution programme can be reviewed periodically in different geographical locations.
- The government (responsible for supply of substitute medication and monitoring of regulatory procedures) and the NGOs involved in community based services for IDUs should become partners in the delivery of treatment
- The substitution programme should have linkages to existing drug treatment/rehabilitation services and should be part of a comprehensive continuum of care for IDUs
- In places with high potential for HIV transmission among injecting opiate users, substitution treatment should become a key component of HIV prevention strategies for IDUs. A broad range of dosages (with, if possible, a range of substitution substances – methadone and buprenorphine) should be offered in the clinics to match the profile of the patients
- Due consideration will be taken of local communities when locating clinics to ensure no public nuisance
- OST is a facility based programme, which should have close linkages with existing facilities for IDUs (e.g. DICs, space for group meetings) so that drug users have meaningful ways to network with each other after utilising OST services (e.g. to discuss their difficulties, make plans for their future)

4.4.3 Minimum Standards for OST Clinics

A. Location

OST clinic must be easily accessed from points where drug users congregate, as the drugs must be administered daily.

B. Infrastructure/requirements

- Separate space for clinical interview by staff, drug dispensing, and counselling where privacy for the IDU client is assured
- Adequate space for record keeping, drug storage
- Adequate and established mechanisms to ensure safe keeping of OST medicines. The mechanism for supply/storage/dispensing of OST medicines should be clearly established.
- Provision of condoms in spaces which are easily accessible to IDU clients

- Linkages should be established with centres offering other services to IDU clients. These may include:
  - VCTC and HIV counselling centres
  - ART and HIV related care for HIV infected clients
  - Detoxification centres
  - Hospitals/emergency rooms for management of overdose/complicated abscess/other general health conditions including tuberculosis (in case the OST staff is unable to provide treatment at the OST clinic)
  - Rehabilitation centres/programmes
  - Self help groups
  - Involvement and advocacy with local communities/leaders/law enforcement agencies
- Redressal mechanism for IDU clients should be established

C. Staffing

- One medical doctor with a minimum qualification of MBBS (preferably full-time. If part-time, back-up coverage for other days as well as absence/leave of the doctor should be established.)
- One nurse
- One peer counsellor or family worker
- ORW
- Support staff

The technical staff (doctor, nurse) should have received specific training on OST from agencies specified/approved by NACO. Other staff education and training is also mandatory as per NACO guidelines.

4.4.4 Accreditation

A. Facilities where OST can be provided based on accreditation

OST will be established in the context of health care facilities – including teaching hospitals, provincial and District hospitals and primary health care facilities

- Primary Health Centres/Health Clinics
- Community Health Centres (CHC)
- District Hospitals
- Medical Colleges
- OST will also be established in “informal” settings such as drop-in centres and other NGO/CBO facilities
  - DICs
  - Outreach community clinics (GO/NGO)

- OST services can also be delivered through established regional de-addiction centres and other specialised clinics providing HIV/AIDS prevention and treatment facilities such as ART clinics
- Other settings, e.g. prison

(All the facilities except prison should have provision for drop-in)

International experience suggests a maximum caseload per clinic will be around 200 clients daily.

B. Process of accreditation

Any of the facilities mentioned above delivering any OST service will mandatorily require accreditation from NACO. Any public or private sector facility that fulfils the basic minimum infrastructure, staffing and location norms indicated above is eligible to apply for accreditation by the National Accreditation Committee.

- Applications can be submitted by 30 October of any year to the NACO Programme Office on IDUs. **For details of the application form see Annexure 17.**
- These applications will be screened/reviewed by NACO by 30 November each year based on a checklist as per **Annexure 18**
- Those which meet the criteria will be visited/reviewed by a committee of NACO Assessors whose reports will be filed with the National Accreditation Committee
- The National Committee will review the reports and based on field/coverage requirements, will issue accreditations valid for three years, with renewal of contract by NACO on an annual basis and subject to evaluation
- Based on this accreditation the SACS can enter into annual contracts with agencies by the end of February to deliver OST as per the NACO NGO/CBO contracting guidelines, with validity from April to March each year

- **The accreditation certificate must be displayed in the clinic at all times.**
- **Non-availability or non-display of the certificate may lead to revocation of the license.**
- **Accreditation can be revoked if the facility does not conform to minimum standards.**

C. National Accreditation Committee

The National level Accreditation Committee will consist of:

- Drug Controller General of India (DCGI) or his representative
- Director General, Narcotics Control Bureau or his/her representative
- Director General, NACO as Chair
- Two technical experts to be appointed by NACO
- Two civil society representatives with experience in implementing IDU interventions

- Project Director of a SACS to be nominated by NACO (annual rotation)
- Programme Officer IDU will be the convenor
- Other members may be co-opted by NACO as required

**Tools**	
**Annexure 17**	***Application Form for Accreditation to Run OST Services***
**Annexure 18**	***Checklist for Scoring Proposals to Run OST Services***

4.4.5 Implementation

A. Eligibility criteria for admission to OST

- Diagnosed case of opioid dependence with injecting drug
- Age more than 18 years
- Failed detoxification
- Willing to provide informed consent for OST
- Deposit of Rs. 500 must be paid, refundable after completion of treatment (6 - 9 months). Family or partner to sponsor the applicant at time of admission.
- One month probationary period; absence of more than one week warrants disqualification without refund of deposit. Re-admission requires repeating the process above.
- Serious medical conditions (acute respiratory conditions, severe liver disease), pregnant and breastfeeding female clients, known hypersensitivity to buprenorphine are relative contraindications for starting OST treatment
- Only 20% of the IDU population can access OST at any point in time

B. Initiating OST

OST should be initiated by a physician trained in this treatment. The physician shall initiate treatment after taking the patient's history and performing a general physical examination to rule out serious medical illness. The goals of treatment should be clearly established with the client prior to initiation of treatment. An informed consent form signed by the client must be filled before starting OST. If facilities exist, a baseline haematological investigation along with liver function may be performed.

C. Administering OST

The drug is preferably to be given in substitution clinics by way of Directly Observed Treatment (DOT). This will ensure that the drug is not taken away, crushed and injected by the clinic patients. After recording dose details in the necessary documentation system, the following procedures should be observed:

1. Prior to administering the medication, staff must:
   - Establish the identity of the patient. An ID card should be issued by the TI to identify the client.
   - Gauge current usage of illicit drugs to determine eligibility
   - Check the quantity of the drug in the prescription
   - Check for current prescription
   - Check that the current day is a dose day on the patient's regime
   - Confirm the dose for the current day if it is an alternate-day or three-times-a-week regime
   - Record the dose in the recording system
2. Count and check the buprenorphine tablets into a dry dosing cup. Double-check the number and strength.
3. Crush the tablets into powder (to prevent diversion)
4. Place the powder under the tongue of the patient
5. Give the following instructions:
   a. Do not swallow saliva until powdered tablets have dissolved (2–5 minutes on average)
   b. Do not swallow the powdered tablets
   c. Once the tablets are given to you, they are your responsibility and will not be replaced
6. Observe the patient until you are satisfied tablets are not divertible (usually >2 minutes)
7. Ask to see "how the powdered tablets are dissolving" enough times for this to become an acceptable part of the patient's delivery routine
8. Patients should sign/affix thumb impression that they have received their dose. Offer water to rinse taste out of mouth.

The doctor should be notified if the dosing administrator has concerns that patients may be attempting to divert the medication.

D. Follow-up of OST clients

The client should be followed up by the staff on a regular basis:

- The physician should follow up with the client twice a month for the initial month; subsequently, monthly follow-up may be done. During the follow-up, the physician should enquire regarding the current status of drug use along with a general physical examination. The client should in addition report to the physician whether he experiences any side effects due to the OST drug. The follow-up details should be recorded in the client record.
- The counselling team should also follow up with the client regularly. The client should be provided counselling on various topics including HIV/AIDS, risk reduction practices (including safe sex practices), relapse prevention strategies, OST dos and don'ts. Counselling should be provided on a one-to-one basis as well as in group settings (group discussions and focus group discussions).

Additionally, the client may be encouraged to undergo VCT, and adequate pre- and post-test counselling should be provided. The treatment goals should be revised periodically by the counsellor and new goals may be set. Family members/spouse should be actively engaged in the treatment process and the progress of the client reviewed along with them.

E. Record maintenance for OST

- During initiation, a client intake form (details of socio-demography, drug use history, high risk behaviours, along with other clinical and psychosocial history details) along with a consent form.
- OST dispensing record: this should be strictly maintained. At the initiation, the physician should prescribe the drug and the record must be maintained in the client register form. During daily dispensing of the OST, the staff should maintain a register with the client dose intake. An opening and closing stock of OST drug should be maintained by the dispensing staff. In addition, the programme manager/project coordinator should maintain a register of the total stock received and supplied by him/her.
- Record of counselling conducted, including group discussions
- Records of referral made

4.5 PROGRAMME MANAGEMENT

4.5.1 Recording Information Using CMIS

Key Objective: To provide both quantitative and qualitative information which is precise, user-friendly and timely.

Given the importance of information gathering and analysis in determining the effectiveness of TIs, there is a need for a computerised management information system (CMIS) capable of generating information at the push of a button which can be made available to decision makers. A CMIS is most effective when:

- There is a consensus among all its users on its being a useful tool to assist in decision making
- There is a consensus on monitoring indicators and processes
- The data is shared with all stakeholders

A. The CMIS protocol

The CMIS seeks to record both the process and the outcome indicators of the TI and thus is divided into the following sections:

1. Behaviour Change
   - Outreach activities
   - Events
   - Group education sessions
   - Counselling
2. STI management
3. NSEP
4. Oral Substitution Therapy
5. Condom promotion
6. Enabling environment
   - Advocacy
   - Mainstreaming
7. Referral and actual access of services by those referred
8. Organisational Capacity
   - Governance
   - Structures and systems
   - Accountability
   - Capacity of the service providers

4.5.2 Monitoring and Evaluation

Monitoring systems and protocols make it possible to understand needs and analyse from the available data the impact of the project. Monitoring and Evaluation (M&E) forms an integral part of the TI and is essential to capture the progress of the project and gain feedback on its efficiency and effectiveness.

The objectives of this process are to:

- Ascertain whether a project is able to achieve its objectives in a given time frame
- Ascertain how this is being carried out
- Track the scope, quality, coverage, impact and success of the project
- Collect information on project input, process, output, outcome, and impact levels
- Clarify project barriers and successes, suggest new programme directions and inform resource allocation decisions
- Track activities of the project, milestones achieved, financial aspects, HR aspects, etc.

A. MIS system for monitoring

Figure 4.3 shows the flow of information for programme monitoring.

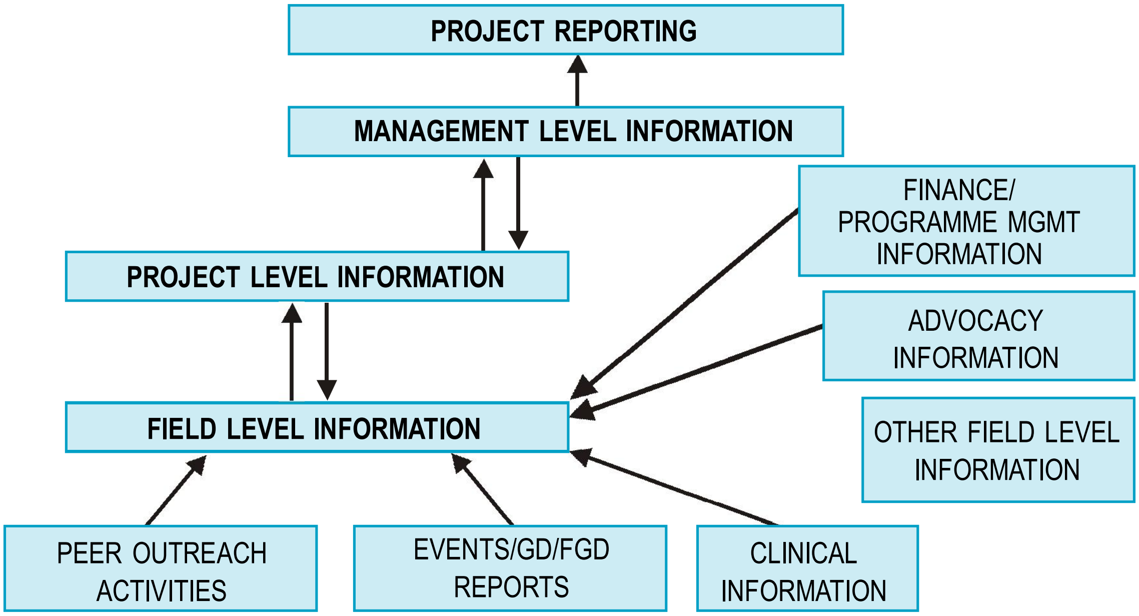

Figure 4.3 Suggested MIS system for monitoring

**Tool**	
**Annexure 19**	***Quality Assurance Protocol***

For details on programme management, see Chapter 3, section 3.4. The processes for programme management of TIs for FSWs and MSMs apply also to TIs for IDUs.

B. Indicators for IDU programmes

The following indicators will track IDU project performance at the TI level.

Area	Programme Component	Indicators	Description	Frequency of Reporting
**Coverage** (This is an indicator for programme coverage)		Proportion of clients accessing services	Calculated as total number of clients accessing any type of service (combined DIC and outreach) ÷ total number of clients in the area as allocated to TI	Quarterly
		Number of clients contacted in the reporting period	Refers to the number of individuals contacted by the PEs/ORWs for any outreach, service, or clinical activity. Calculated as total number of clients accessing any type of service (combined DIC and outreach)	Monthly
**Outreach** (Through peers and DIC)		Number of clients registered in DIC	This is an indicator of the number of clients accessing DIC voluntarily. Calculated as no. of individuals visiting DIC.	Monthly
		Proportion of outreach contacts (individuals met) made by Peer Educators	Calculated as number of individuals contacted by the PEs/ORWs ÷ total number of clients in the area as allocated to TI	Monthly
**Clinical**	*NSEP*	Proportion of clients accessing NSEP services	Total number of clients accessing NSEP ÷ total number of clients in the area as allocated to TI	Quarterly
		Total number of clients registered for NSEP (separately for the DIC and outreach activity)	Cumulative number of clients registered for NSEP	Monthly
		Number of *new* clients registered for NSEP in the month (separately for the DIC and outreach activity)	New additions on a monthly basis	Monthly
		Number of injecting occasions estimated by each peer educator(a)	Calculated by adding the sums of each peer educator's microplan estimate of injecting episodes in the month	Monthly

Area	Programme Component	Indicators	Description	Frequency of Reporting
**Clinical (cont.)**	*NSEP (cont.)*	Number of needles distributed by peer educators(b)	Calculated by adding the sums of each peer educator's needle distribution numbers in each month	Monthly
		Proportion of monthly injecting occasions covered with new needles and syringes through known NS distribution centres/ outlets	Calculated by dividing b (above) by a (above)	Monthly
		Proportion of needles and syringes returned – "needle exchange rate"	Number of needles and syringes returned ÷ number of needles and syringes distributed (combined DIC and outreach)	Monthly
		Average number of needles and syringes distributed to a client	Total number of needles and syringes distributed in the month ÷ Total number of clients registered for NSEP for that month	Monthly
		Use of safe needles and syringes (combined DIC and outreach)	Number of clients reporting use of clean needle and syringe during their last injecting episode (in the preceding 3 months). To be measured through focus group or one-to-one discussions every three months by peer educators/ORWs.	Quarterly
	Abscess management	Number of new clients who received abscess management in the month (separately for the DIC and outreach activity)		Monthly
		Total number of clients who received abscess management		Quarterly
		Number of clients who complete abscess management treatment		Quarterly
	Overdose management	Number of clients treated/referred by the centre for overdose management in the month		Monthly

Area	Programme Component	Indicators	Description	Frequency of Reporting
**Clinical (cont.)**	*Overdose management*	Total number of clients treated/referred by the centre for overdose management		Quarterly
	Condom usage	Total number of condoms distributed in the month (separately for DIC and outreach)		Monthly
		Proportion of risky acts covered through programme for free condom distribution	Total number of condoms distributed in a month through DIC, Outreach, Secondary Distributors ÷ Total number of estimated risky sexual acts among IDUs in a month (through peer microplans)	Monthly
		% of IDU clients reporting use of a condom the last time they had intercourse	Number of clients reporting use of a condom the last time they had intercourse (in the preceding 3 months) ÷ Number of clients reporting sexual intercourse in the preceding 3 months. To be measured through focus group discussions or one-to-one every three months by Peer Educators/ORWs.	Quarterly
	STI services	Total number of clients accessing STI services		Monthly
		Number of new clients accessing STI services		Monthly
	OST	Total number of clients registered for OST treatment		Monthly
		Total number of clients regularly receiving OST treatment (on OST drug > 80% days)		Monthly
		Number of clients completing OST treatment		Monthly
		Number of clients reporting injecting of drugs while on OST treatment		Monthly
		Number of clients who '"dropped out" of OST treatment		Monthly

Area	Programme Component	Indicators	Description	Frequency of Reporting
**Clinical (cont.)**	*OST (cont.)*	Proportion of clients re-employed or employed post-OST	Number of clients who have started employment or gone back to old employment after OST ÷ Total number of clients accessing receiving OST in that given quarter	Quarterly
**Referrals**	*VCTC*	Proportion of clients referred to VCTC	Number of clients referred to VCTC ÷ Total number of clients accessing any kind of services (separately specify for client receiving NSEP and OST)	Monthly
		Number of clients who reported having been tested in VCTC		Monthly
	Detoxification	Proportion of clients referred to detoxification	Number of clients referred for detoxification ÷ Total number of clients accessing any kind of services (may be separately specified for client receiving NSEP and OST)	Monthly
	ART centres	Proportion of clients referred to ART centres	Number of clients referred to ART centres ÷ Total number of clients tested positive and requiring ART treatment	Monthly
**Counselling services**	*Activity by counsellor (one to one interaction)*	Proportion of clients counselled by the counsellor	Number of clients counselled by the counsellor ÷ total number of clients accessing any type of services (specify separately for NSEP and OST)	Monthly
	Activity by outreach worker	Number of group discussions that are led by the outreach worker or other staff (counsellor)		Monthly
	Activity by peer educator	Number of group discussion led by peers		Monthly
	Regular access to counselling services	Proportion of clients having received more than 4 group discussions (separately by PE and other staff) per month	Number of clients who have received more than 4 GD (separately by PE and other staff) per month ÷ number of clients accessing services (specify separately for NSEP and OST)	Monthly

Area	Programme Component	Indicators	Description	Frequency of Reporting
**Counselling services (cont.)**	*Reaching out to family members*	Proportion of clients whose spouses were reached by the staff	Number of clients whose spouses were contacted ÷ Total number of married clients staying with their spouses	Monthly
		Proportion of clients whose family members were reached	Number of clients whose family members have been contacted ÷ Total number of clients accessing services (specify separately for NSEP and OST)	Monthly
**Enabling environment** (The data for enabling environment may be collected from the minutes of the meetings held with the respective group)	*Community meeting*	Number of community meetings held		Quarterly
	Advocacy meeting	Number of advocacy meetings held with the community leaders		Quarte.rly
		Number of advocacy meetings held with the local police station		Quarterly
	Self help group (SHG) formation	Number of self help groups formed in the centre		Annually
		Number of clients in the centre who are a part of the SHGs that exist in the community/ locality		Annually
	Harassment	Number of instances of peer educators/outreach workers/other staff/ clients (while accessing the services of the DIC) facing harassment in the field by the community members/ law enforcement agencies and steps taken to address the complaint.		Quarterly

CHAPTER 5

Development of CBOs and Community Led Responses

TABLE OF CONTENTS

5.1 INTRODUCTION AND RATIONALE

- **5.1.1 Why a Community Led Response for HIV Prevention?**
- **5.1.2 Current State of TIs**
- **5.1.3 Community Based Organisations**
- **5.1.4 Challenges**

5.2 STEPS IN TRANSITIONING AN NGO LED TI TO A CBO LED TI

- **5.2.1 Preparatory Stage**
- **5.2.2 Actualisation Stage**
  - Step 1: Seek endorsement from the community
  - Step 2: Prepare road map and build consensus
  - Step 3: Make structural adjustments
  - Step 4: Develop leadership
  - Step 5: Institutionalise community mobilisation
  - Step 6: Initiate process of transition
  - Step 7: Follow up and monitor

5.3 STEPS IN DEVELOPING A CBO LED TI *DE NOVO* (FROM SCRATCH)

- **5.3.1 Preparatory Stage**
- **5.3.2 Actualisation Stage**
  - Step 1: Lay down the management structure
  - Step 2: Build a common understanding of work, value, ethics and processes
  - Step 3: Build trust and confidence
  - Step 4: Mapping and enumeration
  - Step 5: Initiate formation of an enabling environment
  - Step 6: Lay down the components of intervention
  - Step 7: Process of ownership building

5.4 MANAGEMENT AND TECHNICAL SUPPORT

- **5.4.1 National-Level Support**
- **5.4.2 State-Level Support**
- **5.4.3 Technical Assistance**
- **5.4.4 Capacity Building and Training**
  - A. Capacity building plan
  - B. Resources
  - C. Training for NGO workers
  - D. Training for community members
  - E. Capacity building matrix

5.1 INTRODUCTION AND RATIONALE

Targeted intervention (TI) strategy in containing the spread of HIV among marginalised and vulnerable high risk populations has been an essential part of the National AIDS Control Programme (NACP). NACP III will continue to place emphasis on this approach but will use the lessons learnt from the past towards attaining scale, coverage and quality. This is expected to be achieved through the involvement and ownership of the at-risk communities themselves.

NACP II has been supporting TIs that are primarily implemented by Non-Governmental Organisations (NGOs) which reach groups at highest risk to promote safe behaviour, provide condoms and refer to services. NACP III will bring in the at-risk communities to play a more proactive role in implementation as community based organisations (CBOs), while the NGOs will continue to play a role as capacity builders and support agents, thereby putting the prevention responsibility on those who are themselves at risk.

The programme will be similar to that of the earlier generation of TIs (NACP II), but with a greater emphasis on community mobilisation, enabling community leadership development and community self-organising, so that the community takes the lead. Apart from developing the programme content and process to promote the programme on the ground (through community involvement, engagement and organising), there is a need to create community-friendly tools and systems along with high quality technical support in order to achieve NACP III's stated prevention objectives of containing HIV among populations at highest risk.

In this document, the term "community" refers to NACO's core high risk groups (HRGs) – female sex workers, men who have sex with men, transgenders and injecting drug users.

5.1.1 Why a Community Led Response for HIV Prevention?

**Achieving scale and coverage**	■ Makes HIV prevention a community priority<br>■ Uses organic bonding, through which individuals share emotions and understand/share responsibility so that their colleagues utilise services, etc.
**Improving quality**	■ Strengthens collective bargaining power, ensuring safe practices between people with unequal power relations<br>■ Community acts as pressure group to maintain and reinforce quality of services and not just as beneficiaries
**Sustain ability**	■ Each member shares responsibility for consolidation and continuation of intervention<br>■ Community takes active initiative to mobilise resources and evolve innovative mechanisms

The targeted intervention design of NACP III aspires to initiate and strengthen community led or community owned programming. This is intended to enhance the utilisation of services as well as create sustainable impact among high risk and vulnerable populations. This helps to make a transformation from service provision to demand generation, leading to greater utilisation of services and commodities. There are many evidence based examples available in the country to substantiate the importance and significance of community led processes in enhancing quality and coverage of preventive interventions. This shift in paradigm is essential to address the hitherto unaddressed aspect of scaling-up for saturation and coverage.

The community led process helps to make HIV prevention a priority issue for the community. Current NGO driven TIs do not aim for this objective. As long as programmes are driven by community members who are not themselves at risk, members of the at-risk population do not give adequate importance to the issue of HIV. Therefore they neither comprehend nor prioritise HIV and its prevention vis-à-vis their engagement in the programme. Community members only start to fully understand the issues once they obtain control and ownership over the processes of intervention. Thereafter, the community starts defining HIV prevention as its own agenda.

Community led interventions thrive through organic bonding among community members, where individuals take the initiative to support their colleagues in accessing information and services.

In addition to this, the process of communalisation strengthens collective bargaining power, which is immensely important in ensuring safer practices between individuals with unequal power relations. The dynamics of this approach lead to the programme's rapid expansion and greater saturation. This has been observed in many community owned interventions programmes steered by sex workers, MSM and the transgender community, in India and abroad.

Community-based organisations (CBOs) are found to be most effective in scaling up HIV prevention programmes covering large geographical areas and in dealing with various structural barriers. Coming together as a group helps members of marginalised communities strengthen their personal and social identity and enhance their self-esteem. This gives them confidence to negotiate with individuals, other social groups and institutions, and their collective strength can help them overcome difficult situations.

The Sonagachi project in Kolkata (inception 1992) was handed over to a sex worker organisation, DMSC, in 1999. DMSC was able to expand the programme in 45 red light districts in West Bengal within a span of two years. It played the role of lead agency in increasing coverage of the sex worker population in the State of West Bengal to 80% of the overall population size. A similar experience occurred in Bangladesh during the period from 2001-2003, when the programme was handed over to the sex worker organisation Durjoy. Following the transition, they took the initiative to scale up the prevention programme across Bangladesh, covering all the country's major sex work sites. The gay/MSM network has similarly played a significant role in expanding HIV intervention programmes among their community members, both nationally and internationally. One of the domestic examples is Humsafar Trust in Mumbai.

Community owned initiatives enable the HRG to play the role of a pressure group as consumer to maintain and reinforce quality services. The empowered community thereby plays the role of a "gatekeeper of services" and not merely that of recipients or beneficiaries.

Sustainability of a programme depends on various factors, including the ownership of the community. If community based organisations lead the process, individual members of the community share the responsibility to consolidate and continue. Programmatic sustainability is thus ensured as the community can take the initiative to mobilise resources or evolve innovative mechanisms to sustain the intervention effort. For example, even after the withdrawal of the donor's support, the Durjoy sex workers organisation was able to sustain basic minimum services through its organisational resource base.

It is important to understand and articulate the processes of community mobilisation and ownership building that lead to self-organising and the establishment of community based organisations. This document reflects the step-by-step approach in developing community based response and organisation building (CBO formation) through the use of community friendly tools, systems and technical assistance.

5.1.2 Current State of TIs

Targeted interventions have been supported primarily through SACS (over 700) and implemented by NGOs. There is a high degree of variability when it comes to community mobilisation and involvement of high-risk populations among the TIs. This variability poses certain challenges in shifting the paradigm of programme development and implementation. However, the steps towards enhancing community participation, mobilisation, involvement and taking the lead may be similar for different settings and stages, except in situations where TIs are yet to start.

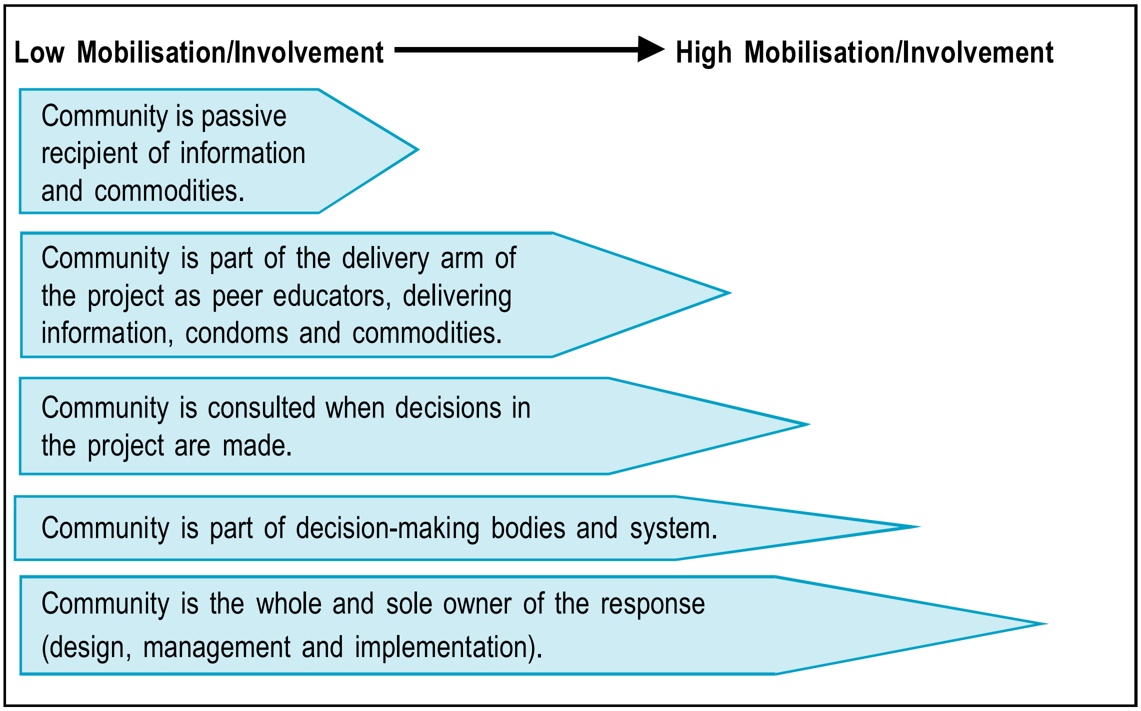

5.1.3 Community Based Organisations

Community Based Organisations (CBOs) bring the community together so that they are able to organise themselves. Thereafter the community is able to define its purpose in coming together and develop a process of institutionalisation through democratic mechanisms. This leads to articulation of their vision and mission, and the laying down of policies and principles to govern and run the organisation to achieve both short- and long-term goals and objectives.

What a CBO Is	What a CBO Is Not
■ A process of coming together of HRGs who share common threats and seek common benefits<br>■ The process brings incremental engagement of the community members over a period of time<br>■ The CBO moves through the collective knowledge and wisdom of the community<br>■ The CBO provides a democratic space for all members of the community to vent their views, choices and rights of participation in all decision making processes<br>■ Members are primarily accountable to the community<br>■ Office bearers of the CBO are members of the community and elected by the community<br>■ They plan for the organisation and steer the community's agenda<br>■ CBO enables, empowers and promotes egalitarian leadership as well as democratic functioning through which office bearers are changed after a certain period of time (e.g. the same individual is not to hold a position for more than two terms)<br>■ Accountability of CBO leaders is not just to the implementing agencies (NGOs) but to the community at large<br>■ There is a built-in system to develop 2nd/3rd generation of leaders<br>■ CBO empowers a larger, broader constituency of the community to exercise their rights	■ An organisation set up by an external agency (NGO) for the sake of project implementation<br>■ A small group of community members controlling all decision making processes with or without the support of the NGO implementing members<br>■ The NGO or implementing partners registering another organisation (Society) through inclusion of a couple of community members of their choice<br>■ Promoting a certain set of individuals<br>■ Office bearers overtly or covertly chosen by the NGO or implementing agency<br>■ Peer educators or outreach workers also being office bearers<br>■ Executive positions in the organisation occupied by community members through personal relationship or manoeuvring<br>■ The capacity building process directly or indirectly manoeuvred by a third party to keep control over the organisation<br>■ No efforts to develop second-line leadership – which maintains the status quo and impedes the empowerment process

5.1.4 Challenges

- HRG's social and legal status leads to denial of their rights and entitlements
- Lack of faith in the potential of HRG or community members
- Attitudes, prejudices and practices of service providers and society at large
- Difficult to work as a team with the HRG community because of less acceptance of the community's capacities
- Sensitising staff of NGO to change attitudes and develop bonding with HRG
- Repositioning of the role of HRGs in project management
- Lack of (or low) self-esteem of the community
- Formalising community's role and "positioning their representation" in the project management structure
- Orienting the implementing organisation to the importance of "taking the side" of the community
- Developing a process of "unlearning" followed by opening spaces for learning of new experiences in a non-threatening atmosphere

5.2 STEPS IN TRANSITIONING AN NGO LED TI TO A CBO LED TI

5.2.1 Preparatory Stage

Level	Steps
**At NACO Level**	■ Establish NGO selection criteria and select the NGOs<br>■ Develop a resource pool centrally<br>■ Integrate community led M&E within the existing M&E<br>■ Establish a monitoring system to gauge the progress of transition in consultation with SACS
**At SACS and TSU Level**	■ Discuss with all members of SACS and orient them towards CBO formation<br>■ Develop the State transition team<br>■ Prioritise and identify NGOs jointly with NACO<br>■ Develop an advisory committee with officials at District level to provide programme support<br>■ Train the NGO staff on their new skills
**At NGO Level**	■ Meet with board and staff<br>■ Redefine staff roles<br>■ Build staff skills on their new roles<br>■ Develop transition plan (refer to Actualisation Stage)

NACO, SACS, TSU and the NGOs (selected for transitioning) must undergo the above stages of preparation over a period of six months. A set of criteria for transitioning NGOs has been finalised by NACO. NGOs that have been implementing the TI for at least 3 years and have community members as peer educators for delivering information and other prevention services will initially be short listed. These short-listed NGOs will then be prioritised based on the following performance based criteria:

- Performance of the project implemented by the NGOs as measured through standard indicators provided by SACS/TSU
- Ability to create an enabling environment in and around the community
- HRG's overall presence in the project
- Implementing NGO's interest in and staff members' attitude towards building community ownership

Since there is variability in the TIs, the initiation should be based on the criteria stated above. Each of these criteria has a subset which has been put in a scoring sheet. ***(Indicators Scoring Sheet)*** This will be done at the field level by the NACO TI team, SACS/TSU. NACO recommends that this scoring sheet be strictly followed by SACS, TSU, NACO to select the NGOs for transitioning.

**Tool**
**Annexure 20** ***Indicators Scoring Sheet***

5.2.2 Actualisation Stage

Steps in Actualising the Transition from an NGO led TI to a CBO led TI

1. Seek endorsement from the community
2. Prepare road map and build consensus
3. Make structural adjustments
4. Develop leadership
5. Institutionalise community mobilisation
6. Initiate process of transition
7. Follow up and monitor

Guiding Principles

1. Should follow the principle of "of the community, by the community and for the community".
2. Should be based on community aspirations.
3. Should not be restricted to the peer educators of the project but draw as many community members as possible who aspire to take part.
4. Should assist the community to learn from the community: Draw upon the experience and resources of other CBOs that have proven experience of setting up as well as functioning.
5. Should do not underestimate community capacity, but at the same time invest in capacity building among the community members for self-organising.
6. Community members employed by the project should not be the office bearers of the CBO or in the governing body of the CBO.
7. Should ensure appropriate representation in decision making processes and the governing body of community members of different types, sects and locations.
8. Keeping in view the incremental engagement of community members, there should be a process for creation of space/positions through change in the management structure and functioning.
9. NGO's role is to move from being a benefactor to a partner and mentor.

Step 1: Seek endorsement from the community

Develop trust and instil confidence with community members. Constitute a core team of community members who will assist in transition

- Always adhere to the principle of giving respect and dignity to all community members. Establish through your actions that you respect their views
- Help existing peer educators to comprehend their new role as change agents, as opposed to health educators only
- Identify critical problems faced by community members, seek solutions from the community and help implementing activities through peers and other project staff members with the inclusion of interested community members

Initiate process of community mobilisation through sharing common threats and opportunities

- Define purpose of community mobilisation by identifying areas where community members and peer educators can find common ground
- Invest in community building so that community members develop unity, so that the entire community sees itself as a body and establishes itself as an occupational group as well as being citizens of the country
- Facilitate a series of consultative meetings on various topics to help the community develop a process of democratic decision making
- Help the community comprehend the shift in paradigm through vision building

- Initiate a process (series of meetings) that informs the community about the shift in paradigm through PEs, volunteers and staff
- Based on the suggestions, aspirations and dreams of the community, assist them in articulating the community vision through workshops

Step 2: Prepare road map and build consensus

Develop a road map / work plan with community members for transition planning and initiation

- Involve the community members in developing a work plan for action

Step 3: Make structural adjustments

Create positions in the project for community members

- In consultation with the staff, PEs and other community members, identify positions in the project (in outreach, services, coordination, etc.) for which community members can be selected
- Make the process transparent and give equal opportunity to all community members

Develop committees, designate authority and provide a budget

- Identify areas of the programme where the project can constitute committees with community members who will oversee implementation, e.g. DIC Committee, Health Committee, providing input on improvements, enhancing scope of utilisation, quality and community satisfaction, and addressing issues of discrimination
- Develop a project steering committee with representation of 33% HRG staff, 33% non-HRG staff and 33% community members who are not project staff
- Allocate the budget line for the components which are overseen by the committee and provide support through the project's human resource staff

Step 4: Develop leadership

Develop community structures and train leadership

- Help identify community members (PEs and non PEs) who have the potential (natural leadership qualities) to become community leaders
- Help develop community based structures (e.g. branch committees, SHGs, etc.) which have representatives drawn from different domains
- Promote leadership skills and build appropriate capacity for organisation building, conflict resolution, management of different processes and systems, etc.
- Develop skills in all community members (not just the chosen one or two) to represent the project and project its activities to the outside world

Step 5: Institutionalise community mobilisation

Facilitate formation of CBO with office bearers in a democratic way

- Through nomination, identify a working group drawn from several committees to develop the process of CBO formation
- This group will prepare the constitution and the election of the board
- Assist the group in holding democratic elections for the board
- Connect the group with a lawyer to develop by-laws and register the organisation
- Develop and promote capacity for CBO office bearers to manage the CBO
- Develop a work plan and capacity building plan for the CBO functionaries in CBO management
- Develop a capacity building plan for other set of community members to manage the intervention programme

Step 6: Initiate process of transition

Identify components that can be sub-contracted to a CBO and develop a joint proposal to SACS for the first round, or a new proposal to be managed predominantly by the CBO with the assistance of the NGO

- Work closely with the CBO management and develop a simple project that can be implemented by the CBO, e.g. running the DIC or implementing outreach, referrals, community led advocacy, etc.
- Transition the community members/committees from the NGO to the CBO and provide technical, financial and mentoring support for them
- Develop a joint proposal with full involvement of the CBO to be funded in the next funding cycle of the SACS, with clearly articulated areas that will be managed and delivered by them, and the role of the NGO delineated as capacity builder, facilitator and mentor
- Develop systems and support to enable the CBO to develop a stand-alone proposal for implementation in which the facilitative role and responsibility of the NGO is clearly laid out

Step 7: Follow up and monitor

Commission a joint monitoring process with clearly defined process and outcome indicators with SACS/TSU, with the inclusion of CBO representatives

5.3 STEPS IN DEVELOPING A CBO LED TI *DE NOVO* (FROM SCRATCH)

5.3.1 Preparatory Stage

At NACO Level

- Identify geo-locations and develop the support system
- Work plan with SACS of specific States
- Conduct mapping enumeration using NACO guidelines
- Develop centralised monitoring team

At SACS and TSU Level

- Discuss with SACS and orient them towards CBO led programme development
- Finalise geo-locations
- Discuss and define the support structure that will assist the programme development
- Develop a work plan at the SACS/State level and finalise contracting modalities

For initiating CBO formation and a CBO led TI *de novo*, NACO has developed a specific approach for District based programming in States (low-prevalence and highly vulnerable) where programmes need to be scaled up. District programming focuses on the community's needs and interests from the members' own perspective. It also promotes community building and a gradual development of ownership. The components of the programme will remain the same, but the strategy of implementation will be different:

- STI management will be provided either through static clinics (where number is more than 300) or by establishing strong referrals with public sector outlets
- There will be one District based strategy for advocacy with police as well as other stakeholders
- TSU will provide the lead in building capacity (in collaboration with appropriate CBOs identified by TSU), as well as in developing the advocacy strategy and establishing appropriate referrals to public service outlets
- The main responsibility of identifying the geo-location or District as well as to set up the process of initiation will also rest with TSU
- The NACO-TI unit and TSU/ SACS will jointly monitor the progress of CBO formation

The major tasks at the preparatory stage for initiating CBO formation and CBO led TIs *de novo* are to identify the process for initiation as well as finalise the geo-location. Following are the criteria developed by NACO for selecting the District or geo-location:

a. Size of HRG in the District
b. Cohesiveness among the community members
c. Incidence of violence, stability of the sex work site (for FSWs)
d. Absence of any parallel intervention at the same site, or level of intervention is very poor or terminated abruptly for some reason
e. History of collective resistance by the community against any form of injustice and discrimination, etc.

The NACO-TI team, SACS and TSU after the field visit, will also choose the geo-location for CBO formation. A scoring sheet has been developed based on these criteria. **(See Annexure 21, *Site Selection Scoring Sheet*)**.

NACO recommends contracting out the initiation of the process to an established external CBO with the credentials and capacity to develop a programme in the new location. If this option is not viable, TSU should contract professionals and community consultants who have done similar work (as a team) for a short term to carry out specific activities for programme initiation and CBO formation. Finally, linkages should be developed with community based networks, regional or national, from which support can be drawn as necessary.

5.3.2 Actualisation Stage

Steps in Actualising a CBO led TI *de novo* (from scratch)

1. Lay down the management structure
2. Build a common understanding of work, value, ethics and processes
3. Build trust and confidence
4. Mapping and enumeration
5. Initiate formation of an enabling environment
6. Lay down the components of intervention
7. Process of ownership building

Guiding Principles

1. Start with a belief that the community has the requisite potential and capacity to carry out the programme.
2. Right from the beginning, initiate processes which place the community in a proactive role and thereby develop the programme as a community led programme.
3. Constitute teams of resource persons (community and non-community) who have been involved in similar programmes to initiate the process of implementation.
4. Develop a common understanding of the work, values and ethics.
5. Transparent and honest with individuals, groups and power bases about who you are and what you can and cannot do.
6. For mapping, enumeration, needs assessment and power analysis, adopt a policy to select tools that will enhance community participation and facilitate data collection.

Step 1: Lay down the management structure

Form the team and prioritise the geo-locations

- SACS and Technical Support Teams (central and State-specific) prioritise the geo-locations where new interventions need to be initiated
- Compose a team comprised of community as well as non-community resource persons for initiation

Step 2: Build a common understanding of work, value, ethics and processes

Provide orientation to the composite team on their tasks and deliverables, as well as mapping, assessment and monitoring tools

- To all parties involved in the initiation of new interventions, provide an orientation to the new paradigm of programme development
- Develop "ground rules" non-negotiable principles in dealing with the community and other stakeholders
- Develop a short-term work plan (6 months) for programme initiation
- Give orientation on a basic monitoring format for gauging progress

Step 3: Build trust and confidence

Explore the local HRG community through repeated and regular field visits

- Place emphasis on exploring new contacts among community members through structured, regular field visits
- Consult with the community to identify key gatekeepers and facilitators, gain access to them, motivate and mobilise them
- Identify those with the potential to be guides or peer educators

Explore HRG's needs, try to address them and initiate mobilisation based on their perceived needs and rights

- Do needs assessment through constant interaction in groups
- Identify common issues that they need to address
- Facilitate a series of discussions within the community, identify rallying points and mobilise them around the same

Step 4: Mapping and enumeration

Develop ground rules and a set of operational guidelines to conduct mapping

- Generate consensus on the methodology
- Recruit community members as "community guides" and train them on the methodology of mapping and size estimation
- Conduct mapping and enumeration

Step 5: Initiate formation of an enabling environment

Identify structural determinants in HIV prevention programmes

- Do a power analysis and identify power structures and their role in influencing programme implementation
- Prepare a batch of community members to be advocates and train them accordingly
- Engage them in field based advocacy efforts, linking them with other community advocates through TSU, and design a District-level plan of action
- Discuss the outcomes of advocacy in wider community meetings and constitute a crisis management team from among the community

Step 6: Lay down the components of intervention

Initiate a service delivery mechanism from the community's perspective which increases access to and utilisation of services

- Initiate a process to continue regular consultation with the community
- Establish DICs and clinics to provide health services in consultation with the community members (re site, venues, timings, etc.)
- Gradually start delegating responsibility to service users though development of committees (e.g. DIC committee, clinic committee, etc.)
- Generate consensus to assess the quality of services
- Develop a system to build capacity of community members to manage clinic as well as basics of a community based STI management programme
- Develop a combined team with representatives from clinic and outreach to look for gaps in service at regular intervals
- Triangulate the data from the field with the clinic data and orient community members towards this process

Establish the outreach

- Train the PEs on i) dialogue based communication for STIs/HIV, ii) service utilisation, iii) community mobilisation, iv) advocacy and v) monitoring
- Orient PEs on community mobilisation and advocacy related activities
- Introduce community based monitoring systems and orient community members to deal with tools and interpretation of data
- Strengthen the network based outreach and start scaling up

Establish baseline markers

- Train guides/PEs/staff on the importance of baseline markers
- Generate consensus through a series of community meetings
- Develop "community watch-dogs"
- Conduct baseline survey
- Discuss results with the guides/PEs and help them to make changes in the programme if required
- Prepare the guides/PEs for wider dissemination of the results

Step 7: Process of ownership building

Help articulate policies/systems of management that will enable CBO to oversee intervention

- Assist in forming "committees" with clear-cut TORs for overseeing different aspects of programme
- Hold series of meetings with HRG members and not just guides/PEs
- Assist the community in identifying and promoting leadership as an ongoing process
- Assist CBO to monitor progress and functioning of different committees, and train them on their roles
- Invest resources in the committees
- Actively involve community members (in addition to two selected members) in advocacy with different power structures, including media

Facilitate formation of the self-run organisation

- This can happen at any time, depending on the community building skills, cohesiveness and maturity of the first-level leaders
- Hold series of meetings with the HRG and not just guides/PEs
- Bring HRG members from different places to facilitate the process
- Orient the team to take the side of the community in dealing with conflicting issues
- Facilitate a democratic process in terms of selecting the board as well as the type of organisation
- Train the community members on the importance of forming a CBO, the essential role of a CBO, types of organisation, etc.
- Elect the leaders, develop by-laws, get legal assistance for registration of CBO

Establish linkages and networking

- Based on needs and demands, identify appropriate agencies and establish services and other linkages
- Assist the local CBO to network with other CBOs in the same community (District, State, regional, national and international)
- Facilitate exchange of ideas and experiences with groups of marginalised communities to promote solidarity
- Establish linkages with rights based organisations engaged in the development field
- Establish linkages with professional institutions and legal organisations
- Engage with other civil rights movements in the country

Strengthen ongoing capacity building and skills development for the community

- Develop an ongoing capacity building plan for the CBO
- Develop plan to increase membership for the CBO
- Assist the new CBO in writing grants
- Conduct vision building exercise and assist in developing work plan
- Identify community members (PEs and non-PEs) who have the potential (natural leadership qualities) to become community leaders; promote leadership skills and build appropriate capacity to deal with organisation building, conflict resolution, management of different processes and systems
- Help in developing community based structures (e.g. branch committees, SHGs, etc.) with representatives drawn from different domains
- Develop skills in all of them (not just the chosen one or two) to represent the project and its activities to outside world

5.4 MANAGEMENTAND TECHNICAL SUPPORT

To bring about a smooth transition in programmes that are already being implemented as well as programmes that are to be initiated, a high level of efficiency is required in the provision of technical inputs (capacity to various constituencies and players), assistance and support for community mobilising and self-organising, and development and promotion of community-friendly systems.

Operationally, the transitioning and transfer of programmes, and the processes and capacity building to achieve this, will be carried out in select locations in select States. The locations where such measures are being taken will be those where interventions have been on the ground for a few years, as well as those where there is no intervention started.

About 500 TIs will undergo this process in the first phase (24 months) and an additional 500 interventions will go through this process in the second phase (from 24 to 48 months), such that almost 70% of TIs are assisted in going through transition or initiation to second-generation interventions by the fourth year of NACP III.

All new TIs will adopt the new paradigm of programme development and therefore will be fast-tracked to be run by community-based initiatives in each location.

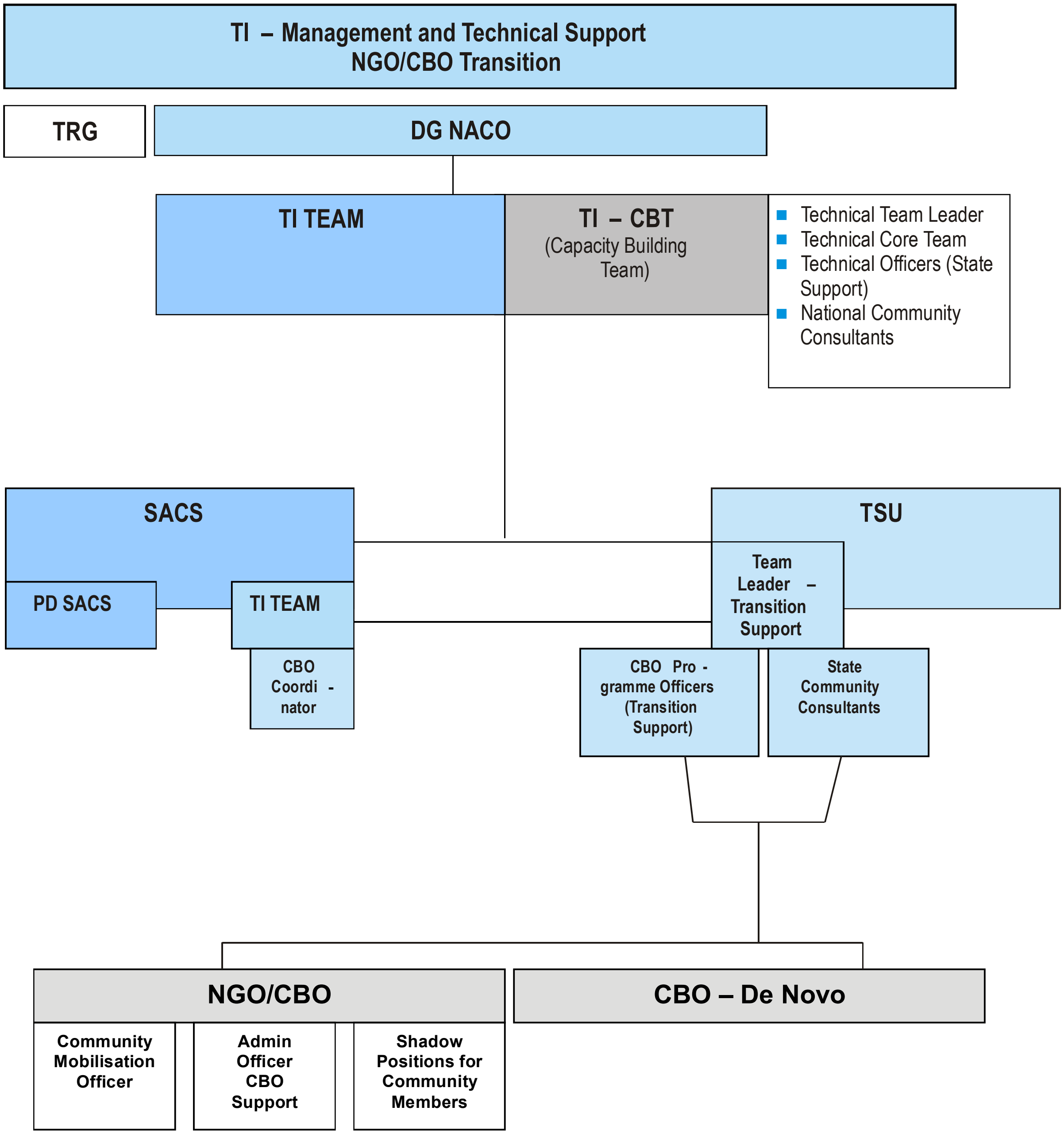
TI – Management and Technical Support
NGO/CBO Transition
TRG
DG NACO
TI TEAM
TI – CBT
(Capacity Building Team)
Technical Team Leader
Technical Core Team
Technical Officers (State Support)
National Community Consultants
SACS
PD SACS
TI TEAM
CBO Coordi-nator
TSU
Team Leader – Transition Support
CBO Pro-gramme Officers (Transition Support)
State Community Consultants
NGO/CBO
Community Mobilisation Officer
Admin Officer CBO Support
Shadow Positions for Community Members
CBO – De Novo

5.4.1 National-Level Support

The National Programme at NACO level will have a TI support unit. A core team that will provide high-level input to the TI support unit in technical and programme management will be institutionalised as the Targeted Intervention Capacity Building Team (TI-CBT). It will comprise a Technical Team Leader, Technical Officers (State Support) and National Community Consultants.

Technical Officers (State Support) should be placed as the national officers designated for delivering technical and management assistance in designated States with the cohort of organisations identified for the transition programming. A team of community consultants will perform the role of community advisors as well as community monitors who will be able to assess and give feedback to the Technical Officers (State Support) with the TI-CBT.

Main Tasks of TI Capacity Building Team

- Provide input to the SACS in the processes leading to transitioning
- Provide capacity to Technical Officers (State Support) and develop operational plans for each State and NGOs/CBOs
- Provide capacity to the Community Consultants and develop work plans for providing support and supervision to the transition
- Provide oversight to the overall operation under the leadership of a team leader
- Develop core documents and processes for overall consistency in the work plan for transitioning

Main Tasks of Technical Officer (State Support)

- To be designated for transitioning among a specified number of organisations (NGO/CBO) in designated States
- To provide hand-holding support to the State, NGOs and CBOs in transition planning and implementation
- To organise technical assistance in support of transitioning
- To interface with rest of TI-CBT to ensure that inputs are streamlined with the vision, mission and objectives

Main Tasks of National Community Consultants

- To be deployed to provide support for transitioning among a specified number of organisations (NGO/CBO) in designated States
- To provide technical input and hand-holding support to community members in taking a greater role in implementing the TI
- To monitor transitioning and engage in discussion with the Technical Officer (State Support)
- To interface with rest of TI-CBT to ensure that the objectives of transitioning are met

5.4.2 State-Level Support

Since the main implementation of transitioning and the creation of a community based response to HIV must happen at the State level among NGOs currently implementing TIs as well as new initiatives, a management and technical support arrangement is essential. In States where there is a well embedded Project Management Unit or Project Support Unit, or a State Management Unit is present, the transition management support process will be organised to have a close working relationship with the existing support. The State-level Management and Support Arrangement will also be linked to and work closely with the State AIDS Control Society.

This unit will be composed of a Team Leader (Transition Support) and a team of Programme Officers (Transition Support) and community members drawn from within the State as State Community Consultants (SCC). Each of the programme officers will be responsible for a specified number of NGOs/CBOs and will work closely with the NACO Capacity Building Team. The State Community Consultants will be assigned to a specified number of NGOs/CBOs and will work closely with the Community Consultants drawn from the National Team.

The Number of persons on the State Support Team will depend on the number of initiatives that are to undergo transition in a particular State. Initially, it is envisaged to work with about 25 States, covering 500 TIs where transitioning will take place.

5.4.3 Technical Assistance

While the Central and State Teams will provide support for transitioning, technical assistance will be assembled as the need arises. Organisations that are required to go through a rapid learning process will be provided opportunities to do study tours to sites that are to be supported through the National Programme. Teams of key HRG members from the community organisations may also be contracted from time to time to provide hands-on assistance where there is a need.

The key constituencies, areas and aspects of technical support that will build capacity are outlined here. A detailed capacity building plan should be developed in order to provide technical assistance. The intensity and nature of technical support for capacity building may differ between the group of NGOs already implementing TIs and the group that will initiate new interventions.

5.4.4 Capacity Building and Training

A. Capacity building plan

The main areas of capacity building will be the following (see details in capacity building matrix below):

- Vision building for the CBO/NGO
- Leadership development

- Management training
- Organisational development
- Conflict resolution
- Advocacy with different stakeholders
- Outreach based on programme components

B. Resources

- Guidelines for CBO formation – theoretical aspects
- Training module on CBO formation
- Operational Guidelines for FSWs/MSM/TGs, Chapter 3
- Participatory enumeration and assessment
- Training for peer educators in community-led programming
- Training on community led monitoring systems
- Training on community led advocacy

These resources are available and can be provided during the capacity building process.

C. Training for NGO workers

Classroom training

- Importance of community mobilisation and CBO formation
- How to initiate CBO formation
- Orientation on tools and methods for community led mapping, enumeration, needs assessment and programme development
- Community based monitoring systems
- Vision building workshop

Immersion visit

- 3- to 5-day visit to any of the recognised sites where CBOs exist and the community is taking the lead in project development

On-site training and capacity building

- A pool of resource persons will provide on-the-job support to NGO staff, showing them how to facilitate and be a mentor

D. Training for community members

Classroom training

- Training on CBO formation – why and how
- Community led mapping and enumeration

Immersion visit

- 3- to 5-day visit to any of the recognised sites where CBOs exist and the community is taking the lead in project development
- Various aspects of developing a community based organisation

On-site technical support for capacity development

- Vision building for the CBO
- Developing work plan
- Leadership development
- Management training
- Organisational development
- Office management
- Conflict resolution, etc.

E. Capacity building matrix (see following page)

The TI framework within NACP III emphasises seven components within TI projects, and also includes components of community mobilisation/ownership building and creation of an enabling environment.

Sl. No.	Skills Required	State-Level Coordinat-ing Agency	Point Person	Capacity Building Partners	Mechanism for Involvement	Mentors
1	Collectivisation and SHG building	Technical Support Unit	Community Mobilisation Officer (TSU)	■ Forums of HRGs which are already organised either within State or outside State, e.g. DMSC, Kolkatta, can be the capacity building partner for sex workers in other States; for MSMs, Humsafar trust can be the capacity building partner for MSM interventions in other States.<br>■ Partnerships can also be made with CBOs wholly or partly controlled by HRGs	Plan and budget for various activities including joint development of CBO modules, training sessions, visits, etc.	TSUs can suggest mentors from these organisations to TI projects, if there is a need. TI projects can choose their own mentors.
2	Advocacy, building linkages and alliances	Technical Support Unit	Advocacy Officer	■ CBOs and NGOs with proven track record of people-centred advocacy<br>■ NGO networks<br>■ Advocacy institutions	-do-	-do-
3	Special health services	Technical Support Unit	STI Management Officer	■ Professional organisations with adequate track record of helping the health system in delivering services<br>■ Organisations/NGOs with experience of making systems accountable through established mechanisms	-do-	-do-
4	Condom promotion	Technical Support Unit	Condom Promotion Officer	■ NGOs with adequate experience of condom promotion<br>■ Private sector organisations with expertise in condom promotion, especially social marketing	-do-	-do-
5	BCC	Technical Support Unit	BCC Officer	■ NGOs with adequate experience of BCC<br>■ Forums of HRGs for ensuring key approaches<br>■ Private sector organisations with experience of BCC	-do-	-do-
6	HIV care and support	Technical Support Unit	Care and Support Officer	■ Networks of HIV positive people, e.g. INP+, PWN, etc.<br>■ Organisations with experience of care and support interventions	-do	-do-
7	Management	Technical Support Unit	Project Management Officer	■ Management Institutes<br>■ Rural Development faculty in universities	-do-	-do-

Framework for support for CBO led TI

Level	Broad Areas of Support	Specific Areas of Support
**TSU**	Building an integral support system to facilitate the process of CBO formation	1. Identify capacity building partners, preferably a combination of a State/region-specific institute with a community based organisation/network, with delegation of specific roles and responsibilities.<br>2. Create a position in TSU of **CBO coordinator** who will:<br>a. Coordinate selection of NGOs, process of transitioning from NGO to CBO led programme and capacity building of CBOs, and monitor the progress of CBO development and help build linkages with other services, in addition to networking of CBOs both at District and State level.<br>b. Promote and support the process to incorporate community members in various decision making bodies within the projects, District committees, SACS and outside.<br>3. Create a position in TSU of **State Community Consultant** who will work as a team member with CBO coordinator.<br>4. Help strategise District- and State- level advocacy and negotiate the plan with the community led advocacy programme and its execution with relevant policy makers in support of CBO led intervention.
	Development of training materials	1. Translate CBO development guidelines and CBO development toolkit into regional language<br>2. Develop appropriate training module to impart knowledge and transfer skills to community members, e.g. in management, monitoring, etc.<br>3. Develop a new set of IEC materials addressing issues of stigma related to target communities.<br>4. Develop a new set of advocacy materials for use by the community based advocacy groups.<br>5. Develop community led monitoring tools to be integrated with the existing M&E tools.
**Project**	Management and style of functioning	1. Recruitment of **Community Mobilisation Officer** to help build self help groups and assist with organisational development, registration, managing offices, meeting minutes, etc. S/he will be focal person for capacity building of community members.<br>2. Selection and recruitment of 5/6 community members in addition to peers who will be developed as community mobilisers, with adequate knowledge and skills to deal with organisational issues including governance, conflict resolution, negotiation and network building.<br>3. Create horizontal structures across the line hierarchy (e.g. Project advisory committee, DIC management committee, clinic management committee, grievance redressal forum, etc.) with majority representation from the community members, clear-cut TOR and supportive budget for effective functioning.

Level	Broad Areas of Support	Specific Areas of Support
		4. Selection and recruitment of 4 additional members from the community: 2 will receive specific skill building in community led advocacy programmes, and 2 will work for community based monitoring, with a specific focus on the impact of the intervention.
**NGO**	Capacity building in transitioning NGOs	1. Vision building workshop (2 days) for the board members of NGOS with the project officials (who are likely to be inducted into the CBO led TIs later) and in the presence of community leaders.<br>2. Immersion learning (5-day programme) for community Mobilisation Officer and other potential members who could be inducted as the administrative officer in the CBO led programme.<br>3. A 2-day workshop to be held with the participation of NGO staff members with the core members of CBO to lay down the process of transition and transfer of ownership including the establishment of monitoring and supervision of the progress of transition through the creation of an independent review body.
**CBO**	Management structure and support system	1. Recruitment of **Administrative Officer** (for the first 3 years) to support running of the intervention programme and also to help guide the process of capacity building for the community members, so that over time community leaders will be able to administer and manage the programme in the Officer's absence.<br>2. A yearly grant of 2 lakhs will be provided to CBOs to run the process of collectivisation. In addition to running the provisional office, the CBO will receive 1.5 lakhs for capital expenditure (furniture and fixtures, computer and internet connection) in the first year.<br>3. Selection of 3 "shadow leaders" to work with the project manager, clinic in-charge and outreach in-charge.<br>4. Work with the community mobilisers recruited from among the community members, to reach the difficult-to-reach members of the community in addition to strengthening the collectivisation and organisation building effort.<br>5. Strengthen community-based advocacy programme through community members with close linkages with TSU, and by expanding their role at District level.<br>6. Integrate community led monitoring process with the ongoing monitoring system and enable a selected community member as a member of the monitoring team with delegated power and responsibility.

Level	Broad Areas of Support	Specific Areas of Support
**CBO (Cont.)**	Management structure and support system	1. Vision building workshop with 20 community leaders to clarify mission and vision of CBOs as well as their role in community mobilisation and management of HIV intervention programmes.<br>2. Immersion learning for 10 members (6+4) already selected for specified role in community mobilisation, community led advocacy programme and community led monitoring.<br>3. Capacity building of community members holding the position of project manager and senior-level positions in the project, as well as for shadow leaders, to cover management, administration and basics of financial management (week-long courses on each area).<br>4. Week-long training programme for 4 members who will carry out community led advocacy.<br>5. Week-long training programme to build capacity on community led monitoring, including power analysis.<br>6. Short-term placement of shadow leaders in well-established community led projects (1 month each)<br>7. Field visits for another 10 members of the community with a view to building their comprehension and confidence.<br>8. Development of community led monitoring tools (adapting existing one developed by Avahan) and creating a system of triangulation with State- and project-level monitoring.<br>9. Hiring of community leaders from other projects to provide hand-holding training for CBO members (2-3 months in each year for first three years)<br>10. Support community led advocacy programme, preferably at District level, dealing with different categories of policy members.<br>11. Support lead members of the community to develop networking with other CBOs in the District and also to develop linkages with other similar groups/organisations.<br>12. Capacity building of the various committee members (15-20 community members).

ANNEXURE 1

Broad Mapping

STEPS IN BROAD MAPPING

Steps		Lead Organisation
1.	Estimate time and human resource (local HRG consultants, and support staff) required for Broad Mapping	State/regional mapping organisation
2.	Selection, recruitment and contracting of local HRG consultants to do Broad Mapping	State/regional mapping organisation
3.	Training of HRG consultants and support staff	State/regional mapping organisation
4.	Forming site-specific mapping teams	State/regional mapping organisation
5.	Implementation of Broad Mapping	State/regional mapping organisation
6.	Feedback and analysis of data	State/regional mapping organisation
7.	Report on Broad Mapping with recommendations for TI locations	State/regional mapping organisation
8.	Finalising Districts where TIs are to be placed and configuration of the TIs	SACS and TSU
9.	Contracting of TIs	SACS

Purpose

A broad map provides HRG mapping teams with a geographical and social overview of a site, with landmarks in the areas and with location of HRGs in different parts of the site. Information about different categories of HRGs present in the site and about mobility patterns is also collected through this method.

Respondents

Mapping can be used with the general population as well as HRGs in the site. The mapping team can approach auto drivers, local shopkeepers, flower vendors, lodge owners, petty vendors, liquor vendors, and so on, near public parks and theatres, where HRGs gather. Anybody in the area who has good knowledge about the area and the HRG could be involved in drawing the map. This of course includes the HRGs themselves.

Location

Anywhere in the site – usually close to likely or obvious hotspots.

Process

1. Identify likely respondents. Start by asking them about the site and requesting them to draw the geographical outline of the area.
2. Spread the chart on the ground and hand over sketch pens to the respondents to draw maps on the chart.
3. Request respondents to mark the important landmarks in the area.
4. They then mark the specific locations or hotspots where HRGs are available.
5. While the respondents draw the map, ask them probing questions to generate information about categories of HRGs available at different hotspots, and note down key information in your field diaries.
6. Ask specifically about mobility patterns of HRGs – within the site, from outside the site, out of the site. Ask who migrates from where, which places they come from, or which place they go to, why do they do so, when do they do so and in what numbers?
7. At the end of the session, note down the date, place, number of respondents (disaggregated by HRGs and non-HRGs) and your mapping team number on the back of the chart paper.
8. Once a number of broad maps are generated from different groups of non-HRGs and *visible* HRGs in the site, develop a composite broad map, compiling information generated through the multiple interactions.

Essential Outcomes

1. Identification of important hotspots (i.e. locations where there is a high concentration of HRGs)
2. Major landmarks of the site so that the hotspots may be easily located
3. Categories of HRGs in the site
4. Inward, outward and within-the-site mobility patterns of HRGs, with reasons and timings

Potential Outcomes

1. While marking landmarks, some services such as government hospitals, popular dispensaries, or NGOs could also be marked on the map
2. Numbers of different categories of HRGs available at each hotspot or in the site in general may also be suggested by respondents. But these would be 'weak' numbers, so there is no need to note them down at this stage. Numbers of HRGs would be more precisely estimated through the next method.

General Guidelines

- Carry a letter of introduction or business card from the organisation so that respondents know you have legitimate business and will have a contact point to find out more about the project. Carry an official letter of introduction to enable HRGs to access secondary sources like government clinics if necessary.
- Pay attention and be sensitive to the privacy and the needs and wishes of respondents, especially HRGs.
- Take care not to put HRGs at risk in any way during this process, nor to make them lose income or future clientele.
- Explain the purpose of the exercise verbally and give the respondent the choice to participate or not.
- All participants must know they are free to walk away or ask for the session to be terminated if they are not comfortable with it.
- Once they choose to participate, make a verbal contract detailing how long they are being asked to participate and covering how the information will be kept confidential and secure. Explain how the information will be used.
- In the case of an HRG respondent, explain what further involvement (s)he could choose to have at a later date.
- Water and fruit may be offered during the session, and mapping teams should carry condoms, lubricant and referral information to give to HRGs as requested.

Estimating Time and Human Resource Required

The size of the team and duration of fieldwork will depend on the size of the site for assessment and on how easy it is to travel around the District. Experience shows that a team of ten HRG members is sufficient to make up a District mapping team in India. Working in pairs, they will need between 10 and 15 working days in the District to develop a sufficiently accurate picture of the key population context to facilitate project design. Multiple teams can be recruited and trained to concurrently assess multiple sites.

Selection, Recruitment and Contracting of HRG Consultants for Broad Mapping Teams

When selecting local HRG consultants as mapping team members, it is important to remember that they are being selected not just to generate information about the key population situation in a particular site. They are also being recruited to help analyse the information, to help design or re-design HIV/STI intervention components for HRGs, to mobilise HRG members for the project and potentially to become part of the project themselves in the future.

HRG members recruited as consultants should therefore:

1. Be representative and true peers[1] of HRG members in the sites being assessed
2. Be known in the site and know the site well
3. Be acceptable and credible to HRG members
4. Be proficient in the language of the site, and familiar with the subcultures of the local HRGs
5. Be motivated to work with their peers on HIV/STI risk reduction
6. Be available to follow the entire Broad Mapping, and preferably the Site Assessment process. This means they need to agree to take time off from their regular occupations for a considerable period of time at a stretch, for which they would be financially compensated.
7. Although literacy is not an absolute requirement for mapping team members, it is useful if at least one in four has good literacy and numeracy skills for recording basic data.

In addition, it is good to recruit HRG members who may have some experience of *facilitating group discussion* with other HRG members, who have *good communication* and *interpersonal skills*, who can demonstrate some *aptitude for analysis*. They should be *willing to listen and learn* and be able to summarise and *represent the views of other people* even when they might not agree with them. Mapping teams should be *well balanced* so that each team mirrors the known diverse HRGs who will be part of the assessment.

Recruitment Procedure

HRG members should be recruited to the mapping teams in a *systematic* and *transparent* way. It is important that HRG members are not chosen just from those who are friendly with NGOs implementing existing TIs. HRG members who do not use services are very important to involve, since it is people like them with which the project will need to work.

HRG members who work on mapping should be contracted and paid as consultants who bring valuable skills to the project that are not available elsewhere. As well as a reasonable fee, their contracts should include provision for out of pocket expenses and travel and support mechanisms to minimise the risk of any harm coming to them through their work. Success or failure of the mapping process can rest on the proper recruitment and contracting of HRG members to the teams.

[1] In this situation, peers are those who accept and respect *or* follow the same sexual or addiction norms and practices of a particular HRG

TRAINING

Overview

Mapping training is intensive and usually residential so that HRGs recruited for the teams can focus on learning how to implement mapping effectively. An initial training of 5 days depending on the existing skills of those recruited is carried out, including a substantial amount of fieldwork. Training sites must be located away from the sites which will be assessed so that the teams can first practice facilitating mapping methods with other HRGs. After the initial training, the teams are given technical and moral support by National HRG consultants (NHRGCs) who already have experience in mapping. Logistical support is provided by the organisation that is conducting the assessment.

Who are to be Trained?

1. The local HRG consultants recruited for mapping
2. Mapping organisation staff who will provide logistics and other support to the HRG mapping teams
3. Mapping organisation staff responsible for data analysis

Objectives of the Training

To provide the HRG mapping teams and mapping organisation staff members with an overview of the mapping study, and equip them with the knowledge and skills required for implementing broad mapping.

Structure

Day	Topics
1	**Understanding HIV/AIDS, sex and sexuality, attitudes and values**<br>n What is HIV /AIDS<br>n Transmission routes<br>n What is STI and its connection to HIV epidemic<br>n Testing procedures<br>n Myths and misconceptions regarding HIV/AIDS<br>n High risk groups and vulnerable groups<br>n Mindsets and attitudes<br>n Gender, Sex and Sexuality<br>n Working with HRGs<br>n Working with sex workers and their clients<br>n Working with MSM and *hijras*<br>n Working with IDUs

Day	Topics
2	**Understanding broad mapping, scope, methodology, tools and techniques, field work process, data collection and documentation**<br>n Mapping methodology<br>n Interview tools for HRGs<br>n Understanding the tools<br>n Team structure<br>n Roles and Responsibilities<br>n Reporting mechanisms<br>n Communication Skills<br>n Talking to strangers<br>n Body language<br>n Interpersonal communication (IPC)<br>n Field Techniques (What actually happens when you go into the field, how methodologies evolve, common problems & positive experiences, confidentiality agreement)<br>n Field Process (entry to exit: how to identify respondents, how to start conversations, self-introduction, how to broach the subject at hand, whether to continue or terminate the conversation)<br>**Mock Session and Understanding Documentation formats**<br>n Mock Exercises (Role Plays, Mock Interviews and Recording)<br>n Documentation formats and how to use them<br>n Planning for pilot field work (Identification of places, grouping, logistical arrangements)
3	**Level 1 field work practice – Understanding the field process, data collection, documentation and data compilation**<br>n Planning of Level 1 field work<br>n L1 field work and data collection<br>n Documentation and data compilation<br>n Preparation of analysis charts and town map<br>n Addressing the problems encountered<br>n Do's and don'ts<br>n Quality control<br>n Planning for the next day
4	**Level 2 field work practice - Understanding the field process, data collection, documentation and data compilation**<br>n Planning of Level 2 field work<br>n L2 field work and data collection

Day	Topics
	n Documentation and data compilation<br>n Preparation of Table C<br>n Addressing the problems encountered<br>n Do's and don'ts<br>n Quality assurance
5	**Accounts and Contracting, field implementation scheduling**<br>n Candidates who are suitable are contracted<br>n Accounting system and methods (Accounts formats, fees, advances deductions, taking care of bills and vouchers, what can be claimed and what can't, daily allowances, attendance, group insurance and roles and responsibilities in settlements)<br>n Logistics (Board and Lodging, Travel, Team Communication, Team assigning for field work)

Training Approach

The participants have differing levels of education and experience. The pedagogy has been developed with this in mind:

a) Information sessions – presentations
b) Discussions – small & large group
c) Exercises – individual and group
d) Role plays (Mock exercises)
e) Field visits
f) Experience sharing

The training package consists of handouts of presentations in local language & interview tools.

Some Caveats

This training will provide participants with basic information on HIV/AIDS, definitions of high risk groups and attitudes and values towards these groups, all of which is relevant to the mapping HRGs. The training is meant only to enhance their skills to implement broad mapping and not to confer any degree of expertise on the topics that are taught.

Due to the wide range of issues and the limited time of five days, the choice of resource persons is critical to the success of the programme. Resource persons need to have proven participatory training skills and experience of mapping HRGs, and good understanding of training issues.

Pre-training Activities

The following steps need to be completed before the training programme can commence:

n **Inform** selected HRG members of training, and provide them with details of time, venue, number of days of stay, lodging and boarding arrangements.

- **Date:** While fixing dates for the training the following should be considered:
  - Since the training will follow shortly after selection in the field, and Broad Mapping implementation will immediately follow the training, the participants should be given adequate time (3-4 days) to wrap up business at their homes
  - Training should ideally not clash with any state holidays and local festivals
- **Venue:** The venue should be a convenient District headquarters or a big town that is centrally located and accessible to all participants. District headquarters or large towns are preferred so that fieldwork practice during training can be easily arranged, without placing too much load on local HRGs. The venue should also be selected with care to ensure that HRG members will be comfortable and respected there, and not discriminated against.
- **Materials:** Materials for the training need to be finalized well in advance and materials like handouts need to be translated into local language. Training kit: flip charts, badges, chart paper, marker pens, white board, T.V, V.C.P. and LCD, etc.

Day 1 Session 1: Welcome and Introduction

Key objective(s):

- To welcome the participants
- To help participants know each other better and get comfortable with the training
- To provide brief introduction about the organisation

Materials required: Postcard size chart papers, Pen/marker to each participant, Whiteboard, Notice board to stick the pictures, sticking tape

Methodology: A short speech, game

Content:

- The welcome should set out the objectives, introduction and overview of 5 days training
- Explain that the training will include classroom, participative sessions as well as a one-day fieldwork.
- The participants should be clearly informed of the need to take the training seriously, informing them that their contract would be signed at the end of the training based on evaluation by trainers.
- Logistics and stay arrangements will also be discussed. Key administrative staff that can be contacted for any kind of support should be introduced.

Facilitator Briefing: The facilitator explains the process, terms and conditions of the game.

Process:

Step 1: Distribute cards and pens to all the participants.

Step 2: Ask the participants to draw a picture on their card which reflects their personality.

Step 3: After drawing, each participant will come to the front and introduce themselves to the large group, while introducing he/she should explain the reason for having drawn their particular picture.
Step 4: Facilitator should collect all the cards and stick them to the board, while sticking the cards makes categories with same type of pictures (e.g. trees, birds, animals, nature)
Step 5: After completing step 4 analyse the different categories of picture and draw a comparison with the mapping study.

Time: 45 Min

Day 1 Session 2: Basics of HIV/AIDS

Key objective:

- To familiarise the participants with the basics of HIV/AIDS, what it is, its transmission and prevention

Materials required: LCD projector, Chart paper, White board marker, Presentation slides

Methodology: Group work, presentation and discussion

Content:

- What is HIV/AIDS
- Transmission routes
- Progression of the disease
- Testing
- Prevention of transmission of HIV
- What are STIs and their connection to HIV epidemic
- High risk groups

Group work process:
The resource person will divide the participants into groups of 4-5 persons. Each group will be given questions on HIV/AIDS to discuss in small groups.
The group is asked to discuss the questions for 10 minutes and write out the discussions point-wise on a chart. Each group then presents their charts and a group discussion follows. At the end of or during the group presentation the resource person briefly goes through all the issues through power point slides, making it a point to clarify all wrong perceptions regarding HIV/AIDS and touching upon issues which may not have come up in the discussions.

- Group 1: what is HIV/AIDS and how does HIV enter the body?
- Group 2: How does HIV spread and how can it be prevented?
- Group 3: What are the signs and symptoms?
- Group 4: Who are most at risk of getting infected?

Time: 1 Hour

Day 1 Session 3: Values and Attitudes and Working with HRGs

Key objective(s)

- To examine how mindsets affect behavior, work and work culture.
- To become aware of mindsets of different people looking at the same issues, specifically concerning HIV/AIDS.

Materials required: Chart papers, markers

Methodology: Games, small group discussion, large group discussion, story telling, group work

Content:
- What is an attitude, how is it formed?
- Positive and negative attitudes, helpful and unhelpful attitudes
- Attitudes and intelligence, emotional and behavioral components
- Perception bias
- Changing attitudes

A) Small group discussion

Step 1: Select 6 volunteers from the participants and request them to wait outside the conference hall until you call them in.

Step 2: Request the remaining participants to form 6 small groups and give instruction to the groups to ignore the volunteers when they try to join the group for discussion.
Facilitator will go out and ask volunteers to join the group for discussions. (Talk to them separately so that volunteers and small groups are unaware of what was told to each party.)

Step 3: Start the process formally. Give signal to volunteers join the group where heated discussion is going on. Listen and observe the process happening between the group and volunteers.

Note down individual and group behaviour to understand attitudes that are expressed, which will flow into the next topic of attitude, thought and behaviour.

Step 4: Facilitate discussion by getting feedback from volunteers about their feelings.

Step 5: Facilitator concludes the exercise by explaining that it is necessary to be cautious about attitudes since they can hurt others' feelings.

B) Large group interaction over social issues

Step 1: Statements on social issues (5 minutes) Facilitator will call out statements on social issues. Participants will note down the same (examples are given in the process summary).

Step 2: Discussion on their attitudes (8 minutes) Discuss about each issue which reflects the attitude of the participants to gauge the attitude prevalence in the society.

Step 3: Conclude/summarise (2 minutes) Facilitator will conclude by explaining that different people have different attitudes, family and society have a very large influence on attitudes.

C) Story telling

Step 1: Story telling (5 minutes) Story about Shankar and Asha will be narrated to the participants in large group.

Step 2: Generating discussion (10 minutes) Facilitator will generate discussion among participants to talk about their feeling and thoughts.

Step 4: Conclude and summarise (3 minutes) Generate discussion on mindset of the family and community towards HIV+ people.

D) Small group discussion on caselets

Step 1: Form small groups and issue a copy of caselet Facilitator needs to make small groups of the participants; issue a copy of the caselets to the group.

Step 2: Open discussion: (20 minutes) Take each caselet and throw it open for discussion for a few minutes. After the discussion project the mindset which is highlighted in the caselet.

Step 3: Conclude/summarise (5 minutes) Close this module by categorising helpful and unhelpful mindsets.

E) Group work to commit the participants to field work

Step 1: Forming small groups Request participants to form 6 small groups. Each group takes chart paper and marker pen.

Step 2: Listing qualities of social worker (5 minutes) Each group should list qualities that are required for social worker.

Step 3: Sticking it on notice board (2 minutes) Ask the participants to stick on the notice board what they have listed.

Step 4: Conclude and summarise (3 minutes) Conclude by saying how helpful these qualities are for us to follow in the field for effective work.

Time: 1 Hour

Day 1 Session 4: Sex and Sexuality, Sexual Terminology

Key objectives:

- To provide an orientation and sensitise participants about the topic of sex and sexuality.
- To generate local terminology for words used in the field.
- To get participants to overcome their inhibitions regarding use of sexuality related terminologies.
- To create awareness of local/slang words so that participants would understand such references from Key Informers.

Materials required: Chart papers, markers, LCD, black/white board. 6 sets of envelopes each containing chits bearing the letters of the alphabet

Methodology: Group interaction, word game, presentation, lecture

Content:

- What is sex?
- Definition of sexuality
- Why should we speak of sexuality?
- Meaning of sexuality
- Sexual Terminology
- Different types of sexual contact
- Risk involved in each of the sexual contacts

Word Game

Step 1: Forming small groups: Form small group of participants and distribute envelopes with chits that have different letters written on them to all the groups.

Step 2: Appointing referee: One of the facilitators (or a participant, in case there is only one facilitator) will become referee and roam about some place slightly away from the training hall to observe the group dynamics.

Step 3: Writing word on the board: The facilitator writes a word on the board relating to sex or terminology that would be used in the study – such as commercial sex worker, condoms, etc. and starts the game formally.

Step 4: Participants should build word: Facilitator will ask the group to paste the corresponding letter onto a sheet, the group which rushes to show it to a referee with maximum words wins.

Step 5: Sharing of experience and presentation: Enter all the terminology on the chart (facilitator to add to the list if important terms are missing) and ask participants feedback and let them share their perception, experience and learning. Initiate further discussion by presentation.

Step 6: Conclude/Summarise: Facilitator will conclude saying how important it is to shed inhibitions, feel comfortable, non-judgmental in using sexual terminology while interviewing HRGs.

Time: 1 Hour

Day 1 Session 5: Sexual Orientations

Key objective:

- To make participants feel comfortable while working with HRG

Methodology: Presentation and discussion

Materials required: Chart paper, markers, LCD

Contents:

- Sexual orientations, sexual behaviors and sexual identities (homosexual, bisexual, heterosexual, gay, lesbian, etc.)
- Various sexuality/gender identities in India (e.g. *kothi*, *panthi*, double decker, *jogappa*, *hijra*, *jogti*)
- How to identify different sexual minorities

Time: 45 Min

Day 1 Session 6: Feedback Session

Key objective(s):

- To get the participants' feedback on the day's sessions
- To understand the training impact
- To clarify any doubts

Methodology: Group exercise

Materials required: Chart paper, marker pen, whiteboard, prepare a checklist

Group exercise:

Step 1: Selecting leaders: Select 2 leaders from participants and ask them to facilitate the session.
Step 2: Recollecting each session: Facilitator will ask the participants to recall each session. If there is any doubt it will be clarified through other participants on the spot.

Time: 30 Min

Day 2 Session 1: Warm-up Exercise and Presentation of 1st Day's Report

Key objectives:

- To energise
- To start the session with continuity from Day 1

Methodology: Game or song, discussion

Time: 30 Minutes

Day 2 Session 2: Introduction to Broad Mapping

Key objectives:

- To provide an overview of mapping study
- To help the participants understand the process and methodology of mapping

Methodology: A classroom session in which the facilitator provides the above information through the lecture method. Participants are encouraged to ask questions regarding the study.

Materials required: Presentation slides, handouts, chart paper, white board and marker pen

Contents:

- What is mapping?
- Purpose of broad mapping
- Scope of broad mapping
- Methodology of broad mapping
- Levels of data collection
- Timeline of the broad mapping
- Team members involved in field work

Time: 1 Hour

Day 2 Session 3: Scope of Information and Levels of Field Work

Key objectives:

- To make the participants understand different levels of field work
- To understand the scope of information at each level
- To understand and internalise the tools used in the study

Methodology: Classroom session in which facilitator provides information through lecture method. Participants are encouraged to ask questions and doubts regarding the study.

Materials required: Chart paper, white board, marker pen, L1 & L2 check lists, documentation formats for practice work, presentation slides, handouts

Contents:

- Scope of information
  - On estimates
  - On mobility
  - On timings of availability
  - On contact information
- Levels of information
  - Tools required collecting L1 and L2 information
  - Different types of stakeholders (Key Informer)
  - Different approaches (Selection of Key Informer)
  - Use of local terminology
  - Main questions and probe questions
  - Criticality of information
  - Definitions of terminologies used in the study

Time: 1 Hour

Day 2 Session 4: Mock Exercises (Key Informant Interview)

Key objectives:

- To understand and internalise the tools used in the study

Methodology: Interactive session

Materials required: L1 checklist, chart paper, and marker pen, white board

Process:

Facilitator explains the checklist question for participants to understand and internalise. Participants are also provided with list of questions (checklist) which they need to practice with local dialect.

Step 1: Facilitator asks participants to sit in pairs (selecting one partner from the group), distribute L1 checklist to all and ask them to go through it.

Step 2: Ask each pair to do mock interview by using the L1 checklist. One person will act as interviewer and the other will act as respondent. Guide the groups to repeat the exercise by changing the role. Facilitator should observe each group to assess the interviewing skills of individual participants and to clarify any doubts.

Step 3: Discussion on the mock exercise

- Ask the groups to share their experiences
  - Learning
  - Difficulty
  - Confusion or lack of clarity

- Facilitator should share own observations
- Explain the process of interview and discuss importance of probing questions

Step 4: Conclude/summarise: Ask the participants to summarise the important learnings of the session

Time: 90 Min

Day 2 Session 5: Field Work Processes

Key objective:

- To explain the process involved in field work

Methodology: Lecture method, brainstorming, demonstration, small exercises.

Materials required: Chart paper, white board, marker pen, presentation slides, handouts. LCD

Contents:

- Planning
- Data collection
- Documentation
- Data compilation
- Validation
- Town wrap-up

Time: 3 Hours

Day 2 Session 6: Feedback Session

Key objective(s):

- To get the participants' feedback on the day's sessions
- To understand the training impact
- To clarify the doubts

Methodology: Group exercise

Materials required: Chart paper, marker pen, whiteboard, prepare a question of checklist

Group exercise:

Step 1: Selecting leaders: Select 2 leaders from participants and ask them to facilitate the session.

Step 2: Recollecting each session: Facilitator will ask the participants to recall each session. If there is any doubt it will be clarified through other participants on the spot.

Time: 30 Min

Day 3 Session 1: Warm-up Exercise and Presentation of 2nd Day's Report

Key objectives:

- To energise
- To start the session with continuity from Day 2

Methodology: Game or song, discussion

Time: 30 Min

Day 3 Session 2: Planning for L1 Field Work

Key objectives:

- To understand the planning process

Methodology: Group work

Materials required: Town map, marker pens, chart paper, white board

Contents:

- Selection of places for conducting interviews
- Pairing the field staff
- Fixing targets
- Establishing communication system

Process:

Divide the participants into 2 groups, give town map to each team and ask them to prepare field plan as explained in the planning session.

Time: 30 Min

Day 3 Session 3: L1 Field Work

Key objectives:

- To get field experience
- To put skills acquired during classroom training to situations on the field
- To understand the skills and attitudes of the field staff
- To build the confidence of the field staff
- Protocols – time, grouping, travel, meeting with FS, return
- To understand field etiquette
- Potential problems and solutions

Methodology: Field visit and conducting interviews with Key Informants

Materials required: L1 formats, transport facility

Contents:

- Selection of places for conducting interviews
- Identifying Key Informants
- Communication skills
- Starting a conversation with an informant
- Approaches in communication
- Attitude and body language
- Coordination with the partner
- Communication with the field supervisor

Process:

Participants are divided into 2 teams, each team selects a team leader who will act as a field supervisor (FS) and prepare the field visit plan as explained in the planning session.

Each field staff is required to conduct at least 5 successful interviews, in the place which has been given to them.

The FS are supposed to follow the teams and from a distance observe them, and take notes on the way they are conducting the interview for feedback later. FS are also required to conduct 3 interviews.

Time: 2 ½ Hours

Day 3 Session 4: Data Compilation (L1 Formats)

Key objectives:

- To familiarise FW's, FS's and Data Collators with the documentation (L2 format)
- To understand the process of data compilation
- To explain the role of FS and DC in data compilation
- To explain the concept of quality check
- To make participants aware of validation of information

Methodology: Group work, brainstorming, presentation

Materials required: L1 formats, transport facility

Contents:

- Documentation of L1 formats
- Quality check
- Preparation of K I Tracking sheet.
- Preparation of Table – A
- Preparation of Table – B
- Preparation of Town Map with Location and spot
- Preparation of Table – C

Process:

Step 1: Complete the documentation of conducted interviews (20 Min)
Step 2: Sharing of field experience from the participants (15 Min)
Step 3: Sharing of FS observations (10 min)
Step 4: Addressing the concerns and field problems (if any) (15Min)
Step 5: Facilitator will **e**xplain the process of preparing Table-A, KI tracking sheet, Town map and Table B (20 Min)
Step 6: Group work to prepare the tables (60 Min)
Step 7: Presentation of tables by all groups (60 Min) & discussion
Step 8: Facilitator will explain the process of preparation of Table – C (10 Min)
Time: 3 ½ Hours

Day 3 Session 5: Feedback Session

Key objective(s):

- To get the participants' feedback on the day's sessions
- To understand the training impact
- To clarify any doubts

Methodology: Group exercise

Materials required: Chart paper, marker pen, Whiteboard, prepare a question of check list

Group exercise:

Step 1: Selecting leaders: Select 2 leaders from participants and ask them to facilitate the session.
Step 2: Recollecting each session: Facilitator will ask the participants to recall each session. If there is any doubt it will be clarified through other participants on the spot.

Time: 30 Min

Day 4 Session 1: Warm-up Exercise and Presentation of 3rd Day's Report

Key objectives:

- To energise
- To start the session with continuity from Day 3

Methodology: Game or song, discussion

Time: 30 Min

Day 4 Session 2: Planning for L2 Field Work

Key objectives:

- To understand the planning process

Methodology: Group work

Materials required: Town map, marker pens, chart paper, white board

Contents:

- Prioritising places for profiling
- Pairing the field staff
- Fixing targets
- Establishing communication system

Process:

Prepare field visit plan with the help of table B and town map

Time: 30 Min

Day 4 Session 3: L2 Field Work

Key objectives:

- To get field experience
- To apply skills acquired during classroom training to situations on the field
- To understand the skills and attitudes of the field staff
- To build the confidence of the field staff

Methodology: Field visit and conducting interviews with Key Informants.

Materials required: L2 formats, transport facility

Contents:

- Identifying Key Informants
- Interviewing primary stake holders

- Network breaking
- Communication skills
- Starting a conversation with an informant
- Approaches in communication
- Attitude and body language
- Coordination with the partner
- Communication with the field supervisor

Process:

- The same teams will continue the field work as were formed during L1 field work
- FS and DC will prepare the field visit plan as explained in the planning session
- Each field staff is required to conduct at least 3 successful interviews (1 primary and 2 secondary), in the place which has been given to them
- The FS follow the teams and, from a distance, observe them and take notes on the way they are conducting the interview for feedback later. FS are also required to conduct 2 interviews (1 primary and 1 secondary).

Time: 2 ½ Hours

Day 4 Session 4: Data Compilation (L2 Formats)

Key objectives:

- To familiarise FWs, FS's and Data Collators with the documentation
- To understand the process of data compilation
- To explain the role of FS and DC in data compilation
- To explain the concept of quality check
- To make participants aware of validation of information

Methodology: Group work, brain storming, presentation

Materials required: L2 formats, transport facility

Contents:

- Documentation of L2 formats
- Quality check
- Preparation of K I Tracking sheet
- Preparation of Table – C
- Cross-checking L2 formats
- Compilation of L2 formats
- Reconciling of L2 estimates
- Spot validation

Process:

Step 1: Sharing of field experience from the participants (20 Min)

Step 2: Facilitator will **e**xplain the documentation of L2 formats (20 Min)

Step 3: Field staff will complete the documentation of conducted interviews (30 Min)

Step 4: Sharing of FS observations (10 Min)

Step 5: Addressing the concerns and field problems (if any) (20 Min)

Step 6: Facilitator will explain the process of preparing Table-A, KI tracking sheet, Town map and Table B (20 Min)

Step 7: Group work to prepare the tables (60 Min)

Step 8: Presentation of tables by all groups (60 Min) & discussion

Step 9: Facilitator will explain the process of preparation of Table – C (10 Min)

Time: 4 Hours

Day 4 Session 5: Feedback Session

Key objective(s):

- To get the participants' feedback on the day's sessions
- To understand the training impact
- To clarify any doubts

Methodology: Group exercise

Materials required: Chart paper, marker pen, whiteboard, prepare a question of checklist

Group exercise:

Step 1: Selecting leaders: Select 2 leaders from participants and ask them to facilitate the session.

Step 2: Recollecting each session: Facilitator will ask the participants to recall each session. If there is any doubt it will be clarified through other participants on the spot.

Time: 30 Min

Day 5 Session 1: Warm-up Exercise and Presentation of 4th Day's Report

Key objectives:

- To energise
- To start the session with continuity from Day 4

Methodology: Game or song, discussion

Time: 30 Min

Day 5 Session 2: Contracting and Accounts

Key objective:

- To explain the terms and conditions of the contract
- To explain the accounting procedures to be used in the field

Methodology: Presentation

Materials required: Contract copies (FS, DC and FW), accounts formats, chart paper, marker pens

Content:

- Terms and conditions of contract
- Responsibilities and hierarchy for field finance management
- Salary structures, Leave allowances and other benefits
- Budgeting for town
- Processes for taking advances and settlement

Time: 60 Min

Day 5 Session 3: Responsibilities of Team Members

Key objectives:

- To clarify the roles and responsibilities of team members
- To form the teams

Methodology: Presentation, discussion, group work

Materials required: marker pens, chart paper, white board

Contents:

- Roles and responsibilities of FS, DC & FWs

Time: 60 Min

Day 5 Session 4: QMS (Quality Assurance Mechanism System)

Key objectives:

- To establish the QMS in field work
- To understand the concept of NC (Non-Conformities)
- Quality assurance

Methodology: Presentation, discussion

Materials required: marker pens, chart paper, white board

Contents:

- Accuracy in listing of locations
- NC at different levels
- Accuracy in estimation of volumes
- Timeliness in completion
- Clarity, comprehensiveness in reporting
- Friendly attitude and acceptable behaviour

Time: 60 Min

Day 5 Session 5: Field Scheduling and Pilot Field Work

Key objectives:

- To finalise the field schedule
- To distribute towns to each team
- To plan pilot field work

Methodology: Presentation, discussion

Materials required: marker pens, chart paper, white board

Contents:

- Overall field scheduling
- Planning for pilot field work
- Set-up activities

Time: 60 Min

ATTACHMENT 1: BASICS OF HIV/AIDS

- **AIDS**
  - **A: ACQUIRED**: Got from outside the body
  - **I: IMMUNO-** : Immune or defense system
  - **D: DEFICIENCY**: Lack
  - **S: SYNDROME**: Group of signs/symptoms
- **HIV = Human Immunodeficiency Virus**
- **HISTORY OF HIV/AIDS**
  - 1981 – First reported case of AIDS in USA
  - 1983 – HIV Virus identified by 2 scientists
  - 1986 – First Indian AIDS case in Chennai
  - 1988 – First AIDS case in Karnataka
- **HOW DOES HIV ENTER THE HUMAN BODY?**
  - **Unprotected** sexual contact – vaginal/anal/oral
  - **Transfusion** of infected blood or blood product
  - **Unsterilised** sharps – needle, syringe, blades, dental/surgical instruments
  - **HIV infected** mother to child
- **TYPES OF PROGRESSORS**
  - **Typical**: 8-10 Years
  - **Rapid**: 6 Months – 2 Years
  - **Slow**: More than 10-12 Years
  - ***Depends on:***
    - Virus: Type of virus, viral load (Amount of virus in the body)
    - Previous immune status
    - Current health and nutrition
- **PRIMARY PREVENTION OF SEXUAL TRANSMISSION OF HIV**
  - Use condoms correctly, consistently
  - Have non-penetrative sex: mutual masturbation, thigh/breast sex, etc., when condom not available
    - Get STIs of self and partners treated promptly and completely
- **STIs AND HIV**
  - Routes of transmission of STIs and HIV are more or less the same! HIV is an STI!
  - STI prevalence - an indication of HIV risk

- **HIGH RISK GROUPS**
  - FSWs
  - MSM
  - *Hijras*
  - IDUs
- **BRIDGE GROUPS**
  - Clients of sex workers
  - Sexual partners of MSM and *hijras*
  - Sexual partners of IDUs
- **NACO CLINICAL CASE DEFINITION OF AIDS**
  - A: Two positive tests of HIV (E/R/S)
  - B: Any one of the following:
    - Significant weight loss (>10% in 1 month)
    - Chronic diarrhea (intermittent/persistent)
    - Prolonged fever (>1 month intermittent/continuous)
- **KILLING HIV**
  - HIV is a very fragile virus
  - At 56 Deg. Centigrade dies within 30 minutes
  - Boiling kills the virus within a few seconds
  - Hypochlorite solution 0.5 to 1%
  - 70% ethanol
  - 2% ether inactivates HIV virus

ATTACHMENT 2: MINDSETS AND ATTITUDES

A) CASELETS: MINDSETS / ATTITUDES AND BEHAVIOURS

Objectives: The main objectives of the following caselets are to:

- Become aware of the perceptions, mindsets, attitudes and behaviours of different people looking at the same issues concerning HIV/AIDS and develop sensitivity to the implications of the different attitudes and behaviors
- Gain some insights on the management of key mindsets/attitudes and behaviours (both in ourselves and others) in order to effectively achieve project objectives
- List a set of helpful work attitudes and behaviours which we need to adopt and practise during HIV/AIDS social research and/or social field work in order to project a professional image and also to produce the desired project results

Caselet 1: During the course of field research you are asking some questions about HIV/AIDS to an elderly couple. The husband shows you the newspaper and replies – "I do not know why there is so much noise about HIV/AIDS these days. In all these years, I am yet to come across a single person who has this problem. I think this is yet another case where the government is trying to create panic, where there is none!" The wife adds – "I think so too, what is HIV/AIDS anyway?"

- What are the reasons for these opinions?
- What are your opinions?
- What should be the opinions and behaviours of the HIV/AIDS project?

Caselet 2: After the day's field work, you are having dinner in a hotel and you hear two people talking at the next table. One says – "Did you know? There is some talk that Chikka has HIV/AIDS." The other person responds – "I am not surprised. I think he asked for it by the way he was behaving!"

- What are the reasons for these opinions?
- What are your opinions?
- What should be the opinions and behaviours of the HIV/AIDS project?

Caselet 3: You are talking to two government servants while on field work and asking questions about the truck halt points and location of commercial sex workers. One says – "I think there is no use blaming the truck drivers for the HIV/AIDS problems, just because they form a high risk group. We need to do something about the commercial sex workers." The other replies – "I know what the police should do. They need to crack down on the commercial sex workers on the highways. That is the only way to stop this problem from this uncontrollable spreading".

- What are the reasons for these opinions?
- What are your opinions?
- What should be the opinions and behaviours of the HIV/AIDS project?

Caselet 4: While you are on field work, you happen to walk into a college where there was a debate on "Drug Abuse and Implications for Students." The main thrust of the group of students who were talking about the dangerous implications of drugs was that – "All drug users are potential HIV/AIDS cases." The main thrust of the opposite group was that – "If you are careful with needles, then you can beat HIV/AIDS."

- What are the reasons for these opinions?
- What are your opinions?
- What should be the opinions and behaviours of the HIV/AIDS project?

Caselet 5: During field research, you happen to meet two legislators and get into a discussion on prostitution in the area . One says – "In the larger interests of the society , we must legalise prostitution and introduce health checks". The other replies – "I do not agree. Legalising prostitution goes against our culture over the centuries. It should be controlled by very strict anti-prostitution laws and close policing."

- What are the reasons for these opinions?
- What are your opinions?
- What should be the opinions and behaviours of the HIV/AIDS project?

Caselet 6: During the afternoon coffee break, you meet a group of students and you ask them some questions about sexual preferences and homosexuality. One replies – "I do not know how men enjoy having sex with men. It is disgusting". Another one says – "The same applies to women also. It is definitely unnatural. Since it is against the law, legal actions should be taken to stop such perversions."

- What are the reasons for these opinions?
- What are your opinions?
- What should be the opinions and behaviours of the HIV/AIDS project?

B) MINDSETS AND ATTITUDES AND WORKING WITH VULNERABLE GROUPS

What is an attitude?

- An internal set of beliefs, opinions about myself, other people, events, things and about life itself
- Predetermined approach
- Predispositions
- Conditioned thinking

How are attitudes formed?

- From birth, as we grow up, we go through different experiences in life
- Past experiences leave us with some information, learning, beliefs, opinions, conclusions and paradigms
- These form a database, get stored in our memory and become our attitudes
- Attitude is an internal function/process
- Over the years, all of us have formed attitudes about many issues

Attitudes: Positive and Negative

- If past life experiences are positive, the attitude is generally positive in those areas
- If these experiences are negative, the attitude may be negative in these areas
- Generally, all of us have a combination of both, depending upon the sum total of our life experiences
- Some may also have more of either one: this is called the predominant or persistent attitude
- This may sometimes override all other rational information or considerations

Helpful or Unhelpful Attitudes

- Apart from positive or negative, we may also call them favourable/helpful or unfavourable/unhelpful attitudes
- Let us also look at these as a continuum:

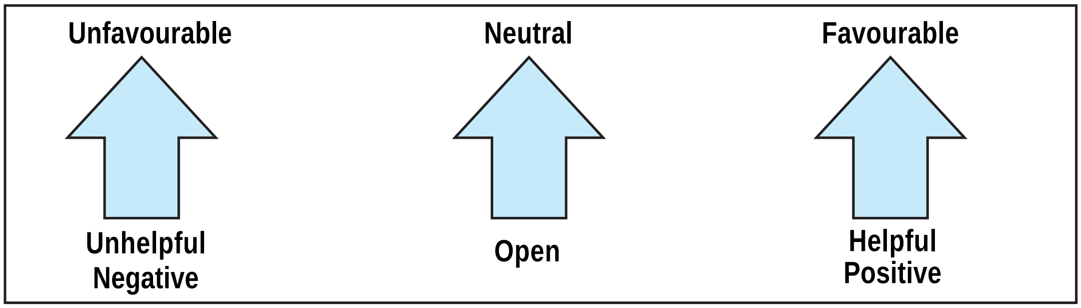

Attitudes and Valence

- Mindsets can be measured by their 'valence'
- Valence is the strength of the psychological attractiveness (for/liking or against/dislike) towards anything
- Positive valence indicates a liking
- Negative valence indicates a dislike
- Middle indicates ambivalence – not sure either way – may be torn between the two

Attitudes drive our behaviour!

- Internal attitudes prepare us for a behavioural response to an external event
- Every time we face a situation, our attitudes guide us to handle that event
- Sometimes, we may blindly follow our attitudes to handle the event
- Some may change their mindsets because of new learning, and some may not!

Attitude – another good definition!

- A persistent tendency to think, feel and behave in a particular way in response to information from the external world
- Every attitude has three components, which get integrated in a dynamic stage:
- Intellectual (Thoughts)
- Emotional (Feeling)
- Behavioural (Action)

Intellectual Component

- Information oriented thinking
- Deals with our stored information, irrespective of whether we are empirically right or not – no validity check
- A Cognitive Process – How we come to know and understand the world, process information, make judgments/decisions, and describe our knowledge/understanding to others
- Cognitive Dissonance – A state of psychological tension in which two or more conflicting thoughts/ attitudes are held simultaneously in our mind
- May lead to an evaluative/judgment approach in interpretation of information

Emotional Component

- Involves the feelings of a person
- May be positive/favourable/liking or negative/unfavourable/dislike or neutral
- A very strong and powerful component
- It is this "emotional loading" which gives mindsets their insistent, stirred up, intense and motivating character
- Could be highly subjective and irrational at times, even when there is valid information to the contrary

Behavioural Component

- Action oriented approach
- Tendencies to behave in a particular way
- How we are going to put our mindset into a dynamic action
- A positive attitude may result in help, support, encouragement or reward
- A negative attitude may result in opposite actions and may also cause harm
- The actions of one may be perceived as rational or irrational by the others depending upon their own mindsets

Interventions for Change

- Transforming attitudes is a continuous process
- Changing our mindsets – Being open to the feelings of a person, new information and developing new perspectives
- Changing mindsets of others
  - Involves the feelings of a person
  - Education, awareness, co-opting
  - Continuous focus on distinction between opinions and biases vs. facts and reality
  - Giving insights on incongruities
  - Personalising the biases to see implications
  - Peer and social pressure
  - Building an open-minded work culture

ATTACHMENT 3: SEX AND SEXUALITY

"When God was not ashamed of creating sex, why should I be afraid of talking about it?"

—Sigmund Freud

Sexuality is an important element of reproductive health and should be an integral aspect of reproductive health care. Yet many health providers are uncomfortable discussing sexuality with clients, may not even perceive the need to do so, or are judgmental about certain sexual behaviors that differ from their own.

In recent years, as providers have been faced with the realities of the HIV epidemic and the critical role of sexual behavior in reducing risk, it has become ever clearer that STIs and HIV cannot be addressed effectively without a frank and direct dialogue about sexuality and sexual practices. Indeed, obtaining information about clients' feelings and attitudes about sexuality forms a core component of assessing need for appropriate health services.

While a frank and sensitive discussion of sexual practices in a nonjudgmental environment can best meet clients' needs, this ideal can be difficult to achieve for many reasons, including:

- **Cultural taboos:** In most cultures, explicit discussions of sexual practices and sexuality are generally taboo, and great stigma surrounds STI/HIV infection. Experience in many settings has indicated, however, that such taboos can be overcome, and when discussed with sensitivity, most clients are willing to talk about such issues and are grateful for the opportunity to discuss their concerns in a safe environment.
- **Discomfort:** Providers often are inhibited or uncomfortable and frequently lack the information that would support them in discussing sexuality and STI/HIV issues with clients.

What is Sex?

- Sex has two distinct functions in our lives - one is how we have babies, called reproduction, and the other function is pleasure and an expression of love, intimacy and affection.

Being sexual can mean:

- Feeling attractive and good about your body
- Feeling emotionally close to someone else
- Enjoying being touched and hugged
- Touching your own body
- Feeling attracted to another person
- Making up romantic stories in your head
- Having sexy thoughts or feelings
- Engaging in sexual acts with another person

Sexuality

Sexuality is complex. It is much more than simply your sexual feelings or having sexual intercourse. Sexuality includes:

- Your awareness and feelings about your own body and other people's bodies
- Your ability and need to be emotionally close to someone else
- Your understanding of what it means to be female or male
- Your feelings of sexual attraction to other people
- Your physical capacity to reproduce

Sexuality is an important, joyful and natural part of being a person.

Definition of Sexuality

- Human sexuality encompasses the sexual knowledge, beliefs, attitudes, values, and behaviours of individuals. It also includes the anatomy, physiology, and the biochemistry of the sexual response system; roles, identity, and personality.

Why should we talk about sexuality?

- Awareness of one's own sexuality
- To respect ourselves and hence respect others
- To feel comfortable
- To be non-judgmental
- To prevent the spread of STIs/HIV
- To acknowledge that sex plays an important role in HIV prevention
- To discuss sex and sexuality issues with clients
- To understand the risks involved
- To talk about safer sex

ATTACHMENT 4: BROAD MAPPING – OVERVIEW

What is mapping?

Mapping is the process used to depict the distribution of specific characteristics over a geographical area

Approaches of broad mapping

Broad approaches:

- Enumerating the risk groups – risk group approach
- Identifying and typifying locales where risk activities occur – geographical approach

Key elements

- Clearly defining risk activities and asking strategic questions
- Staged approach and complete trawling – locations, spots
- Direct contact with stakeholders - secondary and primary
- Direct spot validation and triangulation

Scope of Mapping

- Information will be collected on prevalence of high risk activities (HRA) in towns. HRA refers to:
  - Commercial sex work: This activity is further sub-divided by its typologies and gender
  - Males having sex with males (MSM)
  - Injecting drug users (IDU)
- Geographic location profile
- Estimation of HRG group numbers
- Typology of soliciting (e.g. Street, Lodge, etc.) among female sex workers
- Variations in the HRG volume
  - Weekly
  - Seasonal
- Number of HRG persons
  - Belonging to the same town
  - Coming from outside town

Source of information

Information for the study is gathered from Key Informants (KI):

- Primary stake holders
- Secondary stake holders
- Tertiary stake holders

Methodology

- The study will adopt a Geographical Mapping approach, which has been developed and tested in Karnataka, Kerala and Maharashtra
  - Step 1: 30 to 75 field interviews of KIs of tertiary and secondary stakeholders, to identify places of HRA
  - Step 2: Ranking of places based on HRA, frequency of mention; consolidation
  - Step 3: All the spots with activity of >3 visited and mapping carried out through at least five interviews (3 primary and 2 secondary stakeholders)
  - Step 4: One group discussion with each HRG in the town. The GD will validate information from interviews and also provide information about cross-cutting issues like STI providers, movement of sex workers, timing, etc.
  - Step 5: Compilation, analysis, qualitative reflections

Tools used in the study

A combination of the following tools and methods will be used

- In-depth individual interviews (lead through checklists)
- Group discussions
- Observations

Techniques used in the study

- Clean slate method
- Snowballing technique
- Reaching saturation point

Terms used in the study

- KI (Key Informant)
- Primary Stake holder
- Secondary Stake holder
- Tertiary Stake holder
- Location
- Spot
- HRA: HRG,MSM,IDU,ESW,OHRA
- Estimates

Typology of places where sex work is solicited

- Street-based: SSW
- Brothel-based: BSW
- Home-based: HSW
- *Dhaba*-based: DSW
- Lodge/Hotel-based: LHSW
- *Devadasi*: DEV
- *Jogati/Jogamma*: JGM
- *Jogappa*: JGP

ATTACHMENT 5: LEVELS OF FIELD WORK

Information for the study is collected at two levels

Level 1

- First day of the FW is called Level 1; during this stage the field team will use the "clean slate method" to reach out to each part of the town to collect information about the places
- FW during L1 continuous until it reaches "saturation point"

Saturation point: In L1 stage of FW the team will get a lot of new places. As the FW progresses, the number of new places will come down. At a certain point the number of new places will stagnate. This point is called the "saturation point".

- **Protocol for deciding saturation**
  - Prepare 1st list of places by compiling the KI interviews carried out during the morning session
  - Prepare 2nd list of places by compiling the KI interviews carried out during afternoon session
  - Comparing the two lists will provide the following results

Results	What it means	Further action
Same places are mentioned	Saturation is reached	Move to L2 FW
1 to 5 new places are mentioned	Saturation is reached	Move to L2 FW
6 to 20 new places are mentioned	Not reached saturation	-Retain some teams to continue L1 till saturation reached<br>-Assign remaining teams for L2 FW
21 to 30 new places	Not reached saturation	Continue L1 FW till it reaches SP
31 to 40 new places	L1 FW is not complete	Continue L1 FW till it reaches SP

Level 1 Information requirements

- Names of places in the town where HRA, i.e. commercial sex work, MSM and IDU activity is reported
- Estimates of number of participants engaging in HRA at each place in the town

Some tips:

- Mention specific name of the place (place where the sex workers/MSM/IDU stand to solicit or perform the risk activity), not just the area
- Don't consider interviews where the KI gives only 1 or 2 place names
- In places like bus stands, railway stations and cinema halls, there is a chance of getting several places (e.g. toilets, entrance, auto stand, near ticket counter, etc.). Consider all the places separately as mentioned by the KI
- Take clear address of each place to avoid confusion and duplication while doing compilation

- L1 information will be solicited from all three types of KI, namely, primary, secondary and tertiary stakeholders
- Use L1 checklist for getting the information
- The information will be entered in L1 format

Level 2 Information requirements

- Spot name
- HRA type – typology and gender
- HRA estimates – typology and gender
- Where participants are coming from (same town or outside town)
- High-volume and low-volume days (weekly increase and decrease)
- Fluctuations in number of participants in HRA

HRA Typology

SN	HRA	Typology	Gender
1	Female sex worker	SSW (Street-based sex worker)	F
		BSW (Brothel-based sex worker)	F
		LHSW (Lodge/Hotel-based sex worker)	F
		HSW (Home-based sex worker)	F
		DSW (*Dhaba*-based sex worker)	F
2	Male having sex with Male (MSM)		M
3	*Hijra* sex worker (ESW)		E
4	Injecting Drug Users (IDUs)		M/F

ATTACHMENT 6: CODE SHEET, L1 CHECKLIST

CODE SHEET

Gender	
Male	M
Female	F
Hijra	E

KI Profession	
Driver(Auto/Taxi/Lorry, etc.)	DRV
Petty Shop owner	PSO
Brothel owner	BRO
Lodge/*Dhaba* owner	LDO
Watchman	WTC
Police	POL
Pimp	PMP
Madam/*gharwali*	MDM
Sex worker	SXW
Drug peddler	PED
Employer/contractor	EMP
Vendor	VED
Clint	CLI
Network Operator	NOW
Other	OTH

High Risk Activity (HRA)	
Female sex work	HRG
Men having sex with man	MSM
Hijra sex work	ESW
Injecting drug user	IDU
Other HRA Groups	OHRA

KI Type	
Primary	PRI
Secondary	SEC
Tertiary	TER

HRA Typology	
Brothel-based SW	BSW
Street-based SW	SSW
Lodge/Hotel-based SW	LHSW
Home-based SW	HSW
Other type of SW	OTSW
Traditional Risk Groups	
Devdasi	DEV
Jogati/Jogamma	JGM
Jogappa	JGP

HRA Gender	
Male	M
Female	F
Hijra	E

STI Service Provider	
Private Doctor	PVT
Govt. Doctor	GVT
Pharmacy	PCY
Self-medication	SMD
Not treated	NTD
Traditional Healers	TRH

L1 - CHECKLIST

	**Female Sex Workers**
1	Do female sex workers operate in this place?
2	What do they do here – soliciting, having sex or both?
3	Where do the female sex workers perform sexual activity?
4	In a day, on an average, how many female sex workers of this type work here?
5	How many of them belong to this town?
6	How many of them come from outside?
7	Which day of the week is the number of sex workers very high? Why do you say so? What is the volume?
8	Which day of the week is the number of sex workers very low? Why do you say so? What is the volume?
9	In a year, when does the volume of sex workers in this place increase? (Probe for event, time and increasing volume)
10	In a year, when does the volume of sex workers in this place decrease? (Probe for event, time and decreasing volume)
	**Men who have Sex with Men (MSM)**
1	Do men who have sex with men come to this place?
2	What do they do here - soliciting other MSM, having sex or both?
3	Where do the MSMs perform sexual activity?
4	In a day, on an average, how many MSM operate here?
5	How many of them belong to this town?
6	How many of them come from outside?
7	Which day of the week is the number of MSM very high? Why do you say so? What is the volume?
8	Which day of the week is the number of MSM very low ? Why do you say so? What is the volume?
9	In a year, when does the volume of MSM in this place increase? (Probe for event, time and increasing volume)
10	In a year, when does the volume of MSM in this place decrease? (Probe for event, time and decreasing volume)
	***Hijra* sex workers**
1	Do *Hijra* sex workers operate in this place?
2	What do they do here – soliciting, having sex or both?
3	Where do the *Hijra* sex workers perform sexual activity?
4	In a day, on an average, how many *Hijra* sex workers of this type operate here?
5	How many of them belong to this town?
6	How many of them come from outside?
7	Which day of the week is the number of *Hijra* sex workers very high?
8	Which day of the week is the number of *Hijra* sex workers very low? Why do you say so? What is the volume?
9	In a year, when does the volume of *Hijra* sex workers in this place increase? (Probe for event, time and increasing volume)

10	In a year, when does the volume of *Hijra* sex workers in this place decrease? (Probe for event, time and decreasing volume)
	**Injecting drug users (IDUs)**
1	Do Injecting Drug Users come to this place?
2	What do they do here – buying or injecting drugs?
3	In a day, on an average, how many injecting drug users inject drugs in this place?
4	How many of them belong to this town?
5	How many of them come from outside?
6	Which day of the week is the number of IDUs very high?
7	Which day of the week is the number of Injecting Drug Users very low? Why do you say so? What is the volume?
8	In a year, when does the volume of Injecting Drug Users in this place increase? (Probe for event, time and increasing volume)
9	In a year, when does the volume of Injecting Drug Users in this place decrease? (Probe for event, time and decreasing volume)
	**Other HR Groups**
1	Do you know any other groups who are at the risk of getting HIV/AIDS?
2	What do they do here?
3	In a day, on an average, how many of such type come to this place?
4	How many of them belong to this town?
5	How many of them come from outside?
6	Which day of the week is the number of such group very high? Why do you say so? What is the volume?
7	Which day of the week is the number of such group very low? Why do you say so? What is the volume?
8	In a year, when does the volume of such increase in this place? (Probe for event, time and increasing volume)
9	In a year, when does the volume of such group decreases in this place? (Probe for event, time and decreasing volume)
	**Common questions**
1	If somebody has an STI, what do they do?
2	Where do they go for treatment?
2	What type of doctor treating STIs available here?
3	Could you give me the contact details of the doctor?

ATTACHMENT 7: FIELD WORK PROCESS

FIELD WORK - PROCESS

A. Planning
B. Data collection
C. Documentation
D. Data compilation
E. Validation
F. Town wrap-up

A. PLANNING

Field work planning is done in 3 stages

- Morning meeting
- Mid-day meeting
- Evening meeting

Morning meeting

Purpose

- Identifying places for visit
- Pairing of field staff
- Allocating places to the pair
- Briefing the team about the level of information needed to be collected
- Preparation of Key Informant list
- Assigning daily targets
- Fixing place and time for mid-day review
- List key tasks for the day
- Disbursing allowance

Identifying places to visit

Day 1:

- Divide the town map into four parts
- Decide how many researchers will visit each part, depending on:
  - Density of population
  - Presence of arterial roads
  - Presence of slums

Identify and list the following:

Day 2 on wards: Places to profile are taken from Table B

- Pairing of field staff for a combination of skills
  - Some may be very good at starting a conversation with sex workers

- Some strike rapport with transgender persons easily
- Another is good at opening up networks

- It is critical for the FS to constantly evaluate, identify and recognise these skills in order to pair FWs effectively
- Each pair of FWs must have at least 1 FW with a recognised skill that is relevant to the place being visited
- This pairing of FWs will change each day, depending on field requirements
- No 2 female FWs will be paired together
- Each female FW must be paired with a male FW

Pairing of field staff

- Each pair is given a specific place to cover:

Places	Sources of information
- 3-4 main movie halls	- Local news paper/hotel staff
- Railway station	- Hotel staff
- 1-2 Main bus stand	- Hotel staff
- Main market area	- Hotel staff
- 3 biggest colleges (including engineering and medical colleges)	- Hotel staff
- 3-4 biggest slums	- Auto driver / hotel staff
- Area with maximum bars	- Auto driver / hotel staff
- Main police station	- Hotel staff
- NGO working on HIV/AIDS	- Secondary data from head office

- No interviews will be conducted by the pair together
- The pair of FWs will always be within sight of each other on the field, leave for the field and report to FS together

Appraising FWs

- While assigning a place to a FW pair, FS should provide instructions on
  - level and type of information to be collected for that place
  - The reasons why that specific place has been selected for visiting

On Day 1

- The instructions are likely to be general in nature, as there is no preliminary information regarding the town

Day 2 onwards

- Frequency of mention of the during the previous day
- HRAs reported in the place

- Estimates of participants in each HRA in the place
- List of Contact Persons generated for the place during the previous day

Preparation of KI list

- Each pair of FW must prepare a KI list depending on the type of place allotted to them and field work timing:
  - The list should have good proportion of K I type and gender
  - FS and DC should help the FWs in the initial period
  - FS should track each FW on the basis of the list

Mid-Day Meeting

Purpose

- To take stock of the fieldwork in the first half of the day and address problems, modify strategies, if required
- Meeting conducted by: Each FS with his respective team members in the predetermined place at the field or at the hotel
- To check the saturation point (during L1 stage)
- To check the field process and progress and take necessary actions
- To complete the documentation of completed interviews
- To understand and address field level problems (if any)
- To change the pairs (if needed)
- To understand the emerging trends
- To fix time for evening meeting

Evening Meeting

Purpose

- To review the day's fieldwork and identify locations for the coming day
- Sharing the field experiences
- Review of the day (by FS)
- Whether targets have been completed
- Completing the documentation
- Preparation of tables A, B, C,
- Qualitative analysis of the data
- Planning for the next day
- Fix time for next days morning meeting
- Review of what the problems were and how they were overcome

B. DATA COLLECTION

Data collection involves 3 issues

- Communication
- Identifying KIs
- Dealing with KIs

Communication:

Communication on the field is largely interpersonal

- Starting conversations with strangers
- Asking questions
- Listening
- Attitudes and body language

Starting conversation with strangers

- Greet the informant with a sincere smile
- Introduce yourself clearly, with a sense of pride and confidence
- Keep the introduction short
- Identify whether the respondent has a few minutes to spare
- Start with general questions and gradually move to the subject
- Speak about an overall health survey rather than HIV
- Make informant understand that his/her personal identity will be kept confidential
- Use a friendly, open, accessible and interested approach

Asking Questions

- Know your questions
- Do not give information
- Be non-technical and relevant
- Use non-formal, commonly used terms and local dialects
- Be gender- and age-sensitive
- Ensure that questions related to all HRAs are asked
- Ask probe questions
- Try to repeat questions eliciting crucial information
- Stay focused
- Do not insist that the informant answers questions which he/she seems to hesitate to answer
- Do not ask very personal and embarrassing questions

Listening

- Ensure that the informant does most of the talking
- Encourage the respondent with proper non-verbal communication
- Don’t interrupt the respondent

- Be attentive to the words the informant is using to talk about sensitive issues
  - Helps for better documentation
  - Helps to understand the body language, the non-verbal messages

Attitude and body language

- FWs body language
  - Be friendly
  - Be confident
  - Be interested
  - Be relaxed
  - Be patient
  - Look directly at the respondent while talking
  - Don't judge
- Respondent's body language – read your informant
  - Is the informant friendly, interested?
  - Is the informant distracted/hesitant?
  - Is the informant too casual or "shifty"?
  - Is the informant hostile?

Approaches in communication

- Positive approach
- Traditional morality approach
- Denial approach
- Fear approach

Identifying KI:

- Seek known contacts in a place
- Approach people based on occupations
- Use members of a channel to contact other people
- Generate lists of contact persons through "Snowballing"
- When you go to places like lodges, hostels or brothels
  - DO NOT ENTER unless you have some contact person inside
  - Stand around the place and start conversation with people hanging around the spot
  - Attempt to interview at least 1 person entering/exiting the place, but not exactly at the entrance

Strategy for identifying good KI

The "Observe, Confirm, Act" strategy

- **Observe** – Before selecting the KI spend some time in the place, move around the area or sit in an inconspicuous place and observe the people and activities to identify potential KIs.

- **Confirm –** Observe these few potential KIs in greater detail. Based on factors such the work they do, how they are talking to people or how busy they are, decide on the KIs who could be approached.
- **Act –** Approach the KI and begin a conversation. It is critical to choose a time and place where the respondent is undisturbed so that, s/he can concentrate on your questions and can also speak without fear of being overheard.

Common problems

- Lack of confidence in FW
- Respondents refuse to speak
- Respondents get annoyed if pushed too much
- People may be suspicious
- May demand something return for sharing the information
- They may not give you much time

Some tips

- Informants should be chosen with care:
  - Different places will have different types of KI who are more useful than others.
- Select the time and venue of your interaction based on their convenience, not yours.
  - For example, do not try and get interviews with sex workers during their business hours.
  - If it is convenient to the informant to meet at night you may have to fix meetings accordingly. Remember: On the field there are no "9-5" office hours.
- Do not waste your time talking to a person who is completely intoxicated and seems out of control
- If you feel uncomfortable, make sure you have a teammate with you, within sight

Interviewing KI:

- Don't use the checklist while asking questions
- Don't write down the responses in front of the respondent
- Do not give false assurances
- No money/alcohol/other rewards may be provided in exchange for information
- Some informants ask for information on STIs/HIV/AIDS
  - Refer to VCTC or hospital if a respondent complains of STI symptoms or any discomfort
- Do not provide medical information yourself

Dealing with KI

- Some informants express interest in participating in the study with the team
  - Take him/her to the FS
  - Don't negotiate about money
  - Confirm whether he/she has some links with the sex work/IDU network; otherwise he/she is not an asset

- Informants might want to talk at length in a private place, ask to meet with the rest of the team, etc.
  - Introduce such person to your FS if he/she insists on meeting with the team
  - Do not bring anyone back to your hotel unless absolutely necessary
  - If any one must meet with you in your hotel, make sure it is in a common area like the lobby

Tips on team work in the field

- Work in pairs
- Do not lose sight of each other at any point
- Help each other in selecting Key Informants
- If you get HRA information, contact person, or name of other place, give it to the concerned FW
- Avoid standing in large groups
- If you cross other team mates on the field, act as if you don't know each other, unless absolutely necessary
- Do not disturb others while they are working
- If you have a message to pass, make signals from a distance; never interrupt an interview
- Keep in touch with the FS (over phone)
- Share all the information in the team meetings

C. DOCUMENTATION

- L1 Format: City/town HRA Information
- L 2 Format: Place Profile Information Sheet
  - All sheets should be filled in legibly and correctly
  - All sheets should have the date, FW ID, district/town/place name
  - Depending on the type of information sought, entries in the cell could either be:
    - A tick mark
    - A number
    - A code
    - Words/Sentences (names, contact details, observations, etc.)

How to fill in L1 format

No.	Term	What it refers to	Type of entry	Filled by	Criticality of information
1.	Date	The date on which the interview is taking place	Date: date/month/ year, e.g., 05/03/07	FW	Yes
2.	FW ID	The ID of the person conducting the interview	ID number	FW	Yes
3.	Location Name	Name of the location as given by the DC	Location Name	FW/DC should check	Yes

No.	Term	What it refers to	Type of entry	Filled by	Criticality of information
4.	Spot Name	Name of the spot where the interview has been conducted	Name	FW	Yes
5.	KI Name and address	Name of Key Informer: Contact details of the KI– where he may be found for further information gathering	Name: Contact Details/Blank	FW	Yes
6.	KI Type	Type of KI based on profession	Code: PRI/SEC/TER	FW	Yes
7.	KI Gender	Gender of KI	Code: M/F/E	FW	Yes
8.	KI Profession	Profession of KI	Code/Blank	FW	If available
9.	HRA Typology	The typology of HRA reported in the place being profiled, as listed in the Codes list – HRA Types.	Code: DEV/JGM/ JGP/BSW/HSW/ LHSW/DSW/SSW/ ESW/IDU/MSM	FW	Yes
10.	Gender	Gender of the participant in HRA	Code: M/F/E	FW	Yes
11.	Estimated Minimum	The lower number of the range of estimated participants in HRA at that place as given by the KI	Number	FW	Yes
12.	Estimated Maximum	The upper number of the range of estimated participants in HRA at that place as given by the KI.	Number	FW	Yes
13.	How many of them come from inside town	Number of HRA persons coming from same town (Min and Max)	Number (Min and Max)	FW	Yes
14.	How many of them come from outside town	Number of HRA persons coming from out side town (Min & Max)	Number (Min and Max)	FW	Yes
15.	High-volume day	Name of the day when the volume of HRA persons will be very high	Name of the day	FW	Yes
16.	Estimates	Number of HRA persons Min and Max	Number	FW	Yes
17.	Low-volume day	Name of the day when the volume of HRA persons will be very low	Name of the day	FW	Yes
18.	Estimates	Number of HRA persons Min and Max	Number	FW	Yes

No.	Term	What it refers to	Type of entry	Filled by	Criticality of information
19.	Increase event	Name of the event in which number of participants in HRA in the place increase (More than the normal estimates)	Name of the event	FW	Yes
20.	When	The time period when the event takes place (Month)	Name of the month	FW	Yes
21.	Increased volume	Increased number (Min and Max)-it should be more than the normal estimates	Number	FW	Yes
22.	Decreased event	Name of the event in which number of participants in HRA in the place decrease (Lower than the normal estimates)	Name of the event	FW	Yes
23.	When	The time period when the event takes place (Month)	Name of the month	FW	Yes
24.	Increased volume	Decreased number (Min and Max)– it should be less than the normal estimates	Number	FW	Yes
25.<br>26.<br>27.	NTD<br>SMD<br>PCY	Tick if they say Not treated<br>Tick if they say Self Medicated<br>Tick if they say take treatment from medical shops	Tick Mark<br>Tick Mark<br>Tick Mark	FW<br>FW<br>FW	Yes<br>Yes<br>Yes
28.	TRH	Tick if they say they take treatment from traditional healers	Tick Mark	FW	Yes
29.	DOC	Tick If they say they take treatment from qualified doctors	Tick Mark	FW	Yes
30.	SN	Serial Number (start from 1)	Number	FW	Yes
31.	Name and address	Name and address of the mentioned doctor	Name and address	FW	Yes
32.	Gender	Gender of the doctor	Code(M/F)	FW	Yes
33.	Type	Type of the doctor (Qualified/ Unqualified)	Code	FW	Yes
34.	Note	Qualitative information about the spot	Text information only	FW	Important
35.	FS Signature	Signature of the Field Supervisor	Signature	FS	Yes
36.	DC Signature	Signature of the Data Collator	Signature	DC	Yes

Some pointers

- All level 2 interviews must be documented, even if the KI reports that there is no HRA in the place
- The total number of L2 formats for a place, including those from KIs who reported no HRAs, should not be less than 5
- Weekly market details need to written at the top of the sheet. Please do not ignore this space
- Each HRA is entered in a separate column. For example, SSW will be recorded in 1 column, BSW in another and IDU in the third
- The gender of the participants in HRA will be listed below the HRA typology
- Each gender will be entered in a new column, even for the same HRA typology. For example, if a place has both male and female SSWs, then in one cell on the HRA typology row write SSW and in the cell below it in the gender row put 'F'. In the adjoining column, in the typology row write SSW and in the cell below it write 'M', and follow these with relevant details in each of the rows below
    - *Jogappas* will be recorded only as eunuchs, not males
    - Care should be taken in recording eunuchs. Not all eunuchs are sex workers
- Estimates will be reported in a range, for example, 35-40. The lower figure, 35 in this case, will be written in the "Minimum" cell, the upper figure, 40 in this case, will be written in the "Maximum" cell
    - In case an absolute number is reported instead of a range, the same number will be entered as both, maximum and minimum
    - While noting estimates of *devdasis*, only those involved in sex work should be recorded. Others may be mentioned in the notes. Sex work includes occasional sex with multiple partners or annual rotating partnerships with single partners
- Details of fluctuations must have three pieces of information:
    - The event around which the fluctuation takes place
    - The time in terms of month, week of the month, day of the week
    - Increased volume

Protocol for documentation

- FWs will not show any documentation sheets in the presence of the KI
- In case the KI is providing too much information to be memorised in the conversation, the FW will take the permission of the KI and write the information down on his/her note pad
- Interviews conducted in the morning will be documented at lunchtime or even between interviews, if the time and space is available. It is suggested that documentation should be done after every 2 interviews so that information is duly recorded before it is forgotten.
- No documentation will be done in crowded places in view of the public

- Documentation will be submitted to the DCs as and when it is completed by each FW. If the FW is in the field, it will be submitted to the FS.
- All documentation on each day will be completed and handed over to the DC on that day itself. NO documentation will be carried forward to the next day.
- In case 2 FWs conduct an interview together, it is imperative that only one documentation format be filled in, rather than 2.
- Each documentation sheet MUST have the IDs of the FW, FS and DC
- The DC must ensure that all information has been duly filled in

D. FIELD WORK REVIEW AND DATA COMPILATION

The purpose of data compilation is:

- Timely identification and correction of errors in reporting, data inconsistency and gaps in data
- Processing of the data for planning fieldwork on the subsequent day
- Entering information into pre-defined formats to send for data entry
- This sheet will include Key Informant information on all days of field work, including Level 1 and Level 2 interviews

KI Tracking sheet

Name of the Town _______________ Name of the District _______________

Field work Stage	Key Informant Type	Male	Female	Eunuch	Total
L1	Primary				
	Secondary				
	Tertiary				
L1 Total					
L2	Primary				
	Secondary				
	Tertiary				
L2 Total					
TOTAL(L1+L2)					

Characteristics	Level 1	Level 2	Total
Profession			
Sex worker			
MSM			
Hijra			
Lodge or dhaba owner			
Madam or *gharwali*			
Pimp			
Driver (auto, taxi, lorry, etc.)			
Vendor			
Petty Shop Owner			
Drug peddler			
Watchman			
Police			
Student			
Hamali			
Coolie/Farmer			
Client			
Employer/contractor			
Others			
**Total Interviews**			

- The purpose of this sheet is to find out whether the appropriate or all types of KIs have been covered for each place visited
- This will also ensure that coverage is not restricted to only 2-3 types of KIs, in which case the information is likely to be skewed
- The primary responsibility for maintaining this table lies with the DC
- This table needs to be updated every day
- FS would review this table daily before dispersing the team to field, it helps to ensure the coverage of all types of Key Informants
- The number of people in each category being interviewed will be entered in pencil, so that it can be updated and changed everyday
- A fresh sheet will be started for every town
- During the discussion on methodological review at the time of wrap-up, this sheet should be referred to in order to reflect on any new learnings about KI types
- This sheet should also be referred to during the wrap-up session in the town in order to reflect upon the type of KIs the team is more comfortable working with.

Collation of HRA info

- Preparation of Table A on Place Details
- Preparation of a Town Map
- Preparation of Table B on Locations - spot Details
- Preparation of Table C – Place Profile List

Table A

The DCs will prepare chart papers with the following table

Name of the town: _______________ Name of the District:____________

Dates of data collection: ______________________

S N	Place Name	Frequency of Mention	Estimates				
			HRG	MSW	ESW	IDU	MSM

Some pointers

- Table A is prepared to consolidate the places reported in L1 interviews
- DC and FS should prepare this table with the participation of all FWs
- Table A will be updated everyday, until the last day in the town
- The place name that appears more than once will be entered only once
- Each time a new place appears on Table A, the team should discuss it and ensure that the place does not already exist under another name on the table
- The frequency of mention will be cumulative of all formats for all days
- Estimates will be updated everyday – they are the averages on Day 1 and Day 2.
- The DC should check all sheets for errors in reporting such names
- Each of the places, the larger as well as the smaller place, need to be profiled and their estimates gathered separately

Town Map

- A local person will be invited to join the FS, DC for preparing a map
- The map provides clarity among the team with respect to locations and spots
- This exercise ensures that double counting does not occur
- In case no printed map is not available, prepare rough map
- The cluster of places are marked with closed boundary and named as a location
- Give important and identifiable names to the locations; e.g. markets, gardens, railway station/bus terminals and their locales, etc.

- Some places will not come under any cluster; these places are called isolated places
- The emerging map could have clusters of places located inside boundaries, as well as isolated places
- Ensure that the original chart showing HRAs in each place is not shown to the local person, in the interest of confidentiality

Table B: Location - Spot Details

SN	Location name	Spot Name	Contact Persons

Some pointers

- Table B is prepared based on the town map, it will have location and spot names
- All places appearing in Table A should be reflected in this table
- Isolated places will be listed last in the table
- Leave some space between two locations to accommodate any new spot that may emerge
- The table should be prepared by DC and FS
- This table will help the team to understand the size of the location

Table C

Name of the town: ___________Name of the district:________________

SN	Location Name	Spot Name	Number of Interviews			Final Estimates (HRA-wise)
			PRI	SEC	Total	

Some pointers

- Table C is prepared to identify places for visit in the level 2 field work.
- Table C will be prepared with 4 criteria:
  - Places with highest frequency of mention
  - Places with highest estimates of HRA
  - Places with higher numbers of HRA
  - Places with rare HRA
- This table will be prepared by the DCs and FS with the assistance of FWs
- This table will be basis for deciding the number of spots in the town and the volume of town HRA, hence must be done with adequate care

- Even interviews that report no HRA in that place should be entered in this table, with estimates showing 0
- This table should reflect 5 interviews in each place at the end of field work in a town
- All estimates should be reconciled, agreed upon, finalised before being put on this table
- FS should validate at least 25% of the spots listed on this table

Guidelines for reconciling estimates

- Address the problem on the spot, by asking the KI to explain the difference between the estimates
- If the estimate, vary by more than 10 persons:
  - Give preference to the estimates provided by primary stake holders and network operators
  - Count the number of estimates in the similar range – the estimates reported by a majority of KIs receive preference over others
  - Arrive at an estimate based on the FS and FWs' experiences on the field
  - If there is still some doubt, the FS must visit the place and arrive at a reasonable estimate in agreement with the team
  - Take average of min and average of max separately
- If the estimates vary by less than 10 persons
  - Consider the lowest and the highest mentioned estimates. **Example:** If KI 1 says there are 5-10 HRGs and KI 2 gives an estimate of 12-15, take the final estimate as 5 – 15
- Revisit the L2 interviews
  - If all 5 interviews show exact estimates
  - If all 5 KIs were from the same group
  - If the spot shows nil HRA which has maximum estimates in L1
  - If some KIs report the prevalence of HRA at a particular place and others report that the HRA does not exist

Guidelines for review of documentation

- While reviewing L1/L2 formats the DC should ascertain that:
  - All the formats are complete and correct with all required signatures and codes
  - The required number of interviews have conducted by each FW
  - The required type of KIs contacted by each FW
  - Estimates are being taken for places in the town
  - Information on all the HRAs is being asked
  - Different names are not being used for the same place

Some pointers

- All places that appear on Table C need to be profiled
- Each spot must be profiled by at least 5 interviews

- DC should provide the following information with respect to the place:
  - HRAs reported in the place
  - Frequency of mention in Table A
  - Estimates in Table A
  - Contact persons' list if any

E. VALIDATION OF INFORMATION

- **At least 5** interviews have to be conducted in/around each place – 2 must be PRI
- Reconcile the estimates, if five interviews widely vary
- FS must validate at least 25% of the profiled spots
  - FS should conduct at least 2 interviews in order to validate it
  - These interviews must be conducted with PRI
  - The findings from these interviews must be recorded on L2 formats
- The report of validation of the place must be documented in the Validation format by FS

Validation format

District__________________ Town _________________

Place _____________________Frequency of mention _________________

	HRA reported (tick)	HRA validated (Y/N)	Reported estimates	Validated estimates
HRG				
ESW				
MSW				
MSM				
IDU				
DEV				
JGP				

Date___________ FS ID_____________ FS signature_______________

Note: In case the FS estimates contradict those of the FWs by more than 50%, the final estimate will be arrived by looking at the profile of KIs, and following the exercise for reconciling estimates

F. TOWN WRAP-UP PROCESS

- Packing L1 & L2 formats
  - Check all the formats for clarity and correctness
  - Arrange them in order

- Update all tables:
  - Table A
  - Town Map
  - Table B
  - Table C
- Prepare qualitative report

QUALITATIVE REPORT

Qualitative learnings are those that cannot be captured on the documentation formats. The supervisor needs to write up a report highlighting the following, in bullet points:

- What is the total estimate of each typology of HRA in the town?
- What are the main factors that promote practice of HRA in this town?
- If any typology of sex work exists in much larger numbers than others, why is it so?
- What are the unique and special features of HRA in this town?
- Are there any stories that you heard about certain places (with regard to HRA) that led to or went against the HRA?
- Is there any traditional or religious places or commercial places where large numbers of HRGs congregate?
- Are there any large settlement camps around the town?
- Are there any social groups that try to control or encourage HRGs?
- Is there any information on inter-state/town movement of participants in HRA?
- Did you hear about any extraordinary, shocking or funny events with respect to HRA in the town?
- What are the main types of places where HRA takes place?
- What are the main places where HRA takes place?
- Are there any places where estimates were not validated but the team perceives the existence of HRA?
- If yes, which are these places, and why could the estimates not be validated here?
- What difficulties were faced in data collection in this town?
- What types of KIs provided maximum useful information in this town? Why?
- Did you hear of any NGOs or social workers' names in this town? If yes, name and contact details?
- The team should refer to the 'Notes' box on their L2 formats for qualitative findings.
- This qualitative report is critical as it will capture the essence and spirit of the town better than all the documentation formats, and hence should be prepared in detail
- The report should be named **Qualitative Report for Town X (name of the town)**.

ATTACHMENT 8: ROLES & RESPONSIBILITIES

ROLES AND RESPONSIBILITIES OF FS

Field Supervisor is the overall in charge of the fieldwork his/her main role is to supervise the field staff, plan the fieldwork according to the situation and complete the fieldwork as per the plan. Responsibilities include:

1. Taking care of set-up process
2. Administration of field work, include financial management
3. Capacity building
4. Ensuring field discipline
5. Identifying places to visit for level 1 KI interviews
6. Pairing of field teams
7. Allocation of places to team
8. Building clarity across the team on the information to be collected
9. Planning schedule for the day
10. Conducting team meetings
11. Visiting places to conduct the level 1 and level 2 interviews
12. Providing handholding support to the field team to conduct the interviews
13. Taking care of the field team
14. Validation of the collected information
15. Checking all data sheets, tables and map updating by DCs
16. Collating quantities Data in Town Map, Table A-D through extensive sharing and review of field records
17. Doing 25% back-checks
18. Providing regular feed back to the team coordinator
19. Coordinate between the field team and data entry team
20. Coordinating with the QMS team
21. Conducting town wrap-up meetings and sending all the collected data to the centre office

ROLES AND RESPONSIBILITIES OF FW

Field Workers are the backbones of the study. Data collection and reporting are the main roles of field workers. Other responsibilities include:

1. Maintaining field discipline
2. Following the rules of the team
3. Doing field work as per the field plan
4. Ensuring the quality of the collected information
5. Getting clarity about the study and its requirements

6. Identifying the correct KIs as per the requirements of the levels of field work
7. Completing the number of interviews as per the given target
8. Completing the documentation of the collected data
9. Helping FC in planning and also in doing set-up arrangements
10. Helping Data Collator in compiling the collected information
11. Participating in all the sharing meetings and expressing their findings and concerns
12. Coordination with QMS

ROLES AND RESPONSIBILITIES OF DC

The main role of Data collator is to compile the collected data and to provide guidance to the field team. Responsibilities include:

1. Maintain the stock of required formats, stationery and town maps
2. Preparing timetable with FS & FWs
3. Building clarity across the team about the information to be collected
4. Preparing all the charts before the field team sits for sharing sessions
5. Supporting FS in listing all major places in map
6. Providing regular feedback to the FS regarding the performance of FW and also quality of the collected information
7. Keeping check on the quality of the information
8. Building the capacity of the FW depending on needs
9. Selecting the place for verification and to be given to FS
10. Providing feedback about FWs to FS for pairing
11. Organising documentation and charts
12. Checking all the L1 and L2 formats filled in by the FWs
13. Collating quantitative data on Town Map, Table A-D through extensive sharing and review of field records
14. Collecting town summary, validation sheets, form FS
15. Arranging documents in order and packing for dispatch along with charts and map

ATTACHMENT 9 QUALITY MECHANISM SYSTEM

What is Quality Control?

Quality control is a mechanism to check quality at critical steps/periods and take corrective actions based on findings

Corrective Action: Corrective action is not only about taking steps to tackle a problem but also taking preventive steps so that the problem does not reoccur.

Pre-activity Prevention: Pre-activity preventive action means drawing up a list of non-conformities (quality problems) that can happen at any stage of field work and then taking preventive strategies to ensure non-occurrence.

Post-activity Prevention: Post-activity preventive action is like corrective action and is used when a quality problem has already happened, to ensure particular problem does not reoccur.

Quality for Mapping Study

- Accuracy in listing of locations
- Exhaustiveness in the listing
- All types of HRA mapped
- Accuracy in estimation of volumes
- Timeliness in completion
- Clarity, comprehensiveness in reporting
- Client interface at important junctures
- Providing expected support to NGO partners
- Friendly attitude and acceptable behaviour

QMS for Field Work

Quality plans have been developed for all five key roles in field work:

1. Zonal Manager
2. Field Supervisor
3. Data Collator
4. Field Workers
5. Quality Supervisors

Role and Responsibilities of QS

The main role of the quality supervisor is handholding the teams in ensuring non-occurrence of potential non-conformities (NCs) and in supporting the team in initiating corrective and preventive actions on NCs, if they happen. Responsibilities of the team include:

- Emphasis on working with the field team in ensuring quality
- Observing the field processes at all stages of field work and identifying NCs in those processes
- Recording NCs and working with the relevant team and supervisors in identifying root causes
- Working out corrective actions and preventive strategies with the field team
- Implementing corrective actions with the teams
- Discussions with FS and ZM for initiating preventive action of all NCs detected
- Ensuring assignment of responsibilities for taking corrective and preventive actions
- Following-up on all corrective and preventive actions and recording observations/findings after follow-up
- Resolving any concerns and doubts of the field team regarding quality assurance process (e.g. inspection)
- Emphasis on identifying major NCs rather than minor NCs following the principle of the "vital few, trivial many"
- Analysis of NCs by their root causes
- Sharing best practices and approaches of one team with other teams
- Working in co-ordination with FS and ZM
- Covering as many teams as possible to observe different stages and processes of field work with different teams
- Analysis of NCs by identifying the most frequently happening NCs at different stages of fieldwork
- 5% back-checks of locations
- Recording specific observations during fieldwork and providing them to report writing team
- Preparing a detailed NC register
- Continuously revising field work quality plan (adding more NCs, fieldwork process revisions, etc.) based on experience
- QS will complete three formats: NC Register, Validation Format and Analysis of NCs and their root causes

Note: Use this NC list for conducting training for DC, FS and Quality supervisors

LIST OF NON-CONFORMITIES AT EACH STAGE OF FIELD WORK

Key Processes	Non-Conformities
Set-up Process	All necessary documents and maps NOT Available with team (Town Map, DC letter, SP letter, ID Cards, field work plan)
	Insufficient copies of L1& L2 formats are available with the field teams
	Stationery not available in sufficient quantity during time of need
	Improper logistical planning & communication to the team
	Field team coming late to the town
Identifying places to visit for level1 KI interviews	Potential places (e.g. market, bus terminals, cinema halls, railway stations, arterial roads, etc.) not identified
	Improper exercise for identifying places of visit for Day 1 & D2
Pairing of field teams	Pairing not based on complementary skills
	Pairing not based on gender
	Insufficient understanding of field workers skills by FS
Allocation of places to team	Allocation of minor locations to some pairs
	Allocation of adjoining locations to different teams
	Allocation of too far away locations to a team
	No or partial clarity in some pairs about locations to be covered
Building clarity across the team on the information to be collected	Improper briefing of the different pairs of field team
	FS not clear about their roles and responsibilities
	Sharing session with the team not properly conducted
	No validation exercise to assess whether there is clarity
	Field Workers' attitude and sincerity problems
Preparation of list of KIs	Lack of sincerity in preparing the KI list by FW
	Team not clear about KIs before proceeding to field work
Planning schedule for the day	Impractical schedule to implement: too many places in a day
	Sloppy schedule: too little activity during the day
	Schedule not clear to teams
	Schedule not based on discussions with every team member
	Schedule not based around conveniences of KIs
Team meetings	Team meetings place, time and importance not clear to some of the teams
	Co-ordination problem between FS and DC
	DC/FS not allowing Field Workers to share their views freely
	FS not clear about importance of daily team meetings
Visiting Places Following and Sharing the Leads (Contact Persons)	Timing of visiting the place not as per the place/KI convenience
	Leads obtained from KIs are not followed
	Leads obtained from KIs are not recorded
	Supervisors not informed about the lead
	Supervisor does not pass on the lead to relevant team
	Any lead about other towns, villages, districts are not passed on to the relevant team

Rapport Building with KIs	No proper introduction
	Raising sensitive issues (e.g. this study is about finding sex workers, HIV/AIDS persons etc.)
	Continuous speaking with no listening
	False assurances raising expectations
	Providing faulty information
	Treating KIs with disdain
	Not addressing their fears/concerns and doubts
	Unfriendly body language
Identifying KIs - level 1 and 2	Speaking to KIs in a hurry
	Non-serious KIs (esp. those under the effect of alcohol)
	Improper observation of the area for selecting KIs
	Non-selection of variety of KI (in different occupations, primary/secondary/tertiary)
	More emphasis on secondary and tertiary stakeholders
Interviewing KIs - level I	FW hesitating to talk to strangers
	Lack of confidence in field team members
	Writing in front of KIs
	Meeting institutional KI (hotel, NGO etc.) without prior appointment /notice
	Bringing KIs to hotel/place of residence
	Paying KIs for information
	FS not demonstrating few initial interviews
	Exchanging addresses with KIs
	Not answering KIs' queries, doubts and concerns
	Discussion in a group
	Non-observation of the area during interview
	Field team partners not keeping close watch on each other
	FS disrupting the interview process by FWs
	Sudden gestures and mannerism disturbing interview process
	Not ensuring full understanding of the questions asked
	Not all or relevant questions related to level 1 are asked
	Not probing sufficiently
	Not covering at least 10 interviews per day at level 1
	FS -interviewing network operators and committing all the above NCs
	KI details (Education, occupation etc.) are not collected
	Large number of incomplete interviews
	Not validating information from one KI with another KI
	Not asking for leads (other contact persons)
	Some details as required in the format are not asked
Sharing sessions in the Field	Field meetings do not happen
	Field meetings are not conducted properly
	Not all teams participate in field meetings

	Teams feel that field meetings are not useful
	Crowded place for sharing session attracting attention
	Sharing session creates confusion
	Non-sharing of problems by FWs (only good things are shared)
	FS shouting at FWs in the field
	FS not motivating team members for sharing problems
	FS not able to solve or suggest corrective measures for problems
	FS not conducting sharing session properly
	Arguing too long with KIs
	Arguments un-necessarily between field teams
Interviewing KIs - level2	FW hesitating to talk to strangers
	Lack of confidence in field team members
	Interviewing KIs of the same profile
	Only one field worker profiling the spot
	Meeting institutional KI (hotel, NGO etc.) without prior appointment/notice
	Bringing KIs to hotel/place of residence
	FS not demonstrating place profiling initially to all field teams
	Paying KIs for information
	Exchanging addresses with KIs
	Not answering KIs' queries, doubts and concerns
	Fake interview
	Non-observation of the area during interview
	Field team partners not keeping close watch on each other
	FS disrupting the interview process by FWs
	FS shouting at FWs in the field
	Sudden gestures and mannerism disturbing interview process
	Not ensuring full understanding of the questions asked
	Not all or relevant questions related to level 2 are asked
	Not probing sufficiently
	Not covering at least 5 interviews per day at level 2
	FS interviewing network operators and committing all the above NCs
	KI details (Education, occupation etc.) are not collected
	Large number of incomplete interviews
	Non-validation of information from one KI with another KI
	Not asking for leads (other contact persons)
	Following up the leads given
	Contact persons details not given to Field worker by FS

	Arguing too long with KIs
	Arguments un-necessarily between field teams
	Some details as required in the format, are not asked
Key informant sheet	Date, district, FW ID, Town not filled in
	No separate sheet for each KI
	No separate sheet for each town
	Name and Address not taken
	Codes not filled in or in-correctly filled up
	Occupation not mentioned/recorded
	Not filled in legibly
	Not verified and signed by DC and FS
City / Town HRA information sheet (L1 format)	Wrong marking of target group (e.g. MSW for MSM)
	Date, district, FW ID, Town not filled in
	Codes not filled in or in correctly filled in
	Place and HRA estimates are not filled in/or not filled in properly
	Not verified and signed by DC and FS
	Notes column is mostly empty
Place profile information sheet (L2 Format)	Date, place, FW ID, KI no. Weekly market day not mentioned or incorrectly mentioned
	HRA typology not coded correctly
	Service providers, networks operators and spot details not given or given incorrectly
	Notes column is mostly empty
	Not verified and signed by DC and FS
	Five forms are not filled up
Collating data on key informants (KI Tracking Sheet)	DC does not review all KI sheets
	DC does not share problems observed in KI sheets with FS/FWs
	KI sheet update leaves out KI details of some places
	FS do not support DC in data collation
	DC corrects the problems/mistakes him/herself
	Number of completed interviews is not updated
	Recording in formats in not legible or correct or complete
Collating quantitative data in Town Map, Table A-C through extensive sharing and review of field records	No planned meetings for data collation
	Meetings creates more confusion rather than resolving them
	FS not supporting DC in data collation
	Frequency and estimates of HRA are wrongly shared (oral sharing without verification)
	Meetings are conducted in a hurry
	Meetings are not conducted every day
	Not maintaining updates on all the tables

	Table A: Frequencies and estimates are incorrectly/incompletely recorded (day-wise updates)
	Town Map: Not done at all
	Town Map: Information not properly sourced from table A
	Key local informant not used for preparing town map
	Info not validated twice before marking on map
	Revealing primary data on HRA to outsiders (local key informant for marking places
	on the map)
	Not updating map every day
	Unclear map boundaries
	Spots are not properly marked on the map
	Table B: Area and spot details have same spot by different names
	Table B: Not completed seriously
	Table B: Non-participation of all team members in compilation; esp. for problems arising in compilation
	Places for visit next day are not identified on the basis of table B
	Not updating the table B everyday
	Table C: not full reference to table B while preparing table C
	Same place is mentioned by different names in table C
	Updating process does not have verification element: table C
	HRA typology not filled up correctly
	Verifying whether table C covers all places of table B
	Support to DC not available from FS
	Estimates are not properly reconciled
Extracting qualitative data from the mapping	Lack of interest among FWs for such a sharing session
	Lack of initiative from DC and/or FS
	Lack of validation of qualitative comments made by FW
	Only few questions in the checklist are discussed
	Time of sharing session allows no fruitful discussion
	Points discussed in the session are not recorded or recorded incorrectly
	Team does not realise the importance of qualitative observations and sharing
	Qualitative Report not prepared
	Planning for next day done without participation of field workers
	Briefing about plan done hurriedly
	Some fieldworkers fail to understand the plan for next day
	Communication mechanisms are not re-planned for next day
	Peak days selected for spot profiling

Back-Checks by Field Supervisor (25% of the places)	Not conducting any back-checks
	Conducting back-checks not to the extent of 25%
	Not interviewing primary KIs as part of back-checks
	Sample for back-checks is not distributed geographically or for all target groups and for different field teams in a District
	No sharing of back-check findings with field workers
	Field workers take criticisms made by supervisor after back-check negatively
	Back-check findings are not entered in the validation format
	No actions taken on findings
	No follow ups on action taken
Back-Check by Quality Supervisor (5 % of the places)	Not conducting any back-checks
	Conducting back-checks not to the extent of 5%
	Not interviewing primary KIs as part of back-checks
	Sample for back-checks is not distributed geographically or for all HRGs and for different field teams in a District
	No sharing of back-check findings with field workers/FS
	Back-checks findings are not entered in the validation format
	No actions taken on findings
	No follow ups on action taken
Finalising Table A-C, Town Map, and other quantitative and qualitative details	Final sharing meeting for a town not conducted
	Sharing meeting conducted hurriedly
	Field teams do not participate proactively in the meeting/do not understand the importance of such a sharing session
	Verification of completion of all tables, maps and qualitative reports is not done
	Methodology learnings not discussed and captured
	Not all team members participate in sharing
	Lack of patience for the proceedings
Organising Documentation and Charts Transporting the documents	FS/DC do not organise charts and all documents properly
	Documents and charts are misplaced
	No proper handling during transportation
	No proper packaging of documents
	No immediate transportation of charts and documents

ANNEXURE 2

Site Assessment

1. EXERCISES FOR SITE ASSESSMENT

Number and Trend Map ("How Hot is the Spot"?)

Respondents

Visible and self-identified HRGs

Location

At all hotspots identified through Broad Map, and any other hotspot that might be subsequently identified through the course of the Mapping implementation in the site

Process

1. Settle respondents with an icebreaker
2. Ask the group to draw a map of the local area, including any local landmarks to orient the map. Ask them to mark the hotspots they themselves frequent, in reference to the landmarks.
3. Ask the group to rank the hotspots using symbols for high, medium or low according to the level of risk practice that puts HRGs at risk of HIV/STI infection at different hotspots.
4. Ask the respondents why they have marked different hotspots differently – is it according to numbers of HRGs who frequent that hotspot or the particular risk practice usually carried out at the hotspot which may carry more or less risk of HIV/STI transmission, or the frequency of risk practice, or any other reason? Let the HRGs suggest their own reasons rather than asking them leading questions. Do not contradict unless you have to clear misconceptions and myths.
5. Then ask respondents to look at the hotspots ranked as high. Ask them to discuss what change needs to happen generally to make the location into a medium or low rank. Then ask what individual HRGs or small peer groups could do to reduce risk practice in these locations. Again, do not contradict unless you have to clear misconceptions and myths.
6. Ask respondents to estimate the numbers of HRGs from different categories who usually frequent each hotspot on an average day. Let respondents debate among themselves to arrive at figures most members of the group are happy with. Against each hotspot on the chart ask respondents to put different symbols for different categories of HRGs and put the corresponding number next to each symbol (numbers can be represented through symbols too).
7. Ask respondents to draw a clock (or a line representing 24 hours of a day) and indicate on the clock (or the line) at what time of the day the numbers they have mentioned is to be found at the hotspot. Ask them to mark (with + and – signs, or with spots or *bindis*) different hours of the day to indicate how that number might fluctuate during the day.
8. Ask participants to draw a line indicating 7 days of a week and ask them to similarly mark the line to indicate fluctuations during a week.

9. Ask them to put symbols against the hotspot to indicate events or festivals in a year when the number might significantly go up or down.
10. Finish the session by asking the group to reflect on what they have shared and learned during the session that would be useful for them.
11. At the end of the session, note down the date, place, number of respondents (disaggregated by HRG categories) and your mapping team number on the back of the chart paper.

Outputs

1. Estimated numbers of different HRG categories in different hotspots
2. Timings when the HRGs are available at the hotspots (daily, weekly and special annual events or festivals)

Seva Chitram (Services Map)

Purpose

This is a method to assess availability and accessibility of different services in the site to HRGs

Respondents

Visible and self-identified HRGs

Location

At all hotspots identified through broad mapping, and any other hotspot that might be subsequently identified through the course of the mapping implementation in the site

Process

1. Ask the participants to draw a map of the site including a few main landmarks and ask them to indicate the hotspot where the HRG mapping team contacted them.
2. Ask the participants to include in the map any places or people that their HRG group could go to get support for HIV/STI prevention and treatment.
3. Ask the participants to put against each intervention:
   - What each service provides
   - How each service helps reduce risk of HIV/STI infection
4. Now ask the participants to rank the services high, medium, or low according to how accessible they are to HRGs like themselves (how often they access or utilise the services – often, sometimes, never).
5. Ask them to identify factors that make them use the services marked high or medium (such as distance, cost, behaviour of service providers, confidentiality, effectiveness of services provided, availability and timing and so on).

6. Now ask them to discuss the services ranked with low accessibility. What could be done to make these important services more accessible to HRGs like themselves?
7. Finish the session by asking the group to reflect on what they have shared and learned during the session that would be useful for them.
8. At the end of the session, note down the date, place, number of respondents (disaggregated by HRG categories) and your mapping team number on the back of the chart paper.

Outputs

1. Location of different HIV/STI related services in the site
2. Range of services offered by each service provider
3. Criteria by which HRGs judge a service to be accessible and available
4. Recommendations from HRGs about how to make services accessible and available to them

Why is it so?

Purpose

The method will help HRGs analyse the range of risk and vulnerability factors they experience that increase their susceptibility to HIV/STI transmission. This is will help to identify the strategies and intervention components that have to be put in place to enable them to avert the risks.

Respondents

Visible and self-identified HRGs

Location

At all hotspots identified through broad mapping, and any other hotspot that might be subsequently identified through the course of the mapping implementation in the site

Process

1. Ask participants to name the different kinds of behaviours that put people at risk of HIV/STI infection. Correct any misconceptions.
2. Pick one of the risk behaviours.
3. Ask them to draw a symbol of this risk behaviour in the centre of the flipchart inside a circle.
4. Ask “Why is it so?” and ask them to draw and or write the reasons for the risk behaviour in balloons.
5. Keep asking “Why is it so”, adding further reasons in connecting balloons until they can think of no more.
6. Ask the participants what the diagram says about:
   - What are the most important reasons (vulnerability factors) for risk behaviour?
   - What are the ways that the HRG group already try and reduce risk behaviour?
   - What would further help the HRG group avoid the risk behaviour in the diagram?
7. Finish the session by asking the group to reflect on what they have shared and learned during the session that would be useful for them.

8. At the end of the session, note down the date, place, number of respondents (disaggregated by HRGs and non-HRGs) and your mapping team number on the back of the chart paper.

Outputs

1. The factors that make particular categories of HRGs vulnerable to HIV/STI risks
2. Recommendations from HRGs about how to address some of these factors and risk reduction strategies

Sex Life

Purpose

The range of sexual partners of HRGs can be explored through this method. The method will also indicate the kinds of sex acts usually practised by a HRG with his/her sexual partners, helping to estimate the volume of penetrative sex, and therefore project needs for condom supplies.

Respondents

Visible and self-identified HRGs

Location

At all hotspots identified through broad mapping, and any other hotspot that might be subsequently identified through the course of the mapping implementation in the site

Process

1. Administer this method on a one-to-one basis with an individual HRG
2. Ask the HRG to put himself/herself at the centre of the chart
3. Ask him/her to draw pictures of his/her sexual partners all around his/her own picture in the middle and describe the partners (without naming them) – who are they, what do they do, how old are they, how are they related to the HRG, how did they meet, etc.
4. Then ask the HRG to indicate against each partner's picture or symbol what kind of sex did he/she had with the partner in the last one week, and how many times?
5. At the end of the session, note down the date, place and your mapping team number at the back of the chart paper.

Outputs

1. The range of sexual partners HRGs have – clients, *panthis*, boyfriend/husband, fellow *kothis*, wife, other women, etc.
2. The proportion and frequency of penetrative sex acts they engage in and with which category of sexual partners

2. CAPACITY STANDARDS FOR PARTICIPATORY SITE ASSESSMENT WITH HRGS

Rather than use site assessment as a one off process to begin a project, many organisations will carry out site assessment on a regular basis to review their programmes. For this reason, capacity standards have been developed so that organisations can continually improve their site assessment implementation, outputs and outcomes.

The site assessment capacity standards shown below are not indicators which can be objectively measured: rather they are designed to stimulate discussion in the organisation so that creative ways to optimise the site assessment process can be found. The capacity standards should be used in planning, then checked throughout the site assessment. The scores are intended to indicate where an organisation needs to take action to maximise the impact of their site assessment. The basic capacity standards in this guide are useful only to the extent that users are committed to honest and critical reflection, and they can be used by organisations (with or without an external facilitator) to identify their own capacity building needs, plan technical support and monitor and evaluate their site assessment progress.

Scoring of capacity standards can be carried out using the scores below:

DK= Don't know or not applicable
1= Needs urgent attention
2= Needs major improvement
3= Satisfactory, room for some improvement
4= Satisfactory, room for a little improvement
5= Exemplary, cannot be improved

Although difficult, a frank and critical approach will mean that the final scores are more meaningful and useful to the organisation. In particular, participants should think carefully before assigning a '5' – is there really no room for improvement? Even if the standard is being reached, are there opportunities to improve the quality of the work?

Capacity Standards for Site Assessment with HRGs	DK	1	2	3	4	5
1. Methods used in site assessment should be dialogue-based, highly participatory and give the opportunity for HRGs in the site to analyse barriers to reducing HIV risk and find solutions. In other words, as well as generating information, site assessment should mobilise HRGs and strengthen their ability to critically reflect on reducing HIV risk.						
2. Reporting formats should be developed which are easy for site assessment team members to use. The team should meet at the end of each day to assess the information generated, look at what gaps still remain and to plan site assessment activities for the following day.						
3. During site assessment, the teams need to be very careful to keep information secure and confidential. They must also take care not to make false promises or raise unrealistic expectations about what will happen after the site assessment.						
4. At the end of site assessment, a feedback and project design or planning meeting needs to be held immediately. All the main stakeholders, including the site assessment team members and HRG representatives from the site should be present. The site assessment team members should have time before this meeting to organise how they will present the findings to make sure that confidentiality is maintained.						
5. If nothing happens or there are no changes in the site after site assessment, the momentum will be lost. Prior to site assessment, funding must be secured for follow up activities. Any activities initiated by the HRGs themselves as a result of site assessment should be applauded and supported.						

ANNEXURE 3

Peer Educator Training

COURSE CONTENTS

Section I	Sex and Sexuality
Section II	Understanding Sexual and Reproductive Health
Section III	STIs and the Role of PEs in STI Management
Section IV	HIV and AIDS
Section V	Identifying Risk and Vulnerability Factors
Section VI	Negotiation Skills
Section VII	Condom Promotion
Section VIII	Self-Esteem
Section IX	Care for Persons Living with HIV and AIDS (PLWHA)
Section X	Monitoring
Section XI	Networking
Section XII	Advocacy
Section XIII	Community Mobilisation
Section XIV	Evaluation

SECTION I SEX AND SEXUALITY

Every human being is a sexual entity. Feelings about the body and sensual pleasures are all part of the human personality and sexuality. Sex is one of the basic physiological needs of human beings, and people engage in sexual activity primarily for intense pleasure. In many societies, popular norms limit sex to the purpose of reproduction. But in fact, people enjoy sex for reasons of physical and emotional pleasure and gratification.

Procreation can be one of the results of heterosexual intercourse. But there is a wide range of sexual activities which are not related to procreation. This is clear from the ratio of how many times a married couple engage in sex to how many children they have during the span of their conjugal life. As another example, homosexual males and females do not reproduce through their sex acts. And women are not fertile throughout the whole period of their menstrual cycle, but they may be sexually active all the time. Hence it is imperative to separate the two issues of sex and reproduction.

Sex and sexuality has remained a secret subject for many years, but with the emergence of HIV it has become an area of prime concern. The concept is deeply entrenched in the social, cultural and historical construct of a given society and far exceeds the biological arena. In Indian society, sex is generally seen as a necessary evil, and it has no social sanction beyond its function for reproduction within the marital bounds of a man and woman. Society does not acknowledge the aspects of pleasure, comfort, happiness and intimacy which are intrinsic to sexuality. Any sexual practice other than sex for reproduction is perceived as a moral sin. In this context, sex work is considered a sinful profession.

This session on sex and sexuality addresses this conceptual framework. It is necessary to differentiate and discuss on the meaning of the terms “sex” and “sexuality”.

Step 1 Participatory Exercise

The purpose of this exercise is to make participants consider differing moral perspectives on sexuality. In particular, it helps FSWs analyze and clarify their understanding of how moral judgments are associated with sex acts in situations where the FSWs have no bargaining power, are completely exploited and are highly vulnerable, as against situations where they can engage in sex for mutual benefit. The discussion following this exercise can also enable FSWs to reflect on the psychological attitudes behind such moral judgments. This exercise is expected to bring clarity to their thinking about their self-image.

A story is told that depicts three mutually exclusive options for a girl to cross over to the other side of a river, where her fiancé is waiting. In all three situations, she must negotiate with others who are creating obstacles for her. The FSW must put herself in the place of the girl and choose one of the following three options which she thinks she could morally stand by.

1. She crosses the river by walking over a bridge, but she will unavoidably encounter a man on the bridge who is a habitual and brutal rapist.

2. She crosses the river by boat, but the boatman will ask to have sex with her in return for rowing her over to the other side of the river.
3. She swims across the river, but she risks her life as the crocodiles in the river might eat her.

The participants take the role of the girl in the story and individually choose one of the options. Having chosen, they divide into three groups, one for each option. In their groups, they discuss the moral reasons for their choice. Each group has a facilitator who allows the FSWs to freely imagine and derive the consequences of their choice. No one's views should be undervalued. Each group is asked to make a presentation to the other groups at the end.

The attitudes derived from each of these options may be summarised as follows:

1. Despite brutal physical abuse by the rapist, the moral response is that she was a victim of the situation.
2. An atmosphere of mutual benefit where the girl too has an equal bargaining power and an opportunity to voice her priorities.
3. Moral chastity placed over physical survival.

Discussions on moral judgments after the presentations should be guided by the facilitators in order to apply them to the contexts of "sexuality" and the "sex trade".

Step 2

Participants brainstorm the terms they know which are somehow related to sex. List also all the local terminologies they know, including slang.

For example:

- Sexual encounter
- Menstruation
- Man and woman
- Woman gives birth to child
- Penis
- Vagina
- Anus
- Pubic hair
- Vaginal sex
- Anal sex
- Homosexual
- Male who has sex with males
- Persons who like to have sex both with male and female partners
- asturbation
- Sex worker
- lients of FSWs

Group discussion on how FSWs perceive their sexuality and the sex work profession. Encourage them to raise questions and myths regarding sex and sexuality. Group presentation and listing of all the issues raised.

Issues that may be raised include:

- Clients are sinners and we have to engage ourselves to meet their sinful desires
- Clients visit us to fulfil their sexual desire; there is nothing wrong about it
- Masturbation is abnormal, and women especially should not do this act
- Menstruation is a curse
- Sex is unclean and genitals are dirty
- Sexual urge is biological and emotional need. We the FSWs are giving sexual services, and we should be considered as professionals

Step 3

Discuss the dominant discourse on sexuality. How does society perceive sexuality?

Possible responses:

- Sex is a sin
- One should not discuss sex
- One should not learn about sex
- Sex is necessary only for procreation
- Any sexual activity not intended for reproduction is morally unacceptable
- Sex outside a marital relationship should be banned
- People who visit FSWs are doing wrong as this behaviour contravenes socially sanctioned sexual rules
- If there is any acknowledgement at all of sexual needs beyond procreation, it is only for men. Women should always be faithful to a single man
- Social practices strictly prohibit the expression of women's sexuality. For generations of women, sex has tended to be more a duty than a pleasure
- FSWs are morally corrupt as their sexual behaviour is different from the socially accepted sexual rules

Step 4

Discuss and define the concepts of sexual desire, sex and sexuality.

- **Sexual desire** is a fundamental need of human beings as biological creatures. Sexual desire has components of mutual pleasure, comfort, and satisfaction. Fulfilment of sexual desire makes a person healthy. One of the results of sexual intercourse between a man and woman may be the birth of a baby.
- **Sex** refers to the **biological attributes** that identify a person as male, female or transgender. Terms such as man and woman, pregnancy and childbirth, menstruation, and terms denoting sexual organs all come under the concept of sex.
- **Sexuality** refers to **manifestations of sexual preferences and behaviours**. Terms such as vaginal sex, anal sex, homosexual, males who have sex with males and persons who like to have sex both with male and female partners all come under the concept of sexuality.
- Understandings of sex and sexuality are deeply rooted in the social, cultural, and historical construct of a given society. In Indian society, sex and sexuality are seen as sinful and only necessary for reproduction. Any sexual activity beyond the boundaries of reproduction is not sanctioned by society. The aspects of mutual pleasure, comfort and happiness are not acknowledged by society. But these are a very basic biological and psychological need of human beings.

Discuss appropriate and inappropriate notions of sex and sexuality.

Step 5

Participatory discussion on different type of sexual activities.

- Different kind of sexual activities
- Penetrative, non-penetrative and safe sex

Time: 1.30 hrs

Expected Outcomes

- Participants identify different local terminologies related to sex and sexuality
- Participants understand society's perceptions of sex and sexuality
- Participants understand the broad concepts of sex and sexuality, and an empowering attitude towards sex, sexuality and sex work profession

Section II Understanding Sexual and Reproductive Health

Low socio-economic class and social marginalisation impede FSWs' access to information, including health-related knowledge. Their opportunities to learn about reproductive health are very limited. Poor basic knowledge and misconceptions about the body, its different parts and their functioning, hygiene and disease processes, etc. increase their vulnerability to ill health, and especially infection with STIs and other reproductive health hazards. Health education can help FSWs understand their bodies, the importance of self-examination and the need for health check-ups, and increases their control over their bodies and their health.

Step 1

Discuss PEs' perceptions about their bodies.

Some of the opinions may be:

- We work with our bodies to give services to our clients
- The body is like a machine, having different parts for specific functions
- Those different parts and components work as a whole and in coordination with each other
- Like a machine, the body needs food, cleaning, maintenance and caring

Based on the perceptions expressed, the discussion can cover what the body is and why every person should respect his/her body to remain healthy. FSWs offer bodily pleasure to their clients, and they do not need to consider their bodies as dirty or shameful. They should keep their bodies healthy and well cared for like anybody else.

Time: 20 minutes

Step 2

- Discuss health and hygiene, including the necessity of maintaining personal and environmental hygiene, and its relevance to controlling diseases.
- Discuss common communicable and non-communicable diseases

  - **Communicable diseases:** air-borne, water-borne, contamination through body fluids, etc. (e.g. TB, malaria, cholera, diphtheria, STIs, AIDS)
  - **Non-communicable diseases:** diabetes, arthritis, cancer, etc.

Time: 20 minutes

Step 3.

Brief discussion on various parts of the body and its systems.

For example:

- Digestive, circulatory, skeletal, nervous, reproductive systems, etc.
- Different organs and sensory organs associated with the systems

Time: 10 minutes

Step 4

In this session, major elements of the male and female reproductive systems, their anatomy and physiology are discussed. A simple diagram showing the male and reproductive organs can be used to explain the anatomy.

- **Female reproductive anatomy:** External organs – vulva, labia and clitoris. Internal organs – vagina, uterus, cervix, ovaries, fallopian tubes
- **Female reproductive physiology:** menstruation, menarche, menopause, ovulation, fertilisation, conception, pregnancy, childbirth, etc.
- **Male reproductive anatomy:** External organs – penis, scrotum, testes. Internal organs – vas deferens, seminal vesicles, prostate gland, urethra
- **Male reproductive physiology:** puberty, formation of sperm and semen, storage of semen, erection and ejaculation

The discussion should emphasise that reproductive organs are like any other part of the body and we should not neglect them if problems occur.

Time: 1 hour

Step 5

Discuss common reproductive health concerns.

- RTIs/STIs
- Problems related to early and teenage pregnancy, repeated pregnancy, prevention of pregnancy, MTP, care for pregnant mother, safe delivery, etc.

Time: 15 minutes

Step 6:

Discussion of RTIs.

Reproductive Tract Infections (RTIs): Infections that affect the reproductive tract of males and females. RTIs are of three types:

1. Sexually transmitted infections (STIs). Caused by virus, bacteria, or fungal microorganisms, which are passed through unprotected sexual intercourse with an infected partner.
2. Microorganisms that are normally present in the vagina multiply and cause infection. This type of RTI is mostly caused due to inadequate maintenance of personal, sexual and menstrual hygiene.
3. Infections caused due to inappropriate medical procedures, such as unsafe abortions.

Time: 15 minutes

Step 7:

Discuss reproductive health rights of FSWs.

- Availability of reproductive health information and services
- Right of FSWs to take decisions about their reproductive and sexual health

Time: 10 minutes

Expected Outcomes

- Participants understand the basics of the body, its different parts, systems and functions
- Participants understand why every person should respect his/her body
- Participants understand the basics of health and hygiene
- Participants know about common communicable and non-communicable diseases
- Participants know the basics of reproductive functions and reproductive health concerns

Section III STIs and the Role of PEs in STI Management

In community mobilisation among FSWs, the significant issues regarding STI management are:

- Community must identify the core issues and generate options for solutions
- Solutions must be tailor-made and integrated with adaptability, and services must be provided with active participation of the community
- The community members must be in a position to monitor the delivery mechanism and quality of services
- Clinic setting must not be seen merely as a place for treatment but must be positioned as a space for social interaction and for nurturing relationships. The structure and processes of the STI service delivery mechanism must be largely designed and controlled by the FSW community

These are the basics to establish control over access and utilisation of the services by the community. The STI management approach in community mobilisation calls for the Four "D"s:

1. De-stigmatisation of sex and sexual illness

The fundamental prerequisite is to remove the stigma attached to the profession of sex work and develop a non-judgmental attitude towards sex and sexuality. One should have respect for her body and its different parts, including the genitals. There is nothing sinful in acquiring an STI, and she has the right to quality treatment.

2. Demystification of technical aspects of STI services

Conscious efforts must be made to demystify STI management services. Treatment procedure must be made very clear to the community members. Clinic attendees have the right to be informed about their illness and the treatment procedure.

3. Decentralisation of STI management services

Decentralise the STI management procedure from clinic to community level. Prescription is not the only component of treatment: PEs and ORWs are responsible for counselling, communication, ensuring compliance with treatment and condom promotion.

4. Democratisation of STI management services

From a governance perspective, the FSW community's control over STI management must be ensured. An STI management team should be built up, comprising representatives from PEs, doctors, counsellors, paramedical staff, etc. An information-sharing mechanism between clinic and outreach staff should be established for efficient service delivery.

Step 1

Keeping this conceptual framework in mind, discuss STIs and their management. Ask PEs what they know about STIs.

Different opinions may be:

- STDs are sinful diseases
- Symptoms should not be disclosed
- If we disclose that we have STIs, customers won't visit us
- We are in the sex work profession, and STIs are a professional hazard
- STIs are like any other infection and should be treated properly

Discussion continues based on the understanding of PEs. Explain clearly what sexually transmitted diseases are. Emphasize project's non-discriminatory attitude towards STIs. STIs are viewed as an occupational disease; explain vulnerability of FSWs to these diseases.

Time: 30 minutes

Step 2

- Ask the participants to discuss their knowledge regarding the symptoms of STIs, including local terms used to denote symptoms of STIs. Record all the known symptoms on chart paper.
- Start the discussion on STI symptoms according to partcipants' understanding. Make necessary clarifications where their knowledge on symptoms and the diseases is inappropriate or incomplete. Encourage the participants to raise questions where they feel uncomfortable. Avoid using too many medical terms and use local language.
- This session can be arranged with a slide show demonstrating the symptoms to give PEs a clearer idea.

Symptoms

- Genital ulcers: single painless, multiple painful
- White discharge: vaginal (curdy, frothy, with offensive smell), cervical
- Urethral discharge
- Burning sensation while passing urine
- Pain in lower abdomen and deep dyspareunia
- Scrotal swelling
- Vulval swelling
- Swelling of inguinal glands
- Warts: pearly and cauliflower
- Jaundice

STIs

- Syphilis
- Gonorrhea
- Chancroid
- Lympho Granuloma Venereum (LGV)
- Bartholonotis
- Trichomoniasis
- Candidiasis
- Chlamydia
- PID
- Herpes simplex
- Warts: Condyloma accuminata, Moluscum contagiosum
- Scabies
- Hepatitis B & C
- AIDS

Time: 1.5 hrs

Step 3

Discuss the risks from STIs if they remain untreated.

- Can cause serious illness
- Enhance the chance of contracting HIV (ulcerative STIs)
- Untreated syphilis can lead to mental inertia
- Some STIs can be passed through next generation if a pregnant mother is infected (e.g. syphilis/ gonorrhoea)
- Longstanding gonorrhea can constrict or even block urinary tract
- Chronic cervicitis can cause infertility

Encourage participants to ask questions, and give clarifications accordingly.

Time: 20 minutes

Step 4

Discuss the ways STIs are transmitted. Presentation of pictures or animations on the issues can be arranged.

Transmission Routes

- Unprotected penetrative sexual encounter with infected person
- From infected mother to child eg. HIV, syphilis
- Use of infected blood for transfusion, e.g. HIV, hepatitis B and C
- Through infected needle/syringe

Talk about why women are more prone to get infected by STIs/HIV than men.

Physiological factors

- Wider mucosal area in the reproductive area in women, and semen remaining in vagina for long period of time
- Due to concealed nature of reproductive organs of women, symptoms often apparent only after a long period

Social factors

- Low socio-economic status families ignore or overlook health issues, including reproductive health. Even women themselves give little attention to their health
- Lack of information regarding the diseases
- Women have less control over their reproductive health
- STIs have linkage with sexual behaviour and thus it is not socially acceptable for a woman to disclose her disease

Participatory discussion on why FSWs are the most vulnerable – being women and in the sex profession.

Time: 30 minutes

Step 5

Discuss management of STIs. With few exceptions, STIs are fully curable. One should get treated as early as possible and complete the treatment cycle as per the advice given by the doctors.

Treatment aspects

- Self-examination of genitalia
- Need for regular health check-ups since STIs often remain asymptomatic in women: opportunistic screening through speculum examination and blood test for VDRL
- Get treated immediately after occurrence of symptoms
- Compliance with treatment and consequences if treatment is not completed
- Follow-up of treatment
- Treatment of partner
- Referral to higher institution if the symptoms recur or persist

Prevention aspects

- Use of safer sex measures, consistent use of condoms

Time: 20 minutes

Step 6

Discuss PEs' role in STI management.

- Disseminate information to FSWs and their clients regarding STIs, counsel and motivate them to come to the clinic for health check up, and be alongside clinic attendees to give them confidence
- Ask and counsel them about compliance with treatment
- Put effort into bringing the partners to clinic for health screening
- Provide counselling on consistent condom use
- Monitor quality of services: maintenance of confidentiality, privacy, pay attention to whether non-judgmental attitude and friendly behaviour is extended by the project staff
- As active members of clinic management team, PEs should stay familiar with clinical procedures
- Participate actively in clinic meetings and provide feedback that will help in triangulating data gathered by the clinic team and outreach team as well. For comprehensive management of STIs, PEs are the link between clinic and outreach team

Time: 1hr

Expected Outcomes

- Participants understand that STIs are occupational hazards of the sex work profession.
- Participants know the common symptoms of STI and their local terminologies
- Participants know the name of some common STIs
- Participants know the risks from STIs if they remain untreated, mode of transmission of STIs and prevention of transmission
- Participants understand why FSWs are more vulnerable to STIs
- Participants know how to control STIs (prevention and treatment)
- Participants understand the role of peer educators in STI management

Section IV HIV and AIDS

Step 1

Ask the participants whether they have any knowledge about HIV/AIDS. Begin discussion about definitions.

The term AIDS stands for:

A = Acquired – not born with

I = Immuno – body's defence system

D = Deficiency – not working properly

S = Syndrome – a group of signs and symptoms

AIDS is not a single disease but a syndrome, a group of signs and symptoms resulting from weakening of the body's defence system, which is caused by a virus. HIV is the name of the virus that causes AIDS.

- HIV stands for Human Immunodeficiency Virus
- Being HIV positive does not mean that a person has developed AIDS
- Once a person gets HIV infection, he/she remains infected and infectious throughout his/her life
- Treatment can extend the lifespan of an AIDS patient, but it is expensive
- No curative treatment for AIDS has been discovered so far and thus AIDS is fatal

Step 2

Discuss signs and symptoms of HIV.

- When a person first becomes infected with HIV there may be some signs of illness or no signs at all, but the virus is multiplying in the body (window period)
- In the second stage of infection, HIV infected person has no symptoms
- In the third stage, AIDS-related symptoms occur. These include severe weight loss, persistent diarrhoea, night sweating, persistent fever, etc
- In the fourth stage the person suffers recurrent opportunistic infections, cancers, severe weight loss, fatigue, etc. This is the stage known as AIDS
- The infected person can transmit HIV to another person during **all** stages of infection through sexual or contacts or blood

Step 3

Ask participants whether they know the mode of transmission of HIV. List all their conceptions, and discuss any misconceptions.

How HIV can be transmitted

- Unprotected sex
- Blood and blood products
- Sharing of infected needle/syringe
- Infected mother to child (in utero or through breast milk)

Misconceptions (myths) about modes of HIV transmission

- Insect bite
- Sharing common toilet, bed, common clothing
- Casual contact e.g. hand shake, hugging, kissing
- Eating together
- Air-borne or water-borne
- Using common toilet
- While taking care of HIV infected persons

Step 4

How to prevent HIV transmission:

- Practice non-penetrative sex and use condom for every penetrative sex act. This is understood as "safe sex"
- Use of screened blood and blood products
- Use of sterilized needle and syringe
- Getting treatment of STIs as early as possible

Step 5

Discuss the social dimensions of HIV. Ask PEs what their attitude and behaviour would be if they learned that any of their colleagues was HIV positive. Role play a supportive attitude towards an HIV positive person.

Individuals with HIV or AIDS are kept isolated from society and alienated even by their family members. This creates tremendous emotional and psychological stress, which may lead to extreme depression and feelings of fear and guilt.

Time: 2 hrs

Expected Outcomes

- Participants know what HIV and AIDS are
- Participants know the signs and symptoms of HIV infection
- Participants know the mode of transmission and prevention of HIV
- Participants understand the desired attitude towards HIV positive persons

Section V Identifying Risk and Vulnerability Factors

Step 1

- Divide the participants into groups. Assign each group a particular category of sex work, e.g. brothel based, highway-based, *dhaba*-based, home-based, street-based, etc.
- Ask each group to prepare a role play depicting a situation or behaviour that puts them at risk of STI or HIV transmission.
- After each role play ask participants to identify the risk behaviour and vulnerability factors depicted in the act. List the risk behaviour and vulnerability factors in relation to each group.

> **Risk behaviour** is behaviour that puts someone directly at risk of HIV/STI infection, such as unprotected anal or vaginal sex.
> **Vulnerability factors** are factors that make risk behaviour more likely and which therefore put someone indirectly at risk of HIV/STI infection. For example, having group sex, being poor or being female.

Step 2

Facilitate a discussion to encourage the participants from all groups to enhance the list. Ensure that the participants clearly understand the difference between risk and vulnerability and also the link between the two.

> Risk behaviours are made more likely by vulnerability factors, but vulnerability factors in themselves do not lead to HIV infection.

Ask the group if risk and vulnerability are mutually exclusive and if any programme would be successful if we work on only one element, either risk or vulnerability. Discuss a few risk reduction and vulnerability reduction strategies in the context of sex work.

> **Risk reduction** addresses the immediate factors of sexual transmission, which is mainly because of sex work as an occupation. Risk reduction strategies include:
> - Ensuring correct knowledge about STIs/HIV
> - Ensuring access to treatment of STIs and other health problems
> - Access to male and female condoms
> - Improving condom negotiation and decision making skills in sexual encounters
> - Working with clients/partners of sex workers

Vulnerability reduction addresses underlying factors affecting transmission: poverty, lack of human rights, gender relations, stigma and discrimination, and legal framework. Vulnerability reduction strategies include:

- Providing economic alternatives to FSWs
- Basic amenities like ration cards
- Children's education
- Promoting legal reforms
- Sensitising/educating clients and police against violence against FSWs
- Promoting participation and decision-making of FSWs in sex work programmes

Be sure to emphasise that without understanding and addressing vulnerability factors, behaviour change is not possible. Most of the time changing behaviour is not easy. Only when vulnerabilities are addressed do people respond favourably to knowledge and information. If we are willing to address and accept the vulnerability factors, the HRG is more likely to be willing to find effective and lasting solutions.

Time: 1.5 hrs.

Expected Outcomes

- Participants understand the risk and vulnerability factors involved in sex work, and can distinguish between the two
- Participants know several risk and vulnerability strategies

Section VI Negotiation Skills

This section addresses how peer educators can help FSWs to improve their negotiation skills. Providing information on safer sex practices to FSWs is not enough to ensure safe behaviour. It is not a question of the attitude and behaviour of the FSW, but rather of the power of her clients. Even being fully aware of the necessity of using condoms, an FSW may be compelled to jeopardize her health out of fear of losing her customers. In the case of FSWs controlled by madams or pimps, a significant share of the FSW's income usually goes to these people, leaving the FSW with meagre resources. In this situation a FSW cannot easily refuse her clients. These power relations often determine the outcome of negotiations between FSWs and their clients.

Improvement of self-esteem along with the attainment of technical negotiation skill is imperative for FSWs to negotiate better with their clients and other power brokers. Discussion of negotiation skills must be carried out keeping in mind the context in which FSWs have to negotiate.

Step 1

Discuss the issues that hinder safer sex practices by FSWs. List all the issues raised by the PEs.

- Clients are not willing to use condoms
- Madams/pimps force the FSW to practice unprotected sex
- FSWs don't know how to negotiate condom use with their clients
- Some FSWs are extremely depressed and see little difference between living and dying
- FSWs' inability to make decisions about their life
- Clients or power brokers force FSWs to have sex without a condom
- FSWs have limited income opportunities and are afraid of losing customers
- If a street-based FSW keeps condoms in her bag, police may arrest her and demand money for her release

Discuss how to resolve these situations. Take the points one after another, determine the stakeholders with whom FSWs have to negotiate and identify possible solutions. Most of the issues may not have any immediate solutions. Issues that may come up include collective bargaining, empowerment of FSWs, improving self-esteem, advocacy, the need for more economic options, etc.

Step 2

Discuss approaches to negotiation with different groups.

Clients	Exploring their business acumen and packaging of services to motivate clients in safer sex practice. For example:<br>- Showing keenness to ensure pleasure through a variety of sexual activities<br>- Showing caring and loving attitude towards the clients<br>- Adequate foreplay for the maximum pleasure depending on client's desire<br>- First, stimulate the client and when the client gets aroused explain that a condom will not reduce the pleasure but enhance the enjoyment and protect client's health<br>- FSW puts the condom on client as a loving gesture
Madams/Pimps	Convincing the madams/pimps by raising the issue of mutual benefit from their business perspective. For example, if the FSW remains healthy she can earn more and ensure income for madam/pimp. Emphasizing their positive role such as setting norms for condom use by the clients that can help their girls to convince the clients
Police/Administration	Sensitizing judiciary and its administrators on the technical socio-clinical issues that the TI strives to address and how the judicial attitudes and legal provisions intersect. The sense of absolute authority, moral guardianship, and power with which the police deal with the FSWs often leads to harassment and violence. Such abuses directly increase the vulnerability of FSWs, who lack a legal remedy
Persons belonging to mainstream society	Raising the issues pertaining to their social and legal status and its consequences. Expressing how this situation restricts the FSWs' enjoyment of their human and citizens' rights. Emphasizing the role of people other than FSWs in challenging the exploitative situation and in establishing FSWs' rights to self-determination. Creating broader alliance and support base by involving people from various spheres of society.

Role-play how FSW could negotiate on condom use with a client, with a madam and with exploitative police personnel.

Time: 1.5 hrs

Expected Outcomes

- Participants identify the factors that hinder FSWs from negotiating with their clients on safer sex practices
- Participants identify some of the issues to improve FSWs' negotiation skills
- Participants learn basic negotiation skills

Section VII Condom Promotion

Step 1

Ask the participants to talk about what they know about condoms. List terminology they use for condoms. Ask them to explain what a condom is.

- It is a rubber sheath. It is a long thin tube when rolled out. At the lower end it is closed and has a teat, which collects the semen. The condom acts as a wall and prevents the sperm and STI-causing germs and HIV from entering the vagina, and from female genital parts to the penis
- It acts as a barrier against STI and HIV/AIDS transmission
- It acts as a contraceptive device

Display a condom and give a condom to each participant so that they can see and feel it.

Step 2

This session teaches PEs about the correct use of condoms. Ask PEs to demonstrate putting a condom on a penis model. Then demonstrate correct condom use with the penis model.

- Put on the condom only after the penis becomes fully erect. Open the packet carefully without damaging the condom
- Hold the tip of the condom ensuring no air bubbles form inside and slowly unroll it to full length so that the penis is completely covered
- Ensure that the condom is in the correct position before beginning sexual intercourse.
- Immediately after ejaculation withdraw the penis from the vagina (or anus)
- Remove the condom carefully without spilling the semen
- Tie a knot so that the semen can not spill out and then dispose of in a dustbin
- Do not reuse a condom
- Improper use of condom can damage it, resulting in tearing of condom, which could lead to HIV/STIs or unwanted pregnancy. Care should be taken while using condom
- While giving the condoms to the FSWs, PEs should check the expiry date

Step 3

Ask participants why people do not use condoms and the misconceptions about using condoms.

- Using condom during sex is irritating
- Condom will tear during intercourse
- Condoms reduce sexual pleasure
- Condom is sticky and oily
- Erection goes before using condom
- Problem of buying
- Double condoms will provide better protection
- Use of condom implies lack of emotional feeling of her love for the partner
- Condom is barrier of “mistrust”between two partners

Clarify misconceptions.

- Condoms are soft and lubricated, and proper use of a condom does not cause irritation
- The process of wearing a condom is pleasurable, as the FSW puts the condom on her client as a loving gesture
- FSW must convince the client that if he uses condom he will enjoy himself more without any tension or apprehension about getting infected by STIs/HIV

As an exercise, ask a PE to put a condom on one finger. Tell her to touch various materials with the finger, and ask whether she can differentiate between them. Explain that the condom does not create any barrier of feeling.

Ask PEs to share practical experience of what do they do in these situations.

Step 4

Discuss availability of condoms. Ask PEs where condoms are available. List all locations/channels.

- With PEs
- Medicine shops
- Other shops
- Clinic

Step 5

Condoms should be stored in a cool dry place. Discuss how and where the FSWs can store their condoms.

Time: 2 hours

Expected Outcomes

- Participants understand what condom is and why it should be used
- Participants know proper use of condoms
- Participants know some of the methods for convincing clients for use condoms
- Participants know about the availability of condoms and condom storage

Section VIII Self-Esteem

Women engaged in the sex work profession are rarely seen as an occupational group. Rather they are categorized as a group of women that poses a threat to sexual morality and social stability. Although sex work is an age-old profession, FSWs are an invisible part of society. Their class, caste, gender, and occupation relegate them to a most marginalized position. Most FSWs have a low social class and economic background, and being in this socially unaccepted profession they have very low self-esteem.

These are critical concerns while dealing with the lives of FSWs. Only if they learn to value themselves will they think to protect their life and health. It is thus imperative help FSWs value themselves as human being and establish a positive sense of their identity. This section aims to boost the morale and self-worth of FSWs.

Step 1

Discuss what PEs think of themselves. Address different aspects of self-esteem following the matrix.

Aspects of self-esteem	What we think of ourselves	What we must do to enhance our self-esteem
**As women engaged in sex work profession**<br>- Do we consider our work like other livelihood options or it is something else?<br>- Do we think of ourselves as sinners or as workers who earn our own subsistence?<br>- Are we ashamed of being in this profession?<br>- Are we able to disclose our occupational identity to our families and children?		
**As human beings**<br>- Do we think that our lives are valuable?<br>- Do we also have dreams and aspirations for our future and can we express these feelings?<br>- Do we think that we should have right to live with dignity?		
**Ability to make decisions about our mental and physical well-being**<br>- Do we think that we can take decisions about entertaining customers when we feel sick?<br>- Do we take decisions about seeking treatment?<br>- Do we think that we should get equal and non-discriminatory health services from health service providers?<br>- Do we think that we should have the right to information?		

**Social identity beyond our occupation**<br>n Do we think of ourselves only as women in the sex work profession? Or do we have other material and emotional needs?<br>n Do we think of ourselves as having responsibility for other social causes?		
**Our legal status**<br>n Do we think that we can ask police about the cause of an arrest or raid?<br>n Do we think police should not harass us during raids?		
**Our political status**<br>n Do we think we should have a ration card, voter identity card?<br>n Do we think we should enjoy our rights as citizens and voters of this country?		
**Our civic amenities**<br>n Do we think we should get the same basic civic amenities as any other citizen?		
**As mother**<br>n Do we take decisions regarding the lives of our children?<br>n Can we admit our children to school with only the mother's name as legal guardian?		
**As peer educator**<br>n Can we be respectful health educators?<br>n Can we be community representatives?<br>n Can we be community organisers?<br>n Can we be responsible social beings?		

Discuss what actions need to be undertaken to strengthen confidence and enhance self-esteem.

These issues of empowerment of FSWs may come up:

- Strengthening information base on the issues concerning their health and rights as human beings
- Creating space so that they can articulate their needs and demands within the programme, within sex trade and within broader society
- Making them more visible in public sphere as persons with social responsibility and dignity.
- Building up community feeling through networking and collectivization
- Enabling them to take decisions as an individual and as community
- Supporting them so that they will be able to take actions on the basis of their decisions through formation of self-help groups

Time: 1.5 hrs

Expected Outcomes

- Participants able to identify the factors behind the low self esteem of FSWs.
- Participants motivated to initiate the process of boosting their self-esteem.

Section IX Care for Persons Living with HIV and AIDS (PLWHA)

HIV/AIDS has emerged as a major social problem as well as a medical challenge. The stigma attached to the disease often causes social discrimination and the ostracising of the person living with HIV/AIDS and their family. The PLWHA may be denied employment, housing and basic social amenities, and may even be discriminated against by healthcare providers. The psychological pressure of living with the disease or having a family member with AIDS can lead to depression. Care and support for PLWHA has become a significant concern in HIV/AIDS intervention programmes.

Step 1

Discuss social and psychological problems faced by PLWHA.

Step 2

Discuss the services needed by PLWHA.

- **Medical care:** general treatment, blood test with pre- and post-test counselling, treatment of opportunistic infections, anti-retroviral therapy (ART), maintenance of health and hygiene, nutrition, safer sex practice, testing of spouse, care for expectant mothers, etc.
- **Legal support**
- **Psychological support**: counselling, coping with trauma
- **Social care:** restoration of human rights among family, immediate community, at healthcare service institutions and in workplace

Step 3

Discuss the role of HIV positive persons in HIV prevention activities and the necessity for their involvement in the decision-making process in programmes for PLWHA. Discuss the role of positive people's networks for care and support for PLWHA. Discuss how HIV positive people can unite in forums to extend psychosocial support to their peers, generate awareness and undertake initiatives against social injustice and discrimination.

Step 4

Discuss PE's positive attitudes and roles towards PLWHA.

- We will stand by the HIV positive person and their family
- We will be the last-stage counsellor
- We will motivate people, especially those with persistent STIs and the spouses of PLWHA to undertake voluntary counselling and testing
- We will take them to doctors and counsellors for referrals
- We will help them to cope with their HIV status
- We will counsel the family members with the permission of PLWHA so that they can provide support
- We will arrange awareness programmes against discrimination
- We will liaise with health care service providers to give proper treatment and non-discriminatory behaviour

Discuss the availability of Voluntary Counselling and Testing Centres (VCTC) in their operational area, and outpatient and inpatient services for the treatment of general ailments, opportunistic infections and anti-retroviral therapy

Time: 1hr.

Expected Outcomes:

- Participants understand the kinds of psychological stress faced by PLWHA
- Participants understand the kinds of support PLWHA need
- Participants know the role of PEs in PLWHA care and support
- Participants know about available facilities for PLWHA in their operational area
- Participants understand the role of positive peoples' networks in HIV prevention, care and support for PLWHA

Section X Monitoring

Monitoring is the continuous assessment of the programme's implementation to improve its quality and help it achieve its goals. It is particularly relevant for community-based programmes. In community mobilisation, PEs are empowered to monitor various aspects of the programme, so they must learn monitoring skills and methods. They should also be encouraged to consider what kind of information needs to be generated and how it can be processed to ensure the effectiveness of the HIV intervention programme and empowerment of the community.

PEs can capture a combination of qualitative and quantitative information to assess both service delivery and community empowerment. Apart from keeping records of day-to-day activities, they should provide suggestions for proper functioning of the programme, e.g. which strategies are not working as expected, measures to be adopted to overcome problems, whether targets are achieved and options for further improvement.

Since PEs must provide primary documentation of many programme activities, the system should be designed in a very user-friendly manner, keeping in mind the PEs' writing abilities.

Step 1

Discuss the necessity and usefulness of monitoring.

- Gives clear idea of day-to-day performance
- Helps us understand how much we have achieved and how services can be improved
- Helps identify problems while performing our daily activities and in developing strategies to overcome the problems
- Helps us plan follow-up activities

Step 2

Discuss what aspects of the programme related to service delivery should be monitored by the PEs.

The activities may be:

- Develop rapport and friendship with other FSWs
- Disseminate information on different aspects of STIs/HIV
- Motivate FSWs/clients to seek health check-ups
- Motivate FSWs/clients to use condoms
- Distribute/sell condoms to FSWs, clients
- Communicate with other stakeholders
- Bring FSWs/clients to VCTC and provide support to HIV infected FSWs

Discuss and list what kinds of information must be recorded to assess PEs' performance in different aspects of service delivery.

- Number of STD patients – FSW, client, other
- Treatment completed – FSW, client, other
- Follow-up of patients
- Number of persons communicated – FSW, client, other
- Number of condoms distributed
- Number of persons referred to VCTC

Step 3

Discuss the assessment of the aspects of self-esteem and empowerment, and how they can be captured in the ongoing record-keeping system. These aspects are essentially qualitative, but some quantitative information is helpful to substantiate the qualitative assessments.

Quantitative information may include:

- Number of FSWs showing keenness to form their own collective
- Number of FSWs showing interest in forming their own banking system
- Number of incidents of violence
- Number of FSWs arrested
- Number of protests against abuse/harassment
- Number of FSWs who participated in protests
- Number of girls rescued by the FSWs

Qualitative information may include:

- Role of FSWs in handling violence
- Role of FSWs in protests against abuse/harassment
- Attitude and assertiveness of FSWs in meetings and programmes
- Showing proactive attitude towards different activities of programme
- Showing proactive attitude towards collectivisation and taking initiatives for the betterment of the community

Discuss ways for PEs to do record-keeping. The procedure should be designed to reflect PEs' writing skills. If these skills are low, documentation can be done using pictures or colours. For example, different colours/pictures may be used to denote FSW, client, general patients, treatment completed, referrals to VCTC, etc.

In the following sample document, symbols and dots are used to convey categories of assessment and quantitative information.

Name of Peer Educator:

Date:

Service Delivery

1. *[Symbol of condom to denote number of condoms distributed]*

•

•

•

•

•

2. *[Symbol denoting FSW with STI]*

•

•

•

3. *[Symbol denoting client with STI]*

•

•

Empowerment

1. *[Symbol denoting incidents of violence]*

•

2. *[Symbol denoting protest]*

•

The form gives the following information:

Number of condoms distributed: 5
Contacts with FSWs with STIs: 3
Clients with STIs: 2
Incidents of violence: 1
Number of protests: 1

Step 4

Besides the ongoing system, PEs can participate in periodic cross-sectional surveys to assess improvement of self-esteem and awareness regarding their rights. Discuss how to assess the improvement of self-esteem of FSWs.

Indicators may include:

- Proportion of FSWs who identify themselves as such
- Proportion of FSWs who are not ashamed of disclosing their occupational identity to their families
- Proportion of FSWs who think that they should have the power to control their trade
- Proportion of FSWs who think that they should get equal civic amenities
- Proportion of FSWs who think that they should enjoy equal rights with other citizens
- Proportion of FSWs who have their own bank account
- Proportion of FSWs who have their citizenship (voter ID) card

Time: 1hr

Expected Outcomes

- Participants understand what monitoring is and the need for monitoring
- Participants understand the process of monitoring and record-keeping
- Participants understand the difference between quantitative and qualitative assessment

Section XI Networking

Networking is a vital part of the process of empowerment of FSWs. It helps them be unified as a community and enables them to take up initiatives to better their lives. Networking is crucial to build solidarity and collective strength. A direct consequence of networking may be the formation of FSW collectives. Through a collective, FSWs can challenge the structural determinants of their lives and fight to establish their right to self-determination.

Step 1

Discuss and brainstorm why networking is necessary.

- Networking brings together FSWs from different red light districts and other settings.
- We can share the problems that pertain to our work
- We can assess the needs and aspirations of FSWs from different areas and identify appropriate actions to respond to needs
- We can evolve strategies to improve our situation
- Networking widens our circle of allies and thus enhances our support base, which will help us to get our demands met
- Networking instils confidence among the FSW community

Step 2

Brainstorm individuals and groups with whom FSWs can network.

- FSWs at local, District State, national and international levels
- Children of FSWs, fixed clients
- NGOs/CBOs working in similar fields
- Other vulnerable groups and marginal communities

Step 3

Discuss strategy of networking. What strategies are needed to network with different groups?

- Visit FSWs in different red light districts, learn about their problems, conduct needs assessment
- Arrange information sharing meetings with FSWs at regular intervals
- Exposure and exchange visits
- Develop system of communication for regular information sharing
- During any emergency situation, immediately extend support for the concerned group
- Create forums for networking with different groups
- Team building for networking with different groups

Step 4

Discuss PEs' expectations for outcomes of networking.

- Formation of District and State level networks of FSWs
- Formation of FSWs' collective
- Detailed plan of action

Time: 3 hrs

Expected Outcomes

- Participants understand the necessity of networking
- Participants know with whom FSWs should network and strategies for networking
- Participants understand potential outcomes of networking

Section XII Advocacy

Advocacy involves influencing policy, be it at the level of an organisation, the government or society. In the context of HIV/AIDS intervention among FSWs, advocacy means systematically enabling key players to understand the core social, economic, political, legal, and sexuality issues linked with FSWs' lives and to shape policy accordingly.

Advocacy requires continuous sensitisation, negotiation, and persuasion with policy makers and opinion builders at all levels. Strong support from various spheres of society is necessary to address and influence existing social and legal norms and policies, which have a negative impact on FSWs' lives and enhance their vulnerability. Advocacy helps to create a broader alliance in favour of the rights of FSWs and the social and legal recognition of the sex work profession.

Creating an enabling environment for the community is one of the fundamental prerequisites of community mobilisation. Advocacy activities help to create this environment. In community mobilisation, FSWs spearhead the advocacy activities. Because of their in-depth understanding of their own lives, the community members themselves can carry out advocacy activities more effectively than outside "experts".

This session focuses on how FSWs can take on advocacy activities by themselves.

Step 1

Discuss why advocacy is needed.

- To convince different bodies/individuals about the rights and demands of FSWs
- To convince policy makers and broader society of the importance of an empowerment approach in TIs among FSWs
- To influence policies which exclude FSWs
- Advocacy is an effective means of increasing social acceptance of FSWs and shaping positive public opinion
- To open dialogue to deal with the issues related to social and moral values and practices in connection with sex and sexuality, particularly with reference to HIV epidemic

Step 2

Discuss identification of the key actors towards whom advocacy efforts should be directed.

Category-wise distribution of different agencies with whom FSWs will advocate.

Level	Functions	Decision Making Bodies/Individuals
Government	State Legislatures/ Ministers	Ministry of Health
		Ministry of Social Welfare
		Ministry of Law
		Ministry of Labour
		Ministry of Panchayat
		Ministry of Home Affairs
		Ministry of Information
		Ministry of Cooperatives
	Implementers	Secretariat
		Directorate
		District level health officers
		Law enforcement officials
Opinion leaders	Political personnel	Local elected representatives: councilors, municipality chairpersons, etc.
		Other political party members at local level
		State elected persons: MLAs
		Other political party members at State level
NGOs		Working in the field of HIV/AIDS
		Working in the field of health
		Working in the field of human rights
		Working in the field of childrens' rights
		Working in the field of women's rights
Media		Mass media
		Other media planners and publishers
Donor agencies		
Trade unions		
Others		Intellectuals, influential persons, democratic fronts, research and academic organisations, religious leaders
Autonomous body		Women's Commission, Bar Council
Corporate house		

Discuss and identify different groups for the respective District and State with whom advocacy could be carried out.

Step 3

Discuss how to carry out advocacy programmes for the different groups that can be organised by community members. Discuss the specific and distinct strategies for advocacy for each set of key players.

- Preparation of materials documenting real situation of FSWs' lives, their demands and endeavours undertaken by FSW collective
- Team building among FSWs for advocacy
- Capacity building for advocacy
- Organise meetings, seminars, workshops with key actors
- Invite key actors to programmes organised by FSWs
- Organise rallies, campaigns, street corners, signature campaigns, circulation of pamphlets, leaflets
- Organise press meet
- Involve FSWs in various activities against social injustices to enhance their social acceptance
- Participate in different forums to enhance interaction with key actors

Time: 3 hrs

Expected Outcomes

- Participants understand the need for advocacy
- Participants can identify groups and individuals with whom advocacy should be done
- Participants understand some strategies for advocacy

Section XIII Community Mobilisation

With limited clinical scope available to control HIV, different locally appropriate community mobilisation models are adopted to contain the spread of HIV in a concentrated epidemic situation. From an HIV intervention perspective, community mobilisation eases the process of accessing hard-to-reach communities. When the community is mobilised to take up intervention, it becomes easier to reach out to socially isolated and geographically dispersed populations.

Empowerment through community mobilisation enhances FSWs' control over their health and lives by challenging the status quo within the sex trade, which in its present form has a sizable negative impact in these areas. PEs acting as "community mobilisers" instil confidence among their colleagues and motivate them. When the latent desires and demands of FSWs are collectivised, they can challenge the social system and barriers which control their lives. As a result of community mobilisation, an FSW collective may be formed. The collective aims for the all-round development of the FSW community and is ultimately responsible for managing health and social intervention programmes. The accountability of the FSW collective towards its own community members ensures optimum quality of service delivery.

HIV interventions working among the FSW community must address the socio-economic, cultural, and political issues which determine the life and behaviour of the community. Broader social perspectives bearing upon health emerge as an important dimension in the programme when community members take the initiative to decide the focal areas of a health-related intervention.

A mobilised community speaks more on the issue of social change. As a consequence, the entire community may join the movement to bring about changes within their own community and in wider society. Even though some community members may lack drive or offer resistance, they are usually pulled in by the collective spirit of majority and thus join the process of mobilisation. Through this process, healthy behavioural norms and practices can be established, and the ambience to sustain the desired behaviour is created.

Step 1

Talk about different types of community participation in programmes. Show the diagram below to explain that participation can vary at different time periods and with different community groups.

Level of Community Control	Type of Participation	Level of Sustainability
High ↑	**Self Mobilisation** – Affected communities start action without outside help	High ↑
	**Joint Decision Making** – Affected communities and organisation make decision together on a equal basis	
	**Functional Participation** – Affected communities are invited to participate at a particular stage of action to fulfill a particular purpose	
	**Participation for Material Incentives** – Affected communities participate in an activity only because they need the material benefit of doing so, e.g. money	
	**Consultation** – Affected communities are asked about an activity by an organisation, but their views may or may not have any influence on it	
Low	**Information Giving** – People are simply informed that an activity will take place and have no say on activity design or management	Low

Ask participants to give examples from their experience as you explain different types of participation. Explain the link between level of community control and sustainability, and explain how different types of participation bring in different levels of community control, thereby affecting sustainability.

Step 2

- Divide the participants into groups of 5–6 participants. If project staff are present, they should form a separate group or groups from the PEs.
- Ask the groups to discuss and analyse what type of participation their programme practises, with valid reasons and examples.
- After the analysis is over, ask each group to present their discussion points. Always give other groups (non-presenting groups) the chance to clarify their doubts.

- As a facilitator, try to analyse the difference of opinion/perspective (if any) between different groups, especially project staff and PEs. Try and highlight these differences and ensure that the community gets a voice. Explore in-depth with the community group the reasons for their opinions and perspectives and ensure that the other groups understand the same and use these perspectives in action planning.

Step 3

Brainstorm ideas among all participants to develop a plan of action to ensure that their programme practices the first two types of participation – Self Mobilisation and Joint Decision Making. Summarise the key points, write on a chart and display the same.

Step 4

Discuss need for collectivisation and formation of self-help groups. Discuss how FSW collectives can help improve their condition.

- If we unite in a collective we will feel more confident
- We can protest against all sorts of abuses and injustices inflicted on us by various power brokers
- We will raise our voices and demands on various issues pertinent to our life
- We can ensure safe sex and our right to say "no"
- We can protect our health and incomes
- We will be able to prevent the entry of minors and unwilling women to sex trade

Step 5

Discuss how to develop an action plan for the formation and functioning of a collective.

- Selection of executive body, secretary, president, office bearers and spokespersons
- Registration of the collective
- Fund mobilisation
- Administrative aspects
- Documentation of activities

Step 6

Discuss proposed activities to be undertaken by the FSW collective.

- Health care services for the FSW community
- Endeavour to create more economic opportunities and financial security
- Initiative for education and proper upbringing of children of FSWs
- Arrangement for alternative occupations for FSWs who leave the profession
- Undertake need-based programmes for the FSW community

- Undertake large-scale programmes to make the general public aware of the problems of FSWs
- Fight for more secure legal status
- Undertake advocacy programmes with power brokers, opinion leaders and policy makers for legal and social recognition
- Provide legal support to FSWs
- Care and support programme for HIV positive persons and their families
- Initiative to prevent forcible entry of unwilling women and minor girls to the sex trade
- Protest against all forms of oppression
- Coordinate local struggles at micro level and build collective network at national level

Time: 3.5 hrs.

Expected Outcomes

- Participants understand the different types of possible community involvement in a programme, and the varying levels of control these give the community
- Participants understand the need for collectivisation and formation of self help groups
- Participants develop an action plan for the functioning of the collective
- Participants design a plan of activities for the FSW collective

Section XIV Evaluation Tool

A very simple evaluation exercise can be carried out after completion of the in-house training programme. It will help to improve the training design.

Evaluation of the training programme may be done with the following questions:

1. To what extent did you like this training? (Not much, To some extent, Very much)
2. Do you think this training will help you to perform better?
3. Which topic did you like the most?
4. Which topic didn't you like much?
5. To what extent was the choice of resource persons for the sessions appropriate? (Not appropriate at all, To some extent appropriate, Quite appropriate)
6. Please suggest any changes that you would like to make in topic selection.
7. Please list five points that you have learnt from this training programme.
8. Was there any memorable experience during this training?
9. What suggestions do you have to improve the training programme?

ANNEXURE 4

Peer Progression

Type	Definition/ Selection Criteria	Selection Process	Role	Remuneration	Possible Next Step - (Career Path)	Possible Capacity-Building Inputs	Performance Indicators for Consideration for Next Level (Qualitative aspects are more important than quantitative.)
**Community Member actively supporting the TI**	n Engaged in sex work<br>n Operates from specific geographic area<br>n Self-identifies as a sex worker among other FSWs (if not among the broader public)	n Recommendation by group of FSWs operating from a locality or type of operating system, e.g. street-based	n Participates in the process of the project<br>n Selects PEs<br>n Supports PEs in fulfilling their responsibilities<br>n Flags issues	n No remuneration offered	n Active member of the local group (Guide)<br>n PE<br>n Member of CBO<br>n Member of SHG<br>n Member of HRG Committees	n Orientation to the project<br>n IPC for safe sex practices<br>n Discussions on rights of FSWs<br>n Build advocacy skills	n Participates actively in project activities for at least three months<br>n Articulates community needs in meetings<br>n Demand for health services, condoms increases<br>n Responds to the common cause (e.g. intervenes in case of violence against a FSW, helps other FSW access services)

Type	Definition/ Selection Criteria	Selection Process	Role	Remuneration	Possible Next Step (Career Path)	Possible Capacity-Building Inputs	Performance Indicators for Consideration for Next Level (Qualitative aspects are more important than quantitative.)
**Active member of the group (Guide)**	• Supports all the activities of the project locally<br>• Comparatively long experience in the community<br>• Commands respect	• Consultation with community members	• Guides community members and PEs on critical issues<br>• Motivates community members to participate in the project process<br>• Mediates in local conflict resolution	• No remuneration offered except TA and nominal compensation for wage loss	• PE	• Community mobilisation skills<br>• Opportunities to participate in formal and informal District level activities<br>• Develops understanding of issues and structures pertaining to FSWs<br>• Develops advocacy skills<br>• Develops crisis management skills	• Continues to associate with the project for six months<br>• Motivates five community members to participate<br>• Is not burdened with self- or social stigmatisation

Type	Definition/ Selection Criteria	Selection Process	Role	Remuneration	Possible Next Step (Career Path)	Possible Capacity-Building Inputs	Performance Indicators for Consideration for Next Level (Qualitative aspects are more important than quantitative.)
**Peer Educator (PE)**	n Selected by the community as representative<br>n Understands community issues<br>n Good relationship with FSWs, communica-tion skills, respect for others<br>n Expresses interest in representing community	n Through internal consultation/ election by community members	n Link between the community and project<br>n Represents and addresses community grievances, problems and needs<br>n Attends PE meetings and workshops organised by other partners<br>n Participates in decision-making on the processes of projects<br>n Delivers services to HRGs	n As per NACO guidelines	n Committee member<br>n ORW<br>n Project Manager	n CLSI perspspective<br>n Rights issues<br>n Skills development in leadership, communication<br>n Opportunities to participate in formal and informal District- level activities<br>n Dealing with authorities<br>n Conflict resolution/ advocay	n Attends most PE meetings<br>n Understands CLSI approach and can communicate it to peers<br>n Brings community issues forward for discussion<br>n Leads/mobilises community members in crisis situations<br>n Confidently interacts with authorities

Type	Definition/ Selection Criteria	Selection Process	Role	Remuneration	Possible Next Step (Career Path)	Possible Capacity-Building Inputs	Performance Indicators for Consideration for Next Level (Qualitative aspects are more important than quantitative.)
**Committee Member**	n Member of the PE group who serves on committee	n By election for a period of one or two years	n Plans, supervises and guides activities of her committee, e.g. DIC, STI clinic, etc.<br>n Facilitates formation of local group/ CBO<br>n Represents PEs/ community members in meetings and workshops and gives them feedback<br>n Addresses barries at peripheral level<br>n Analyses, prioritises and resolves issues	n No remuneration		n Management skills<br>n Assessment of project activities<br>n Advocacy skills	n Identifies gaps in the programme<br>n Assesses activities/ provides input<br>n Problem-solving

ANNEXURE 5

Peer Led Outreach and Planning

CONTENTS

Section I **Overview of Outreach Planning for Peer Educators**

Benefits of Outreach Planning

Outreach Planning in the Organisational Context

Elements of the Outreach Plan

Section II **Outreach Planning Activities**

Process 1 SPOT ANALYSIS

Process 2.1 CONTACT MAPPING (Part 1)

Process 2.2 CONTACT MAPPING (Part 2)

Process 3 NETWORKS

Process 4 OPPORTUNITY GAPS ANALYSIS

Process 5 PARTICIPATORY SITE LOAD MAPPING

Process 6 SEASONALITY DIAGRAMMING

Process 7 FORCE FIELD ANALYSIS

Process 8 PREFERENCE RANKING

Process 9 CONDOM ACCESSIBILITY AND AVAILABILITY MAPPING

Process 10 PEER MAPS

Process 11 SEX WORK TYPOLOGY-WISE OUTREACH PLANNING

Section III **Tools for Monitoring Project Outreach Planning**

PE Daily Activity Report

Individual Tracking Sheet

Outcomes of Outreach to FSWs: The “Minimum Package”

Section I Overview of Outreach Planning for Peer Educators

Outreach planning is a tool that facilitates a peer educator's individual-level planning and follow-up of prevention service uptake, based on individual risk and vulnerability profiles of FSWs and their partners.

Outreach planning at each site is done by PEs. An outreach plan gives a visual picture of the site that a PE is managing. It helps the PE to understand the extent to which programme services have reached the FSWs and to identify and monitor problem areas.

Benefits of Outreach Planning

- **Defined area of operation for PE –** duplication of effort and diffusion of responsibility is avoided when a site is demarcated and responsibility for that site rests with an individual PE.
- **Repeat visits for monthly screening –** The PE is able to monitor clinic visits for monthly screening of the FSWs in the given site.
- **Individual Tracking –** The PE can track how many FSWs are being reached during a given month for various services (clinic/camp attendance, one-to-one sessions, contacts, group sessions, and condom distribution).
- **PE able to collect, analyse and act upon data** – Using the PE daily activity report, the PE is able to generate data and use it to provide minimum services to all FSWs in her site.
- **PE becomes the site manager –** PEs decide and budget for activities to be conducted in their site and take responsibility to ensure service provision to all FSWs in their site.
- **Community ownership –** By addressing felt needs of the community and encouraging active involvement and decision making by the FSWs in all aspects of the programme, a sense of belonging and ownership is cultivated.
- **Shift from delivering services (push) to meeting community's demand for services (pull)** – Ownership by the community generates demand for services. The project services will be community-driven rather than IP-driven.

Outreach Planning in the Organisational Context

To ensure effective implementation of outreach planning, a particular flow system to manage the outreach activities should be put in place, with defined responsibilities for each member. Following is the structure for a typical outreach worker's area:

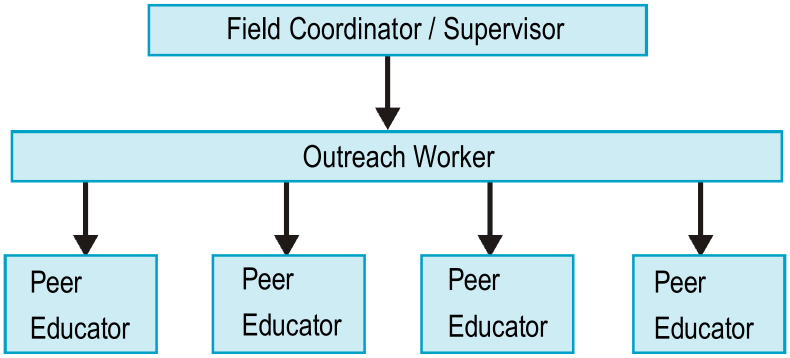

Through the outreach planning exercises, PEs plan their outreach services, including health camps, events, communication sessions, condom distribution and crisis management for the FSWs in their zone. As managers, these PEs monitor their own performance and the delivery of monthly services in consultation with project staff to ensure that the minimum package of health, communication and HIV prevention services reaches all FSWs in their respective zone. This approach has demonstrated that FSWs from low literate and economically challenged backgrounds have the capacity to take up various challenging tasks including managing HIV/STI prevention services.

Elements of the Outreach Plan

A PE creates an outreach plan for her own site and updates and analyses it every month. The essential elements of an outreach plan include:

- Pictorial depiction of the site
- Number of registered FSWs in the site
- Number of new and dropout FSWs
- Number of FSWs accessing services
- Number of FSWs who are members of the NGO/CBO
- Key stakeholders
- Location of condom depots, clinic and health camp areas and location of other relevant local resources

Section II Outreach Planning Processes

Outreach planning is a participatory and interactive process. Following are a set of processes that can be facilitated by outreach workers to help PEs create their own outreach plan. The processes are presented below in a training format, i.e. the tool is designed for outreach workers to train a group of PEs, who will then be able to repeat the processes for themselves as they update and revise their outreach plans.

Process 1 SPOT ANALYSIS

Aim: To help participants compile information collected during urban situation and needs assessment related to each high risk spot/site in their respective project areas to facilitate planning.

Description: Participants, through group work, will compile spot-wise information for planning.

Suggested Teaching Method: Large group discussion.

Materials/Preparation Required: Spot-wise information collected in urban SNA, chart paper, pens, and Handout I (*Planning Outreach for Sex Work Interventions*).

Duration: 120 minutes

Process:

1. Begin the session by asking participants what they learned during the urban situation and needs assessment process. Allot time to share key findings.
2. Clarify the importance and need for outreach planning with respect to HIV prevention programmes. Use the following reasoning:
   - In a programme such as ours, a spot is the smallest geographic location for intervention, and it is important to plan for each and every spot at the *taluk* level. Therefore, outreach plans are developed for the following reasons:
     - Each spot is different, therefore plans have to be spot specific
     - Other characteristics such as client volume and typology of sex work have to be factored into planning
     - Spot-wise planning should facilitate outreach to maximum number of FSWs
3. Ask participants what information they require about FSWs operating in a spot that would help them develop a plan for that spot. Make sure the following is included:
   - Volume of clients - high volume (more than 10 clients/week), medium volume (5-9 clients/ week), low volume (less than 4 clients/week)
   - Typology of FSWs - home-based, street-based, brothel-based, lodge-based, *dhaba*-based
   - Age of FSWs - below 20 years, 20-30 years, 30-40 years, above 40 years
   - Time of operation - morning (6am -10am), afternoon (10am -2pm), evening (2pm -8pm) and night (8pm-6am)
   - Frequency of operation - daily, weekly, monthly
4. Ask participants to divide themselves into groups; group size should reflect the taluks they represent in number. Ask each group to identify a well-known spot in their *taluk* and to do the Exercise 1, Spot Analysis.
5. Give participants 45 minutes to do Exercise 1. Make sure peers in the group participate actively.

6. After everyone completes the exercise, ask each group to present their spot analysis. Encourage peers to make this presentation.
7. After each group presents its spot analysis, ask the following questions:
   - What was the process that each group adopted to do this exercise?
   - What is the analysis for the spot?
   - As a result of the analysis, what is the spot plan?
8. Before concluding, stress the following:
   - **Volume of clients** - Planning should ensure that FSWs with higher volume of clients are reached as a priority.
   - **Typology** - Planning should include typology of sex work and needs to be specific to each type. Street-based FSWs can be reached at solicitation points as well as points of service. Outreach workers can work with them directly or can reach them through network operators. On the other hand, for lodge-based FSWs outreach workers have to advocate with lodge owners and work through lodge boys. Lodge-based FSWs can also be reached at the points of service, that is, in the lodges.
   - **Age** - FSWs' needs differ with respect to age, therefore planning should address that.
   - **Time/day of operation** - Understanding the time and day of operation will help plan outreach with respect to those times. For example, there are certain days in a month, like shandy days, when more FSWs come to a particular spot such as a market. During those days of the month, outreach needs to be strengthened. Similarly, evenings and nights may be very busy in certain spots. Hence, the project needs to ensure that outreach is planned during those times of the day.
9. Distribute Handout I, *Planning Outreach for Sex Work Interventions,* to the participants.
10. Inform participants that spot analysis should be done every six months since ground realities may change.
11. Conclude by reminding the participants the importance of including peers and FSWs in planning.

Note: During this workshop, analysis of only one spot/group can be done due to time constraints. Make sure that, by end of the day, participants plan and develop a time line to complete this exercise for all spots. This analysis can be adapted for understanding characteristics of each location, each *taluk* as well as each District.

EXERCISE 1 - SPOT ANALYSIS

District : , **Taluk :** , **Location :** , **Spot :** , **Date of analysis :**

High Volume	Typology																							
	Street																							
	Age																							
	>20								20-30								30-40							
	Daily				Weekly				Daily				Weekly				Daily				Weekly			
	Time								Time								Time							
	M	A	E	N	M	A	E	N	M	A	E	N	M	A	E	N	M	A	E	N	M	A	E	N

High Volume	Home																							
	Age																							
	>20								20-30								30-40							
	Daily				Weekly				Daily				Weekly				Daily				Weekly			
	Time								Time								Time							
	M	A	E	N	M	A	E	N	M	A	E	N	M	A	E	N	M	A	E	N	M	A	E	N

High Volume	Brothel																							
	Age																							
	>20								20-30								30-40							
	Daily				Weekly				Daily				Weekly				Daily				Weekly			
	Time								Time								Time							
	M	A	E	N	M	A	E	N	M	A	E	N	M	A	E	N	M	A	E	N	M	A	E	N

High Volume	Lodge																							
	Age																							
	>20								20-30								30-40							
	Daily				Weekly				Daily				Weekly				Daily				Weekly			
	Time								Time								Time							
	M	A	E	N	M	A	E	N	M	A	E	N	M	A	E	N	M	A	E	N	M	A	E	N

Medium Volume	Typology																							
	Street																							
	Age																							
	>20								20-30								30-40							
	Daily				Weekly				Daily				Weekly				Daily				Weekly			
	Time								Time								Time							
	M	A	E	N	M	A	E	N	M	A	E	N	M	A	E	N	M	A	E	N	M	A	E	N

Medium Volume	Home																							
	Age																							
	>20								20-30								30-40							
	Daily				Weekly				Daily				Weekly				Daily				Weekly			
	Time								Time								Time							
	M	A	E	N	M	A	E	N	M	A	E	N	M	A	E	N	M	A	E	N	M	A	E	N

Medium Volume	Brothel																							
	Age																							
	>20								20-30								30-40							
	Daily				Weekly				Daily				Weekly				Daily				Weekly			
	Time								Time								Time							
	M	A	E	N	M	A	E	N	M	A	E	N	M	A	E	N	M	A	E	N	M	A	E	N

Medium Volume	Lodge																							
	Age																							
	>20								20-30								30-40							
	Daily				Weekly				Daily				Weekly				Daily				Weekly			
	Time								Time								Time							
	M	A	E	N	M	A	E	N	M	A	E	N	M	A	E	N	M	A	E	N	M	A	E	N

Low Volume	Typology																							
	Street																							
	Age																							
	>20								20-30								30-40							
	Daily				Weekly				Daily				Weekly				Daily				Weekly			
	Time								Time								Time							
	M	A	E	N	M	A	E	N	M	A	E	N	M	A	E	N	M	A	E	N	M	A	E	N

Low Volume	Home																							
	Age																							
	>20								20-30								30-40							
	Daily				Weekly				Daily				Weekly				Daily				Weekly			
	Time								Time								Time							
	M	A	E	N	M	A	E	N	M	A	E	N	M	A	E	N	M	A	E	N	M	A	E	N

Low Volume	Brothel																							
	Age																							
	>20								20-30								30-40							
	Daily				Weekly				Daily				Weekly				Daily				Weekly			
	Time								Time								Time							
	M	A	E	N	M	A	E	N	M	A	E	N	M	A	E	N	M	A	E	N	M	A	E	N

Low Volume	Lodge																							
	Age																							
	>20								20-30								30-40							
	Daily				Weekly				Daily				Weekly				Daily				Weekly			
	Time								Time								Time							
	M	A	E	N	M	A	E	N	M	A	E	N	M	A	E	N	M	A	E	N	M	A	E	N

HANDOUT I: PLANNING OUTREACH FOR SEX WORK INTERVENTIONS

The main objective of outreach, in the HIV intervention context, is to impart behaviour change in targeted populations. The project is attempting to do the following:

- Encourage timely and complete treatment of STIs
- Encourage correct and consistent condom use

The project will work with FSWs and their clients as well as regular partners of FSWs. However, the outreach strategy will differ with respect to FSWs and clients. The objectives of outreach to FSWs are to provide knowledge about STIs/HIV, develop better health seeking behaviour, build skills to negotiate condom use, provide condoms and referrals for services. The objective of outreach to clients is to facilitate safer sexual relationships.

Key elements of outreach with FSWs are as follows:

- **Geographical Coverage** – Outreach needs to be planned for each location/site at which sex work takes place. Each location has its own characteristics/needs, therefore an outreach strategy must address these.
- **Client Volume** – Understanding volume of sex work is important to develop a good outreach strategy. Outreach strategy should ensure that high-volume FSWs (high volume = more than 10 clients/week, medium volume = 5-9 clients/week, low volume = 4 or fewer clients a week) are reached with specific purpose and at specific periods. This is important because, in the context of HIV, FSWs with more clients are most vulnerable and at most risk.
- **Type of Sex** – Type of sex influences risk and vulnerability of FSWs. Anal sex is more risky than oral sex. Therefore, the outreach strategy would also have to address those who are involved in higher risk activities.
- **Typology of Sex Work** – This is very important to understand because outreach strategies differ based on typology of sex work. The outreach strategies for street-based sex work would need to include an intensive peer network in order to reach FSWs both at points of solicitation and points of service. The programme would have to work with madams, owners and lodge boys to reach the brothel- and lodge-based FSWs. Home-based sex work may be hidden and would require different strategies. FSWs in brothels and lodges also normally entertain more clients per week and as a result could be considered high-volume. Outreach strategies need to reflect sex work typologies within the location with a focus on high volume FSWs.
- **Age** – Age of FSWs is also crucial for designing outreach strategies. Interests and needs of FSWs differ depending on age. Vulnerability to risk will differ as a result of age.
- **Time** – It is important to understand time of sex work in the location so that outreach strategies reflect this understanding. For example, male sex workers may normally work in the evening in a specific location, hence outreach to them needs to be planned during that time in those locations. Sex work interventions cannot work on a specific timetable. They have to adapt to field realities.

EXERCISE: SPOT ANALYSIS AT OLD BUS STAND

(DODDABALLAPUR, SURAKSHA NGO)

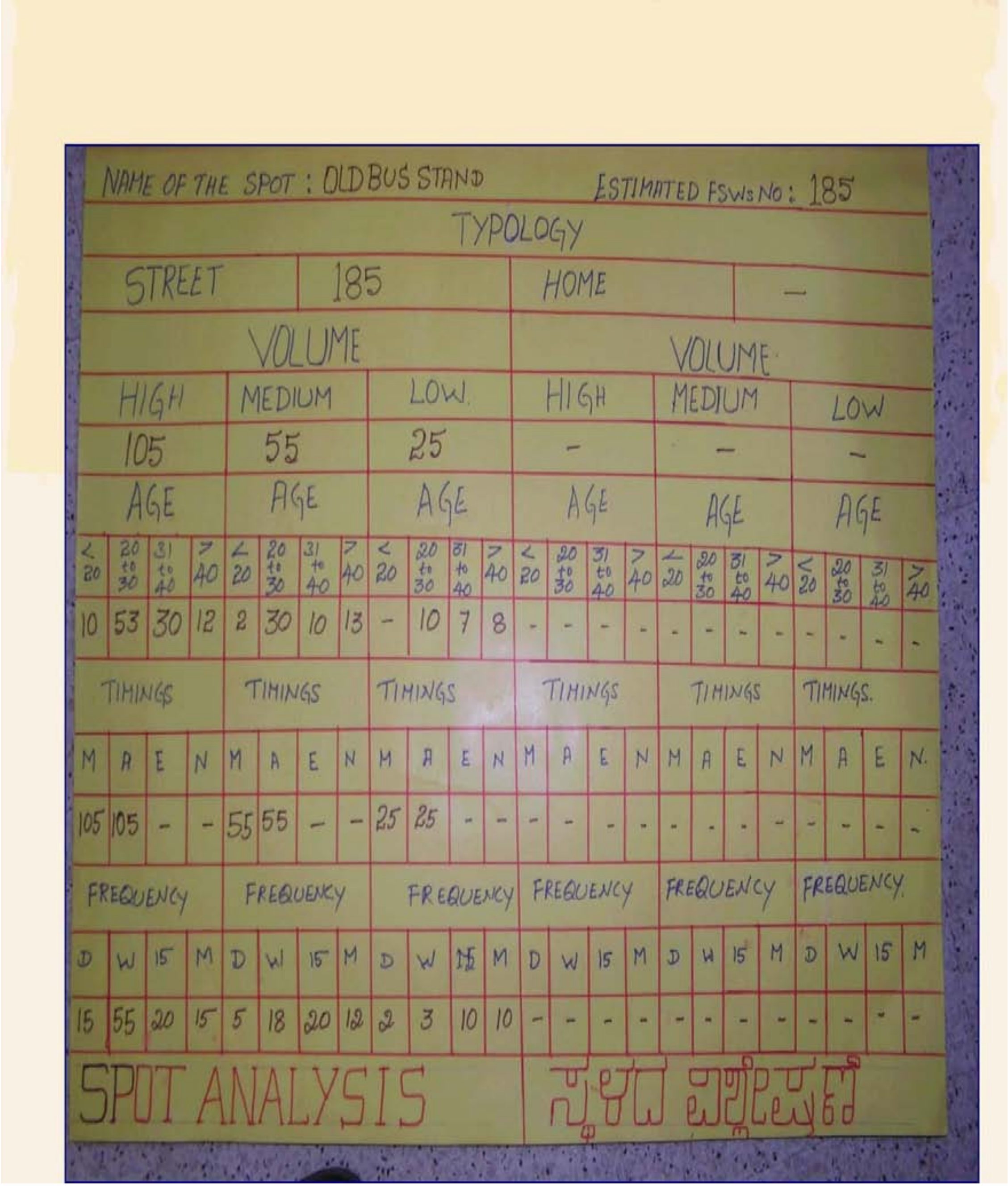

NAME OF THE SPOT : OLD BUS STAND												ESTIMATED FSWs No : 185											
TYPOLOGY																							
STREET				185								HOME						–					
VOLUME												VOLUME											
HIGH				MEDIUM				LOW				HIGH				MEDIUM				LOW			
105				55				25				–				–				–			
AGE				AGE				AGE				AGE				AGE				AGE			
< 20	20 to 30	31 to 40	> 40	< 20	20 to 30	31 to 40	> 40	< 20	20 to 30	31 to 40	> 40	< 20	20 to 30	31 to 40	> 40	< 20	20 to 30	31 to 40	> 40	< 20	20 to 30	31 to 40	> 40
10	53	30	12	2	30	10	13	-	10	7	8	-	-	-	-	-	-	-	-	-	-	-	-
TIMINGS				TIMINGS				TIMINGS				TIMINGS				TIMINGS				TIMINGS.			
M	A	E	N	M	A	E	N	M	A	E	N	M	A	E	N	M	A	E	N	M	A	E	N.
105	105	-	-	55	55	-	-	25	25	-	-	-	-	-	-	-	-	-	-	-	-	-	-
FREQUENCY				FREQUENCY				FREQUENCY				FREQUENCY				FREQUENCY				FREQUENCY			
D	W	15	M	D	W	15	M	D	W	15	M	D	W	15	M	D	W	15	M	D	W	15	M
15	55	20	15	5	18	20	12	2	3	10	10	-	-	-	-	-	-	-	-	-	-	-	-
SPOT ANALYSIS												ಸ್ಥಳದ ವಿಶ್ಲೇಷಣೆ											

Process 2.1 CONTACT MAPPING (Part 1)

Aim: To help participants map contacts they have with FSWs in each spot and plan for outreach based on these contacts.

Description: The participants, through group work, map the contacts they have in each of the spots and analyse needs.

Suggested Teaching Method: Large group discussion.

Materials/Preparation Required: Maps of each town in the *taluk*, chart paper and pens.

Duration: 105 minutes

Process:

1. Begin the session by asking the participants to divide themselves again into *taluk*-wise groups.
2. Ask each group to draw a map of the town and mark all the locations and spots in the map. Write the estimated number of FSWs in each spot.
3. Ask the participants to give a colour code to each of the outreach workers and peers.
4. Using the different colour codes, mark the number of FSWs each outreach worker and peer knows in the spot. For example, assign the colour red to Laxmi, a PE, and mark all her FSW contacts in each spot using red.
5. Allot 30 minutes to complete mapping. Ask each group to present their maps. Encourage the peers to make presentations.
6. After each peer presents, ask the following questions:
   - What does the map show?
   - In which spots are the contacts limited? Why?
   - Where is the outreach not happening?
   - What should be done in those specific locations where FSWs are not reached?
7. Conclude by asking participants if all the contacts that they marked are mutually exclusive, emphasizing the fact that contacts could overlap. For example, PEs may know the same member but count her as two contacts.

Note: Colour-coded maps are easy to understand by all participants, independent of literacy level.

EXERCISE: CONTACT MAPPING DODDABALLAPURA (SURAKSHA-NGO)

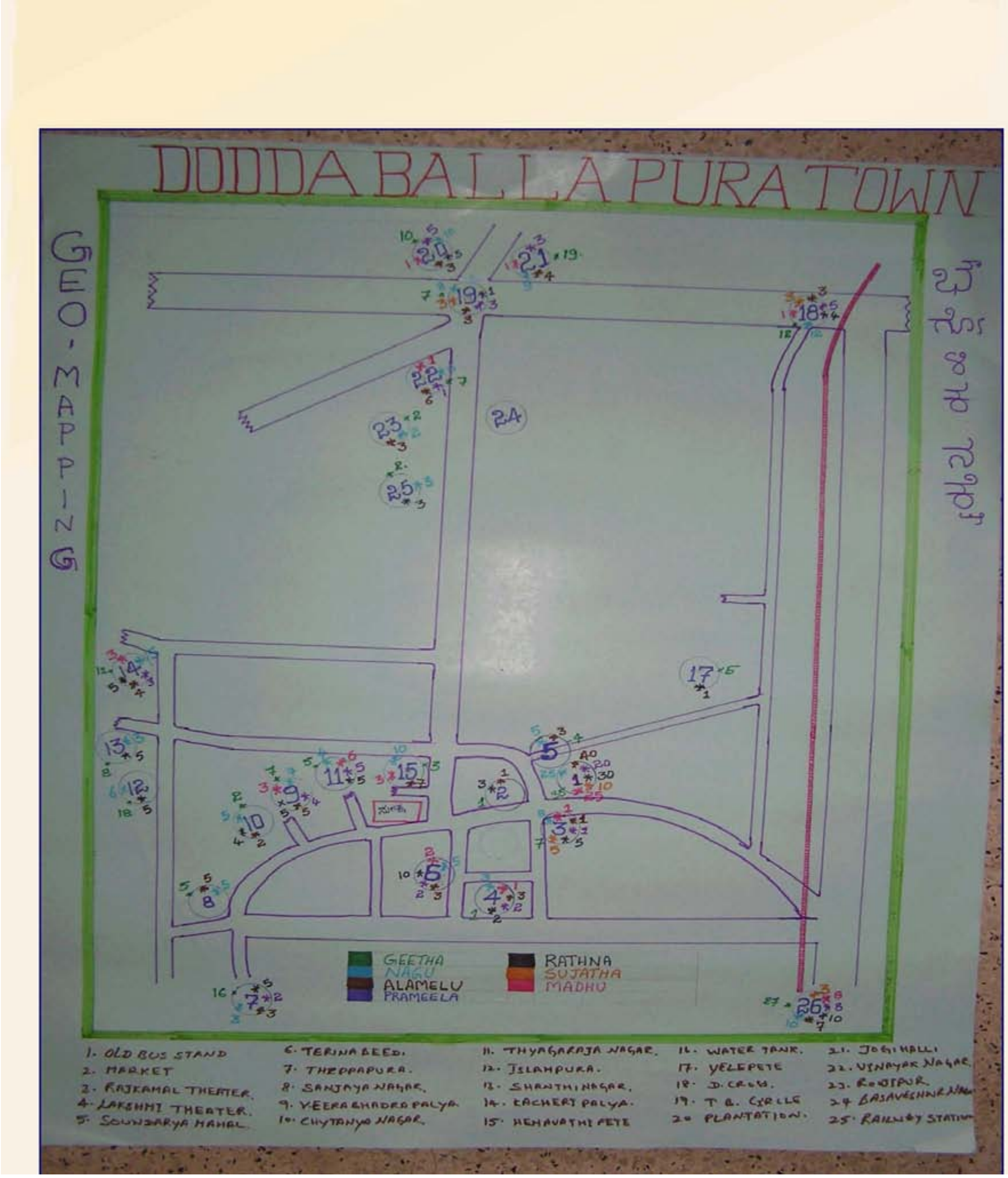

Process 2.2 CONTACT MAPPING (PART 2)

Aim: To help participants understand who the contacts are after mapping them in each spot.

Description: The participants, through group work, list the contacts that they mapped in the previous exercise.

Suggested Teaching Method: Large group discussion.

Materials/Preparation Required: Chart paper and pens.

Duration: 90 minutes

Process:

1. Ask the *taluk* groups to get together and look at their map again.
2. Ask each group to select 3 spots in the map that have the maximum number of contacts.
3. Give the groups 30 minutes and ask them to list names of the contacts in each of the spots as stated in Exercises 2 & 3 (Contact Mapping)
4. Ask each group to answer and record the following:
   - Which contacts does each outreach worker know very well?
   - How many and who are the contacts that are known by more than one outreach worker?
5. After 30 minutes, ask each of the groups to present their group work. Again encourage the peers to make the presentations.
6. Ask participants what they learned and how it will help them in planning outreach. Ensure that the following points are covered:
   - It is important to understand how many contacts we have in each spot and how to increase the number of contacts so that the maximum number of FSWs can be reached.
   - It is important to understand who the contacts are so that we understand whom we are not reaching. That way, we can plan to reach those not yet reached.
   - It is important to understand that outreach workers, especially peers, have contacts in more than one spot.
   - It is important to understand that peers have their own social network, certain FSWs who they are friends with and have influence over.
7. Conclude by informing the groups that both geographic networks and social networks of peers play an important role in planning outreach to FSWs.
8. Also inform the group that mobility is a factor, therefore it is important to conduct Exercise 2 and Exercise 3 every six months. This way the project can ensure that both new and continuing FSWs in each spot are being reached.

Note: Due to workshop time constraints, it may not be possible to conduct this exercise for all the spots. Hence a time line needs to be planned to complete this exercise for all the spots.

EXERCISE 2: CONTACT MAPPING

District: Taluk: Name of Town: Date of exercise:

Estimated number of FSWs in the town:

Contacted Number of FSWs in the town:

Sl. No.	Name of Spot	Peer 1 Name of contacts	Peer 2 Name of contacts	Peer 3 Name of contacts	Peer 4 Name of contacts
1					
2					
3					
4					
5					
6					
7					
8					
Total					

EXERCISE 3: CONTACT MAPPING

District: Taluk: Location: Spot:

Date of exercise:

Estimated number of FSWs in the town:

Contacted Number of FSWs in the town:

Sl. No.	Peer 1 Name of contacts	Peer 2 Name of contacts	Peer 2 Name of contacts	Outreach staff 1 Name of contacts	Outreach staff 2 Name of contacts
1					
2<br>3					
4<br>5<br>6					
7					
8<br>9					
10					
11<br>11<br>13					
14<br>15					
No. of contacts that are known very well					
	# of contacts	# of contacts	# of contacts	# of contacts	# of contacts

Color-code the contacts that are common to more than one list.

EXERCISE: CONTACT MAPPING DODDABHALAPUR
(SURAKSHA-NGO)

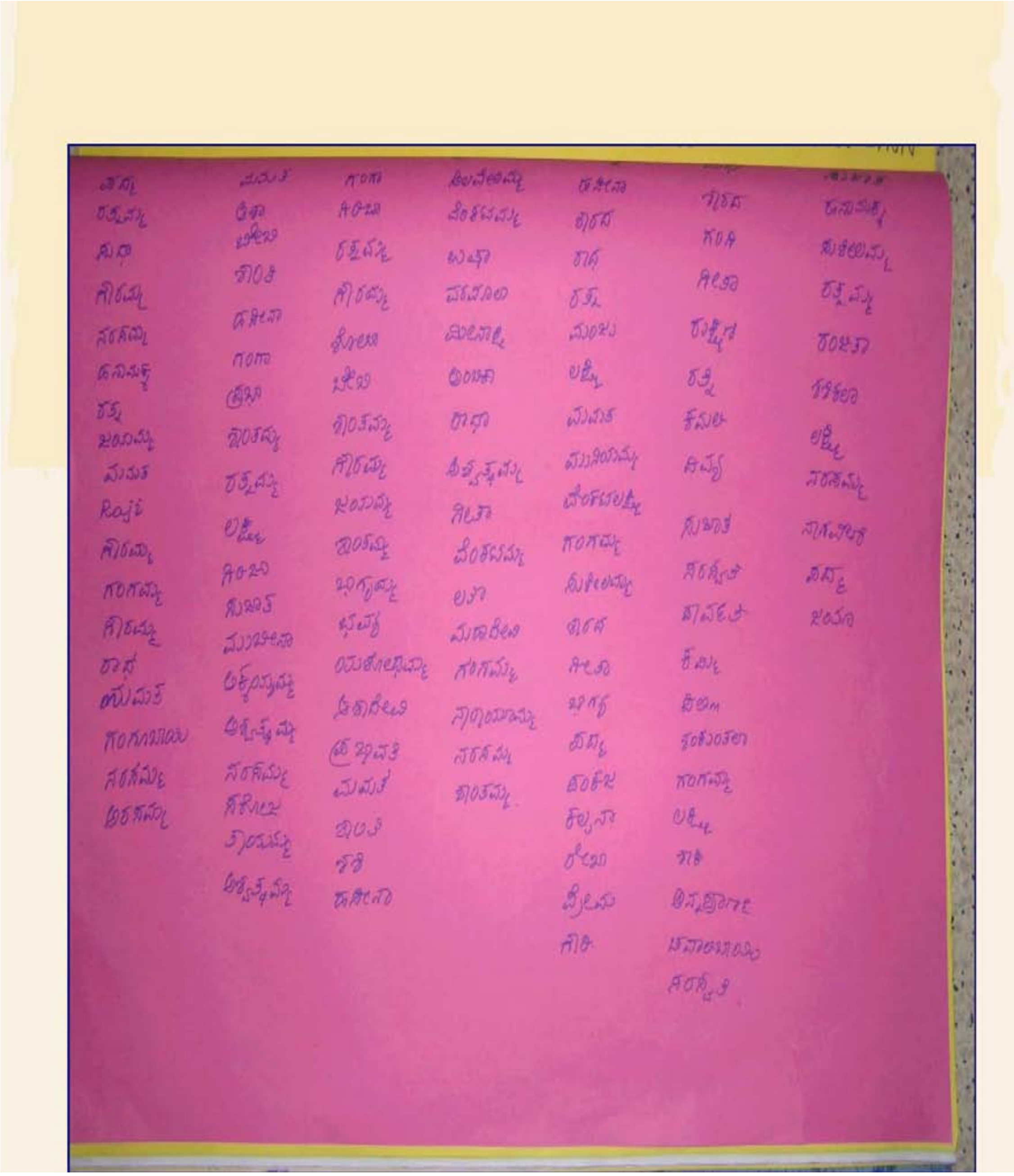

Process 3 NETWORKS

Aim: To help participants understand geographic and social networks of FSWs and advantages and disadvantages associated with both.

Description: The participants, through a debate, discuss the advantages and disadvantages of geographic and social networks and include the same in planning outreach.

Suggested Teaching Method: Debate and discussion.

Materials/Preparation Required: Chart paper and pens, Handout II

Duration: 90 minutes

Process:

1. Deliver a mini-lecture on FSW networks. Clarify that FSWs can have contacts in a particular geographical location, a particular social circuit, and also with network operators. It is important to understand the networks because both "frequency of meeting" and "peer influence" have a great impact on the FSWs. Hence while selecting peers it is important to ensure that peers are selected from all networks so that the project can maximize reach.
2. Distribute Handout II (*Geographical and Social Networking*). Ask one participant to read the case study out loud to the group. Ask them to stop where the case study ends. Make sure that they do not read the definitions.
3. Once you ensure that every participant has understood the case study, divide the participants into two groups using a group-forming energiser/icebreaker.
4. Ask the groups to discuss the following:
   - Group 1 - Advantages of selecting peers from a particular geographical location and disadvantages of selecting peers from social circuit.
   - Group 2 - Advantages of selecting peers from social circuit and disadvantages of selecting peers from within a particular geographical location.
5. Give the participants 30 minutes to prepare for the debate.
6. Appoint a referee for the debate and allot 10 minutes to each of the group to share their viewpoints.
7. Highlight the key advantages and disadvantages of each network and conclude that both networks are important to consider in selecting peers. Peer selection depends on the situation, and a combination of both strategies may need to be used. In the early stage of the project, a social network may be more efficient even though it is time consuming. Once all the social contacts of each peer/ volunteer are introduced to the project and rapport is built by each peer with others in her group, the project should move to geographic networks. At times, depending on the situation, the project may have to use geo-social networks in order to ensure effective outreach. The project should decide which one to adapt and determine this based on the project needs and reach at that time.
8. Conclude by reading out the definitions of geographic and social networking from the handout.
9. Announce that both teams have worked hard and both the teams have won. Distribute small prizes (if possible) to all the team members.

HANDOUT II: GEOGRAPHICAL AND SOCIAL NETWORKS

Case Study of Rani

Rani is a FSW who has been operating in Bangalore City for past 7 years. She is 26 years old. In her early years, she used to operate from the bus stand with her friend Rathna. Over a period of time she developed a friendship with 15 other FSWs who operate from the same area. She comes from her village every day. She arrives at 11am and work until 6pm.

She knows that there are around 100 to 150 women who operate at the bus stand. Some of them operate in the morning hours (6am to 10am), some in the evening (6pm to 10pm) and some in the night (10pm to 5am). Rani has seen many of them but not all are her close friends. She knows about 70 women who operate at the bus stand at the same time as her (11am to 6pm). Of the women who operate at the same time as her, 15 are her close friends and 30 are her acquaintances.

In last 7 years of working in Bangalore City, Rani has moved to different locations in the city, such as the railway station and the market, to solicit clients due to various reasons. Over the years, Rani has operated in the top 10 locations within the city. She has developed close friendships with 80 FSWs in those locations (including 30 women in the bus stand). She also knows 140 other FSWs who operate in those locations regularly.

The SNA and spot analysis estimates 1,500 FSWs in those 10 locations. These women are known to operate at different times. The project has developed a good rapport with Rani. Furthermore, Rani is willing to work as a PE since she understands that STI/HIV is a serious threat to her community, especially to her friends who she loves and is concerned about.

The project staff recognizes that Rani is an asset to the project. They are interested in involving her in the project. The staff has to decide on how to incorporate Rani into the project.

The project has two options:

Option One:

Rani can be given a particular geographical area (1 or more locations) and she has to reach all the FSWs who operate in that area and also identify new FSWs. This would mean that she will have to build rapport with all the FSWs in the assigned location, give them information and condoms and bring them to the clinic.

Option Two:

Rani can be given the responsibility of reaching her close 80 friends on a regular basis whom she knows very well and has good rapport with in 10 different locations within the city.

The Questions:

1. Which option is the most effective and efficient?
2. What are the advantages and disadvantages of each option?

DEFINITIONS

Geo-Networking Concept (Option One)

Geo-networking is defined as networking/reaching FSWs within a fixed geography. Using this concept, a peer educator/community volunteer is given the responsibility of reaching all the FSWs that are operating in a particular geography irrespective of her rapport or relationship with them.

This in practical terms means that the peer has to go and make friends with all the FSWs in the particular spot (geography) irrespective of age, time of operation, etc. For this she may have to work beyond her normal sex work times, make an effort to meet the women or get introduced another way.

Social Networking Concept (Option Two)

Social networking is defined as networking/reaching FSWs within a social circuit. Using this concept, the peer educator/community volunteer is given the responsibility of reaching out to her friends irrespective of a defined geographical area.

This in practical terms may mean that the peer may have to travel to several spots, do her work and also work for the project. The project may have to appoint more than one peer in one spot/geography.

Process 4 OPPORTUNITY GAPS ANALYSIS

Aim: To help participants understand opportunity gaps in each spot, reasons for the same and ways to overcome them.

Description: The participants through group discussions discuss and analyse opportunity gaps in each spot.

Suggested Teaching Method: Group work and discussion.

Materials/Preparation Required: Chart paper and pens, Handout III (*Opportunity Gaps*)

Duration: 120 minutes

Process:

1. Explain to participants that it is very important to understand and analyse periodically what the project has been able to achieve and what it has not been able to achieve. This analysis should be spot-wise since every spot is unique and hence needs a specific outreach plan. Deliver the following mini lecture:

   One of the objectives of the project is efficient outreach to ensure that all FSWs in every spot are reached with information and services. Outreach aims to change the following behaviours of the FSWs:

   - From low/no condom use to correct and consistent condom use
   - From low/no STI treatment to early, timely and complete treatment
   - From poor health-seeking behaviours to regular monthly health check-ups

   Hence to attain this behaviour change, various outreach processes take place in the field. These are as follows:

   - Contact in the field
   - Registration
   - Regular contact
   - STI treatment
   - Follow up
   - Regular health check-up

   However, during these processes in the field there are dropouts, and that is what we call "opportunity gaps". It is important to analyse the reasons for these gaps along with the community to develop an efficient outreach plan which is responsive to the needs of the community.
2. Taking a District into consideration, ask the group to identify the gaps and reasons for those gaps. Make sure to distinguish between external reasons and internal reasons. Along with the participants draw up a plan to overcome these gaps. Encourage the peers to talk about their perspectives on reasons for the gaps and ways to overcome them.
3. Clarify any question the participants may have about the exercise.
4. Divide the participants in to *taluk*-wise groups and ask them to identify one spot in their *taluk* and in groups ask them to do Exercises 4 and 5.
5. Give the groups 45 minutes to complete the exercise. On completion ask them to present their group work.
6. Ask other participants to comment on the action plan. Also give your comments.

7. Distribute Handout III (*Opportunity Gaps*) and ask one participant to read the handout aloud. Clarify doubts if any.
8. Conclude that this analysis needs to be done every six months in every spot to analyse and understand what we are achieving and what we are not and to revise our plans accordingly.

Note: Due to workshop time constraints, it may not be possible to do this exercise for all the spots. Hence a time line needs to be drawn up to complete this exercise in all the spots.

EXERCISE 4: OPPORTUNITY GAPS ANALYSIS - DISTRICT

Activities	Status	Opportunity gaps	Reasons		What should we do?
			Internal	External	
Estimate					
Contact					
Registration					
Regular Contact					
STI treatment					
Follow-up					

EXERCISE 5: OPPORUNIY GAPS ANALYSIS - SPOT

Esimated FSWs in the spot:

Activities	Peer 1	Peer 2	Peer 3	Status	Opportunity gaps	Reasons		What should we do?
						Internal	External	
Contact								
Registration								
Regular contact								
STI Treatment								
Follow-up								
Regular Health Check up								

HANDOUT III: OPPORTUNITY GAPS

Opportunity Gaps are obstacles that impede an individual/community from moving from one level to next level in the behaviour change processes.

The FSW has to undergo different stages/level of the outreach cycle for effective behaviour change to occur. The project should work on removing the obstacles and on creating an environment at every stage/level so that the individual/community can move from one level to the next more easily.

The factors/reasons that cause opportunity gaps may vary from individual to individual in a community. The project should develop systems to assess opportunity gaps at every level by using qualitative/ quantitative information.

Example of Opportunity Gaps

(A spot-wise analysis must be done and an overall analysis for the town must be completed to gain both a spot-wise understanding and overall understanding, since the opportunity gaps may vary from spot to spot.)

**Level 1**		
Estimated FSWs in the project area		218
Opportunity gap (Level 1 - Level 2)	***0***	
**Level 2**		
FSWs who have been contacted at least once by the project -		218
Opportunity gap (Level 2 - Level 3)	***79***	
**Level 3**		
FSWs who have been registered		139
Opportunity gap (Level 3 - Level 4)	***34***	
**Level 4**		
FSWs who are in regular contact with the project		105
Opportunity gap (Level 4 - Level 5)	***47***	
**Level 5**		
FSWs who visited the clinic for STI treatment		58
Opportunity gaps (Level 5 - Level 6)	***12***	
**Level 6**		
FSWs who completed the treatment		46
Opportunity gaps (Level 6 - Level 7)	***46***	
**Level 7**		
FSWs who had regular health check-up		0

See example for details. The reason for opportunity gaps at each level has to be identified and an action plan needs to be developed to overcome these opportunity gaps. The reasons for gaps may be internal factors (where the project has direct control, as in work timing of ORWs and PEs) or external factors (for example, high mobility of FSWs on a daily basis.) The internal factors can be solved immediately so that the quality of input from the project can be strengthened. Proper networking and advocacy with other government and not government organizations can solve most external factors.

DEFINITIONS

**Contact**	Identification of FSW. Purposeful interaction with the FSW.
**Registration with the project**	After building rapport with the FSW, the FSW is registered by filling the registration form. This provides his/her a number and makes it easy for the project to track outreach provided to her. Registration can happen after 1 -8 contacts in the field.
**Regular contact**	A FSW is receiving education regu larly (once every 15 days), over a period of one year or until the FSW is no longer in that location (total 24 interactions a year). FSW is receiving condoms for 90% of her estimated/reported client interaction. Condom distribution is accompanied by demonstration and training in negotiation skills if needed.
**Referral to clinic for STI related services**	Referral is done by outreach workers or peer. Referral should include STI information, condom information and demonstration and distribution of at least four condoms. Address of a clinic should also be shared.<br><br>The doctor provid es syndromic case treatment for STIs. STI treatment includes understanding the symptoms of the FSW, clinical examination, prescription/distribution of drugs to FSW and partner notification/ treatment.<br><br>STI treatment also includes risk assessment and risk reduction counse lling, condom demonstration and distribution. Either the doctor or the counse llor can provide counselling.<br><br>Referral to the clinic needs to be done whenever a FSW ha s a symptom. Every 6 months, the FSW is referred for presumptive treatment.
**Follow up**	FSWs who have been treated in the clinic need to be followed up at home or clinic within one week.
**Regular health check-up**	FSW receiving STI/health care services every three months from the program clinic or through referral doctors (aiming for four check-ups in a year).<br><br>The objective is to promote regular health seeking behavio ur among FSWs. She should be referred every quarter even if she does not have symptoms.

EXAMPLE: OPPORTUNITY GAPS ANALYSIS

Activities	Status	Opportunity Gaps	Reasons		What should we do?
			Internal	External	
Estimate	218				
Contact	218				
Registration	139	79	Lack of rapport with the 79 FSWs	Low volume FSWs<br>Fear of identification<br>Women come to town only once in 15 days	Understand the time when these women come and plan accordingly<br>Build their trust by contacting them through other ex-workers or stakeholders
Regular Contact	105	34	Have not been able to generate interest	Higher mobility of FSWs<br>Few FSWs come only once in a month	Link up with other services in the taluk so that women can be offered varied services<br>Reach women through their social networks
STI Treatment	47	58	Referral clinic is new<br>Clinic is available only on fixed days<br>Lack of trust in the project	No symptoms<br>FSWs drink alcohol	Build trust through peers<br>Inform the FSWs about advantages of check-ups
Follow-Up	12	46	Importance of follow-up not communicated properly<br>Staff did no have clear guidance on follow-up	FSWs are mobile	Provide counselling about follow-up to FSWs along with treatment<br>Motivate doctors to advise follow-up<br>Continuously remind FSWs about clinic day
Regular Health Check-Up	0	46	Communication gap with NGO. This service has not been started		

Process 5 PARTICIPATORY SITE LOAD MAPPING

Aim: To help us understand the gap between estimates of FSWs, the number of unique contacts and the number of regular contacts by studying the FSW load in a day, a week and a month in different sites. Participatory site load maps also give information on potential regular contacts: the potential number of FSWs a *taluk* team can contact in a month.

Description: The participants develop site maps to understand the turnover of FSWs at a given site in a day, week and month and compare the same with the number of unique contacts and the number of regular contacts at these sites.

Materials Required: Charts, pens.

Duration: 120 minutes

Process:

1. Discuss with the participants that in order to reach out to the FSWs it is important to know where, and how many are available on a given day, week and month.
2. Divide the participants, *taluk*-wise and ask them to draw a map of the *taluk* clearly depicting the sex work sites (the sites at which FSWs pick up/solicit their clients) in the *taluk*. Ask the participants to colour-code the sites based on sex work typology such as home-based sites, brothel-based sites, street-based sites, etc.
3. Check that the participants have marked all the sites based on typology. Once all the sites are marked, ask the participants to write down beside the site the number of FSWs who are always available on a normal day.
4. Next ask the participants to write the number of FSWs available at these sites in a week. Check with the participants if there are any specific days in a week when the number of FSWs peaks and reasons for the same, e.g. more FSWs are available on a shandy day.
5. Once the above exercise is done, ask the participants to mark the number of FSWs available in these sites on a monthly basis and also ask if there are specific days in a month where the turnover is high and the reasons for the same, e.g. more FSWs are available on payday.
6. Then ask the participants to add the daily, weekly and monthly turnover in all the sites and draw up a picture of FSW turnover in a *taluk*.
7. Now again ask the participants to compare these figures with their estimate, unique contact and regular contact figures for these sites and analyse in the following way:
   - Are the total FSWs available in these sites/*taluk* more or less than the unique contact and regular contact? Why?
   - Is high weekly and monthly turnover linked with any specific typology of sex work, e.g. is there high turnover seen in mostly street-based sex work? Why?

- Are there specific sites where unique contact and regular contact is less than monthly turnover? Why?
- Which are the sites and typology of sex work that need focused outreach in the *taluk*? Who (outreach team) is responsible for these specific sites? What should they do to improve outreach to ensure higher contacts?

Note: Participatory site load mapping is a visual exercise done along with outreach workers, peers and volunteers. This exercise requires a thorough understanding of the geography of the town/*taluk*.

Example 1.2: Participatory Site Load Mapping

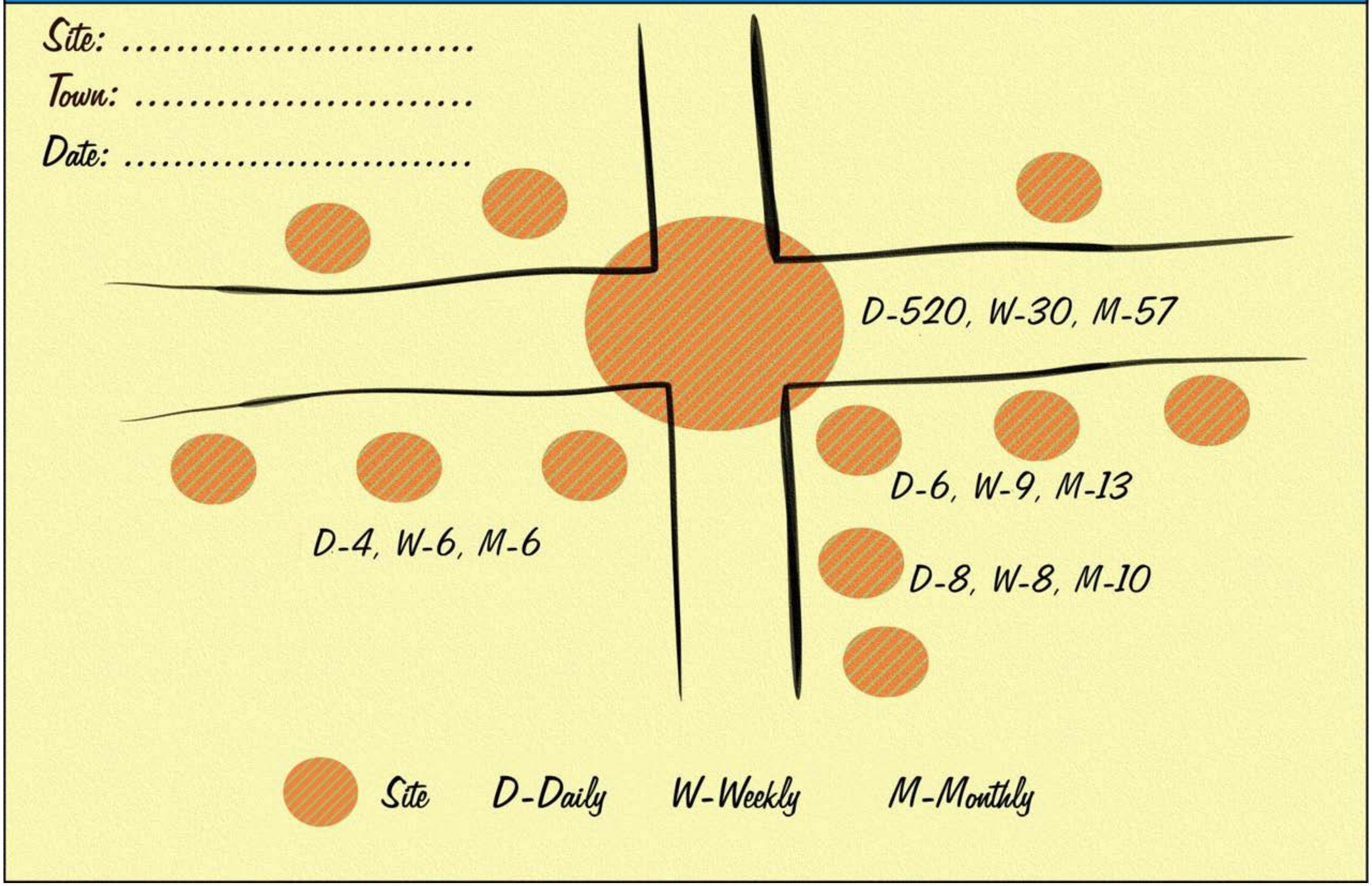

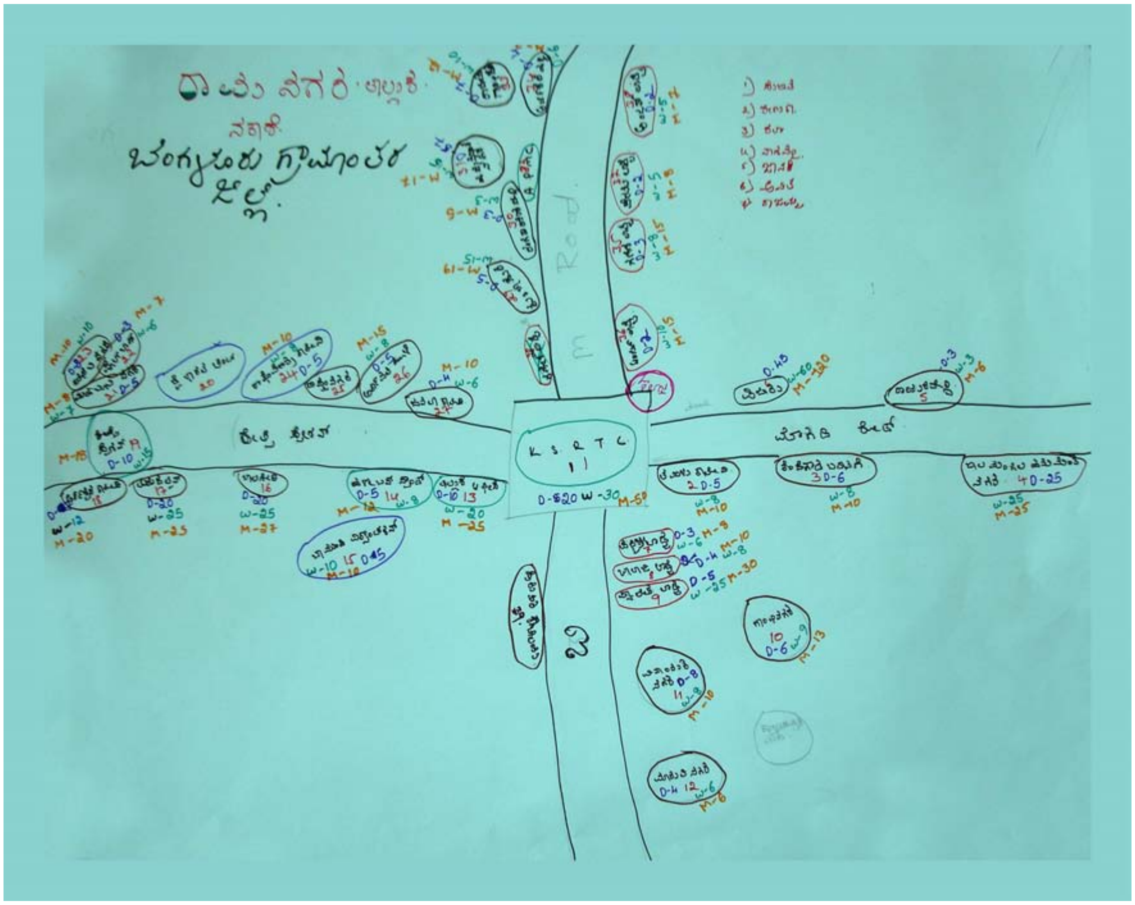

SAMPLE SITE LOAD MAP

Process 6 SEASONALITY DIAGRAMMING

Aim: To understand peaks and troughs of sex work at a given place in a year and its impact on outreach planning.

Description: The participants, through a seasonality map, attempt to understand the peaks and troughs in sex work based on typology in a *taluk* and reasons for the same. They learn to plan outreach based on this seasonal variation.

Materials Required: Pens, chart paper.

Duration: 120 minutes

Process:

1. Inform the participants that in this exercise we will attempt to understand how the sex work scene changes in a year in their town.
2. Divide the participants into *taluk*-wise groups and start by asking them which month of the year the maximum number of FSWs operate in the town. Ask the participants to have a group discussion and finalise the month/s.
3. Next ask them to write the approximate number of FSWs in those high and low months and the reasons for the same.
4. Then identify the next busiest or peak month, the number of FSWs and the reasons. Document results. Similarly continue doing this exercise for all the months in a year.
5. Make sure that the discussions are intensive and all the participants are involved. Make the exercise visual by using chart paper, colour pens, etc.
6. Finally, when the seasonal calendar is complete, verify the results with the participants to ensure that everybody agrees with what the calendar depicts.
7. Ask the group the following questions:
   - During peak months do we find FSWs from other towns coming to our town?
   - Is the peak season specific to our *taluk* or is it valid in other *taluks*, also?
   - In the low season, do the FSWs stop sex work or do they migrate to other towns?
   - How does our outreach plan change based on these seasonal variations?

Note: The seasonal calendar can also be done for a month or even a week to understand the peaks and troughs in a given period. Pay close attention to how the participants understand the different months in a year. Sometimes the participants may be more familiar with seasons in a year or different festivals in a year. In that case ask them to follow that calendar. Ensure that you check the peaks and troughs based on festivals, specific events, etc. A seasonality diagram can be also done to understand seasonal variations in other factors such as STIs or police violence.

Example 1.3: Seasonality Diagram

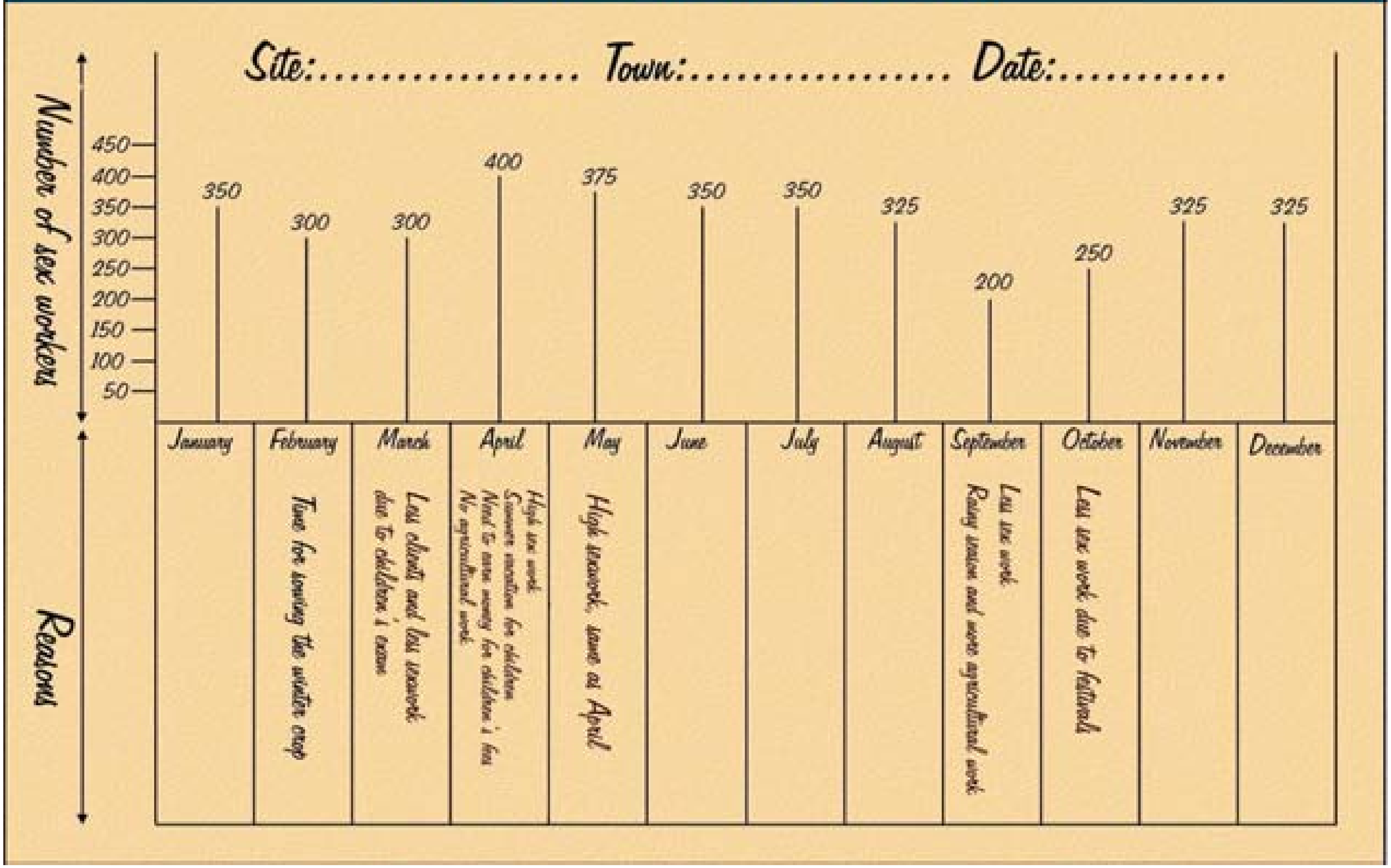

Process 7 FORCE FIELD ANALYSIS

Aim: To understand the reasons for gaps in contact and regular contact, and plan outreach to reduce the gap.

Description: The participants through this exercise analyse the reasons for gaps in contact and regular contact, and develop plans to address these reasons.

Materials Required: Pens, chart paper.

Duration: 120 minutes

Process:

1. Divide the participants into *taluk*-wise groups and ask each group to identify the reasons for the difference between the unique contacts and regular contacts.
2. Ask each group to pictorially depict these reasons in small charts.
3. Ask the participants to rank the reasons in order of priority. Ensure that the participants enter into a lively debate and everyone participates.
4. Once these reasons or constraints are identified ask the participants for ways in which these constraints can be overcome. Ask them to go through each constraining factor and ask the participants to list ways to overcome each of the constraints. Discuss with the participants the various ways listed to overcome constraints and the ways that are easily do-able.
5. Finally compile all results on a chart paper and check with the group for any disagreements.
6. Ask the groups to present their discussions and ask the following questions:
   - Were they aware of these constraints and the ways to overcome them?
   - How will this knowledge help them in planning outreach?

Note: This is a technique to identify and analyse the forces that restrain and facilitate a particular situation, process or outcomes. The assumption is that for a given situation, there will be restraining factors and similarly there will also be factors that help improve the situation. When it comes to finding reasons for opportunity gaps, this exercise can be used at all levels of gaps.

Example 1.4: Force Field Analysis

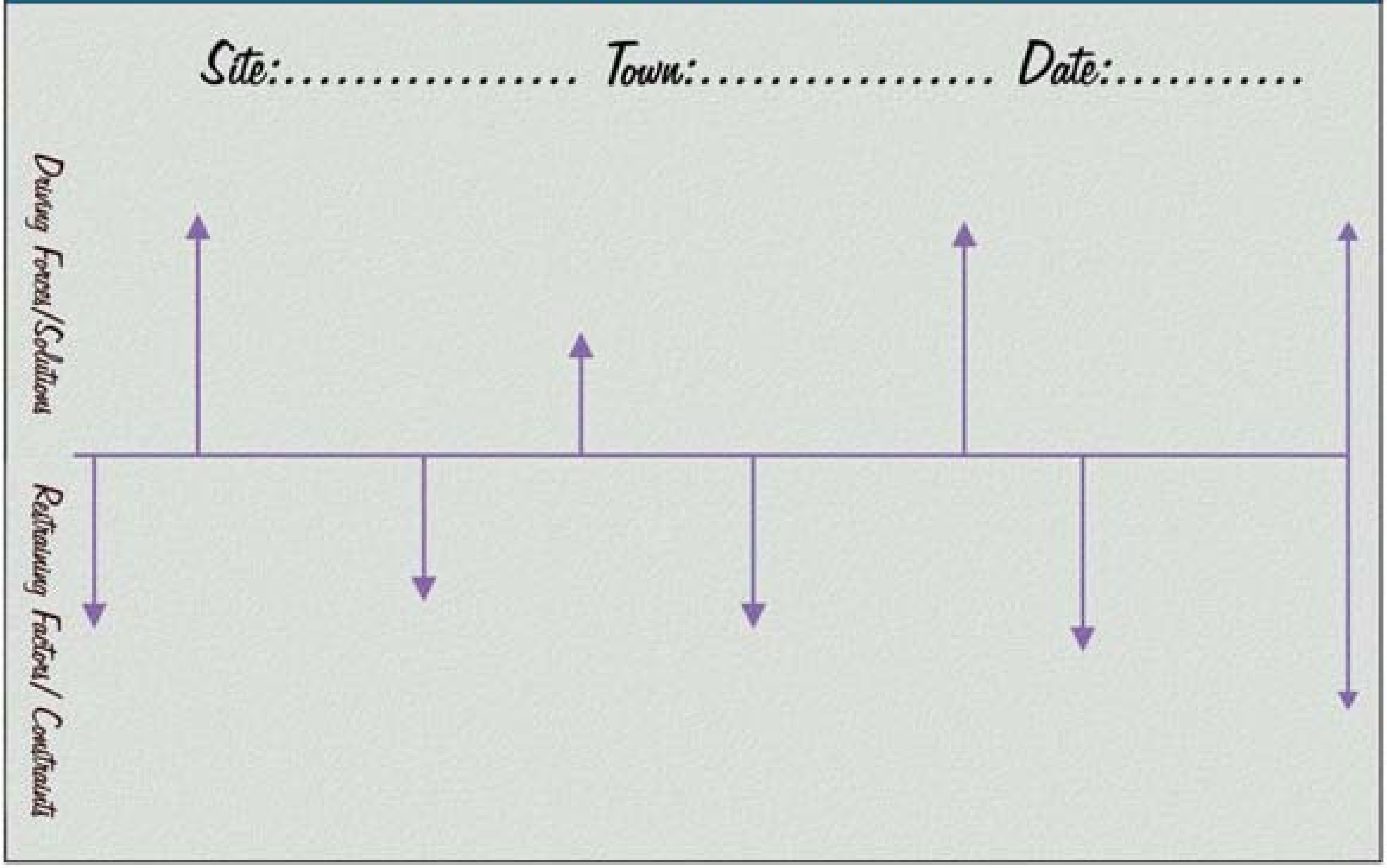

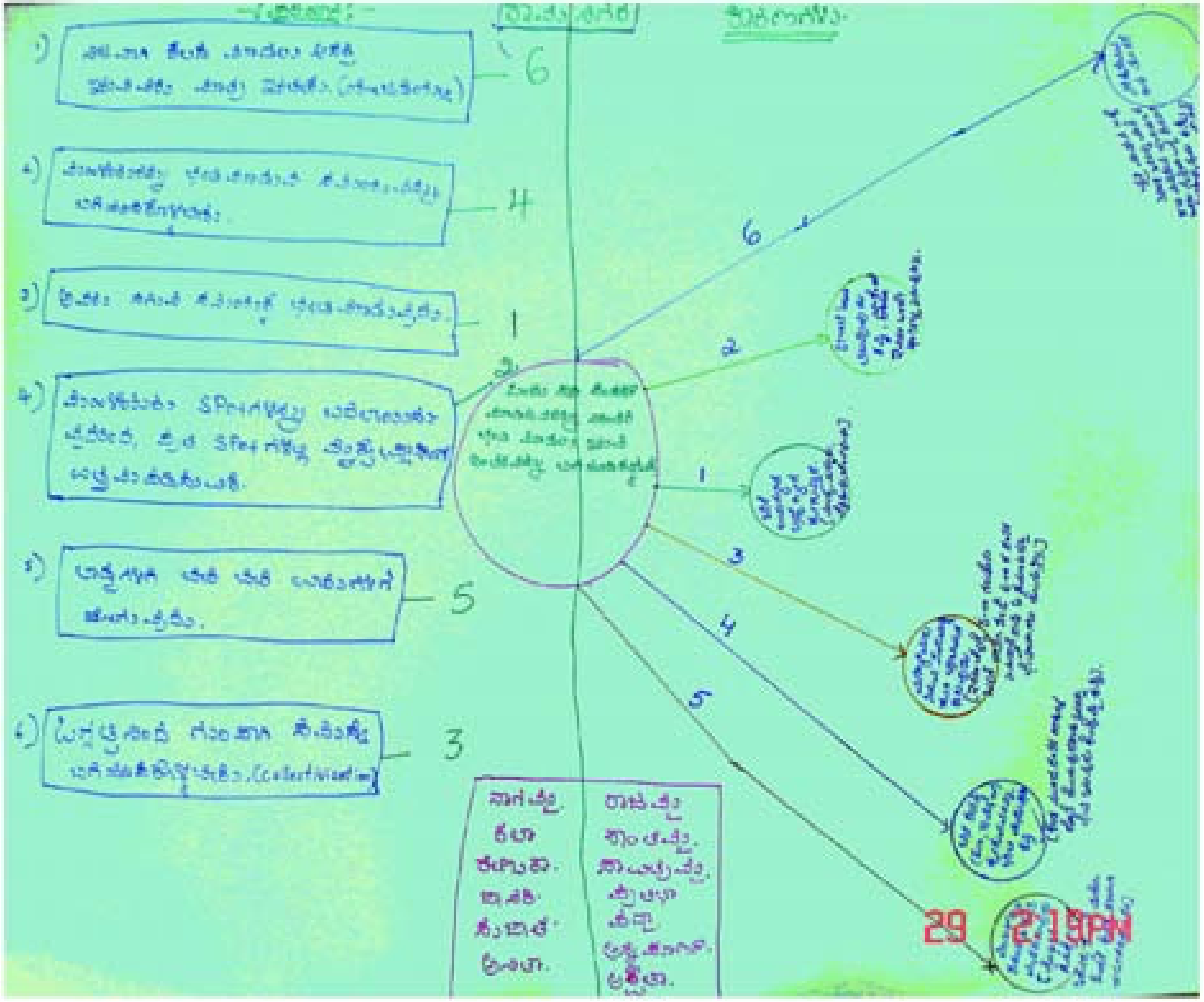

SAMPLE FORCE FIELD ANALYSIS

Process 8 PREFERENCE RANKING

Aim: To identify the reasons for gaps in regular contact and clinic attendance and prioritise the same.

Description: The participants by using the preference ranking tool analyse the reasons for gaps in regular contact and clinic attendance, prioritise the same and make plans to address them.

Materials Required: Chart paper, pens

Duration: 120 minutes

Process:

1. Begin by discussing the general reasons why FSWs do not come to access clinical services.
2. After the initial discussions, ask the participants to list the reasons why FSWs in their town do not access clinical services. Give each of the participants a flash card and ask them to pictorially depict the reasons on the card.
3. Ask the participants to now discuss the reasons in groups, prioritise the same and select the five most important reasons for low clinic attendance.
4. Then ask the participants to do a preference ranking of each of these five reasons and prioritise the most important reason.
5. Ask the participants to make presentations and ask them the following questions:
   - What are the most important reasons for FSWs not coming to the clinic?
   - What are the plans to address these reasons?
   - How would outreach or services change based on this exercise?
6. Conclude by developing an outreach plan to address these priorities.

Note: This exercise can be also done to develop a community/FSW understanding of a good service. We can ask the community/FSW to list the elements of a good service and do a preference ranking to understand their priorities. Compare whether the existing services meet these priorities. If not, then develop a plan to make the existing services better.

Example 1.5: Preference Ranking

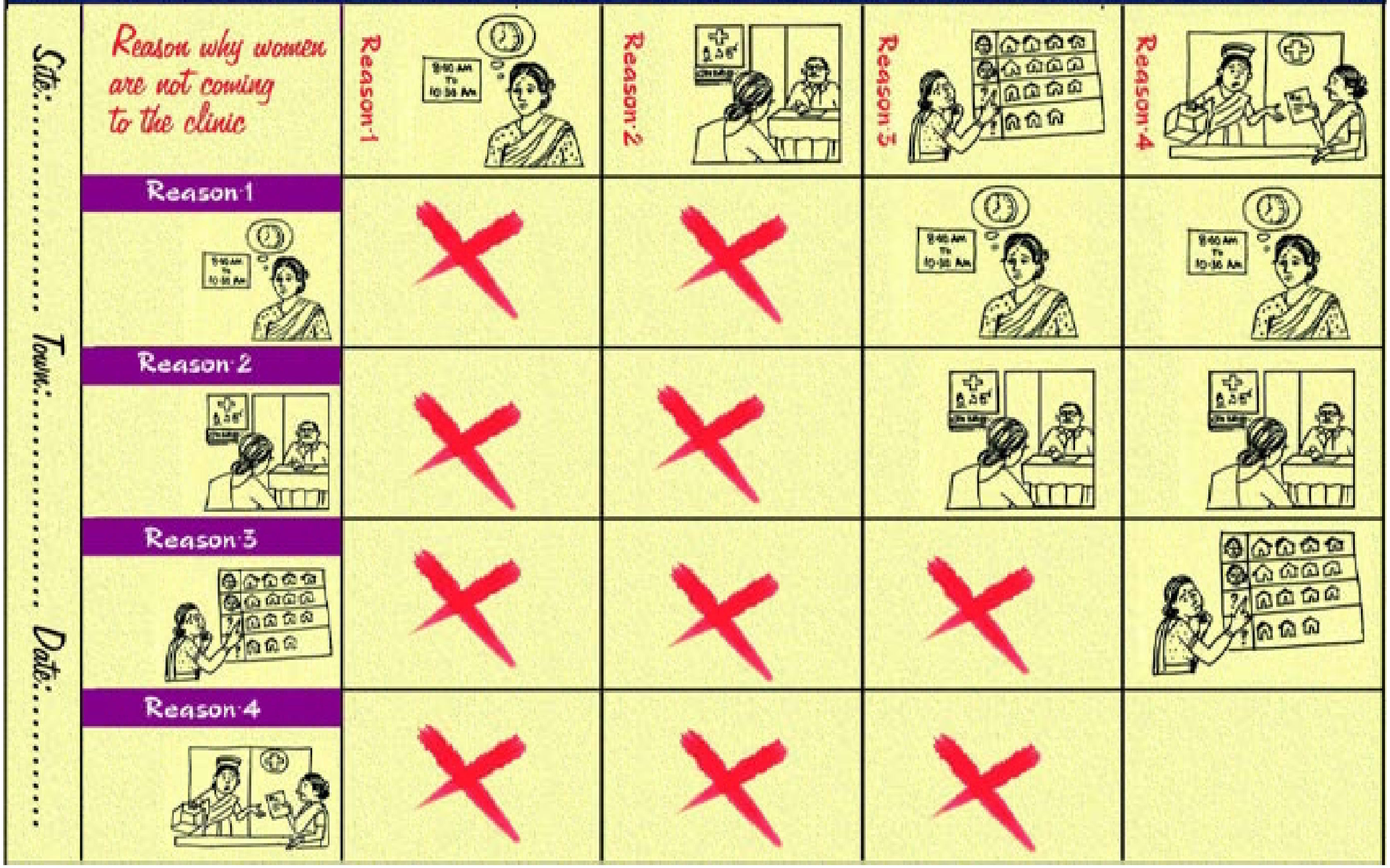

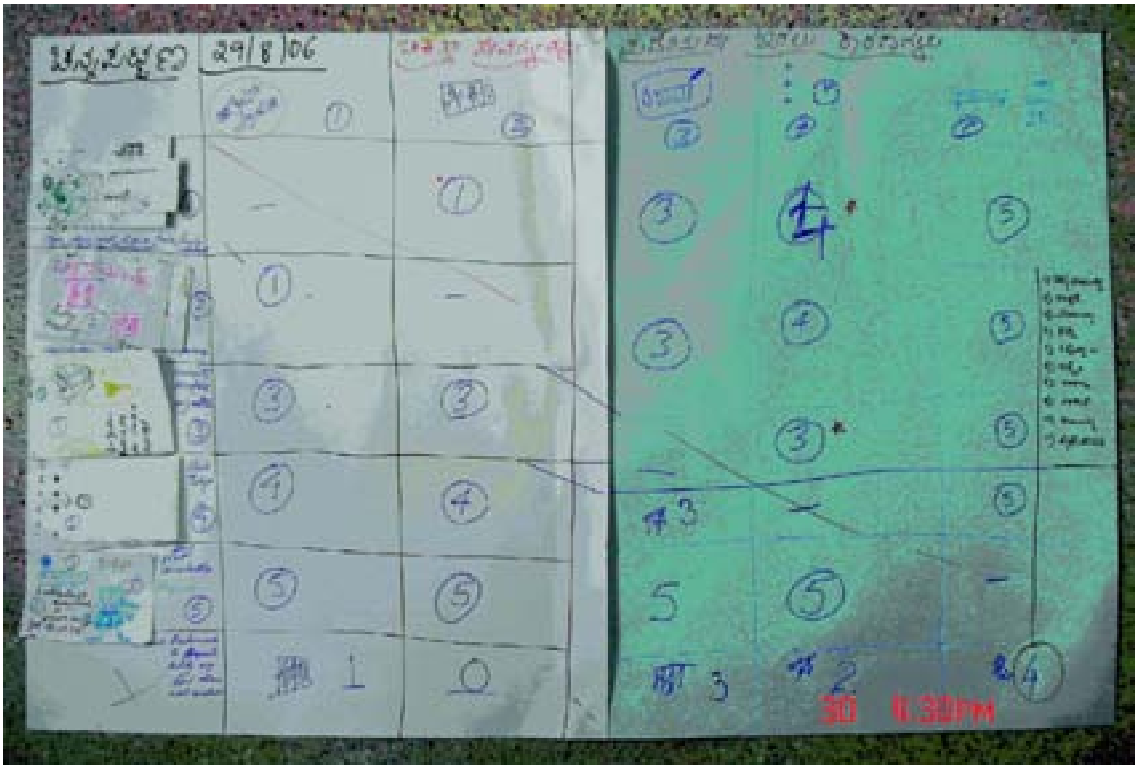

SAMPLE PREFERENCE RANKING

Process 9 CONDOM ACCESSIBILITY AND AVAILABILITY MAPPING

Aim: To map the condom availability points and to understand if they are easily accessible to FSWs.

Description: The participants by using maps identify condom availability points and analyse their accessibility to FSWs.

Materials Required: Maps and pens.

Duration: 120 minutes

Process:

1. Begin by discussing with the participants the importance of condoms to prevent HIV. Also discuss that in condom programming the first priority is to make condoms accessible and available and that this exercise is meant to do so.
2. Ask the participants to draw a map of their town or use an existing map of the town.
3. Ask the participants to mark all the places where FSWs solicit clients. Also ask the participants where the sexual act takes place. Mark all these places on the map using *bindis* of two different colour: one to indicate sites where solicitation takes place and the other to indicate sites where the actual sexual act takes place.
4. Then ask the participants to discuss and understand each site to see when it is active (soliciting and sex work) and at what time of the day. Mark with colour depicting the site as active either only in the day or at night or both the times.
5. Then ask the participants to mark the condom depots in the map symbolically to indicate whether the depots are function during the day or at night or round the clock.
6. Once the map is complete ask the following questions:
   - Are there condoms depots in all the sites where soliciting or sex work takes place? If not, what are the reasons? Do the sites, e.g. home-based sites, which do not have depots, prefer direct distribution?
   - Do all the sites that are active during the day or night or round the clock have condom depots that are open at the same time as the sites are active?
   - Are condom depots accessible to the FSW?
7. Conclude by stating the importance of access to condoms at the right time and place. Draw up a plan to fill the gaps if any.

Example 1.6: Condom Accessibility and Availability Map

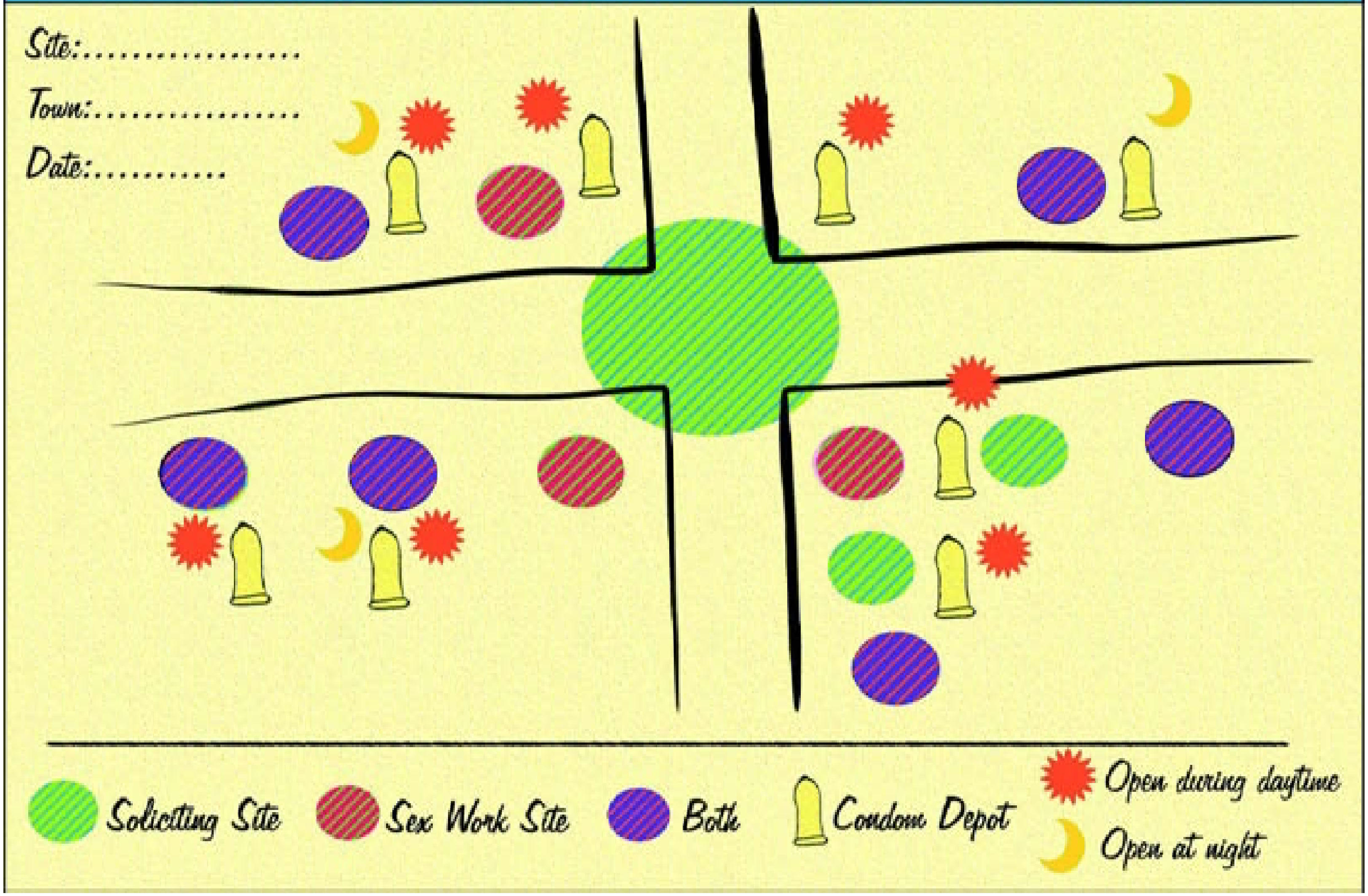

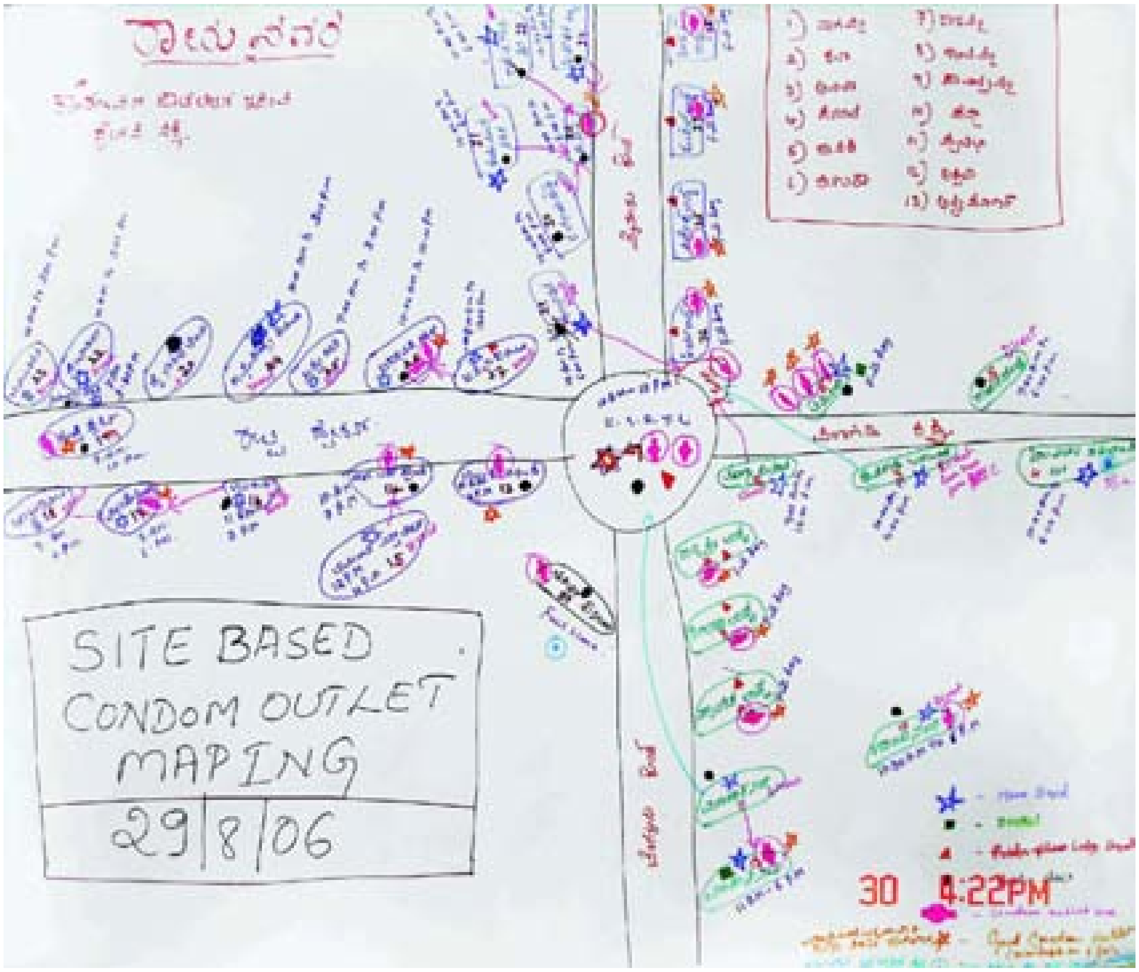

SAMPLE CONDOM ACCESSIBILITY AND AVAILABILITY MAP

Process 10 PEER MAPS

Aim: To understand the nature of outreach done by PEs with the FSWs they work with.

Description: The participants by using maps understand and analyse the outreach with FSWs that they are accountable for.

Materials Required: Charts and pens

Duration: 120 minutes

Process:

1. Ask the peers to map the sites in the town where they work and meet their community members.
2. In these sites ask the PEs to map the FSWs that they are accountable for. Ask them to depict the high-volume, medium-volume and low-volume FSWs in these sites using different colour codes.
3. Now ask the PEs to indicate the number of times each of them met the FSWs they are working with, in the last month.
4. Then ask each of them how many condoms were distributed to each of the FSWs contacted.
5. Also ask each PE to mark the condom outlet boxes in these sites.
6. Now ask each of the PEs to analyse the map by answering the following questions:
   - In the previous month, did the peer meet all FSWs that she is working with? If not, why?
   - Based on the volume of sex work, was there any difference in kind of outreach done by the peer? Did she meet high-volume FSWs more often and the low-volume FSWs less often?
   - Were the condoms distributed based on the volume of sex work? Were enough condoms distributed to cover all the sexual acts of each of the FSWs? Is there a shortfall? How is this shortfall in condom distribution being filled? Is it through the depots? Are the clients bringing condoms?
7. Conclude by saying that it is important to understand the need of each of the FSWs, that a peer is accountable for planning regular contact and condom distribution accordingly. This will ensure that condoms are available with FSWs whenever they are needed and at the same time will avoid dumping of condoms where there is no need.

Note: These maps can be adapted to include other indicators like clinic attendance, access to crisis support, access to entitlements, etc.

Example 1.7: Peer Maps

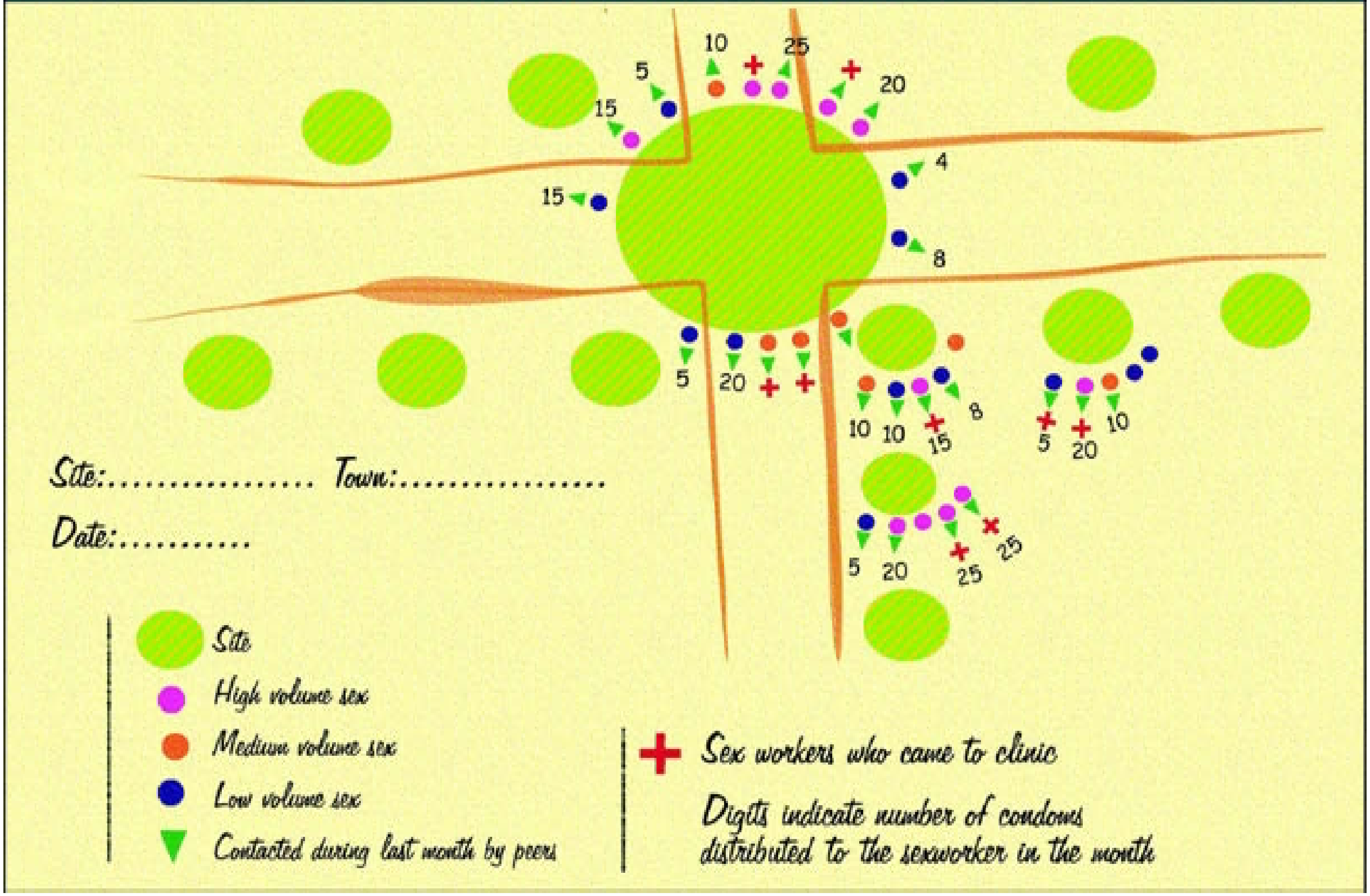

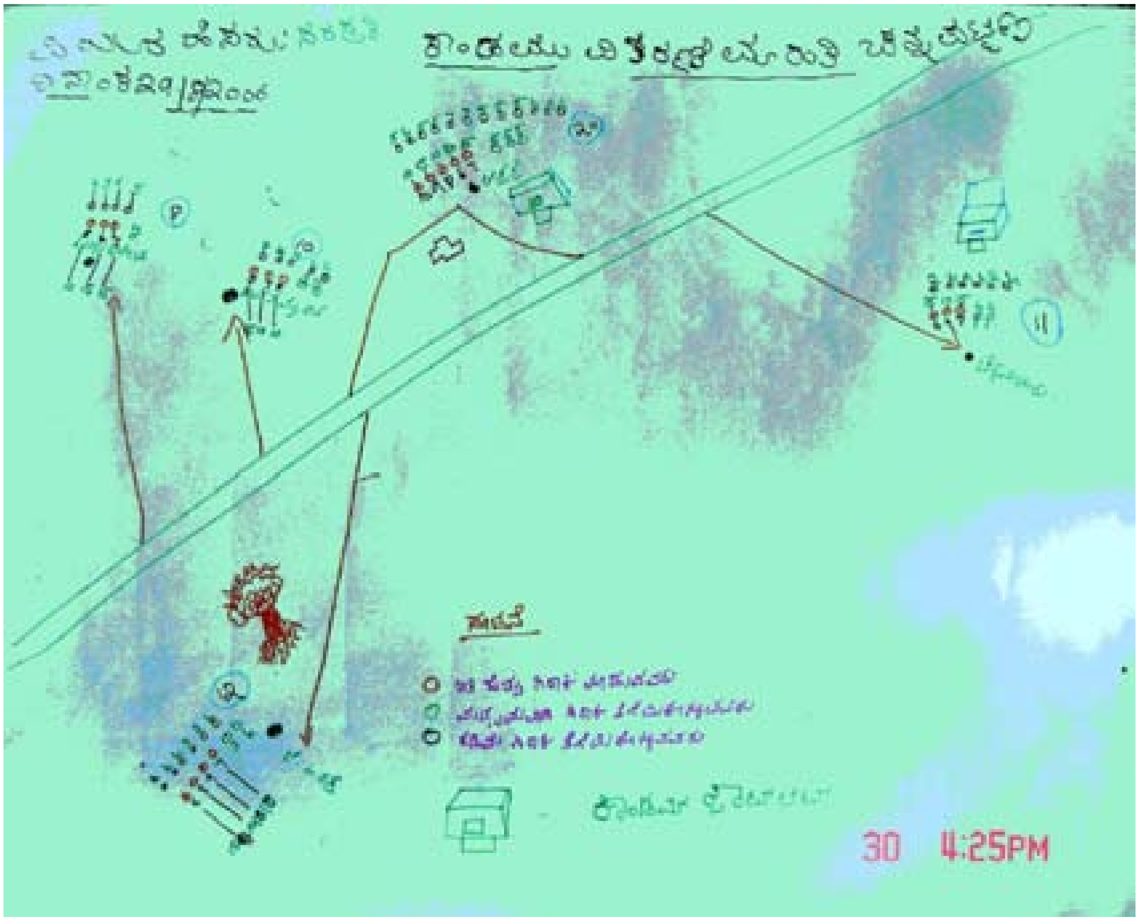

SAMPLE PEER MAP

Process 11 SEX WORK TYPOLOGY-WISE OUTREACH PLANNING

Aim: To understand the link between typology of sex work and outreach

Description: The participants through discussion and analysis of peer outreach understand the link between outreach and typology of sex work.

Materials Required: None

Duration: 120 minutes

Process:

1. Explain to the participants that it is important to recognise and understand the link between outreach, typology of sex work and timing of sex work.
2. Ask the participants to list the FSWs that they are accountable for but have not met in the last two months. This information can be generated from the peer calendars.
3. For each of the FSWs listed above, ask the peers to provide the following information:
   - Place of residence
   - Place of soliciting
   - Place of sex work
   - Ideal timing for outreach (morning, afternoon, evening, night)
4. When the participants complete this information, ask them to identify commonalities in typology and timing of outreach in those mentioned in the list. Bring out the characteristics of these.
5. Then ask the following questions:
   - Is there a link between the number of FSWs who are not contacted and typology of sex work? Which typology of FSW is left out from outreach most often?
   - Is there a link between those who are left out and the timing of outreach? Are FSWs who practise sex work at night or at a specific time of the day left out from outreach?
6. Now ask the participants to develop a strategy for outreach to a typology of FSWs who practice are left out from outreach. Ask the participants to plan how to contact, provide services and give condoms to FSWs who are often or always left out from outreach services.
7. Conclude by asking if there are any questions.

Note: The participants can use pictures.

Example 1.8: STOP

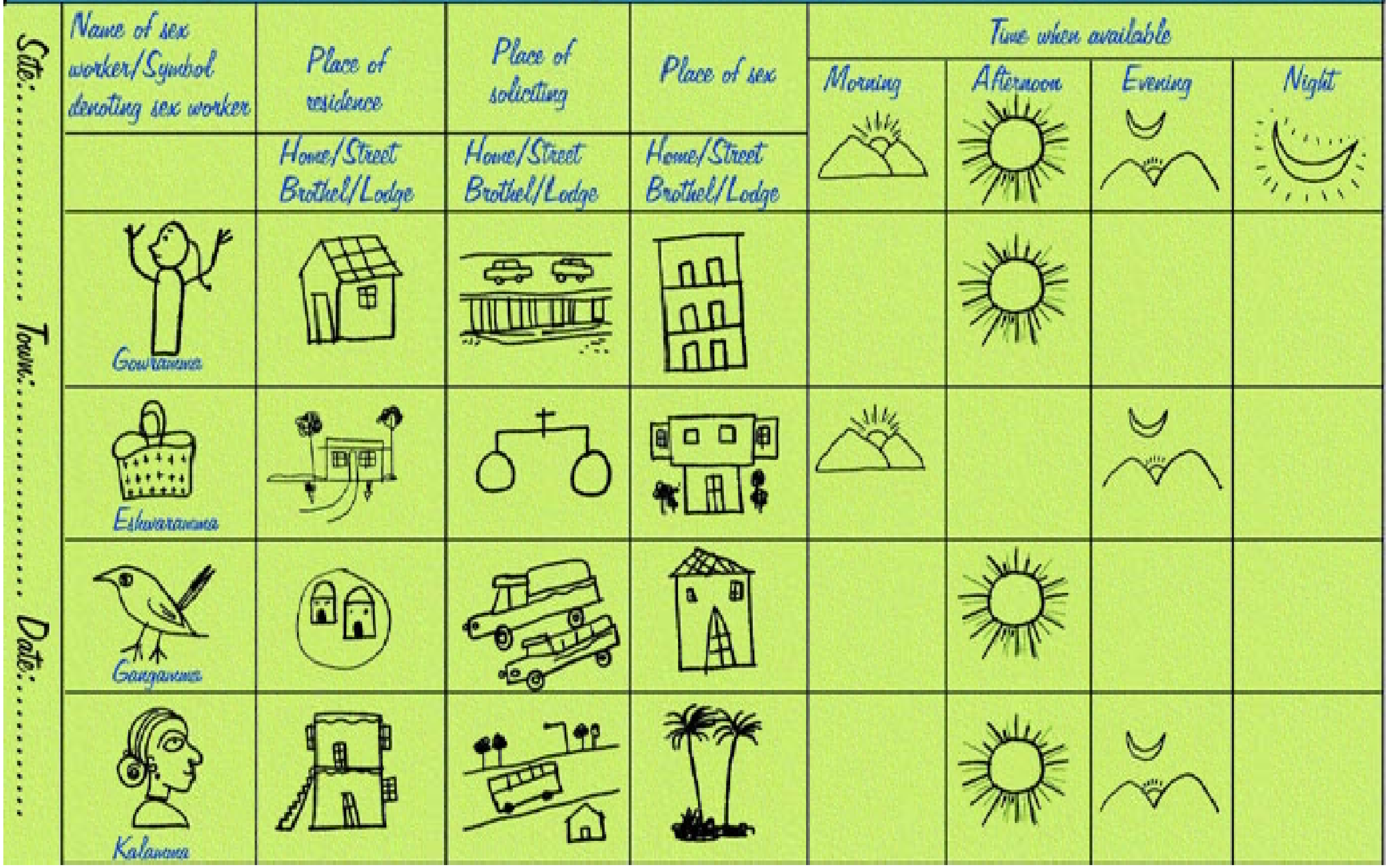

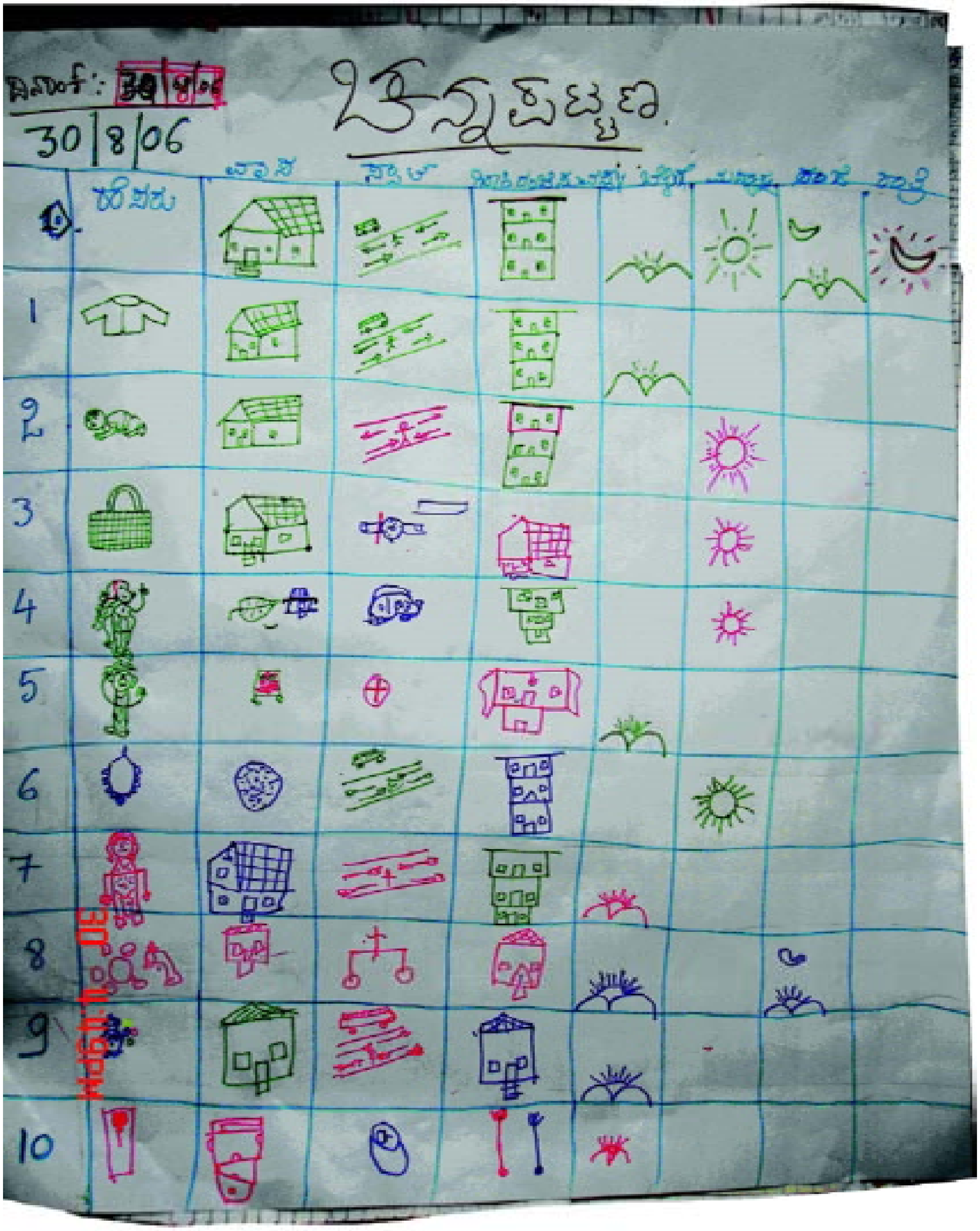

SAMPLE STOP LIST

Section III Tools for Monitoring Project Outreach Planning

The following tools can be used by PEs and ORWs to evaluate progress in outreach and delivery of a minimum package of services to FSWs in their area.

PE Daily Activity Report

A PE records new and repeat contacts, one-to-one sessions, one-to-group sessions, referrals to clinic, condom demonstrations, condom distribution and one-to-ones with a regular partner in the daily activity report. This is pictorial to be user-friendly for low-literate PEs. The data in the daily activity report is used by the ORW to update the individual tracking sheet.

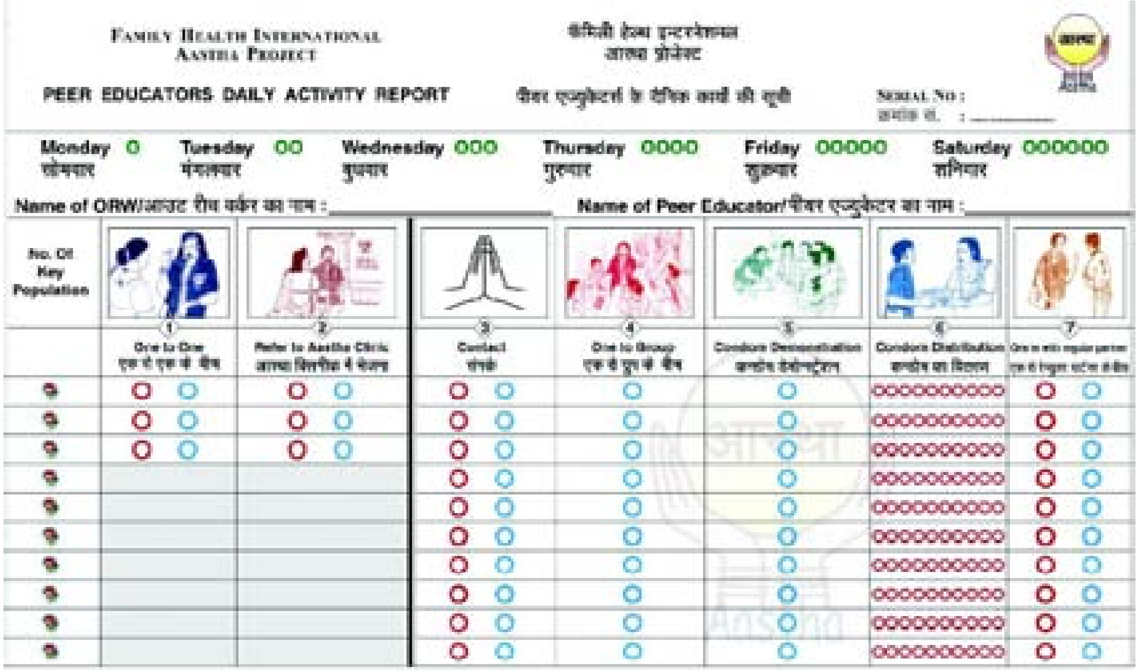

SAMPLE PE DAILY ACTIVITY REPORT

Individual Tracking Sheet

The individual tracking sheet provides the list of all the FSWs in a given site managed by a given PE/ ORW. The services provided to each FSW every week are marked against her name. It helps to monitor the number of FSWs who were provided with the minimum packet of services during the month. Every month the ORW fills up the individual tracking sheet and analyses it along with the PE. The ORW discusses with the PE any difficulties in providing services to the FSWs and makes a plan for the future.

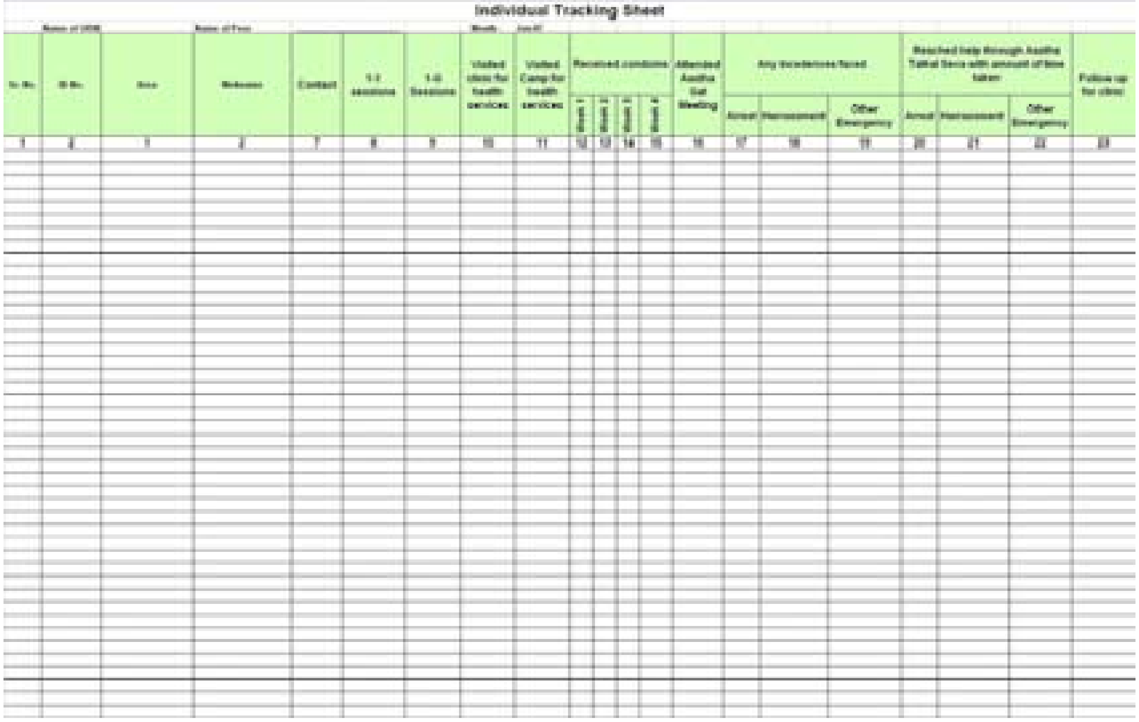

Individual Tracking Sheet

[illegible]	[illegible]	[illegible]	[illegible]	Contact	1-1 sessions	1-G Sessions	Visited clinic for health services	Visited Camp for health services	Received condoms				[illegible]	Any incidences faced			Reached help through [illegible] with amount of time taken			Follow up for clinic
									[illegible]	[illegible]	[illegible]	[illegible]		Arrest	Harassment	Other Emergency	Arrest	Harassment	Other Emergency	
1	2	[illegible]	[illegible]	7	8	9	10	11	12	13	14	15	16	17	18	19	20	21	22	23

SAMPLE INDIVIDUAL TRACKING SHEET

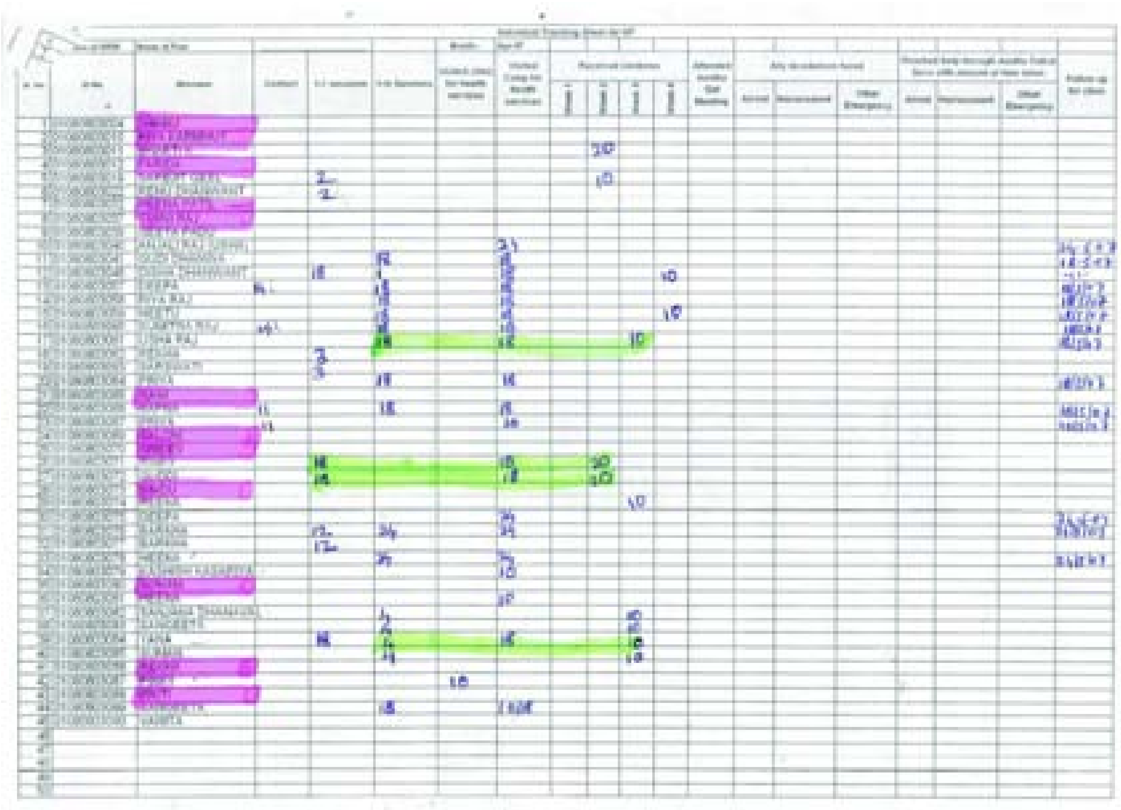

SAMPLE INDIVIDUAL TRACKING SHEET, COMPLETED

Outcomes of Outreach to FSWs: The "Minimum Package"

Each FSW covered by a TI is entitled to a "Minimum Package". An effective outreach strategy should ensure that she gets them. The package includes the following services:

- One quality IPC session provided
- Clinical services offered
- Membership in NGO/CBO
- Quality condoms provided every week

At least one project-related service (clinic, counselling, IPC session, condoms, regular meeting, etc.)

Delivery of Minimum Packages can be summarized using information from the PE Daily Activity Report and the Individual Tracking Sheet.

Peer Educator Zone wise performance of Aastha Minimum Package (AMP) Score

Location of Intervention	ORW Responsible	Peer Educators Responsible	Place of Intervention	No. of Key Population handled	Services: Provided One-One Session	Services: Provided Clinic Services	Services: Attended Aastha Cell Meeting	Provided Condoms: Once in a Month	Provided Condoms: Twice in a Month	Provided Condoms: Thrice in a Month	Provided Condoms: Four Times in a Month	AMP Score	Maximum AMP Score	[illegible]	[illegible]	[illegible]
P M G P Colony	Poonam [illegible]	Lata	P.M.G.P. Colony	50	34	19	19	15	10	5	3	88	200	44%	39%	66%
		Naseem	P.M.G.P. Colony	50	38	21	12	12	6	2	2	81	200	40%	42%	44%
		Laxmi	Shatabdi Nagar	85	29	42	11	20	14	8	4	104	340	[illegible]	49%	54%
P M G P Colony	[illegible]	Lata	[illegible] Nagar	50	30	30	7	27	13	6	3	81	200	40%	60%	100%
		Shamshad	Prem Nagar	47	30	25	7	32	6	4	1	77	188	41%	53%	91%
		[illegible]	Shatabdi Nagar	50	28	18	8	22	16	8	4	77	200	[illegible]	36%	100%
		Ameena	Discovery Bar	30	18	14	8	8	4	2	1	39	120	[illegible]	47%	50%
		[illegible]	Raj palace	13	8	7	0	4	2	0	3	22	52	43%	54%	65%
		[illegible]	[illegible] Nagar	37	30	12	8	7	18	7	5	68	148	46%	32%	100%
		[illegible]	Prem Nagar	50	42	18	8	36	8	4	1	87	200	44%	36%	98%
		[illegible] Shaikh	Sai baba Nagar	75	28	30	5	42	14	8	6	96	300	[illegible]	40%	88%
Transit Camp	Jyoti	Jaitun	Kumbharwada	51	30	32	8	28	8	2	2	86	204	42%	63%	78%
		[illegible]	Kumbharwada	48	40	16	0	27	7	3	3	71	192	[illegible]	33%	81%
		Laxmi [illegible]	[illegible] Nagar	75	33	25	10	31	8	2	0	78	300	[illegible]	33%	52%
		Vanita [illegible]	[illegible] Nagar	50	21	26	10	30	7	5	2	70	200	[illegible]	50%	68%
M L Camp	[illegible]	Laxmi [illegible]	Sanjay Gandhi Nagar	50	36	20	8	28	7	5	3	80	200	40%	40%	70%
		Sharda	Sanjay Gandhi Nagar	50	38	20	10	32	6	3	0	81	200	41%	40%	82%
		Naseem	Sanjay Gandhi Nagar	50	18	18	4	22	5	3	1	49	200	[illegible]	36%	62%
M L Camp	[illegible]		[illegible]	84	60	40	6	38	10	12	3	131	336	[illegible]	48%	65%
		Tarannum	[illegible] Nagar	90	41	37	0	38	15	8	4	105	360	[illegible]	41%	72%
		Shehnaz Khan	Ambedkar Nagar	70	37	34	0	38	16	8	3	98	280	[illegible]	40%	93%
			Total SHED	1,156	670	499	142	492	198	107	52	1,666	4,620	36%	43%	74%

Need Attention	11	6
Moderate but Need attention	10	8
Doing well - Encourage	0	11

SAMPLE SITE-WISE TRACKING SHEET FOR DELIVERY OF MINIMUM PACKAGE, COMPLETED (WITH USE OF COLOUR-CODING TO FLAG AREAS FOR ATTENTION)

ANNEXURE 6a

Dialogue-Based Interpersonal Communication By and With HRGs

Section I The Dialogue-Based IPC Framework

EYE to EYE

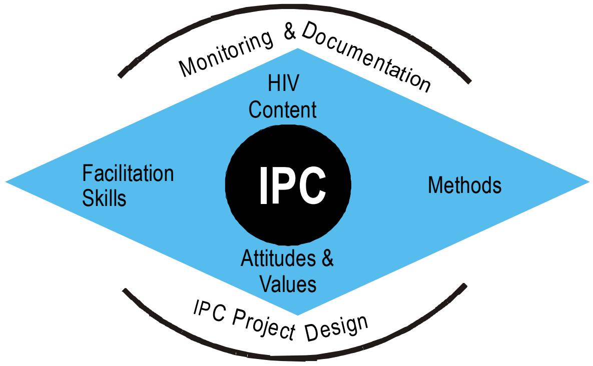

IPC framework for HIV/STI risk reduction with core HRGs

> IPC moves beyond messages and, through face to face interaction, dialogue and critical reflection, helps HRGs identify barriers to STI/HIV risk reduction, analyse these barriers and plan ways to address them.

As represented in the figure above, the IPC framework includes the four cornerstones of IPC – HIV content, methods, facilitation skills, and values and attitudes – as well as the two essential aspects of creating successful IPC programs – IPC project design and ongoing monitoring and documentation.

- **HIV/STI content** covers the barriers to risk reduction for HRGs including social and environmental factors, as well as epidemiological issues.
- **Methods** are processes used to stimulate IPC and are selected to make the best possible use of each IPC opportunity.
- **Facilitation skills** focus on ways to promote real dialogue, discussion and debate rather than merely giving messages.
- **Attitudes and values** deal with the appropriate attitudes and values for working with HRGs and underlie all capacity areas essential to an organisation implementing IPC projects.
- **Project design** looks at how the project is organised to be both efficient and effective.
- **Monitoring and documentation** are used to improve project processes and to share learning within and beyond the IPC project.

These components of the IPC framework complement and reinforce each other, and together enhance the sustainability, quality, integrity and impact of interventions.

Section II Methods for IPC with HRGs for Reducing STI/HIV Risk

Method Typology

All IPC methods are based on participatory learning and action (PLA) approaches, and there are different types:

Type	IPC Methods
Simulation	1. Play Safe<br>2. HRG Advisors<br>3. Statues<br>4. Margolis Wheel
Visual Representation	5. HRG Drawings<br>6. Graffiti<br>7. Body Mapping<br>8. Lovers
Diagramming	9. Why Is It So?<br>10. Chakra Wheel
Mapping & Ranking	11. HIV Services Map<br>12. How Hot Is the Spot?
Stories	13. Story With a Gap<br>14. Storytelling

IPC Capacity Standards for Method Selection and Use

1. IPC tools are field tested with the HRGs before use to determine their suitability, acceptability and effectiveness in different situations (drop-in centre, outreach, clinic etc.).
2. All IPC tools used have moved beyond message delivery to dialogue-based methods that promote critical reflection and enquiry ("from seeing and reading to listening, thinking, asking and talking").
3. All IPC tools used are designed to help HRGs identify and analyse barriers to risk reduction, find acceptable and realistic solutions and plan how the solution will be adopted.
4. IPC tools are selected to maximise the quality of the IPC opportunity (e.g. they are appropriate for the type of HRG, for where IPC is taking place, the time available, the level of facilitator's skill, the HIV/STI risk reduction priorities of the HRGs, number of participants, degree of privacy, whether the encounter is a one-off or a repeat, literacy skills of participants, level of engagement of HRGs).
5. IPC tools help to strengthen the motivation, knowledge and skills for HIV/STI prevention among HRGs. They also help HRGs to access HIV/STI related services and resources in the community and to access peer/social support for HIV/STI prevention (i.e. they help HRGs to strengthen knowledge, resource, positional and personal power).
6. During IPC, HRGs are always encouraged to share their own means of HIV/STI prevention.

Method 1. Play Safe	
Purpose of the method	To help participants explore different safe sex techniques.
Requirements for facilitation	Good knowledge of safe sex and safe injecting and sharing strategies and techniques, comfort with talking about using drugs, sex and with explicit demonstrations of safe sex.
Degree of privacy	High
Material required	Enough space for participants to act out different situations.
Method	1. Divide participants into two teams.<br>2. Ask each team to prepare and simulate a situation where they demonstrate sex acts that are safe.<br>3. One group demonstrates.<br>4. After the demonstration, the other group analyses the simulation to check:<br>■ Whether the acts demonstrated are really safe<br>■ Whether they are realistic and practicable<br>■ Whether they can be practised in any situation or would require special circumstances<br>■ Whether anything can be done to make the act even safer<br>■ The second group demonstrate and the other group analyses using the same set of criteria.<br>6. Based on the assessments the groups are awarded points.<br>7. Finish the session by asking the group to reflect on what they have shared and learned during the session that would be useful for them.
Adaptation for repeat use	For repeat use, the groups can be asked to demonstrate:<br>■ Sex acts of particular kinds, such as vaginal, anal, oral, physical but non-penetrative, non-contact.

Method 2. HRG Advisors	
Purpose of the method	To enable HRGs to discuss who they can go to for advice and to build skills in assessing advice given on HIV/STI risk reduction.
Requirements for facilitation	Knowledge of HRG context and social networks, also of what the group needs to gain in terms of knowledge and skills for HIV/STI risk reduction.
Degree of privacy	Medium, can be done in public depending on the nature of the dilemma being discussed.
Material required	Props or labels to remind people who the advisors are
Method	1. Settle the group with an icebreaker.<br>2. Split the participants into small groups and ask them to come up with a barrier to HIV/STI risk reduction that is a problem for their HRG group/subgroup (e.g. police harassment, fear of HIV testing). Share the problems from each group and decide together which one is a priority to analyse.<br>3. Ask the participants to list "people their community group/subgroup go to for advice, people whose advice is trusted and respected". Choose 5 or 6 of these HRG advisors and ask for volunteers to role play the advisors.<br>4. Ask the group who came up with the problem chosen for analysis to quickly present the dilemma to the advisors. They should do this by telling a short story about a fictional character who has the problem, giving the character a name and presenting some imaginary background information.<br>5. Ask each advisor in turn to give solutions to the problem presented. Ask the participants to say which advisor has given the best solution. Briefly ask volunteers to act out this solution and discuss how/if it worked and if not, why not.<br>6. Now ask the participants if they know of anyone in real life who has faced this problem. What happened?<br>7. Finish the session by asking the group to reflect on what they have shared and learned during the session that would be useful for them.
Adaptation for repeat	This activity can be repeated using different dilemmas and different advisors.

Method 3. Statues	
Purpose of the method	To help HRGs identify and plan ways to address barriers to HIV/STI risk reduction.
Requirements for facilitation	Good knowledge of HIV/STI risk reduction strategies which HRGs can realistically use within their own context.
Degree of privacy	Quite high: this can attract onlookers which can inhibit participants.
Material required	Polaroid cameras can be used to snap the tableaux. The pictures are for the HRGs to take away with them.
Method	1. Settle the group in with an icebreaker.<br>2. Ask the group to brainstorm ways in which you can get HIV. Correct any misconceptions and challenge any prejudices.<br>3. Now split the group into sub-groups of 3 or 4 people. Ask each group to decide on a "freeze frame" or "tableau" (arranging themselves in a particular way then standing as still as statues, not saying anything) showing one way to reduce the risk of HIV.<br>4. Go round the groups if necessary to clarify what you want them to do.<br>5. Now ask each group in turn to show their tableau. Facilitate a discussion amongst the remaining participants about each tableau.<br>▪ What does the tableau show?<br>▪ Will this reduce the risk of HIV?<br>▪ If so, how easy would their suggestion be to put into practice in real life?<br>▪ Are there any changes that could be made to the tableau to make their risk reduction suggestion more effective?<br>6. If there are suggestions for change, if everyone agrees let the group amend their tableau arrangement accordingly.<br>7. Finish the session by asking the group to reflect on what they have shared and learned during the session that would be useful for them.
Adaptation for repeat	Alternatively, ask for tableaux that depict risk behaviours and then ask the participants to rearrange each tableau so that the risk of HIV/STI infection is reduced.

Method 4. Margolis Wheel	
Purpose of the method	To help HRGs identify and plan ways to address barriers to HIV/STI risk reduction.
Requirements for facilitation	Good knowledge of what makes HRGs vulnerable to HIV in the particular context. Knowledge of strategies to reduce risk of HIV.
Degree of privacy	Low
Material required	None
Method	1. Settle the group with an icebreaker.<br>2. Put the group into pairs. Ask each pair to brainstorm situations that might make people vulnerable to HIV/STI infection. Give an example relevant to the HRG group. Go round the pairs, correct misconceptions, challenge prejudices and make sure that each pair has a different situation.<br>3. Arrange the group so that there is an inner and outer circle with pairs facing each other. Explain that the inner group are "consultants" and the outer group have come to get their advice. The outer group have 2 minutes with each consultant to explain the situation that makes people vulnerable to HIV/STI infection and ask them for advice on how to change the situation to reduce the risk.<br>4. Start the clock. After 2 minutes ask all those in the outer circle to move round to the next consultant and ask for advice. Repeat this until those in the outer circle are in their original places. Now ask the pairs to swap round so that those in the outer circle now become the consultant. Repeat the activity.<br>5. Finish the session by asking people to share the best advice they got for their particular situation. Ask if anyone did not get satisfactory advice and ask the group to comment.<br>6. Finish the session by asking the group to reflect on what they have shared and learned during the session that would be useful for them.
Adaptation for repeat use	Ask the group to brainstorm situations in their personal life or work life. Ask the advisors to give solutions that can only be done by an individual or by a group of peers.

Method 5. HRG Drawings	
Purpose of the method	To enable HRGs to discuss how HIV/STI risk can be reduced in the context of their everyday lives.
Requirements for facilitation	Good knowledge of HIV/STI risk behaviours and risk reduction techniques and strategies for HRGs.
Degree of privacy	Low
Material required	Chart paper, markers
Method	1. Settle participants with an icebreaker.<br>2. Give each participant chart paper and markers. If there are many participants, split them into groups and give each group paper and markers.<br>3. Ask each group to draw a scene from the lives of their HRG group/ subgroup. It can be anything they want to portray from the time of waking up to going to bed. It can be part of work or personal lives.<br>4. Ask each group to present their drawing to the rest.<br>5. Ask all the participants to look at the drawings and to pick out aspects of HRG lives that might make them vulnerable to HIV. Correct any misconceptions, challenge any prejudices.<br>6. Now ask the groups to take back their drawings and to make one change to their drawing that would lessen the risk of HIV/STI.<br>7. Discuss the changes to assess them for how realistic and acceptable they are to the HRG group/subgroup.<br>8. Finish the session by asking the group to reflect on what they have shared and learned during the session that would be useful for them. Let the HRGs keep their drawings.
Adaptation for repeat use	This activity can be repeated by specifying the type of scene to be drawn – with relatives/family, with close friends, with the authorities, etc.

Method 6. Graffiti	
Purpose of the method	To help participants explore different kinds of sex acts that the HRGs usually engage in with their sexual partners (whether intimate partners or paying ones) and the HIV/STI risks associated with them, so that they can work out ways of making sex safer.
Requirements for facilitation	Good knowledge of safe sex strategies and techniques, comfort with talking about sex in some detail.
Degree of privacy	High
Material required	Chart paper and coloured markers
Method	1. Ask participants to draw the different sex acts they usually engage with their sexual partners on chart papers. Once the drawings are done discuss with the participants the degree of risk of HIV/STI transmission that each sex act entails. Ask them to put symbols (ticks, numbers or any other) against drawings of each sexual act to denote the degree of risk (High, Low or No risk).<br>2. Discuss with participants if they can suggest any other way of having sex which is safer. Give examples of safe sex practices that are not mentioned by them.<br>3. Through all the steps ensure that the participants are not feeling inhibited or uncomfortable.<br>4. Finish the session by asking the group to reflect on what they have shared and learned during the session that would be useful for them.
Adaptation for repeat use	On different occasions ask participants to draw either intimate sexual partners, or paying partners.

Method 7. Body Mapping	
Purpose of the method	To enable HRGs to explore HIV/STI vulnerability factors relating to the body and to discuss non-penetrative sex techniques .
Requirements for facilitation	Good knowledge of HIV/STI vulnerability factors relating to the body.
Degree of privacy	Medium to high
Material required	Chart paper and markers, or chalk for drawing on concrete
Method	1. Settle the participants in with an icebreaker.<br>2. Ask for a volunteer in each group to lie on the ground and have someone trace the outline of his/her body on the ground or on the chart paper.<br>3. Ask participants to treat the outline as a naked body and to draw in the details.<br>4. Now ask participants to discuss the following questions:<br>▪ Where are the places on the body that feel good when touched?<br>▪ Which parts of the body are vulnerable to HIV? How can the virus enter the body? What makes it easier for the virus to enter the body? Correct any misconceptions.<br>▪ What options are there for safer sex, particularly non-penetrative sex?<br>5. Finish the session by asking the group to reflect on what they had shared and learned during the session that would be useful for them. Let the HRGs keep their drawings.
Adaptation for repeat use	Body mapping can be repeated to look at the symptoms of different STIs or to focus on what gives pleasure in sex.

Method 8. Lovers	
Purpose of the method	To enable HRGs to explore HIV/STI vulnerability factors relating to sexual partners and to discuss risky and less risky sexual behaviours .
Requirements for facilitation	Good knowledge of HIV/STI risk factors.
Degree of privacy	Medium
Material required	Chart paper and markers
Method	1. Settle the participants with an icebreaker.<br>2. Ask participants to draw a picture of a HRG member from their own category at the centre of the chart.<br>3. Ask them to draw pictures of her sexual partners all around the HRG's picture and describe the partners (without naming them) – who are they, what do they do, how old are they, how are they related to the HRG, how did they meet, etc.?<br>4. Ask participants to indicate against each partner's picture or symbol what kind of sex (penetrative or non-penetrative) the HRG member in question did with the partner in the last one week, and how many times.<br>5. Ask participants to deliberate on:<br>▪ How safe each act was<br>▪ What would the HRG have to do to make the unsafe sex acts safer?<br>▪ To act on similar solutions, what practical steps would the participants have to take?<br>6. Finish the session by asking the group to reflect on what they have shared and learned during the session that would be useful for them. Let the HRGs keep their drawings.
Adaptation for repeat use	Lovers can be repeated to look at different categories of HRGs and different behaviours.

Method 9. Why Is It So?	
Purpose of the method	To help HRGs analyse why risk behaviour occurs and what can be done to reduce them.
Requirements for facilitation	Knowledge of risk behaviours and the difference between risk behaviours and vulnerability factors, knowledge of HRG context.
Degree of privacy	Low
Material required	Chart paper and coloured markers
Method	1. Ask participants to name the different kinds of behaviours that put people at risk of HIV/STI infection. Correct any misconceptions.<br>2. Pick one of the risk behaviours.<br>3. Ask them to draw a symbol of this risk behaviour in the centre of the flipchart inside a circle.<br>4. Ask "Why is it so?" and ask them to draw and or write the reasons for the risk behaviour in balloons.<br>5. Keep asking "Why is it so?", adding further reasons in connecting balloons until they can think of no more.<br>6. Ask the participants what the diagram says about:<br>▪ What are the most important reasons (vulnerability factors) for risk behaviour?<br>▪ What are the ways that the HRG group already try and reduce risk behaviour?<br>▪ What would further help the HRG group avoid the risk behaviour in the diagram?<br>7. Finish the session by asking the group to reflect on what they have shared and learned during the session that would be useful for them.
Adaptation for repeat use	Pick different risk behaviours (e.g. unprotected anal sex, unprotected vaginal sex).

Method 10. Chakra Wheel	
Purpose of the method	To help HRGs identify and plan ways to address barriers to HIV/STI risk reduction.
Requirements for facilitation	Knowledge of HIV/STI prevention methods and of HRG context in relation to HIV/STI risk reduction.
Degree of privacy	Can be done outdoors in impromptu locations if the location is not right within the public domain.
Material required	The wheel can be drawn in the dust with a stick or with chalk on concrete, alternatively use markers and flipchart.
Method	1. Settle the group with an icebreaker.<br>2. Ask the group to brainstorm ways in which their HRG group or sub-group can reduce the risk of HIV/STIs. Correct any misconceptions, challenge any prejudices. Get the group to settle on 8 important risk reduction methods or strategies.<br>3. Ask the group to draw a circle and divide it into 8. Assign one risk reduction method or strategy to each segment of the wheel using a symbol or object agreed by the group. Now ask the group to discuss how easy it is for their HRG group or sub-group to use these methods or strategies and shade in the segment accordingly. If it is very difficult for the HRG group to use the method or strategy then only a small part of the segment is shaded in.<br>4. When the wheel is complete, ask the group to reflect on the segments that have least shading. What action would need to happen to make it easier for the HRG group to use that risk reduction method or strategy? Who would need to be involved in that action? What first steps could be taken immediately and by whom?<br>5. Finish the session by asking the group to reflect on what they have shared and learned during the session that would be useful for them. If necessary, offer one-to-one work with people who may have specific and personal concerns.
Adaptation for repeat use	Keep the original charts or ask the HRGs to keep them and work on a different risk reduction method at each meeting.

Method 11. HIV/STI Services Map	
Purpose of the method	To help participants map, assess and learn how to access formal and informal HIV/STI services available to HRGs in the project site.
Requirements for facilitation	Knowledge of types of formal and informal services important for HRG use in HIV/STI prevention.
Degree of privacy	Low
Material required	Chart paper and coloured markers
Method	1. Ask the participants to draw a map of the site including a few main landmarks.<br>2. Ask the participants to include in the map any places or people that their HRG group could go to get support for HIV/STI prevention and treatment.<br>3. Ask the participants to put against each intervention:<br>■ What each service provides<br>■ How each service helps reduce risk of HIV/STI infection<br>■ A symbol if the service is very important in HIV/STI prevention<br>4. Ask them to identify factors that make a particular service attractive to them (such as, distance, cost, behaviour of service providers, confidentiality, effectiveness of services provided, availability and timing, etc.<br>5. Now ask the participants to rank the services marked as important in terms of how accessible they are to HRGs like themselves (high, medium, low).<br>6. Now ask them to discuss the services ranked with low accessibility. What could be done to make these important services more accessible to HRGs like themselves?<br>7. Finish the session by asking the group to reflect on what they have shared and learned during the session that would be useful for them.
Adaptation for repeat use	This activity is long and can be broken up with repeat use. Keep the chart papers to continue the discussion in the next session.<br>Use original papers after some time and ask participants how site has changed.

Method 12. How Hot is the Spot?	
Purpose of the method	To help HRGs identify and plan ways to address barriers to HIV/STI risk reduction.
Requirements for facilitation	Knowledge of HRG context and of relative risk of particular behaviours.
Degree of privacy	This activity generates information that may cause sensitivities between HRGs and the authorities, so some privacy is required.
Material required	Marker pens and chart paper
Method	1. Settle the group with an icebreaker.<br>2. Ask the group to draw a map of the local area, including any local landmarks to orient the map. Now ask them to use a symbol to indicate on the map the locations where behaviour occurs that puts their HRG group at risk of HIV/STI infection.<br>3. Now ask the group to rank the locations using symbols for "high", "medium" or "low" according to the level of risk behaviour in each location (in terms of numbers of people or frequency of risk behaviour occurring).<br>4. Ask the group to look at the locations ranked as high. Ask them to discuss what change needs to happen generally to make the location into a medium or low rank. Then ask what individual HRGs or small peer groups could do to reduce risk behaviour in these locations.<br>5. Finish the session by asking the group to reflect on what they have shared and learned during the session that would be useful for them.
Adaptation for repeat use	This activity is long and can be broken up with repeat use. Keep the chart papers to continue the discussion in the next session. Use original papers after some time and ask participants what may have changed in the site.

Method 13. Story With a Gap	
Purpose of the method	To help HRGs plan ways to address barriers to HIV/STI risk reduction.
Requirements for facilitation	Knowledge of HRG HIV/STI vulnerability factors and risk reduction strategies, ability to facilitate planning.
Degree of privacy	Low
Material required	Markers and chart paper when using the variation with drawing
Method	1. Ask the group to quickly draw two different pictures of "someone like themselves". After they have finished these drawings, tell the group that one drawing represents someone who has risk behaviours and is vulnerable to HIV. If necessary explain what is meant by risk behaviour. Ask them to choose which drawing this will be.<br>2. Now ask them details about the imaginary person in the drawing. Help them to build up a story around the drawing:<br>■ What is the name of the imaginary person?<br>■ Where do they live?<br>■ What is their life like?<br>■ Why are they vulnerable to HIV?<br>3. Tell them the other drawing is of someone who does not have any risk behaviour and who is not very vulnerable to HIV. Ask them similar questions and help them to build a separate story around the imaginary person in the second drawing. This time ask them why the person is not very vulnerable to HIV.<br>4. When the two stories are complete, ask the group to think of things that would help the person in the first drawing become more like the person in the second drawing. After some discussion, ask them to settle on one change (or more than one, depending on the time available) that would really help the person to reduce their HIV risk. It does not necessarily have to be a change that the person in the drawing would make themself; it might be change that other people have to make.<br>5. Now ask the group to make a series of brief drawings outlining the steps necessary for the change to happen.<br>6. Finish the session by asking the group to reflect on what they have shared and learned during the session that would be useful for them.
Adaptation for repeat use	A good story can be revisited several times to plan how change can happen. Alternatively, new stories can be generated with the same group.

Method 14. HRG Storytelling	
Purpose of the method	To enable HRGs to reflect on options and choices in particular situations that may reduce the risk of HIV/STI infection.
Requirements for facilitation	Good knowledge of situations which may lead to HRGs having HIV/STI risk behaviours, and knowledge of realistic strategies to reduce this risk for HRGs.
Degree of privacy	Medium, can be done in public depending on the nature of the situation chosen by participants for the story.
Material required	Any prop that can act as a "trigger" for the story
Method	1. Settle the participants with an energizer.<br>2. Ask participants to list situations in their daily lives that might lead to behaviour that puts them at risk of HIV/STI infection. Discuss priorities and settle on a particular situation for the story.<br>3. Use the trigger, saying that the *object* (book, watch, shoe, etc.) belonged to someone who was in the *potentially risky situation* chosen (e.g. having group sex, etc.)<br>4. Ask the participants to come up with a short story (in groups or in plenary) about a person in the potentially risky situation they have chosen. They should give the story characters names, describe them, and explain events leading up to the potentially risky situation.<br>5. Now ask participants to volunteer to role play the story. Just before the risk behaviour occurs, ask them to "freeze" the action. Make sure that the volunteers are frozen in a comfortable position. Ask the participants if they know of anyone who has been in a similar situation and ask them to describe what happened next.<br>6. Now ask: What options did the people in the story have at the freeze point to avert or reduce the risk of HIV/STI infection? Once the group has agreed on some realistic options, ask different volunteers to role play them to see how they might work in practice. Discuss the outcomes: did the options help to reduce risk of HIV/STI? If not, why not?<br>7. Now ask: Is there anything different people could have done to avoid the potentially risky situation altogether? Discuss these options for acceptability and for how realistic they might be for HRGs to put into practice.<br>8. Finish the session by asking the group to reflect on what they have shared and learned during the session that would be useful for them.
Adaptation for repeat use	This activity can be interrupted and continued when the group next meet, starting with participants being asked to summarise the story and discussion from the previous meeting.

Section III Self-Analysis Process

Self-analysis of IPC capacity standards allows those directly involved in IPC outreach to HRGs to assess strengths and weaknesses of their work in the context of the NGO/CBO. The analysis has five steps:

Step 1: Building the right environment for discussion.
Step 2: Facilitating a discussion of the IPC capacity standards.
Step 3: Facilitating the group to determine scores for the basic IPC capacity standards.
Step 4: Facilitating the group to analyse capacity needs and identify priorities to be addressed.
Step 5: Preparing a report on the outcomes of the process.

Prior to the first step, the following questions should be considered:

- Who will manage and facilitate the process?
- Will external facilitators be used? If yes, how will they be identified?
- Who will participate in the capacity standards analysis?
- What logistical considerations need to be addressed (scheduling, costs, venue)?
- How will the process be documented? And how will the documentation be used?

Materials needed for the process: A4 paper, marker pens, writing pens, flip charts, photocopies of the basic IPC capacity standards and a copy of the reports from any previous analyses of IPC capacity.

Step 1 Building the right environment for discussion

To get the most out of the self-analysis process outlined in this guide, users must be committed to honest and critical reflection. To foster this, a safe environment for discussion needs to be created. This includes the following:

- The analysis should take place at a time convenient to all participants.
- An environment must be created where participants feel they can be openly critical without fear of negative consequences.
- The facilitator should seek to generate a range of opinions; no one person should dominate the group.
- Participants should be encouraged to give high and low scores when it is warranted rather than just rating everything as average.
- The venue should be quiet and private. Participants should not be allowed to wander in and out to answer phone calls or to leave and re-join sessions.
- If it is not possible to have all the staff involved in the analysis, the group should at least reflect a range of views, experiences and roles within the organisation. It should involve senior staff, field co-ordinators, outreach workers, IPC facilitators and peer educators. Participants should be familiar enough with the capacity area to contribute to the discussion in an informed way.
- Representatives from SACS, PSU and capacity building partner could be invited as observers, but this is not mandatory and may constrain the group.

- An objective facilitator with a strong knowledge of the IPC process should be appointed either from within the organisation or externally. The facilitator is central to the success of the session. They *should not take part* in the discussion, but rather guide it.
- Someone should also be appointed to record the key points of the discussion. This can be used for the final report.

Step 2 Facilitating the discussion

The responsibilities of the facilitator include:

- Introducing the concept of self-analysis and explaining the process that will be followed.
- Ensuring that each of the basic IPC capacity standards is discussed, deliberated and challenged within the group.
- Ensuring that all views are heard and respected. This includes being sensitive to existing hierarchies and ensuring that some members do not intimidate others.
- Ensuring that all questions get appropriate attention and not letting participants become embroiled in a side issue or ongoing disagreement.
- Generating positive and productive group interaction. This includes probing for further information and asking the group to respond to statements by an individual (using questions such as "What do the others feel about that?").
- Encouraging critical reflection and guarding against the group tendency to provide only positive responses.

Facilitator's Guide

The facilitator should provide the capacity standards scoring sheet to the participants and explain how to use it. The facilitator should then use the questions provided against each standard to facilitate clearer understanding of the standards to enable proper scoring.

Basic IPC Capacity Standards for HIV/STI Risk Reduction With HRGs	Questions for the Facilitator
1. All IPC sessions have moved beyond giving messages to the HRGs, and IPC facilitators now involve HRGs in discussion, debate and critical reflection about reducing their risk of HIV/STIs.	■ Do the IPC facilitators provide enough space, security and stimulation to enable HRGs to discuss "their" issues for HIV/STI risk reduction? How?<br>■ Do the IPC facilitators take the participants through the 4 stages of IPC –<br>● Do they pick barriers for analysis from those identified by the HRGs or provide their own list of barriers?<br>● Do they suggest solutions and have discussion on what is acceptable and practical for the HRGs?
2. In all IPC sessions, HRGs are helped by the IPC facilitator to:<br>(1) Analyse their barriers to risk reduction<br>(2) Find acceptable and realistic solutions to these barriers (3) Plan how they will put the solutions into practice.	

Basic IPC Capacity Standards for HIV/STI Risk Reduction With HRGs	Questions for the Facilitator
	• Do they discuss individual/group plans to take the solutions to action?<br>• Do the HRGs share their risk reduction techniques?
3. During IPC sessions, HRGs are always encouraged by the IPC facilitators to share their own practical risk reduction techniques and assess the effectiveness of these techniques.	■ How often do the IPC facilitators need to "push" the discussion rather than facilitate it?
4. The organisation promotes and sustains appropriate values and attitudes for working with HRGs amongst all staff, and particularly amongst IPC facilitators (e.g. they are sensitive to HRG's vulnerabilities, are non-judgmental about sexual practices and lifestyles of HRGs, and work on behalf of the HRGs).	■ Have there been any incidents of IPC facilitators being treated "differently" by the organisation because they belong to a particular HRG?<br>■ Do the IPC facilitators hesitate in introducing themselves as HRGs?<br>■ Do they find some of the sexual behaviours practiced by a HRG member unacceptable?<br>■ Do they feel "different" from other HRG members being in the role of IPC facilitators?
5. IPC facilitators who are selected are acceptable and credible to the HRGs in the site.	■ How does the organisation select, train and support IPC facilitators?<br>■ Is the process of selection transparent and capable of selecting the desired IPC facilitators?<br>■ Do the IPC facilitators face difficulty in mobilising a group for a session?<br>■ Do they enjoy the credibility in the group because HRGs respect them, or is there any other reason for this?<br>■ How many IPC facilitators are true HRGs?
6. IPC facilitators meet regularly to share information and are able to access regular training, supervision and feedback to update their skills and knowledge.	■ Is there a mechanism by which regular interactions take place between the IPC facilitators? What is it?<br>■ How does the organisation identify training needs of IPC facilitators?<br>■ How often are training programmes organised for them?<br>■ How does the organisation provide feedback and supervision?

Basic IPC Capacity Standards for HIV/STI Risk Reduction With HRGs	Questions for the Facilitator
7. IPC facilitators have consistent supplies of condoms and lubricants and other risk reduction commodities and information for demonstration and discussion purposes. They also have sufficient supplies of paper and markers and other materials needed for IPC methods.	■ Do IPC facilitators have adequate supplies to enable smooth implementation of IPC?<br>■ If there is a shortage, what do they do?
8. IPC facilitators are able to make a safe space for the maximum number of HRGs to participate in IPC sessions. They can facilitate sessions in which HRGs feel comfortable to share sensitive issues. They use methods which help HRGs be creative in finding solutions to problems. They work at times convenient to HRGs, and which do not interfere with income generation.	■ Do they know how and when and where to work with HRGs?<br>■ Have there been instances of public interference or opposition or violence during or after the IPC sessions?<br>■ Are the HRGs comfortable discussing sex and sexuality with the IPC facilitators?<br>■ What is the proportion of sessions where the facilitator "does" the method for the group?
9. IPC facilitators keep informed about key HIV/STI risk reduction issues that are relevant for the HRGs in the site. They are able to respond to the hierarchy of HRG needs (e.g. on a continuum from basic prevention skills and knowledge for new entrants to the HRGS community, to VCTC and positive prevention for those who have been around longer).	■ Do the IPC facilitators have a knowledge of most (if not all) of the HIV/STI prevention services available in their site?<br>■ Do the IPC facilitators have a knowledge of most (if not all) of other (than HIV/STI prevention) services available in their site?<br>■ How many referrals do they make every month (average)?
10. IPC facilitators can link HRGs with other prevention services in the site (e.g. STI treatment, VCTC, condoms, lubricant, injecting equipment, counselling, mutual support opportunities, etc.). They are also able to refer HRGs to other services that are important to them (e.g. credit, childcare, education, etc.).	

Basic IPC Capacity Standards for HIV/STI Risk Reduction With HRGs	Questions for the Facilitator
11. The organisation is aware of and able to reach HRGs who are hardest to reach with IPC methods and actively targets new HRGs and those HRGs most at risk.	■ What are the ways by which the IPC facilitators get information about new HRGs?<br>■ What is the ratio of new to old participants in a session?
12. Regular feedback from IPC work in the field helps the organisation to understand the changing needs of HRGs in terms of risk reduction and to adapt other intervention strategies accordingly.	■ How does the organisation learn about changes in the site and about issues that affect the vulnerability of different HRGSs?<br>■ How often do the IPC facilitators meet with the NGO coordinator?<br>■ Does the organisation have committees with significant HRGS representation on them?
13. The organisation contributes to, and learns from, the implementation of a State-level communication strategy using information collected on a regular basis from their IPC monitoring.	■ How is the information gathered from IPC sessions collected from the IPC facilitators and documented by the organisation?<br>■ How has the information collected from the implementation of IPC helped the organisation in tuning their programmes/ interventions to the needs of the HRGs?<br>■ How often does the organisation share the information generated from IPC sessions to other stakeholders, policy makers, etc.?

Step 3 Facilitating the group to determine scores for the basic capacity standards

Copies of the IPC standards should be made for participants to refer to. After the group discussion, participants should form pairs or small groups and determine a score for each IPC capacity standard. When they have done this, the whole group can come together and agree on a final score.
Where there are big differences in scoring between the small groups or pairs, each small group should explain why they gave the score they did. It is important that they then try and reach a consensus on a final score, but where it is not possible, an average of the different small group scores can be taken. Keep a final copy of the standards reflecting the scores after the whole group has shared their thoughts and the discussion has taken place.

Basic IPC Capacity Standards for HIV/STI Risk Reduction With HRGs

Scoring Sheet

These standards are not "indicators" which can be objectively measured; rather they are designed to stimulate discussion in the organisation so that creative ways to improve IPC for HRGs can be found. This means that although an organisation can use the standards to see where it has strengthened its own IPC for risk reduction, the score of one organisation cannot be compared with the score of another organisation.

Scores are designed to indicate the degree of action required in order for each statement to be completely true for the organisation:

DK =Don't know or not applicable
1 = Needs urgent attention
2 = Needs major improvement
3 = Satisfactory, need some improvement.
4 = Satisfactory, need a little improvement.
5 = Exemplary, cannot be improved

Although difficult, a frank and critical approach will mean that the final scores are more meaningful and useful to the organisation. In particular, participants should think carefully before assigning a "5" – is there *really* no room for improvement? Even if the standard is being reached, are there opportunities to improve the *quality* of the work?

Basic IPC Capacity Standards for HIV/STI risk reduction with HRGS	DK	1	2	3	4	5
1. All IPC sessions have moved beyond giving messages to the HRGs, and IPC facilitators now involve HRGs in discussion, debate and critical reflection about reducing their risk of HIV/STIs.						
2. In all IPC sessions, HRGs are helped by the IPC facilitator to:<br>(1) Analyse their barriers to risk reduction<br>(2) Find acceptable and realistic solutions to these barriers<br>(3) Plan how they will put the solutions into practice						
3. During IPC sessions, HRGs are always encouraged by the IPC facilitators to share their own practical risk reduction techniques and assess the effectiveness of these techniques.						
4. The organisation promotes and sustains appropriate values and attitudes for working with HRGs amongst all staff, and particularly amongst IPC facilitators (e.g. they are sensitive to HRG's vulnerabilities, are non-judgmental about sexual practices and lifestyles of HRGs, and work on behalf of the HRGs).						
5. IPC facilitators who are selected are acceptable and credible to the HRGs in the site.						
6. IPC facilitators meet regularly to share information and are able to access regular training, supervision and feedback to update their skills and knowledge.						
7. IPC facilitators have consistent supplies of condoms and lubricants and other risk reduction commodities and information for demonstration and discussion purposes. They also have sufficient supplies of paper and markers and other materials needed for IPC methods.						
8. IPC facilitators are able to make a safe space for the maximum number of HRGs to participate in IPC sessions. They can facilitate sessions in which HRGs feel comfortable to share sensitive issues. They use methods which help HRGs be creative in finding solutions to problems. They work at times convenient to HRGs, and which do not interfere with income generation.						

9.	IPC facilitators keep informed about key HIV/STI risk reduction issues that are relevant for the HRGs in the site. They are able to respond to the hierarchy of HRG needs (e.g. on a continuum from basic prevention skills and knowledge for new entrants to the HRG community, to VCTC and positive prevention for those who have been around longer).						
10.	IPC facilitators can link HRGs with other prevention services in the site (e.g. STI treatment, VCTC, condoms, lubricant, injecting equipment, counselling, mutual support opportunities, etc.). They are also able to refer HRGs to other services that are important to them (e.g. credit, childcare, education, etc.).						
11.	The organisation is aware of and able to reach HRGs who are hardest to reach with IPC methods and actively targets new HRGs and those HRGs most at risk.						
12.	Regular feedback from IPC work in the field helps the organisation to understand the changing needs of HRGs in terms of risk reduction and to adapt other intervention strategies accordingly.						
13.	The organisation contributes to, and learns from, the implementation of a state-level communication strategy using in formation collected on a regular basis from their IPC monitoring.						

Step 4: Facilitating the group to analyse capacity needs

After scoring against the standards has taken place, participants should identify where the organisation is strong, and areas where capacity needs to be strengthened.

Ask the participants to focus their attention on the capacity standards which have been scored between 1 and 3:

- Think about capacity gaps that need immediate/urgent attention (score 1).
- Think about the low capacity areas that need major improvement (score 2).
- Think about the average capacity areas that need some improvement (score 3).

Discuss:

- What action can be taken?
- How can that action be taken?

- How urgent is the action?
- Who will take responsibility for this?
- Do we need external help or is this something we can do ourselves?
- Are there any resources that could help us with this?
- Write up the findings using the planning table format below. Include any actions carried over from the previous plan if one was made.
- Discuss what the next steps should be:
  - Deadline for finalisation and distribution of the report (SACS/PSU, other partners, etc).
  - Follow-up on the actions agreed.
  - Discuss how the findings of the analysis will be shared with other staff and stakeholders.
  - Decide when the next bi-annual analysis of IPC capacity will take place.

**Need** Capacity gap identified	**What?** Action needed	**When?** Now / in the next 2 months / in the next 6 months	**Who?** List people to be involved	**Resources required**

Step 5 Compiling a report of the basic IPC capacity analysis

The Basic IPC capacity standards report should include the following sections:

1. Organisation name and date of report
2. Overall conclusions from the session
3. Final capacity standards score sheet
4. What has improved in the last 6 months? (If a similar exercise was done in the past)
5. Capacity strengthening plan
6. Signature of NGO Director or representative

The report should provide a succinct and clear summary of the findings of the capacity analysis and have the final capacity standards scores and the capacity strengthening plan attached for review during the next self-analysis.

A copy of the report should be kept in the organisation and one should be sent to the SACS/PSU. The organisation may or may not want to share it with other partner organisations.

ANNEXURE 6b

Dialogue-Based Interpersonal Communication By and with HRGs

Day 1, Session 1.1<br>Introduction	
**Objectives of the session**	1. To welcome the participants to the workshop.<br>2. To enable participants and facilitators to get to know each other.<br>3. To know the expectations of the participants and match them with the objectives of the workshop.<br>4. To introduce the workshop agenda.<br>5. Process of the workshop – participatory.<br>6. Energisers are learning too.<br>7. To set ground rules for the course of the workshop.<br>8. To explain housekeeping arrangements.<br>9. To organise how participants can review the workshop on a daily basis.
**Process**	**Welcome address**<br>1. Thank SACS and the participants for their interest and setting aside time for the workshop.<br>2. Introduce the Project & role of SACS, NGOs, PEs, PATH.<br>3. Introduce facilitating team.<br>4. Clarify language/translations.<br>5. Terminology (HRGs, IPC, etc).<br>6. Consent process for taking photographs.
	**Introducing ourselves**<br>Nickname game
	**Expectations**<br>1. Participants are asked to decide in pairs one thing each that they bring to the workshop and one thing they expect to take away.<br>2. The pairs report back in plenary and facilitator sums up the group's contributions and expectations.<br>3. Facilitator explains what the workshop objectives are and takes the participants through the broad agenda.
	**Ground rules**<br>Flowers & Thorns
	**Daily review teams**

Day 1, Session 1.2<br>Introduction to Dialogue-Based IPC	
**Energiser**	Paper folding game
**Objectives of the session**	1. To introduce dialogue-based IPC to the participants.<br>2. To help participants understand the risks and vulnerability factors that are barriers to HIV prevention.<br>3. To help the participants understand how IPC methods help to move beyond just giving prevention messages to helping HRGs address barriers to prevention.
**Process**	1. Instructions for the method:<br>■ Do the HRG Drawings exercise (Method 5 in Annexure 6a).<br>2. Participants' feedback on:<br>■ The process of the method: the steps, the discussion opportunities, ease of use, etc.<br>■ The role of HRG Drawing in identifying the risk behaviours which are barriers to HIV prevention for HRGs.<br>■ The role of HRG Drawing in helping to analyse and address barriers to HIV prevention with HRGs.<br>3. Explain the 4 phases in IPC:<br>a) HRGs identify their barriers to HIV/STI prevention<br>b) HRGs analyse their barriers<br>c) HRGs find practical ways to address their barriers<br>d) HRGs plan and take action<br>4. Participants in small groups discuss why it is important to take the HRGs through all the 4 phases. The groups also design a "logo" showing the 4 phases in IPC.<br>5. Screening of the IPC film followed by discussion.
**Material**	Used A4 sheets for the energiser, Charts, Markers, IPC Film
**Handouts**	IPC definition

Day 1, Session1.3<br>Introduction to Dialogue-based IPC Framework	
**Objectives of the session**	1. To introduce the participants to the IPC framework<br>2. To help participants understand the importance of the components of IPC framework.
**Process**	1. Plenary presentation of the IPC framework.<br>2. After the presentation the participants are divided in to 6 groups and each group is given one component of the IPC framework.<br>3. The participants are asked to prepare 2 important arguments for their component to be the most important one in IPC.<br>4. After having discussed within the group the participants debate in the plenary the importance of their respective component.<br>5. The facilitator later ends the session by highlighting the equal importance of every component in the IPC framework.<br>Facilitator shares the possible outcomes of dialogue-based IPC in the plenary.
**Handouts**	IPC framework

Day 1, Session 1.4<br>Introduction to IPC Method — Services Map	
**Objectives of the session**	1. To introduce the participants to another IPC method.<br>2. To show the importance of moving beyond messages to action.
**Process**	1. Facilitator explains the method:<br>■ Do the HIV/STI Services Map exercise (Method 11).<br>■ Essential steps of IPC<br>■ In a plenary brainstorming activity the participants are asked to list the essential steps to be followed, this activity is followed by presentation of essential steps in doing IPC to the participants<br>2. Participants' feedback on:<br>a) The process of the method-the steps, the discussion opportunities, ease of use<br>b) The role of Services Maps in identifying the barriers to services for HIV prevention and treatment for HRGs
**Material**	Charts, markers

Day 2, Session 2.1<br>Facilitation Skills	
**Energiser**	**E-game**<br>Write a large curvy letter E on a piece of paper and place it in the centre of the circle. Ask participants what they see on the piece of paper from where they are sitting/standing. Depending on where they are in the circle, they will either see an 'm', a 'w', a '3' or an 'E'. Participants can move places so that they can see the letter from a different perspective. Ask the participants whether they see different things from different places.
**Objective of the session**	To help participants understand what is good facilitation in dialogue-based IPC
**Process**	**1. Critical Reflection**<br>■ Get the participants to stand in a circle facing outwards so that they can't see each other. Read out the list of behaviours and instruct the participants to raise both hands if they do the behaviour often, 1 hand if they do it sometimes and no hands if they never do that behaviour while working with groups of peers.<br>a) I take over the group and lead the discussion.<br>b) I interrupt others to make my point.<br>c) I disengage with those group members strongly disagreeing with each other.<br>d) I encourage others to contribute to the discussion.<br>e) I avoid discussion on topics I don't have knowledge on.<br>f) I give limited information when I have less time on hand.<br>g) I cut short the discussions when I have less time on hand.<br>h) I allow the more vocal members of the group to lead the discussion.<br>i) I end discussions with a follow up plan.<br>j) I find it OK to conduct the discussion wherever I find a group of people.<br>■ After the exercise put the following questions in the plenary:<br>a) What do you mean by facilitation?<br>b) What skills does a good facilitator need to have?<br>c) What should we avoid when facilitating IPC methods with our peers?<br>d) How do people learn facilitation skills?<br>**2. Questioning Skills**<br>■ Small groups prepare 5 examples of "good" questions and 5 of "bad" questions. These examples are discussed in the larger group to understand what makes a good question (one that can help in discussion) and what makes a bad question (one that can upset/ disturb the discussion).
**Material**	A big curvy 'E' on a chart of paper, Charts, Markers

Day 2, Session 2.2<br>IPC Method - Why Is It So?	
**Objective of the session**	1. To introduce the steps in facilitating IPC methods.<br>2. To introduce the participants to a third IPC method.
**Process**	1. Facilitator presents the 11 essential steps in dialogue-based IPC.<br>■ The facilitating team role plays all the 11 steps while facilitating the "Why Is It So?" exercise (Method 9). The participants are asked to observe the role play and identify and comment on each of the 11 steps.<br>2. After the role play, participants give feedback on:<br>a) The process of the method the steps, the discussion opportunities, ease of use<br>b) The role of "Why Is It So?" in identifying the risks and vulnerability factors which are barriers to HIV prevention for HRGs<br>c) The role of "Why Is It So?" in helping to analyse and address barriers to HIV prevention with HRGs
**Material**	Charts, markers

Day 2, Session 2.3<br>IPC Method – How Hot Is the Spot?	
**Energiser**	Drawing Bricks
**Objectives of the session**	1. To introduce the participants to the IPC methods.<br>2. To help the participants to facilitate the IPC method with their peers.
**Process**	1. After explaining the method the facilitator asks a group of participants to volunteer to role play facilitation of the exercise "How Hot is the Spot?" (Method 12). Each volunteer should be given a specific role by the facilitator (someone to dominate the discussion, someone to be the saboteur, someone who is not interested in the discussion, etc.). The rest of the participants are asked to observe the role play and map the dynamics in the group: communication, body language, participation, etc.<br>2. After the volunteers have role played "How Hot is the Spot?", all participants share the group dynamics they observed to facilitate a discussion by the facilitator on how an IPC facilitator can manage group dynamics

Day 2, Session 2.4<br>Fieldwork	
**Objective of the session**	To help participants practice facilitating IPC methods in the field.
**Process**	1. The facilitator shares the objectives of the fieldwork.<br>The participants are divided into fieldwork teams. Each team is be supported by a HRG consultant during the fieldwork.<br>2. Important things to remember for the facilitator:<br>■ Team division<br>■ Fieldwork site information/details<br>■ Contact details<br>■ ID cards/authorisation letters<br>■ Material for fieldwork

Day 3, Session 3.1<br>Fieldwork Experience Sharing	
**Objectives of the session**	1. To share the fieldwork experience (and check gaps in facilitation).<br>2. To help the participants identify and address any challenges to using IPC methods.
**Process**	1. The teams are asked to discuss in their team and present:<br>a) Learning from the field<br>b) Challenges faced<br>c) Suggestions for overcoming those challenges<br>2. The facilitator gives some time for the teams to prepare feedback on the following points and then asks each point one by one to the teams:<br>a) Experience of facilitating all the IPC methods shown so far (HRG Drawing, Services Map, Why Is It So?, How Hot Is the Spot?)<br>b) Critical reflections on their facilitation skills<br>c) Experience of using all the steps in facilitating the IPC methods<br>d) How they worked as a team

Day 3, Session 3.2<br>IPC Method – Graffiti	
**Objectives of the session**	1. To introduce the participants to another IPC method.<br>2. To update participants' HIV/STI knowledge.
**Process**	1. Facilitator facilitates Graffiti exercise (Method 6).<br>2. Participants give feedback on:<br>a) The process of the method: the steps, the discussion opportunities, ease of use<br>b) The role of Graffiti in identifying the risks and vulnerability factors which are barriers to HIV prevention for HRGs<br>c) The role of Graffiti in helping to analyse and address barriers to HIV prevention with HRGs<br>3. The facilitator opens the floor for questions/doubts/concerns on HIV/STI which the facilitator and the participants respond to.

Day 3, Session 3.3<br>IPC methods - Margolis Wheel	
**Objective of the session**	1. To introduce the participants to another IPC method.<br>2. To help the participants to identify mechanisms for taking action on barriers to risk reduction with HRGs
**Process**	1. The facilitator explains the method and then facilitates the participants in 2 groups to do the Margolis Wheel exercise (Method 4).<br>2. Participants give feedback on:<br>a) The process of the method: the steps, the discussion opportunities, ease of use<br>b) The role of Margolis Wheel in identifying the risks and vulnerability factors which are barriers to HIV prevention for HRGs<br>c) The role of Margolis Wheel in helping to analyse and address barriers to HIV prevention with HRGs<br>3. After this feedback, the facilitator discusses the possible role of a peer educator in an HIV prevention project with a special mention to his/her role "promoting action". The facilitator then picks a couple of solutions generated from the Margolis Wheel exercise and divides the participants into small groups. Groups are asked to identify:<br>a) How the solution can be taken to action<br>b) Who would be involved<br>c) What their role would be

Day 3, Session 3.4<br>Fieldwork (longer duration)	
**Objective of the session**	To help participants practice facilitating IPC methods in the field.
**Process**	1. The facilitator shares the objectives of the field work.<br>2. The participants are divided into field work teams; each team is be supported by a HRG consultant during the fieldwork.<br>3. Important things to remember for the facilitator:<br>■ Team division<br>■ Field work site information/details<br>■ Contact details<br>■ ID cards/authorisation letters<br>■ Material for fieldwork

**Day 4, Session 4.1**<br>**Fieldwork Experience Sharing**	
**Objective of the session**	1. To share the fieldwork experience (and check gaps in facilitation).<br>2. To help the participants identify and address any challenges to using IPC methods.
**Process**	1. The teams are asked to discuss in their team and present:<br>■ Learning from the field<br>■ Challenges faced<br>■ Suggestions for overcoming those challenges<br>2. The facilitator gives some time for the teams to prepare feedback on the following points and then asks each point one by one to the teams:<br>■ Experience of facilitating all the IPC methods shown so far (HRG Drawing, Services Map, Why Is It So?, How Hot Is the Spot?, Graffiti, Margolis Wheel)<br>■ Critical reflections on their facilitation skills<br>■ Experience of using all the steps in facilitating the IPC methods<br>■ How they worked as a team

**Day 4, Session 4.2**<br>**Other IPC Methods – Body Mapping, Statue, Lovers**	
**Objective**	To provide an overview of some other IPC methods.
**Process**	1. Divide the participants into 3 groups. Each group is shown one of the 3 IPC methods. They are asked to practice the method shown and then teach the other groups to facilitate it.<br>**IPC Method: Body Mapping** (Method 7 above)<br>**IPC Method: Statues** (Method 3 above)<br>**IPC Method: Lovers** (Method 8 above)<br>2. Facilitator conducts a plenary discussion on how best to mentor other peer educators in facilitating dialogue-based IPC<br>3. Ask the participants to share their learning from the session on training peer educators to become IPC facilitators

**Day 4, Session 4.3**<br>**Next Steps**	
**Objective of the session**	To help participants plan next steps for putting IPC into practice in their everyday work and lives.
**Process**	Small groups plan their next steps.

Day 4, Session 4.4<br>Workshop Evaluation	
**Energiser**	Cat & Mouse
**Objective of the session**	1. To learn lessons about how this training could be improved.<br>2. To see what capacity building needs the participants feel they still have.
**Process**	1. Small groups discuss and feedback on 3 evaluation questions:<br>▪ What they feel was most useful about the workshop<br>▪ What they feel could be improved the next time this workshop is conducted<br>▪ What they feel they need more training on in order to facilitate IPC effectively with their peers<br>2. End the workshop with the **Gift game**.<br>▪ Starting with the facilitator, each person gives an imaginary gift to the person on their right.

ANNEXURE 7

Crisis Response System

Rationale for Crisis Management

Harassment and violence towards sexual minorities is common and is a significant barrier to targeted interventions towards key HIV affected populations. Harassment may include verbal abuse, arrest on false charges (e.g. of solicitation or for carrying condoms), beatings and even sexual assault. Harrassment and abuse may come from the general public, police, goondas, local leaders, clients, or from within the HRG itself.

When the obstacle of violence and harassment is removed through timely and proper crisis response and regular sensitisation and advocacy programs, an environment is created that supports members of the HRG in building up their self-esteem. This in turn helps them to focus more on their health and specifically issues relating to sexually transmitted infections (STIs), including HIV/AIDS.

As part of a TI, crisis response interventions increase outreach to members of the HRG, thereby strengthening the NGO's or CBO's relationship with them and gaining their trust. Crisis response also facilitates the establishment of a good rapport between field workers and members of the HRG, which helps communication about prevention and treatments of STIs.

Essential Ingredients of Effective Crisis Management

- Trained and committed staff members who are willing to be "on call" 24 hours a day and to respond immediately when a crisis happens
- Effective communication mechanisms (i.e. crisis phones) that the community can contact
- Availability of information about crisis response to community members
- Experienced and committed lawyers who are willing to provide assistance 24 hours a day
- Networking, alliance-building, and sensitisation work with local stakeholders (especially the HRG) through regular meetings and education as appropriate. This includes community-level legal literacy sessions
- Close alliances with other civil society organizations, activists and local media contacts who can advocate on behalf of the community when necessary
- Reflections on crisis management cases to improve and build internal capacities

Establishing a Crisis Response System

The following steps can be taken to establish a crisis response system:

1. A crisis management team is established. This should consist of peer educators, outreach workers, senior project staff, and legal resource persons familiar with the legal issues surrounding harrassment of FSWs. The team establishes detailed protocols for staffing and procedures of the crisis response system, and is responsible for implementing these.

2. Mobile phones are obtained to be used exclusively for community members to call in case of a crisis. The project should have at least 1-2 mobiles available, although in a large urban setting the ideal ratio is at least 1 phone for ~1,000 population.
3. Nominated community members volunteer to manage these phones for crisis management. These members may change every month so that a pool of crisis managers develops and no volunteers are over-burdened
4. Crisis mobiles are never switched off. Volunteers undertake to be available 24 hours a day to respond to a crisis. Many crises happen at night, and the crisis team and project staff should be ready to respond even at odd hours.
5. All crisis mobile numbers are widely circulated within the community through practical, pocket-sized crisis cards printed in the local language as well as English. The card lists the mobile phone numbers and describes the kind of crisis management that the NGO/CBO offers to the community.

The Crisis Response System in Action

1. When a community member calls the crisis number on her own behalf or on behalf of another member who has been harrassed or abused, the member of the crisis management team responding to the call immediately gets in touch with other crisis team members to apprise them of the situation. Depending on the nature of the crisis, and according to the criteria for senior staff and legal response established by the team, the crisis team members may inform senior project staff, including the project coordinator and legal resource person.
2. The team ensures that at least one person from the crisis team goes to the spot where the crisis has happened and meets the person concerned. Any crisis should be responded to within 30 minutes of its being reported. It is important to provide immediate moral support and give the message that the person is not alone in this situation and that the person has support from the project.
3. If a police report needs to be filed, or if the situation involves arrest or the person affected is at the police station for any other reason, a team member and in addition a legal resource person should reach the police station within 30 minutes.
4. Every crisis is documented to record the kind of crisis, perpetrators and response. A formal documentation system can be used to show an increase or decrease in the number or type of crisis cases, and the nature of responses to crises. This information can be used both to strategise for improving crisis response, and for public advocacy.
5. Weekly debriefing meetings are held with the crisis management teams to discuss any crises that have happened during the week, followed by collective brainstorming on strategies for improving the crisis response.

Examples of Crisis Intervention Materials

Below are reproduced an information card and documentation form for a crisis intervention programme targeting MSMs and TGs in Bangalore, operated by the NGO Sangama. The format of these materials can be adapted for other HRGs such as FSWs and IDUs.

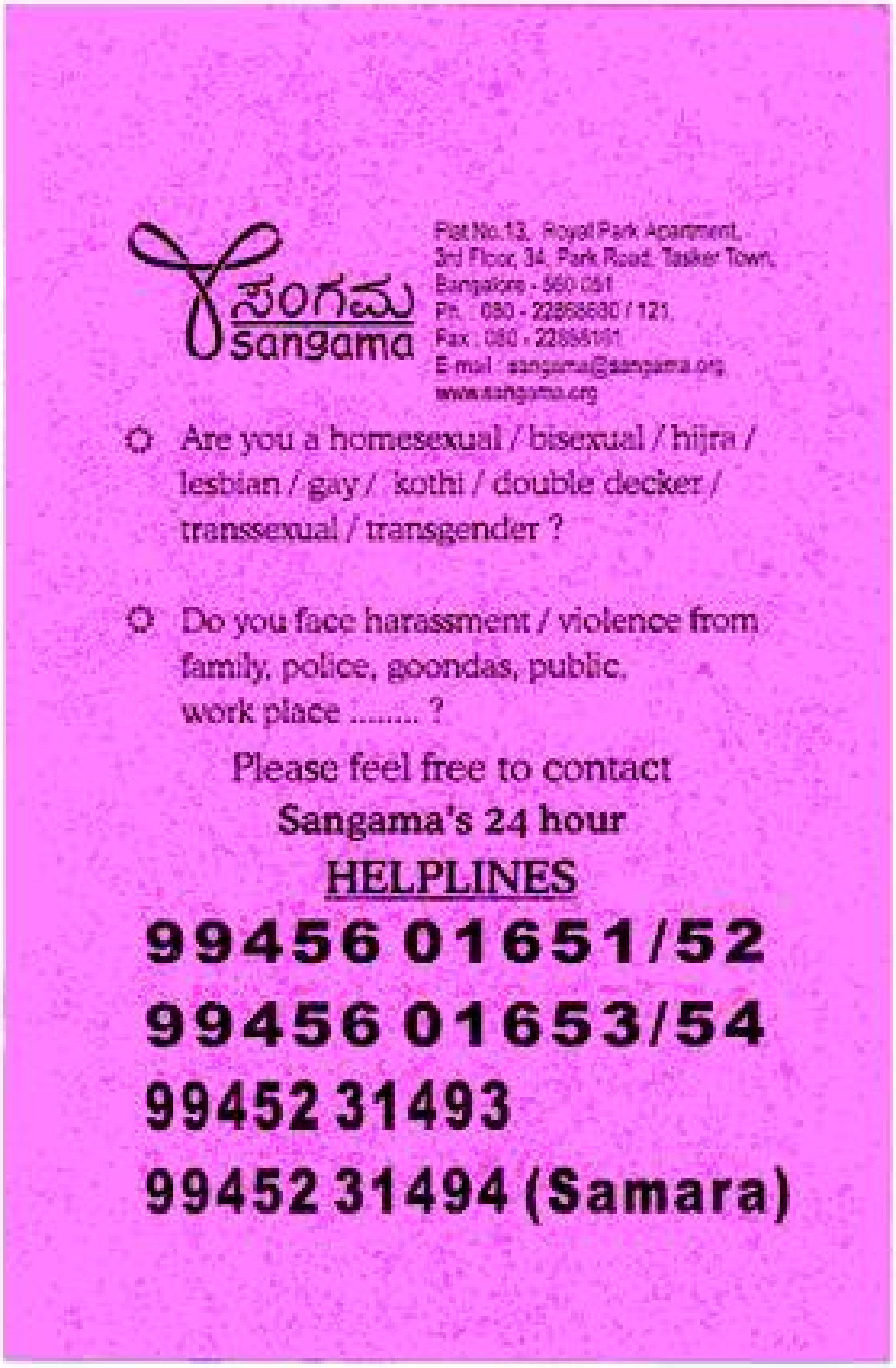

ಸಂಗಮ
Sangama

Flat No.13, Royal Park Apartment,
3rd Floor, 34, Park Road, Tasker Town,
Bangalore - 560 051
Ph. : 080 - 22868680 / 121,
Fax : 080 - 22868161
E-mail : sangama@sangama.org
www.sangama.org

- Are you a homesexual / bisexual / hijra / lesbian / gay / kothi / double decker / transsexual / transgender ?
- Do you face harassment / violence from family, police, goondas, public, work place .... ?

Please feel free to contact
Sangama's 24 hour
HELPLINES

99456 01651/52
99456 01653/54
99452 31493
99452 31494 (Samara)

Format: Situation Report on Community Harrassment/Abuse

This situation report allows for simple and comprehensive documentation of incidents of harrassment or abuse against members of a HRG. Full documentation is essential for the purposes of legal reponse and community advocacy. By following the format of the situation report, the crisis intervention volunteer will not omit information that needs to be collected while events and memories are fresh.

In this example, the HRG is MSM, but the report can easily be adapted for work with FSWs by replacing the MSM typology with that of FSWs (street-based, brothel based, *dhaba*-based, home-based, etc.) and by adding any relevant categories for the specific situation of the TI.

**Situation Report on Community Harrassment / Abuse**					
**Report Serial #**			**District**		
**Abuse against:** Individual: Yes/ No			Group: Yes/ No		
**Who was harrassed / abused? (Type)**					
Hijra	Yes	No	Panthi	Yes	No
Kothi	Yes	No	Pimps	Yes	No
DD	Yes	No	Partners/lovers	Yes	No
Clients	Yes	No	Other (Please Specify)	Yes	No
**Who harrassed / abused the above? (If possible record the name(s) of the abuser.)**					
Rowdies	Yes	No	Community members	Yes	No
Police	Yes	No	Family	Yes	No
General Public	Yes	No	Partner	Yes	No
Clients	Yes	No	Others (Please Specify)	Yes	No
**Date of Incident**	Date		Month	Year	
**Time of Incident**	Time		AM/ PM		
If an individual was harrassed/abused, please record their name/registration number.					
**If Group, please fill in the number of people who were abused and their type, and if possible their names/registration number.**					
**Incident Details (Include kind of abuse and extent of damage. Where did the incident happen (Location, police station area etc.)? If physical injuries were sustained, please record.**					

Date and time the project made its first response to the incident through its staff.<br>Date Month Year Time AM/ PM<br>Action taken by the office/staff (was a report filed, was the abused person taken to hospital, etc.?).
Follow-up actions to be taken
Date the issue was resolved completely<br>Date Month Year<br>**Report filed by** **Date**
Any incident should be recorded in which the target community, staff and the associates of sexual networks, etc. were beaten up, arrested, raided or suffered any other form of abuse, including extortion and forced sex, whether by the police, rowdies, clients or the general public.
This report must be filed on the day the incident happens. Best practice is that the report is shared immediately with the programme manager. It is expected that if the case is genuine, partner will take necessary response in coordination with the District HIV/AIDS committee or any other relevant commitee/individual. Responses could include an FIR, personal visit to the affected person/group, and in the case of beatings the provision of medical aid, medical report, evidence gathering, photographs etc., as well as discussions with the community and building of response strategies. The underlying principle is to make abuse reporting and action a routine activity and ensure that the response is not ad hoc.

ANNEXURE 8

Community Committees

Rationale for Community Committees

Community Committees (CCs) are a model for empowerment of high risk groups (HRGs) as well as a key tool for effective provision of services. As such, they should be formed in close consultation with members of the community, and the structures, roles and responsibilities of the committees and their members should be developed by the NGO/CBO jointly with the community members.

A CC is based in each intervention location (NGO) with one representative from each site. The committee acts as a monitoring agent for the programme in each location and holds periodic meetings to address issues that arise.

Effort is required to bring people together, build trust and encourage participation on the part of the community. Community Committee members (CCMs) should represent the different typologies of sex workers so that each group's interests are sustained. They should be rotated every 3 to 6 months so that the maximum number of community members has an opportunity to serve.

Objectives of the Community Committee

- Identifying the needs of the key population members in their area
- Helping members of high risk groups attain goals of health, socio-economic empowerment and improved quality of life
- Assisting in planning and implementation of the programme
- Working on advocacy, legal help and issues such as prevention of trafficking
- Creating demand for quality STI and HIV/AIDS services
- Motivating members of HRGS to have regular medical checkups
- Organising cluster-level events such as a Women's Day and an annual day on other issues which affect the life of FSWs
- Promoting the collectives of the community and strengthening them
- Promoting Self Help Group formation

Method of Functioning

- CCs meet once every fortnight; the venue and time are fixed by members for their convenience. A meeting can be conducted only with 60% attendance
- The CCs maintain an attendance register, minutes book and follow-up file of their meetings and activities
- A Community Advisor participates in one CC meeting each month as an observer
- The minutes of the meeting are given to the NGO
- The project/NGO may take action in the programme or respond to issues based on the recommendations of the CC

Community Committee Members (CCMs)

A Community committee member (CCM) is an elected representative of 100 members of the key population, and/or may be elected from a particular site. Qualifications include:

- Good communication skills, good relations with peers and a commitment to the Community Committee process
- Willingness to attend all the trainings and meetings of CC and to participate actively in all CC activities
- A CCM can be removed by citing justifiable reasons, e.g. poor attendance, poor participation, involvement in unlawful activities

NGO Sub-Committees

Community members may be part of various sub-committees at the NGO level. These sub-committees are responsible for the various components of the NGO's activities, e.g. STI clinic, Inter-personal Communications (IPC) and Ethics/Grievances. It may be found optimal to have three community members on each sub-committee. Ideally, one committee member from each sub-committee is available on a daily basis to carry out the duties of their respective committee.

Examples of three NGO sub-committees and their roles and responsibilities are given below.

STI Committee

Objective

To create an environment for smooth functioning of the clinic, motivating the community members to seek proper diagnosis and facilitating IPC for STI knowledge.

Roles and Responsibilities

- Ensure cleanliness of the clinic
- Create rapport with referral centres (e.g. Voluntary Counselling and Testing Centres and Care Centers)
- Facilitate hospitality before and after check-up
- Motivate the community member for speculum / proctoscopy / lab investigation
- Participate in periodic review meetings with clinic staff

IPC Committee

Objective

To facilitate IPC sessions for community members and co-ordinate preparation of new IPC materials and other IPC-related activities. The IPC committee members should have a good knowledge and interest in IPC, preferably be literate and have good communication skills. They should be able to operate and maintain audio-video systems.

Roles and Responsibilities

- Maintain the IPC materials stock and distribution with help of the NGO
- Disseminate IPC messages
- Give training to other community members in usage of IPC materials
- Document IPC sessions in proper formats
- Get feedback from community on existing IPC materials and make suggestions for new materials

Ethics/Grievances Committee

Objective

To ensure that basic ethics and the values of the NGO are followed in all its activities. It may also facilitate a 24-hour response system for issues that arise, disseminate various data and ensure proper utilization of services.

Roles and Responsibilities

- Respond to any issues with regard to "respect for the community"
- Ensure that the community is not misused for personal gain
- Respond to issues related to the provision of services by the NGO
- Prevent and address any problems that may arise out of religion or caste among the community
- Coordinate with other committees on ethical matters
- Ensure appropriate confidentiality on all matters regarding the community
- Maintain values in research
- Take up any specific grievances of community members for discussion and resolution

ANNEXURE 9

Power Analysis

CONTENTS

Rationale

Guidelines for Using the Tool

Exercise 1 Are We One Community?

Exercise 2 Daily Routine

Exercise 3 Organizational Mapping

Exercise 4 Sexual Practices, and Risks We Face

Exercise 5 Issues Associated with Type of Sex Work

Exercise 6 Group Discussion of the Meaning and Dynamics of Power

Exercise 7 Identifying Bodies of Control

Exercise 8 Role Playing the Bodies of Control

Exercise 9 Incidents of Raids, Violence and Rescue

Exercise 10 Changeability vs. Impact: Challenging Practices

Rationale

This tool is based on participatory methodology and contains a set of exercises that helps vulnerable communities examine their lives, the risks they are constantly exposed to, the institutional actors and stakeholders that interplay significantly to control their lives and their environment and the power nexus that sustains this control. Beginning with mapping their daily routine, the tool allows the group to run through a systematic analytical process. This culminates in insight into necessary action as a group to change the landscape of power and control that dominate their lives. The process takes on momentum and allows for optimal output when a few simple guidelines are followed.

The tool when run fully takes 3 days. However, it is designed to allow facilitators and/or groups to choose portions that best suit them at a given point of time. The tool can be run using basic material such as chart papers and marker pens.

Guidelines for Using the Tool

General Guidelines

- **Facilitators:** It is ideal to have a combined team of facilitators comprising programme/project staff and senior community members.
- **Group size:** A group of 15-20 participants is an ideal size.
- **Preparation:** It is essential for the facilitators to go through the tool carefully as a group before running it with the participants.
- **Recapturing the process:** The group of facilitators must meet at the end of each day to recapture the process and outcomes. A good analysis of this will serve as the basis for taking the analytical process forward.
- **Recording the output:** Outputs can be generated using large sheets of card or paper. Coloured cards can also be used to facilitate the process of participatory analysis. As and when possible, digital recording of generated outputs is helpful. Outputs generated must be put together in a structured report.
- **The tool is a guide, not a set of rules:** The tool is an illustrative guide and the matrices in it are outputs meant to facilitate data collation, encourage analysis and prepare for action. If you can think of a better way of reaching the goals of understanding the bodies and the means of control, go for it!
- **Facilitative and empowering, not extractive:** The tool attempts to facilitate concicentisation and empowerment and to lead to action by the community groups. Any data produced is meant primarily for the community themselves. To use this tool for the purpose of extracting data would defeat its purpose.

Guidelines for Conducting Participatory Structural Assessments

- **Look, listen and learn.** Facilitate. Don't dominate. Don't interrupt. When people are mapping, modeling or diagramming, let them focus.
- **Give adequate time.** Participants should be given ample time to think and discuss before replying.
- **Embrace error.** We all make mistakes, and do things badly some times. Don't hide it. Share it.
- **Ask yourself –** who is being met and heard, and what is being seen, and where and why; and who is not being met and heard, and what is not being seen, and where and why?
- **Relax.** Don't rush. Allow unplanned time to walk and wander around.
- **Meet people** when it suits them, and when they can be at ease, not when it suits you. This applies even more strongly to women than to men.
- **Probe**. Interview the map or the diagram.
- **Ask about what you see.** Notice, seize on and investigate diversity, whatever is different, the unexpected.
- **Use the six helpers –** who, what, where, when, why and how?
- **Ask open-ended questions.**
- **Show interest** and enthusiasm in learning from people. Be sensitive to people.

Guidelines for Conducting Role Plays

Dramatic enactment, or role play as it is popularly known, is a particularly compelling and efficient technique for presenting a situation. A brief dramatic presentation reveals not only the problem but also the context in which it exists.

Role plays have the following advantages:

- They allow for safe rehearsal of skills and activities, and provide practical options for real-life situations.
- The trainees are able to experience activities and to relate theory to practice.
- They allow for full expression and interpretation of concepts.

While role plays serve as a good learning methodology, they have to be conducted with skill and caution. Optimal outputs can be achieved only when basic guidelines are followed:

- The facilitator should introduce the activity to the participants, clearly explaining the objective of the exercise and the desired outputs.
- Clarify any queries or doubts raised by the group.
- Check if there is anyone who is uncomfortable and/or unwilling to participate.
- Identify and address the issues being raised by the participant.
- Further to this, if any participant chooses to opt out of the exercise, allow her/him to do so.
- Explain clearly the situation or theme on which the enactment is to be built.
- Communicate clearly the time allotted for preparation and enactment.

- While the groups are preparing, be around to clarify doubts and queries being raised.
- Watch out for any discomfort amongst participants at all stages of the exercise.
- The effectiveness of a role play is dependent on the discussions emanating from it. The facilitator must channel the discussion to meet the objective of the exercise.
- Ensure that efforts put in by participants are recognised.
- After all role plays have been presented and discussed, debrief participants.

Guidelines for Conducting a Group Discussion

Group discussion in the context of this tool is a verbal interaction between the group members (may or may not include the team of facilitators) on a specific theme/situation.

Group discussions may be conducted in the larger group or within smaller sub-groups. Depending on the situation, the facilitator plays the following role:

- Allow maximum space for participants to express ideas and opinions.
- Ensure participation by all members in the group.
- Unobtrusively moderate the discussion to remain within the framework/objective of the exercise.
- Take note of and bring up relevant points in further discussions.
- While providing space for diverse opinions to be expressed, the facilitator must constantly watch out for signs of disruption and moderate effectively.
- Address myths and/or factual inaccuracies identified during discussions.
- Keep time and help closure.
- Reiterate conclusions emerging out of the discussion and steer the process forward.
- When running small group discussions, ensure that groups report to the larger group to exchange findings and further develop their ideas.

Note on the Use of Brainstorming

Brainstorming is a technique for generating innovative and creative ideas from the facilitator and the group. It is a useful technique for gaining an overview of a subject before narrowing it down into practical ideas. Often solutions which would not normally occur to people will be brought out through brainstorming in a group.

The technique stimulates everyone to participate and gives the facilitator an idea of the experience of the group. It enables the maximum amount of experience, training and ideas to be shared in the minimum time. The facilitator must take care to ensure that all participants get an opportunity to express themselves.

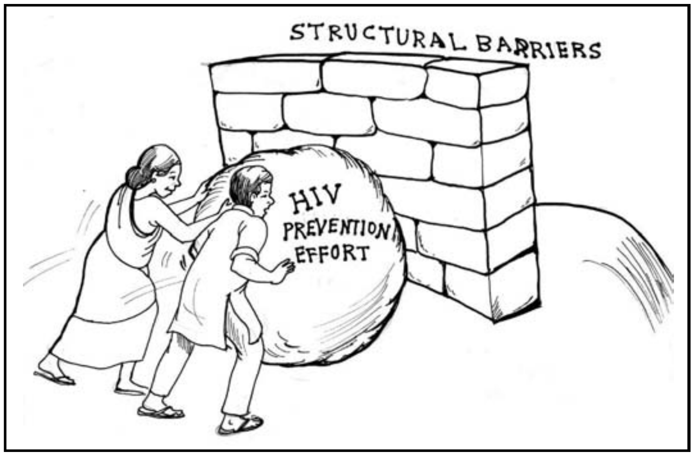

Exercise 1 Are We One Community?

This is a discussion-based session and requires active facilitation. The objective is to help the group understand the word “community” and to recognize the inherent homogeneity amongst themselves that makes them a “community”. This process can be run in different ways depending on the skill and experience of the facilitator. What follows is a description of a process that has been used across different groups and regions in the six high HIV prevalence states.

- The discussion is initiated by asking participants to share their understanding of the word “community”.
- The responses routinely include a variety of social groups and situations based on religion, occupation, caste, geographical location, etc.
- The facilitator leads the group to understand the key feature of a community in the context of the tool/process: “A group of people having a common belief or ideology, value systems, problems and interests.”
- This introduction sets the tone for beginning the exercises with a group which has similar concerns.

> A community can often constitute a very diverse set of individuals who are identified under one category or label based on one or more of the features that define the community. In such a case, the group may not necessarily see itself as a community. An example of this is FSWs. FSWs hail from different backgrounds, religions, etc. The feature of commonality is the occupation they practise. The nature of the occupation is such that it fosters shame and does not naturally allow congregation. Basic acceptance of self is however essential for FSWs to own their life patterns and problems.

Exercise 2 Daily Routine

Objective

This is the next exercise of the tool and profiles the daily routine of individuals. It helps individuals take a look at an "average day" in their lives and the transactions they make during it. The exercise serves as a non-threatening starting point for a group hitherto unfamiliar with systematic and analytical processes. The exercise helps build rapport between members of the group and sets the tone for further analysis.

Process

1. Participants are asked to list activities that they undertake during any regular day.
2. They determine the schedule of activities that the majority of the group follows.
3. Differing schedules due to variability in the group must be acknowledged and recorded. Before beginning the process of recording, the facilitator must allow sufficient time for discussions and encourage participants from within the group to undertake the recording. There is a format provided but groups are free to express themselves innovatively.

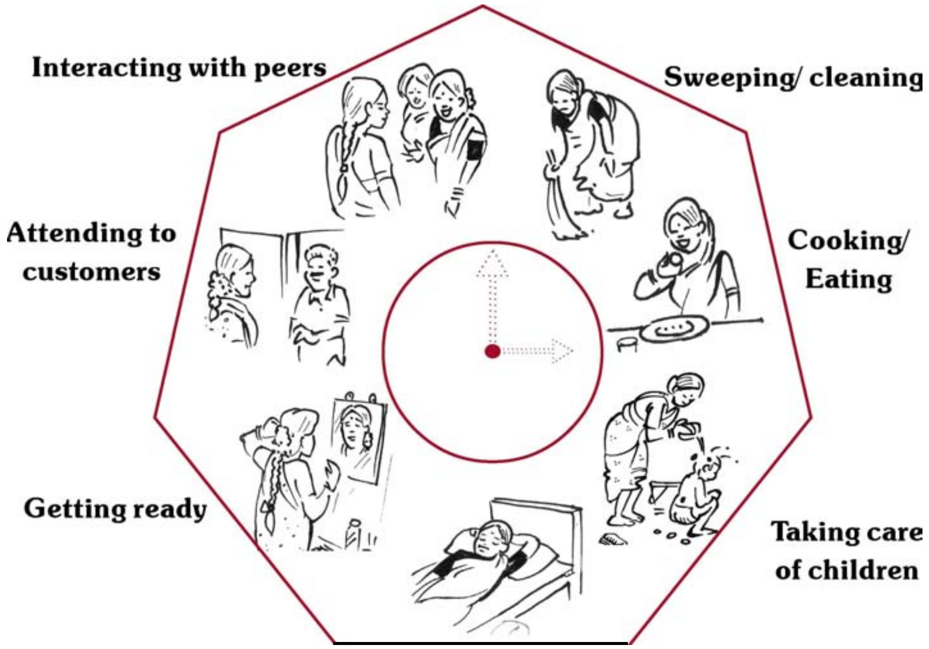

Resting

Average Day in the Life of an FSW

Facilitator's note:

Members within the group may show variability in terms of daily patterns depending on type of sex work (e.g. street- or brothel-based). Depending on the nature and size of the group, the facilitator can use this diversity in various ways to enhance the process of analysis. Smaller groups can be assigned one subcategory to be worked on.

Exercise 3 Organizational Mapping

Objective

The purpose of this exercise is to map out the services and institutions in the neighbourhood that have a bearing on people's lives.

This can be done through a simple Chapati diagram. This tool provides a very simple and visual way of letting participants describe where they place different entities, institutions, social groups, persons, or concepts in relative importance to their lives. The visual distance of the entities from the centre denotes accessibility, interaction and comfort level, and thus their influence on the daily lives of these groups.

Process

- Ask the community members to list the services/institutions that are available to them in the neighborhood. What are the objectives/services provided by the institution/ organisation.
- Ask them to place these at varying distances from the centre which denotes the community, to indicate how accessible and available the services are. For example, the Municipal Hospital is shown far from the FSW community since it is not perceived as accessible by the community.
- After the community has drawn the map, ask them to list the factors that determine the accessibility of the services. For example, the Municipal Hospital is also shown faraway because the doctors have a negative attitude towards FSWs, and hence the hospital is perceived as relatively inaccessible in this respect too.
- What is the attitude of each of these structures towards "community".
- What kind of impact do these organisations/services have on their lives?

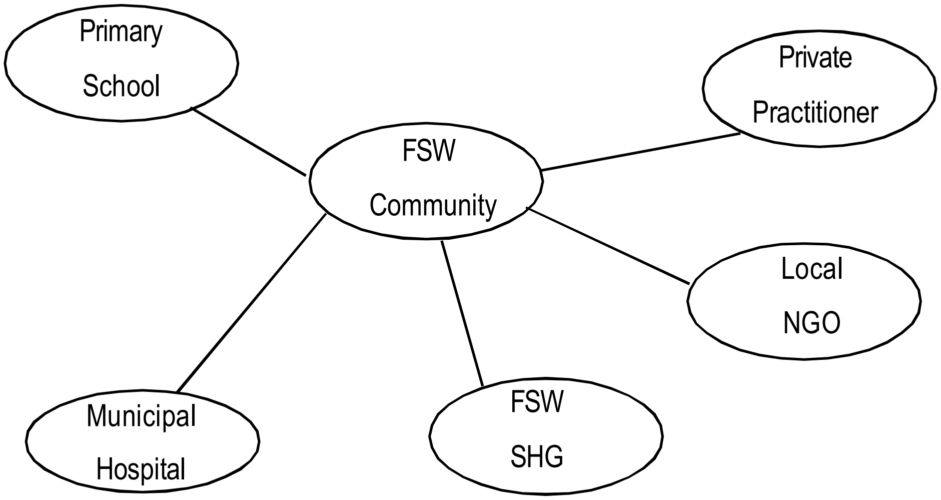

Illustrative Output

Name of the NGO/ group/institution	Objectives/ activities	Operational area	Impact	Attitude towards group/NGO

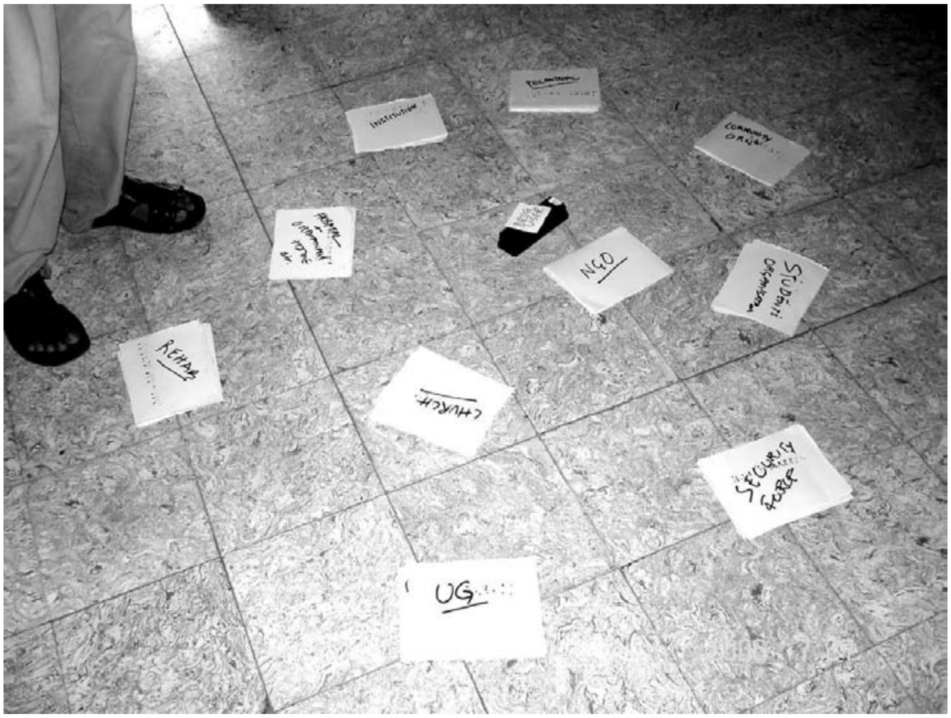

An illustrative output from an exercise with a group of drug users

Exercise 4 Sexual Practices, and Risks We Face

Objective

The objective of this exercise is to help groups identify the various types of sexual practices they undertake, the people with whom they are undertaken and the risks associated with such practices.

This analysis helps groups identify the amount of control they have over each of these practices and helps set in motion a thought process to address the risks they face.

Process

The participants are asked to conduct the process using the following steps:

- List all known sexual practices being used along with the locally used words/slang for each of them.
- For each listed sexual practice, participants are asked to examine and document the following:
  - Place where the practice normally occurs.
  - Method or manner in which the practice is conducted and who it is undertaken with. (E.g. a certain sexual practice is undertaken perhaps only for a regular client.)

Exercise 5 Issues Associated with Type of Sex Work

Objective

This is an important exercise in terms of understanding risk and vulnerability in sex work. The exercise tries to locate the issues/advantages/problems that exist within the different forms of sex work, e.g. brothel-based, street-based, highway-based and home-based.

Process

- Ask the participants to get into groups according to the type of sex work that they belong to, e.g. brothel-based, street-based, highway-based and home-based.
- Ask each group to list the issues / advantages / problems associated with each of these forms of sex work.

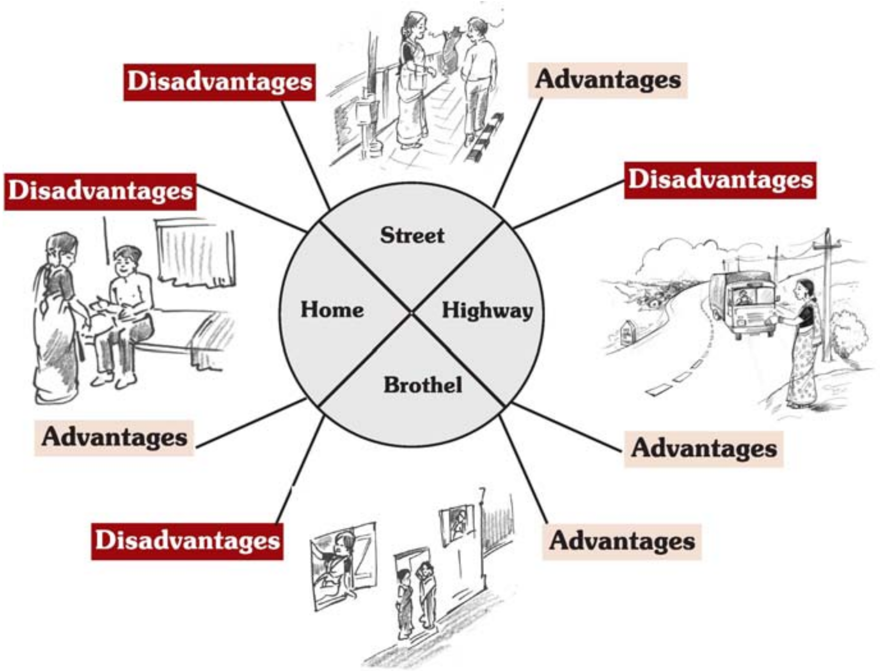

Facilitator's Note:

Each category of sex work throws up advantages as well as disadvantages. If participants experience a hurdle, it might be helpful to suggest looking at the power brokers in specific environments.

Exercise 6 Group Discussion of the Meaning and Dynamics of Power

Objective

This session is central to the entire process and needs to be conducted with skill. The facilitator is required to play an active role and take the group through a discussion process clarifying the concept of power.

Process

The framework described here will help the facilitator take the group through this process.

1. Group's understanding of the word "power" – includes both the meaning of the word and words used to represent it by the group, maybe in local language.
2. Sources and basis of power, through examples which the group can relate to.
3. Expression of power, through examples which the group can relate to.
4. Power places and spaces for participation.
5. Contextual and historical nature of power.
6. Individual versus group power.

This discussion sets the stage for intensive analysis of the dynamics of power that operate in the life of an FSW and ultimately enhance their vulnerability to HIV and co-infections, among other issues.

Exercise 7 Identifying Bodies of Control (Who Has the Power?)

Objective

This is the most crucial step in the process of analysis. It involves listing all the possible people, groups, institutions, etc. as perceived by the FSWs, that affect their lives in either positive or negative ways. *These are the groups that possess power over the lives of the community members.*

Process

Explain to the group the meaning of the phrase "bodies of control". This could be defined as the people or structures that exert influecne in their professional and personal lives.

Ask them to free list all the possible "bodies".

Examine the output with focused questions, e.g.:

- Why are these important to the lives and profession of FSWs?
- Are all the positive players really positive?
- Are all the negative players really negative?

Facilitator's Note

It is important to remember that a given stake holder can exert both a positive as well as a negative influence on the lives of people.

Exercise 8 Role Play: Bodies of Control

Used in the context of this tool, the role play should be carried out using the following guidelines:

- Randomly divide the larger group into three or four smaller groups depending on the bodies of control that have been prioritised in Exercise 7.
- Each group is assigned one body of control.
- The group is asked to discuss and develop a situation depicting a transaction that routinely occurs with this body of control.
- Each group is allowed 30 minutes to prepare the role play and 10 minutes to enact the situation.
- Each role play is followed with a discussion focused on eliciting the following:
  - Sphere of control
  - Means used to control
  - Source of control
  - The nexus that helps maintain control

Facilitator's Note

The facilitator may find it helpful to refer to the role play guidelines given at the beginning of the tool.

Exercise 9 Incidents of Raids, Violence and Rescue

Objective

- This exercise attempts to list the incidents of raids, violence and rescue that routinely occur in the life of FSWs. After listing, there can be discussion and analysis of incidents, intentions and problems related to raids, violence and rescue.

Process

- Ask the group to list the incidents that they think qualify as incidents of raids, violence and rescue.
- List these instances and mark their occurrence in the last 6 months.
- Note the final outcome that each of these incidents brought about. For example, after a rescue operation of minor girls, some of them returned to their native villages while some returned to sex work.

RAIDS

Illustrative Output 10A

No. of sex workers in the exercise:

Free list of practices, elements of raids	Place of raid	Occurrence in the past 6 months	Outcome
Police raid for rescuing minor girls			
Police picking up FSWs above 18 years of age			

VIOLENCE

Illustrative Output 10B:

No. of sex workers in the exercise:

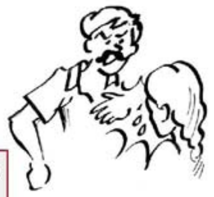

Free list of practices, elements of Violence	Place of Violence	By Whom?	Occurrence in the past 6 months	Outcome
Forced sex	Brothel	Customer		
Physical abuse, beating	Street	Sex Worker/ Brothel keeper		

RESCUE

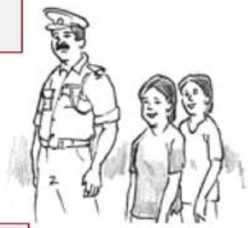

Illustrative output 10C :

No. of sex workers in the exercise:

Free list of practices, elements of Rescue	Place of raid	By Whom?	Occurrence in the past 6 months	Outcome
Rescuing minor girls with police cooperation	Brothel	Local NGO	twice	Girls sent back to their native villages
Forcible 'rescue' of sex workers who are not minors.	brothel	JJB	once	Girls pay hefty bribes to be rescued

Exercise 10 Changeability vs. Impact: Challenging Practices

Objective and Process

This exercise will help the groups to decide which practices they want to challenge based on their changeability, and the kind of impact that could be achieved by this change. Some of the practices that can be changed may have a very small impact, and some which may have high impact will require a lot of effort over a sustained period of time. The community and the project must choose the changes that they want to bring about starting with the more visible and easier, and then graduate to strategic and bigger changes. They can use the sources of power and risks to decide on the steps required for making the changes.

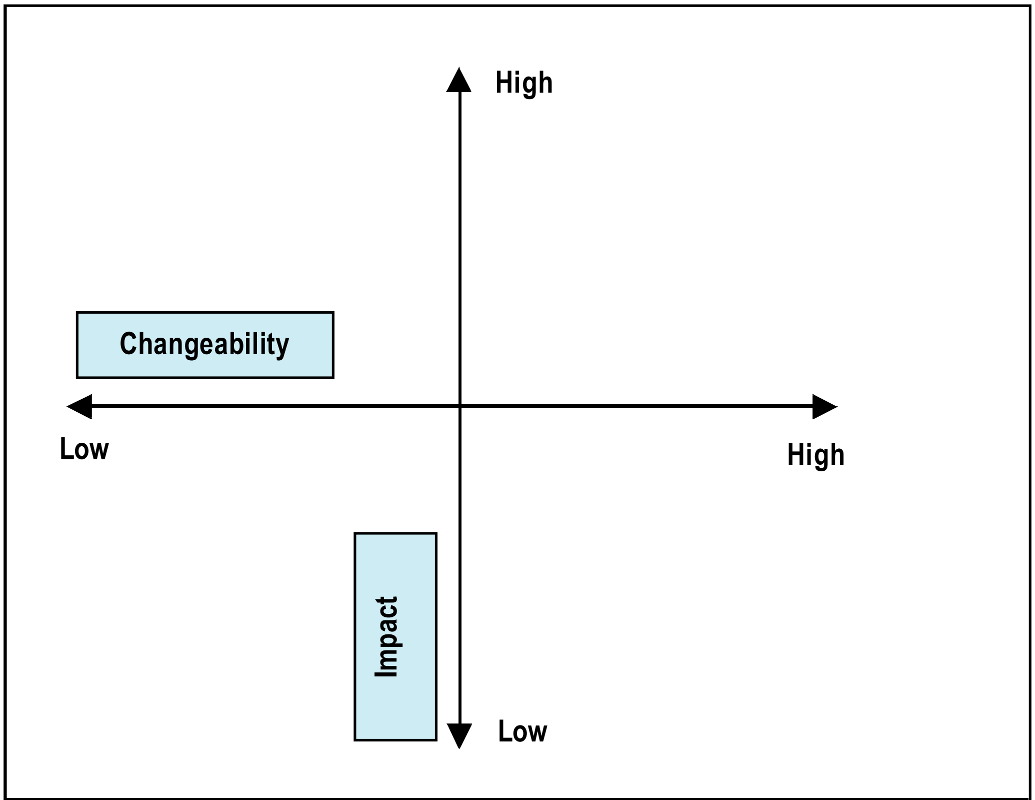

Change priorities	Steps required for this quarter	Responsibility	Allies

ANNEXURE 10

Programme Management

Programme Component	1. Mapping and Enumeration
Prerequisites	1. Mapping budgets released to SACS<br>**2.** TSU established and staffed<br>**3.** NGO contracted and staffed (project coordinator, outreach workers, community guides) - *only for site validation*
Input	1. NACO participatory mapping protocols<br>2. NACO site verification and profiling tools
Output	1. State mapping report with estimated numbers of each HRG with population split by District, site, hotspot and typology (in Year 1 and Year 3)<br>**2.** HRG denominator targets for each TI in the State (targets to be revised annually based on validation process)

Activities	Primary Responsibility		
	SACS	TSU	NGO
Secondary data review to identify locations where mapping is required			
Along with Mapping TRG and State level mapping agency, conduct broad mapping of State and sites identified in desk review			
Oversee agency field work during mapping exercise and provide technical / management support			
Collate mapping data to identify a) potential intervention sites, b) list of hotspots within sites, c) sise of HRG population at each site and hotspot by typology (female sex workers, MSM, TG, IDU) and sub-type (e.g. FSW could be brothel-based, street-based, lodge-based, home-based, bar-based, MSM could be kothi, double decker, panthi, etc.)			
Review findings of mapping exercise			
Approve and publish mapping data for the State in collaboration with State level mapping agency			
**Site Validation (from month 1 onward)**			
Validation and profiling of each intervention site by joint team of outreach worker and community guides (max of 2-4 weeks / site). Key outcomes include:<br>1) Finalised intervention denominator and risk profile by site - HRG population sise, typology split, hotspot distribution, client volume and condom use estimates<br>2) One-on-one meetings with at least 50% of HRG denominator at site<br>3) Create familiarity and acceptability for the project among HRG<br>4) Identify potential peer educators			
Collate site validation results to finalise NGO-level denominator (sise estimates for intervention)			
Periodically (at least every year) update TI coverage targets in the State based on current validated data available from TI NGOs			
Fix denominators for all TIs in the State (review and update at least every year)			

Major Milestones/ Targets	H1	H2
Year 1	**1.** State and District mapping covering entire State completed, reports finalised and published<br>**2.** 100% of NGOs validate mapping numbers for all sites through HRG-led process<br>**3.** Denominators fixed for all TIs in the State (basis mapping data and subsequent validation)	
Year 2	**1.** Mapping numbers at all sites validated by TI NGOs and denominators for TIs revised accordingly	
Year 3	**1.** State and District re-mapping covering entire State completed, reports finalised and published<br>**2.** 100% of NGOs validate re-mapping numbers for all sites through HRG-led process<br>**3.** Denominators for TIs revised basis new numbers<br>**4.**TIs added / reduced as required based on new information	
Year 4	**1.** Mapping numbers at all sites validated by TI NGOs and denominators for TIs revised accordingly	
Year 5	**1.** Mapping numbers at all sites validated by TI NGOs and denominators for TIs revised accordingly	

Programme Component	2. Staff Recruitment and Intervention Start-up<br>*(STI clinic staff covered under STI section)*		
**Prerequisites**	1. TI coverage area and denominator fixed<br>2. TSU contracted and fully staffed<br>3. NGO contracted and funded as per NACO guidelines		
**Input**	1. NACO NGO HR Policy<br>2. Annexure 3, *Peer Educator Training*<br>3. Annexure 2, *Site Assessment*		
**Output**	1. Validated site profiles<br>2. NGO staff and peers fully staffed and trained		

Activities	Primary Responsibility		
	SACS	TSU	NGO
Recruit NGO project staff (non-clinic staff)<br>1) Project coordinator - 1 per intervention<br>2) Outreach workers in the ratio 1 per 250 HRG to be covered by the interventions (TSU support for recruitment, optional)<br>3) Accountant - 1 per intervention<br>4) Office support staff - 1-2 per intervention<br>5) Community guides - 1 HRG member per outreach worker (temporary position only purpose to conduct site validation before peers are hired)			
Training of NGO staff on introductory topics for intervention<br>1) Basic induction on HIV/AIDS<br>2) Understanding FSW/community dynamics of sex work<br>3) Skills in identifying and building rapport with FSW<br>4) Site validation methodology			
**Site Validation (from month 1 onward)**			
Validation and profiling of each intervention site by joint team of outreach worker and community guides (max of 2-4 weeks / site). Key outcomes include:<br>1) Finalised intervention denominator and risk profile by site - HRG population sise, typology split, hotspot distribution, client volume and condom use estimates<br>2) One-to-one meetings with at least 50% of HRG denominator at site<br>3) Create familiarity and acceptability for the project among HRG<br>4) Identify potential peer educators			
Collate site validation results to finalise NGO-level denominator (size estimates for intervention)			

Peer Recruitment (from month 3 onward)	SACS	TSU	NGO
Recruit peer educators from the HRG community (as per NACO peer selec tion guidelines) in the ratio of 1 peer per 60 HRG under intervention coverage. A few critical considerations for peer selection *(see section 3.3.1.D for a detailed list)* :<br>1) Should be active HRG members<br>2) Should belong to the predominant typology of HRG at the hotspot<br>3) Should have responsibility to cover hotspots within one site<br>4) Should have wide networks in the site to be covered<br>5) Will receive an honorarium for time commitment to project			
Selected peers trained as per peer capacity bui lding curriculum identified in TI guidelines			

Major Milestones/ Targets	H1	H2
Year 1	**1.** 100% of NGO staff recruited and trained as per TI guidelines<br>**2.** 100% of sites validated by outreach worker - community guide team	**1.** 100% of sites have recruited and trained peer educators as per NACO peer selection guidelines
Year 2	Maintain previous levels	
Year 3	Maintain previous levels	
Year 4	Maintain previous levels	
Year 5	Maintain previous levels	

Programme Component	3. Infrastructure Set-up<br>(STI clinic setup covered under STI section)
Prerequisites	1. TI coverage area and denominator fixed<br>2. TSU contracted and fully staffed<br>3. NGO contracted and funded as per NACO guidelines
Input	1. Validated site profile information
Output	1. Basic infrastructure established

Activities	Primary responsibility		
	SACS	TSU	NGO
Basic infrastructure setup (during first three months prior to peer identification)			
Conduct community consultations to identify suitable location and premises for safe spaces or drop in centers (DIC) at all loca tions with HRG sise >=500			
Provide free condom distribution through outreach workers and community guides to HRG met during consultations and other meetings			
Establish short-term referral services to STI doctors (to fill service vacuum until full-fledged services are set up)			
Infrastructure Scale-up (after peer recruitment)			
Conduct community consultations to identify suitable location and premises for safe spaces or drop-in centres (DIC) at all loca tions with HRG sise >=100<br>(At smaller locations safe space can be rented for partial duration of a day if full-time facilities not available)			

Major Milestones/ Targets	H1	H2
Year 1	1. Safe spaces established in consultation with HRG at 100% of sites with HRG population >=500<br>2. Outreach workers and guides delivering basic outreach (free condom supply and STI referrals) at 100% of sites with HRG population >=500	1. Safe spaces established in consultation with HRG at 100% of sites with HRG population >=100
Year 2	Maintain previous levels	
Year 3	Maintain previous levels	
Year 4	Maintain previous levels	
Year 5	Maintain previous levels	

Programme Component	4. Outreach Planning
Prerequisites	1. TI coverage area and denominator fixed (including sites, hotspots and target group) per mapping report<br>2. NGO contracted and funded as per NACO guidelines<br>3. NGO outreach staff (project coordinator, outreach workers, community guides) recruited to cover intervention area as per staffing guidelines<br>4. Site validation process completed<br>5. Basic project infrastructure established (project offices, drop-in centres, clinics)<br>6. Critical commodities available (Condoms, STI drugs, BCC material)<br>7. Peer educators from HRG recruited to cover all sites as per peer selection guidelines
Input	Annexure 5, *Peer Led Outreach and Planning*
Output	1. Hotspot level microplans for each peer<br>2. Site level work plans for outreach workers

Activities	Primary Responsibility		
	SACS	TSU	NGO
Adapt NACO specified microplanning tools and guidelines for local use	■	■	
Train TI project staff (project coordinator, field officers, outreach workers and peer educators) to use microplanning tools		■	
Implement Phase 1 of microplanning (spot analysis, contact mapping, geographic and social networks) at all sites to initiate first-time contact with target HRG population and identify the "most at risk" individuals in each peer's network			■
Weekly/bi-weekly review meetings with peers to develop individual plans for regular contact (Phase 2 of microplanning) based on information collected through Phase 1 tools and peer formats (peer education card, calendar and individual-level tracking)			■
Monthly meetings between outreach staff and peers to 1) plan for raising service levels using Phase 3 microplanning tools and 2) update information captured in Phase 1 and Phase 2 tools as per any changes in the field			■
Six-monthly "opportunity gaps" analysis to improve service delivery and coverage of HRG			■
Review quality (accurate, up to date) of peer microplanning tools and formats and provide technical support to raise peer capacity to use and analyse data from peer formats<br>**(Outreach workers to do joint outreach and analysis of formats with each peer at least two days every week)**			■
Review quality of microplans and provide on-site technical support to peers and outreach workers to implement microplanning and use information to prioritise outreach and service delivery to "most at-risk" and "least served" populations<br>**(TSU staff to do joint field outreach and analysis of formats for at least five days per NGO per month)**		■	

Major Milestones/ Targets	H1	H2
Year 1	**1.** 50% of TI NGO staff and peers trained on use of microplanning for outreach	**1.** 100% of TI NGO staff and peers trained on use of microplanning for outreach<br>**2.** At least 50% of sites implemented Phase 1 and Phase 2 mi croplanning tools and peer formats with acceptable quality as per TSU review (i.e. peers are able to identify and direct services to the riskiest and neediest individuals in their respective networks)<br>**3.** 100% of NGOs contact at least 40% of target denominator through peers every month<br>**(each contact to consist of at least 1 to 1 BCC session and condom distribution)**
Year 2	**1.** 100% of sites implemented Phase 1 and Phase 2 microplanning tools and peer formats with acceptable quality as per TSU review<br>**2.** 100% of NGOs meeting at least 60% of target denominator through peers every month	**1.** 100% of sites implemented full suite of microplanning tools (Phase 1, 2 and 3) and peer formats with acceptable quality as per TSU review<br>**2.** 100% of NGOs meeting at least 80% of their target denominator through peers every month<br>**3.** 100% of NGOs have completed opportunity gap analysis to improve service delivery and coverage
Year 3	**1.** 100% of NGOs have robust microplanning systems in place to identify and meet new HRG individuals within three months of entering a site<br>**2.** Maintain previous levels on all other aspects	**1.** Maintain previous levels on all other aspects
Year 4	**1.** Maintain previous levels	**1.** Maintain previous levels
Year 5	**1.** Maintain previous levels	**1.** Maintain previous levels

Programme Component	5. STI Services (includes staffing and infrastructure setup)
Prerequisites	1. TI coverage area and denominator fixed (including sites, hotspots and target group) per mapping report<br>2. NGO contracted and funded as per NACO guidelines<br>3. NGO outreach staff (project coordinator, field officers, outreach workers, community guides) recruited to cover intervention area as per staffing guidelines<br>4. Site validation process completed<br>5. Peer educators from HRG recruited to cover all sites as per peer selection guidelines
Input	NACO *STI Guidelines* and *Tool for STI Approach*
Output	1) STI service coverage plans for each TI<br>2) STI service delivery to HRG as per plans<br>3) STI technical support and quality monitoring systems established to cover all TIs

Activities	Primary responsibility		
	SACS	TSU	NGO
Adapt NACO specified STI guidelines and tools for local use			
**Staffing and establishing infrastructure for STI services**			
Recruit STI technical officers at TSU-level to provide technical support and quality monitoring for STI services delivered by TIs<br>**(At least 1 STI technical officer for every 20 clinics or every 3 Districts - whichever is smaller)**			
Conduct consultations with HRG groups at all sites to:<br>**1)** determine current health seeking practices<br>**2)** identify list of preferred physicians for each HRG<br>**3)** decide optimal mode for STI service delivery at the site (intervention clinics - static or outreach, referrals to public or private sector)			
Establish physical infrastructure (premises, equipment, utility connections) for clinics after consultations with HRG to identify convenient, accessible sites<br>1) Static intervention clinics at all sites with >= 1000 HRG/site or with high risk profile as determined by the TSU<br>2) Outreach clinics - fixed day, fixed time - for smaller sites with high-risk profiles			
Recruit adequate staff at intervention clinics (STI physicians, counsellors and ANM) as per NACO STI management guidelines<br>**(Where feasible select qualified HRG members for appropriate roles)**			
Establish referral clinic services (with public or private sector as preferred by HRG) to cover smaller sites (=< 200 HRG/site)			
Establish logistics network for supply of STI drugs, condoms and other consumables to all clinics			

Linkages (NGOs to establish linkages with technical support from TSU; TSU to monitor quality of linkages)			
Establish working referral linkages at each clinics (ideally HRG members accompanied by NGO staff or peers) for ICTC, care and support and ART services			
Establish working referral linkages at all clinics with District RNTCP for TB screening and treatment			
Establish working referral linkages at all clinics with laboratories for syphilis screening and testing with appropriate quality assurance systems<br>**(Sites with >2000 HRG should set up facilities for serologic testing of syphilis at intervention clinics)**			
Establish working linkages for other services prioritised by the HRG community (medical termination of pregnancy, child delivery etc) subject to availability of budgets			
**Training**			
Finalise STI capacity building requirements and establish linkages with NACO-approved national/regional institutions for training			
Train intervention clinic staff (physicians, counsellors, ANM) and key NGO staff on STI management, clinic operation and reporting as per NACO guidelines - to be repeated every year with updated curriculum as required<br>**(special emphasis on attitudinal orientation while delivering services to marginalised HRG)**			
Train referral clinic physicians and other key staff on STI management, clinic operation and reporting as per NACO guidelines - to be repeated every year with updated curriculum as required<br>**(special emphasis on attitudinal orientation while delivering services to marginalised HRG)**			
**Monitoring**			
Finalise STI clinic reporting formats and train clinic staff and key NGO staff (project coordinator, field officers, outreach workers) on clinic data collection, analysis and reporting into CMIS			
Periodic data entry of clinic forms into CMIS			
Weekly coordination meetings between clinic and outreach staff at all sites to analyse clinic service data and plan for raising service utilisation and monitor follow-ups **(Clinic data critical input for effective microplanning)**			
Regular visits - at least every two months - to each clinic by TSU STI technical officers (at least 2 days/visit to intervention clinics, 1 day/visit to referral clinics) to assess:<br>1) Adherence to technical standards<br>2) Quality of services and linkages<br>3) Clinic utilisation and coordination with outreach services<br>4) Reporting compliance<br>5) Community orientation of clinic staff (especially to prevent stigmatising behavior toward HRG by clinic staff)<br>6) Community representation in clinic staffing (to be increased over time as community capacity increases)			
Quarterly analysis of STI data to track at least the following<br>1) Utilisation trends by clinic,<br>2) STI syndrome profiles and appropriate treatment<br>3) Uptake of regular checkups			
Random review visits to major clinics by SACS STI officer every six months			

Major Milestones/ Targets	H1	H2
Year 1	**Clinic functioning**<br>**1.** 100% of required STI technical officers (as assessed by NACO guideline of 1 STI officer every 20 clinics or 3 Districts) recruited and trained by TSU<br>**2.** Regional capacity building institutions identified and contracted<br>**3.** NACO STI guidelines adapted for State use<br>**4.** Health-seeking behaviour consultations with HRG conducted at 100% of sites<br>**5.** Intervention static clinics established - including infrastructure, staff recruitment and training and commodity provision - at all suitable sites identified by TSU team and NGOs	**Clinic utilisation**<br>**1.** 5 % of HRG denominator accessing clinic services every month<br><br>**Clinic functioning**<br>**2.** Outreach clinics established - including infrastructure, staff recruitment and training and commodity provision - to cover all suitable sites identified by TSU team and NGOs<br>**3.** 100% of static clinics following syndromic case management as assessed by TSU STI technical officer visits<br>**4.** 100% of static clinics developed referral directories and established referral linkages for<br>*a)* voluntary HIV testing and counselling (with full confidentiality) and<br>*b)* care and support<br>**5.** 100% of static and outreach clinics have functioning paper-based reporting formats (data updated and reported every month)<br><br>**Clinic monitoring**<br>**6.** 100% clinics visited by TSU STI technical team at least thrice during the six month period and provided with technical support and guidance as required (to be continued for the rest of the project cycle)
Year 2	**Clinic utilisation**<br>**1.** 10% of HRG denominator accessing clinic services every month<br><br>**Clinic functioning - Maintain previous levels. In addition:**<br>**2.** Referral clinics established - providers contracted, trained and commodity provision - at all suitable sites identified by TSU team and NGOs<br>**3.** 100% of outreach clinics following syndromic case management as assessed by TSU STI technical officer visits<br>**4.** 100% of outreach clinics developed referral directories and established referral linkages for<br>a) voluntary HIV testing and counselling (with full confidentiality) and<br>b) care and support<br><br>**Clinic monitoring. Maintain previous levels. In addition**<br>**5.** At least 5% clinics visited by NACO/SACS team for random quality checks (to be repeated every six months)	**Clinic utilisation**<br>**1.** 15% of HRG denominator accessing clinic services every month<br><br>**Clinic functioning**<br>**2.** 100% of clinics (static, outreach and referral) have functioning paper-based formats (data updated and reported every month)<br>**3.** 100% of static and outreach clinics and at least 50% of referral clinic data entered into CMIS every month<br>**4.** 100% of referral clinics established referral linkages for<br>a) voluntary HIV testing and counselling (with full confidentiality) and<br>b) care and support<br>**5.** 70% of referral clinics following syndromic case management as assessed by TSU STI technical officer visits<br>**6.** 100% of static and outreach clinics established linkages for TB screening and treatment and OI, ART provision<br>**7.** Annual quality audits rolled out at 100% clinics by TSU staff<br>**8.** 100% of clinic staff (including physicians, counsellors, ANM) covered by refresher trainings during the year (to be repeated every year)<br><br>**Clinic monitoring. Maintain previous levels**

Year 3	**Clinic utilisation**<br>**1.** At least 20% of HRG denominator accessing services every month<br>**2.** At least 50% of HRG denominator underwent regular STI check-up during these six months<br><br>**Clinic functioning. Maintain previous levels. In addition:**<br>**3.** 100% of clinics established mechanisms for syphilis screening and treatment<br>**4.** 100% of clinics providing asymptomatic treatment as specified in NACO guidelines<br>**5.** 100% of clinics have at least one HRG member on their staff<br>**6.** 100% of NGOs established clinic committees with HRG representation to oversee quality and function of clinics<br><br>**Clinic monitoring. Maintain previous levels**	**Clinic utilisation**<br>**1.** At least 25% of HRG denominator accessing STI services every month<br>**2.** At least 75% of HRG denominator underwent regular STI check-up during these six months<br>**3.** At least 50% of HRG denominator screened for syphilis (and all positive treated)<br><br>**Clinic functioning**<br>**4.** 100% of referral clinics established linkages for TB screening and treatment and OI, ART provision<br>**5.** 100% of clinics conduct repeat annual quality audit and show improvements on key parameters<br>**6.** 100% of all clinics entered data into CMIS every month<br><br>**Clinic monitoring. Maintain previous levels**
Year 4	**Clinic utilisation. Maintain previous levels. In addition**<br>**1.** At least 80% of HRG denominator screened for syphilis during these six months (and all positive treated)<br>**2.** At least 80% of HRG denominator undergo verbal screening for TB symptoms (and all identified as potential candidates referred for sputum screening and follow up treatment)<br><br>**Clinic functioning. Maintain previous levels.**<br><br>**Clinic monitoring. Maintain previous levels**	**Clinic utilisation**<br>**1.** At least 30% of HRG denominator accessing STI services every month<br>**2.** At least 90% of HRG denominator underwent regular STI check-up during these six months<br>**3.** At least 90% of HRG denominator screened for syphilis (and all positive treated)<br>**4.** At least 90% of HRG denominator undergo verbal screening for TB symptoms (and all identified as potential candidates referred for sputum screening and follow up treatment)<br><br>**Clinic functioning. Maintain previous levels.**<br><br>**Clinic monitoring. Maintain previous levels**
Year 5	**Clinic utilisation. Maintain previous levels**<br><br>**Clinic functioning. Maintain previous levels**<br><br>**Clinic monitoring. Maintain previous levels**	**Clinic utilisation. Maintain previous levels**<br><br>**Clinic functioning. Maintain previous levels**<br><br>**Clinic monitoring. Maintain previous levels**

Programme Component	6. Condom Distribution
Prerequisites	1. TI coverage area and denominator fixed<br>2. TSU contracted and fully staffed<br>3. NGOs contracted and funded as per NACO guidelines<br>5. NGO outreach staff (project coordinator, outreach workers, comm unity guides) recruited to cover intervention area as per staffing guidelines<br>6. Site validation process completed<br>7. Peer educators from HRG recruited to cover all sites as per peer selection guidelines<br>8. Outreach planning tools implemented by peers by site (especially to assess numbers of sex workers in a site, average number of clients in a month and days working in the month)
Input	1. FSW Annexure 5, *Tool for Peer-Led Outreach and Planning*, and NACO *Tool for Condom Programming*<br>2. Site-wise information on sex worker distribution, client volume and transaction frequency from outreach planning tools
Output	1. Condom demand estimates by NGO and site<br>2. Free condom distribution plan<br>3. Social marketing plan (if necessary)

Activities	Primary responsibility		
	SACS	TSU	NGO
Collate at NGO level condom availability information from site validation exercise			
Provide NGOs with survey results on number/proportion of condoms directly bought by clients (required for condom estimation formula)			
Calculate "site-wise" condom requirement figures using the estimation formula provided in the TI Guidelines document (Condom programming section) for each site under coverage by NGO			
Train outreach workers, peer educators and clinic staff on accurate methods for condom demonstrations			
Submit indents to SACS for estimated annual condom demand (after factoring in requirements for condom demonstrations, promotion events and all free distribution (peer, clinic and DIC)			
Consolidate NGO-wise demand estimates to arrive at State-level free condom requirement			
Submit indents to NACO for free condom supplies			
Establish logistics networks to deliver condom supplies to each NGO site			

**Free distribution of condoms**			
Set condom distribution targets for individual peers based on demand calculation for their respective networks			
Identify indirect outlets for stocking free condoms in and around hotspots based on consultations with community to establish outlet timings are suitable (especially for hotspots that operate late at night) and the outlets are accessible and community friendly			
Provide adequate condom stocks to peers for weekly distribution and reconcile stock balance with condom distribution records from peer cards/outreach registers at least every week			
Ensure adequate condom supplies and stock tracking mechanisms at all STI clinics and DICs (records should show number of condoms distributed to each HRG member who avails the service)			
Monitor condom stock levels at indirect outlets twice every week and replenish as required			
Prepare monthly condom utilisation report and submit to SACS/TSU showing distribution through each channel and consumption for other activities (demos, promotions, breakage etc)			
**Social marketing of condoms**<br>*(CBO-led social marketing not recommended unless warranted because of strong demand from HRGs; suggested role of NGOs/CBOs to provide information and feedback to existing SMOs to help improve their distribution)*			
Establish linkages with social marketing organisations (SMOs) operating in the same geographical areas (with TSU support as required) to ensure distribution of socially marketed condoms around hotspots			
Share updated list of hotspots, key outlets (especially outlets that operate late at night) and incidents of stock outs with SMOs to improve distribution at hotspots			
Create awareness among clients and regular partners about the availability of socially marketed condoms at hotspots			
**Monitoring**			
Commission research to assess condom availability around hotspots at all times (special focus on availability at night)<br>**(Target availability is at least 80% at all times)**			
Assess adequacy of direct and indirect distribution to cover all acts estimated (based on outreach planning calculations) within each peer's network and modify distribution channels and quantity to fill gaps			
Perform monthly condom accessibility audits at hotspots through peers (using outreach planning tool)<br>**(Modify distribution plan to address any issues identified)**			
Periodic tracking of condom usage by HRG through peer educators or through clinic counsellors (validate reported usage based on actual condom distribution numbers)<br>**(Other assessment through NACO BSS in Year 1, Year 3 and Year 5)**			

Major Milestones/ Targets	H1	H2
Year 1	**1.** Condom demand estimation exercise completed for 100% of sites<br>**2.** 100% NGOs complete annual demand estimation and submit to SACS<br>**3.** State-level consolidated demand calculated and indent raised to NACO<br>**(Steps 1-3 repeated at the start of every subsequent year based on updated information)**	**1.** Free condom distribution meeting at least 60% of estimated demand across all NGOs<br>**2.** Indirect condom outlets established at 60% of hotspots where need identified<br>**3.** No stockouts of more than five days at any NGO<br>**4.** Baseline condom availability study conducted across State
Year 2	**1.** Free condom distribution meeting at least 80% of estimated demand across all NGOs<br>**2.** Indirect condom outlets established at 80% of hotspots where need identified<br>**3.** No stockouts reported at any NGO<br>**4.** Linkages established with SMOs to improve distribution of SM condoms at 80% of hotspots	**1.** 80% of hotspots report condom availability in excess of 80% (as assessed through condom availability research)<br>**2.** Reported condom usage by HRG at least 60% in sex with commercial partners and 30% in sex with regular partners (as assessed through peer surveys)
Year 3	**1.** Indirect condom outlets established at 100% of hotspots	**1.** Reported condom usage by HRG at least 70% in sex with commercial partners and 40% in sex with regular partners (as assessed through peer surveys)
Year 4	**Maintain previous levels**	**Maintain previous levels**
Year 5	**Maintain previous levels**	**1.** Reported condom usage by HRG at least 80% in sex with commercial partners and at least 50% in sex with regular partners (as assessed through peer surveys)

Programme Component	7. Behaviour Change Communication / Inter Personal Communications
Prerequisites	1. TI coverage area and denominator fixed<br>2. TSU contracted and fully staffed<br>3. NGO contracted and funded as per NACO guidelines<br>4. NGO outreach staff (esp. project coordinator, outreach workers, advocacy officer) recruited to cover intervention area as per staffing guidelines<br>5. Site validation process completed<br>6. Peer educators from HRG recruited to cover all sites as per peer selection guidelines
Input	1. FSW Annexure 6a, *Tool for Dialogue Based Interpersonal Communication (IPC) By and With HRGs*
Output	1. IPC packages for risk reduction

Activities	Primary responsibility		
	SACS	TSU	NGO
Adapt IPC and BCC toolkits for local use			
Train NGO staff and peer educators on IPC methods - especially the value of analytical thinking and problem solving among community members to arrive at local solutions to HIV/AIDS risk and vulnerability issues			
Train NGO staff and peer educators on strategic planning for BCC message development			
Review NGO-developed BCC materials and NACO/SACS materials for message consistency / message reinforcement			
Conduct IPC capacity standards jointly with NGO staff and peer educators every six months to assess quality of IPC and identify areas for improvement			

Major Milestones/ Targets	H1	H2
Year 1		**1.** 100% of NGO staff and peers trained on IPC methods and strategic planning for BCC message development
Year 2	**1.** IPC capacity standards conducted at 100% of NGOs	**1.** 100% NGOs implementing IPC methods
Year 3	**1.** At least 80% NGOs show improvement on IPC capacity standards in second round of assessment	
Year 4	**1.** 100% NGOs show improvement on IPC capacity standards in third round of assessment	
Year 5	**Maintain previous levels**	

Programme Component	8. Enabling Environment
Prerequisites	1. TI coverage area and denominator fixed<br>2. TSU contracted and fully staffed (including advocacy officer)<br>3. NGO contracted and funded as per NACO guidelines<br>4. NGO outreach staff (esp. projec t coordinator, outreach workers, advocacy officer) recruited to cover intervention area as per staffing guidelines<br>5. Site validation process completed<br>6. Peer educators from HRG recruited to cover all sites as per peer selection guidelines<br>7. Service roll-out (STI, BCC, condoms, enabling environment) commenced
Input	1. Annexure 9, *Power Analysis*<br>2. Annexure 7, *Crisis Response System*
Output	1. Site-level advocacy plans<br>2. Site-level power structure analysis<br>3. Well defined crisis response systems

Activities	Primary Responsibility		
	SACS	TSU	NGO
Adapt power analysis and crisis response tools for local use			
Train NGO staff and peer educators on the use of power analysis and crisis response tools			
Conduct peer-led power structure analysis at each site to determine local power structures/stakeholders and their influence on the HRGs environment			
Establish crisis response systems at each site to track and address community crisis incidents within minimum elapsed time<br>1) Prioritise sites that have a high concentration of stakeholders with "disenabling" or "negative" influence<br>2) Train community members to perform critical roles<br>3) Identify and build linkages legal support teams at each site to assist community during cr ises<br>4) Setup 24-hour helpline support systems			
Advocacy with State police leadership (DGP and/or ADGP(training)) to support TI activities in all Districts including identif ication of nodal officers at State and District levels			
Conduct District-level meetings<br>1) With SP and Deputy SP level officials to raise awareness and support for HRG interventions and HIV issues with specific su pport requests (examples listed in Section 3.3.5.D)<br>2) TOT workshops to trai n District nodal officers for subsequent police station -level activities			
Conduct police sensitisations at District and town levels through multi -disciplinary teams (consisting of trained District police officers, lawyers, NGO staff, peers) **(Should b e handled with TSU support so activity will not seem like an NGO -only local initiative)**			
Conduct legal literacy sessions for peers and community members to inform them of legal provisions and their rights			
Set up advocacy and crisis response co mmittees at each site consisting of community members who are identified and trained for this specific role (also mentioned under community mobilisation section)			

Major Milestones/ Targets	H1	H2
Year 1	**1.** 100% of NGO staff trained on power analysis and crisis response tools	**1.** 60% of NGO sites complete power structure analysis and develop local advocacy plans<br>**2.** State-level advocacy workshop completed with DGP / AD GP level officer and support obtained for District activities<br>**3.** 100% of peers covered by legal literacy training sessions
Year 2	**1.** 100% of NGO sites complete power structure analysis and develop local advocacy plans<br>**2.** 60% sites set up crisis management systems to respond to incidents affecting HRG community within 24 hours<br>**3.** District-level police workshop and TOT covering SP and Deputy SP level officers completed in 60% Districts	**1.** 100% sites have set up crisis management systems to respond to inciden ts affecting HRG community within 24 hours<br>**2.** District-level police workshop and TOT covering SP and DySP level officers completed in 100% Districts<br>3. 100% Districts have police signed / police issued ID cards for<br>**3.** 40% of towns covered by police sensitisation workshops during the year<br>**4.** Advocacy committees with full community representation setup at 100% of NGOs
Year 3	**1.** At least 80% incidents being responded to within 24 hours in all sites	**1.** 100% of towns covered by police sensitisation workshops during the year (ongoing activity to be conducted every year)
Year 4	**Maintain previous levels**	**Maintain previous levels**
Year 5	**Maintain previous levels**	**Maintain previous levels**

Programme Component	**9. Community Mobilisation**<br>*(excludes CBO formation and transition from NGO to CBO, which are addressed elsewhere)*
**Prerequisites**	1. TI coverage area and denominator fixed<br>2. NGO contracted and funded as per NACO guidelines<br>3. NGO outreach staff (project coordinator, field officers, outreach workers, community guides) recruited to cover intervention area as per staffing guidelines<br>4. Site validation process completed<br>5. Peer educators from HRG recruited to cover all sites as per peer selection guidelines<br>6. At least 70% of HRG denominator contacted at least once, ideally within six to nine months of intervention start<br>**(This is critical to ensure that representative community members are involved in the project and not just the ones who are contacted first)**
**Input**	Community committees examples from TAI-VHS and Annexure 8, *Community Committees* Chapter 5
**Output**	1) Functioning community committees for project implementation and oversight<br>2) Functioning community networks across all sites

**Activities**	**Primary responsibility**		
	**SACS**	**TSU**	**NGO**
Adapt NACO specified guidelines and tools for formation of community committees and CBOs for local use			
**Raising community engagement and involvement in project service delivery (typically from month 3 of project)**			
Ensure peer selection guidelines are adhered to while recruiting peers for outreach			
Define clear role separation between peers and outreach workers to ensure that from initiation peers handle over 80% of 1-1 outreach contacts and condom distribution (role of outreach workers is to manage/monitor peers and provide technical support)			
Identify list of community leaders from each sites (jointly with peers and community guides)			
Involve community members (through group discussions facilitated by community leaders) in<br>1) selection of DIC and clinics (location, building, facilities)<br>2) selection of clinic staff - especially doctors and counsellors<br>**(Critical that peer involvement not be treated as a proxy for wider community involvement because peers, who draw remuneration, are usually seen as affiliated to the project by other community members)**			
Define ToRs for key committees with community representation (areas include project management, clinic services, DIC management, advocacy and crisis response, event management)			
Constitute community committees with membership from HRG community (not including peers) with following key guidelines - all typologies of HRG should be represented, and community members should be rotated every six months to ensure wide participation from community. Indicative numbers of committees as follows:<br>1) Project management committee - 1 per TI<br>2) Clinic committee - 1 per static and outreach clinic, 1 per site for referral clinics (i.e. to cover all referral clinics in that site)<br>3) DIC committee - 1 per DIC<br>4) Advocacy and crisis management committee - At least 1 per site (all hotspots with >100 HRG should have representation on the site committee)<br>5) Event management committee - 1 per TI			

Networking within the community (typically from month 6 the project)			
Organise quarterly "info-tainment" events to gather all the contacts in a peers network (approximately 60 contacts / peer as per NACO guideline)			
Identify and train community members from each hotspot as designated contacts for "crisis management" - to respond to violence or harassment			
Develop directory of welfare and livelihood schemes available from government for which HRG community members meet eligibility criteri a due to economic or social status			
Train all peers on SHG/community groups (CGs) formation methods by end of month 18			
Foster development of SHGs or community groups (in groups of 10-15 community members) across all sites to address economic and vulnerability issues			
Develop directory of literacy, welfare and livelihood schemes available from government for which HRG community members meet eligibility criteria due to economic or social status			
Build capacity of peers and community leaders to manage linkages for eligible SHG/CG members to access relevant schemes<br>**(NGO should not manage linkages directly - except in the first three months - and should instead build capacity of community members to perform this role) (Priority for developing linkages should be decided in consultation with community members, not by NGO alone)**			
**Increasing community ownership of the programme (typically from month 12 of project)**			
Finalise and publish transparent performance assessment criteria for NGO staff and peers			
Conduct annual NGO staff performance assessment (including peers) with community input			
Annual progression of selected peer to roles with enhanced responsibilities, based on performance assessment and peer progression criteria (NGO-led but with community input)<br>At the minimum by the end of Year 1:<br>1) all DICs to have a DIC manager from the community<br>2) all static and outreach clinics to have a clinic staff member (counsellor, ANM or administator) from the community			
**Improving governance / initiating CBOs (typically from month 18 of project)**			
Initiate community consultations to institute democratic processes for:<br>1) electing and rotating members of community committees (to be led by community leaders)<br>2) peer progression (to be led by peers)			
Organise six monthly District-level and annual State-level meetings of peers, community leaders and SHG members to facilitate networking			
**Monitoring of community mobilisation**			
Conduct group discussions with community members during monthly TSU field visits to assess:<br>1) community understanding of project roles and objectives<br>2) acceptance of project by community<br>3) attitudes of NGO staff towards community<br>4) relationship of peers with community members, especially to assess if peers are members of HRG<br>5) If community priorities are being addressed by project			

Major Milestones/ Targets	H1	H2
Year 1	**1.** 100% of NGOs recruited peer educators from respective HRG groups (in the overall ratio of 1 peer for 60 HRG members) as per NACO peer selection guidelines<br>**2.** 100% static clinics selected and finalised based on group consultations with community members (similar guideline for all DICs set up by the project)	**1.** 100% NGOs finalise and publish peer performance assessment and peer progression guidelines<br>**2.** At least 80% of outreach contacts and condom distribution occurring through peer educators<br>**3.** At least 50% of NGOs have constituted at least two community committees with clear ToRs and monthly meeting records<br>**4.** At least 70% of DICs managed by community member<br>**5.** At least 50% of static and outreach clinics have at least one staff member from the community
Year 2	**1.** At least 80% NGOs have constituted at least three community committees with clear ToRs and monthly meeting records<br>**2.** 100% of DICs managed by community members<br>**3.** At least 80% of static and outreach clinics have at least one staff member from the community<br>**4.** 100% peers trained on SHG formation processes<br>**5.** 100% of NGOs complete performance assessments per guidelines for all their peers (to be repeated annually in subsequent years)<br>**6.** At least 50% of peer educators organise quarterly events that bring together their outreach network	**1.** 100% NGOs have constituted at least four community committees (including advocacy and clinic committees) with clear ToRs and monthly meeting records<br>**2.** 100% of static and outreach clinics have at least one staff member from the community<br>**3.** At least 50% NGOs elevate at least one peer to a role of higher responsibility based on peer progression guidelines<br>**4.** At least 10% of community members are members of SHGs<br>5. At least 70% of NGOs have established linkages with literacy, welfare and livelihood schemes in their sites<br>6. 100% Districts organise community networking events that bring together community leaders and peers for all TI NGOs in the District (subsequently held every six months)
Year 3	**1.** At least 25% of community are members of SHGs/CGs<br>**2.** State-level community networking event for members from all TI NGOs (subsequently held every year)<br>**3.** 100% of NGOs have established linkages with literacy, welfare and livelihood schemes in their sites	**1.** 100% of community committees electing and rotating members through democractic processes<br>**2.** 100% of NGO elevating suitable peers to higher levels based on transparent performance assessments and peer consultations
Year 4	**1.** At least 50% of community members are members of SHGs<br>**2.** At least 80% sites have a functioning SHG with bank account and monthly meeting records	**1.** At least 20% of NGO staff drawn from community members
Year 5	**1.** At least 80% of community members are members of S HGs	

Programme Component	**10. Programme Monitoring**<br>*(STI monitoring covered in the STI services section)*
**Prerequisites**	1. TI coverage area and denominator fixed<br>2. TSU contracted and fully staffed (especially programme team and M&E officers) so that each **TSU project officer covers a maximum of 6 NGOs or 3 Districts** - whichever is smaller<br>3. SACS and TSU M&E and programme staff trained by NACO on TI monitoring and evaluation framework, including TI indicators and CMIS formats<br>4. NGO contracted, funded and equipped (including with a dedicated computer) as per NACO guidelines<br>5. NGO outreach staff (project coordinator, field officers, outreach workers, community guides) recruited to cover intervention area as per staffing guidelines<br>6. Site validation process completed<br>7. Peer educators from HRG recruited to cover all sites as per peer selection guidelines<br>8. Service roll out (STI, BCC, Condoms, enabling environment) commenced
**Input**	1. NACO TI Indicators<br>2. CMIS TI formats<br>3. Outreach planning formats for peer data capture
**Output**	1. TI data input into CMIS<br>2. Monthly MIS analysis reports for State, District, NGO, site<br>3. Monthly peer-level data analysis and workplans

**Activities**	**Primary responsibility**		
	**SACS**	**TSU**	**NGO**
Adapt NACO TI indicators and paper-based formats for local use			
Finalise graphical data capture and analysis tools for use of non-literate peer educators			
Train all NGO staff (project coordinators, outreach workers, accountant/data entry operators) on TI monitoring indicators and formats			
Collate site-wise data every month and submit updated paper-based TI indicators reports to SACS and TSU by 15th of following month *(e.g. data for Mar 2007 should be reported to SACS by 15th Apr 2007)*			
Enter monthly NGO data into NACO CMIS by 22nd of next month and generate monthly feedback reports for sharing with NGOs by 25th of following month *(e.g. NGO paper-based formats for Mar 2007 should be entered into CMIS by 22nd Apr 2007 and feedback reports sent to NGOs by 25th Apr2007)*			

Monitoring by outreach workers	SACS	TSU	NGO
Monthly review meeting with peers to:<br>1) Collate and review monthly outreach progress vs. goals for the month<br>2) Support peers to update microplan formats - especially social and geographic networks to reflect population changes (new individuals, individuals leaving project area)<br>3) Support peers to increase service focus (BCC, condom and clinic services) on the most at risk individuals in their network (those with highest client loads, low condom use or high incidence of violence)<br>4) Set outreach, clinic and condom distribution goals for the month based on above considerations<br>5) Plan thematic BCC campaigns and community mobilisation initiatives and events planned for the month<br>6) Finalise monthly advocacy plan to address key stakeholders (madams, pimps, policemen, regular partners)<br>7) Review clinic service uptake by peers **(very critical area because peers must serve as models for behaviour change through personal example)**<br>8) Develop personal workplan for the next month to support peers whose performance is sub par			
Weekly review meetings with peers (those linked to the outreach worker's monitoring cycle) to:<br>1) Collate and report to NGO weekly peer contact data using paper-based formats<br>2) Review outreach progress within peer's network (how many ever met, how many never met and reasons for the same, how many contacts planned during the week, how many achieved, problems faced)<br>3) Coordinate outreach with clinic service uptake (referrals made by peers, how many referrals actually converted to visits, plans to address failed referrals, tracking individuals with follow up visits to clinic, repeat STI cases)<br>4) Review accuracy and completeness of data records maintained by peers<br>5) Assist peers to develop peer workplans (daily and weekly) based on progress in the field (to focus services on most at risk and least served populations)<br>6) Document incidents of violence/harassment reported by community members and track follow up action taken, if any			
Conduct outreach work and review and analyse peer data cards/registers at least twice a week with every peer to ensure:<br>1) Knowledge of basic BCC and skills (HIV transmission methods, myths, condom demos)<br>2) Outreach planning is implemented per quality standards<br>3) All peers conduct outreach based on a daily work plan (peer should have planned in advance the individuals from her network that must be met on a particular day and the purpose of each meeting)<br>4) Peers transcribe outreach information (individuals met, issues discussed, services provided, number of condoms distributed, enabling environment issues detected) into peer cards at least every two days			
Random field visits (unaccompanied by peer educators) every week to assess if peers conducting outreach in the field as per work plan			
**Monitoring by project coordinators**			
Bi-weekly visits to each outreach worker's area for field observations and technical support on:<br>1) Clarity of project objectives<br>2) Clarity and accuracy of BCC messages and condom demonstrations<br>3) Accuracy and completeness of peer and outreach worker data formats/registers<br>4) Proper use of microplanning tools to raise service reach<br>5) Availability of communication materials, condoms and clinic commodities			
Review and analyse weekly peer contact formats submitted by outreach workers to assess:<br>1) Trends in outreach contacts and condom distribution across sites<br>2) Identify sites with performance issues and plan diagnostic support field visits within two weeks			

Monthly review meetings with outreach staff and clinic staff to:<br>1) Review monthly outreach and clinic performance vs. goals<br>2) Coordinate communication and planning between outreach and clinic teams<br>3) Identify and address enabling environment issues (violence, stakeholder problems, peer conflicts) affecting service delivery or uptake<br>4) Set goals for next month by site and by outreach worker area			
Review and collate NGO-level information on NACO TI indicators for reporting to SACS / TSU and data ent ry into CMIS			
Oversee monthly data entry into CMIS (data entry could be accomplished through accountant or part time staff)			
**Monitoring by TSU TI programme staff**			
Monthly visits to each NGO of at least three days by TSU team of at least 1 Project Officer and 1 technical officer (STI/M&E/Advocacy/BCC/Condom/Community mobilisation/Finance) to conduct field based support to outreach workers and peers to<br>1) Develop the diagnostic and problem solving skills of outreach staff when confonted by field level issues<br>2) Strengthen skills for data analysis and use for planning / diagnosing problems<br>3) Assess quality of microplanning<br>4) Provide on-site technical mentoring (including BCC methods and condom demonstration methods)<br><br>During such visits the TSU team should aim to meet all outreach workers and at least 50% of peer educators			
Monthly analysis of CMIS data reported by all NGOs to assess<br>1) Timeliness of submission and completeness and consistency of reported data<br>2) Perform trend analysis for each NGO on all NACO TI indicators vs targets (where applicable) and share feedback reports with each NGO<br>3) State-wise performance by consolidating data from all NGOs			
Bi-monthly random field interviews with individuals who have accessed program me services (based on outreach or STI records) to assess<br>1) whether project is actually serving HRG members<br>2) track user experience of project services<br>3) record attitudinal issues reported about any project staff (NGO, clinic, peers)			
Quarterly review of each NGO by TSU Project Officer and TSU technical team to assess project performance vs goals and quality of implementation on all major elements - outreach, BCC, condom, STI, enabling environment, community mobilisation, M&E, finance - with all major findings and key action points for next quarter reported in a formal project feedback note<br><br>**Note: A customised version of milestones contained in each section of the programme management guidelines could be used to set NGO goals**			
**Monitoring by SACS/JAT**			
Six-monthly visit by SACS NGO advisor to each NGO to assess financial systems and overall project implementation quality (including field visits to at least two implementation sites and meeting with 50% outreach workers and 25% peers)			
Annual NGO performance assessment by SACS Project Director and TSU project and technical team (could be conducted jointly for all NGOs or individually by NGO based on SACS preference)			

Major Milestones/ Targets	H1	H2
**Year 1**	**1.** 100% of NGO staff trained on TI monitoring framework including indicators and paper-based formats<br>**2.** 100% NGOs visited by TSU M&E staff every month for field level follow up on monitoring systems<br>**3.** 100% of NGOs visited by TSU Project Officer for at least three days every month<br>4. Clear annual milestones set on a comparable set of parameters for 100% of NGOs (process repeated every year)	**1.** At least 50% NGOs reporting monthly MIS data by 15th of following month and 100% reporting by 25th<br>**2.** Monthly CMIS data entry for 100% NGOs completed by 30th of next month (e.g. Mar 07 data entered by 30 Apr 07)<br>**3.** 100% NGOs being covered by quarterly review meetings with TSU project and technical team to assess progress vs. milestones<br>**4.** 100% of NGOs visited by SACS NGO advisor during the six month period<br>**5.** 100% of peers met by TSU staff during monitoring visits able to do accurate and correct condom demonstrations
**Year 2**	**Maintain previous levels. In addition:**<br>**1.** At least 80% NGOs reporting monthly MIS data by 15th of following month and 100% reporting by 22nd<br>**2.** Monthly CMIS data entry for 100% NGOs completed by 25th of next month (e.g. Mar 07 data entered by 25 Apr 07)<br>**3.** At least 40% NGOs meet Year 1 milestones as set during H1 of Year1	**1.** 100% NGOs reporting monthly MIS data by 15th of following month<br>**2.** Monthly CMIS data entry for 100% NGOs completed by 22nd of next month and feedback reports sent by 25th of next month
**Year 3**	**Maintain previous levels. In addition:**<br>**1.** At least 70% NGOs meet Year 2 milestones set during H1 of Year2	**Maintain previous levels**
**Year 4**	**Maintain previous levels. In addition:**<br>**1.** At least 80% NGOs meet Year 3 milestones set during H1 of Year 3	**Maintain previous levels**
**Year 5**	**Maintain previous levels. In addition:**<br>**1.** 100% NGOs meet Year 4 milestones set during H1 of Year 3	**Maintain previous levels**

ANNEXURE 11

MSM: Orientation, Identity and Vulnerability to HIV

1. INTRODUCTION

1.1 Why do Men have Sex with Men?

Men have sex with other men for many different reasons. In every society a minority of men are sexually attracted to other men. Many have wives or girlfriends and children, but they prefer sex with men. Some are single and only occasionally have sex with women. Some never have sex with women. Some men have sex with other men for money or gifts. They may prefer men or they may prefer women, but need or want the material reward that other men give them for sex.

Some men have sex with men because no women are available – because girls have to protect their virginity until marriage and women's sexuality is socially policed. Teenage boys in boarding school or adult men in single-sex institutions like prisons or the armed forces may seek other men for sexual release. Most sexual acts between men are consensual. However, some men are raped or otherwise forced into sex by other men for sexual release, especially if there is a power difference. This is common in prisons, but can occur anywhere. Some men use psychological rather than physical coercion to oblige other men to have sex with them.

When two men have sex, they need not always being doing so for the same reason. In a commercial exchange, for example, the client probably prefers men, while the man he is paying may prefer women but feel forced by economic need to perform sexual favours. Other men may sell their body to gratify a sexual desire to be penetrated.

1.2 Sexual Orientation and Sexual Identity

The current understanding of human sexual orientation is that homosexuality (being attracted to the same sex) and heterosexuality (being attracted to the opposite sex) are not bi-polar, watertight compartments, and that human sexuality flows along a continuum from exclusive homosexuality to exclusive heterosexuality. An individual's sexuality may be located at any point on this continuum, and it may change over the life of the individual. There is no clear scientific evidence for why some people are heterosexual and some homosexual. However, some evidence implies that both nature (biology) and nurture (upbringing) play a contributing role towards sexual orientation.

In most societies, heterosexuality is considered "natural" and "normative" and homosexuality is often considered "unnatural" or "abnormal". However, homosexuality is a fact of life, a social reality that has existed and continues to exist in different cultural and historical periods.

Unlike heterosexuals who do not have a distinct sexual identity, MSM in India are diverse in their sexual identities. Some MSM identify with the modern "gay" or "bisexual" identity, while others identify with indigenous sexual identities like *koti*, *dhurani*, *panti*, *giriya*, double-decker, etc. *Hijras* also have sex with men. In some metropolitan cities like Kolkata and Mumbai, some men who are involved in commercial sexual transactions with male and/or female clients have started

asserting a distinct identity of Male Sex Workers (MSW). Along with these, there are other men who have sex with men in different contexts and social environments, including truck drivers, migrant workers, *malishwala*, gym boys, film extras, etc.

Why do men have sex with men?

- Desire for other men - gender and/or sexual orientation
- Desire for specific sexual acts (anal/oral)
- Pleasure and enjoyment from discharge ("body heat")
- "Play" and curiosity
- Wife will not perform anal/oral sex or husband is ashamed to ask
- Men are easier to access (shared beds and spaces) while women are more socially policed
- Protecting a girl's virginity, maintaining chastity
- For money, employment, favours, rewards
- No one is suspicious when men mix with other men
- Anus is tighter than vagina and gives more pleasure
- No commitment to marriage
- Its is not considered "real" sex

Source: Based on *Induction White Book,* Naz Foundation International, Lucknow, 2006

Most research conducted on MSM agrees that these identities are fluid rather than fixed, and that depending upon the social environment, a person may adopt a different identity or no identity. However, what the diverse population of MSM has in common is the fact that all of them have sex with other men. As professionals working in the field of HIV/AIDS and sexual and reproductive health (SRH), our concern should be focused on their risks and vulnerability to contracting HIV infection and transmitting the virus to their male and female partners. Since HIV prevention programmes should locate MSM with high-risk behaviour in their social environments, a better way of understanding these identities is to look at the various frameworks within which MSM identify themselves.

1.3 What is the Correlation between Sexual Identities and Sexual Behaviours?

"Sexual identity" refers to the ways in which an individual, living in a particular cultural and historical context, experiences and lives out his or her particular combination of sexual orientation[1], sexual behaviour and gender identity[2]. The sexual identity of an individual has both public and private components. Public sexual identity refers to the ways in which a person presents him/herself in their social environment, while private sexual identity refers to the ways in which the person self-identifies with respect to their sexuality. Given the assumption that heterosexuality is the norm in Indian culture, publicly acknowledging one's homosexual identity is an inherently political statement that may have negative social consequences. Hence many MSM do not have a public homosexual identity.

[1] Sexual orientation embraces physical, interpersonal and intrapsychic factors. A person's sexual orientation can be assessed based on the sexual attraction, sexual behaviour, sexual fantasies and self-identification.

[2] Gender identity is a person's internal and deeply felt sense of being a man, woman, or neither male nor female. Gender identity is usually socially and culturally defined. In some cases, it does not match an individual's biological sex. For example, a person may be born biologically male but identify as a woman.

Evidence increasingly shows that there are no defined sexual roles among *kotis* and many are both active and passive depending on circumstances, partner preference, opportunity and desire. There are places where none of the identities discussed above exist, and an MSM takes both penetrative and receptive roles, as in the case of Manipur: "Contrary to popular belief, at times 'B' MSM also play the penetrative role in sex with their 'A' partners. But they do so only with their regular partners and do not reveal this to other 'B' MSM because of the fear of being ridiculed. It is also said that 'A' MSM do not have sex among them[selves], and the 'B' MSM also consider sex among them[selves] as a sin. But there is anecdotal evidence to suggest that these conventions are broken more often than [is] believed."[3]

Since MSM interventions do not exist in many States, the best approach would be to target the larger MSM population, without focusing on sexual identities. Through this approach the intervention would be able to cover all MSM who have unprotected receptive anal sex, have large number of partners and who are involved in commercial sexual acts. At the next stage, the intervention can then focus on specific identity based populations that would evolve through community mobilisation and organisation.

The fact that many MSM do not have a public sexual identity and are subsumed into the population of the country poses a challenge in targeting HIV prevention programmes to them. Unlike injecting drug users or brothel-based sex workers, the majority of MSM are an invisible population, even though more and more people are coming out in the public about their sexuality. The answer to this challenge lies in the fact that those MSM who have a public sexual identity are making efforts to network and form groups and collectives and to register them under appropriate legislation (CBOs). SACS and other donors should avail the information about these initiatives in their States.

1.4 Who is a *hijra*?

Until a few years, *hijras* were the only visible section of society with an alternate sexual (and social) identity. *Hijras* belong to a distinct socio-religious and cultural cult, now recognised as a "third gender" (separate from male and female) by the government of India. They dress in feminine attire (cross-dress) and are organised under seven main *gharanas* (clans). Among *hijras* there are emasculated (castrated) men, non-emasculated men and inter-sexed persons (hermaphrodites). While a sub-section of *hijras* are involved in blessing and gracing during births, marriages and ceremonies, another sub-section is involved in begging, and a third group is involved in sex work.

[3] SAATHII Calcutta: Manipur MSM initiative exploratory visit report, SAATHII Calcutta LGBT Support Centre, Kolkata, April, 2004

Some people equate MSM, specifically the effeminate *kotis/dhuranis* with *hijras*. While there are some similarities among *kotis* and *hijras*, they cannot be viewed through the same lens for HIV/AIDS work. *Hijras* and *kotis/dhuranis* share certain similarities, such as the language they use within their network, feminine attire and effeminate demeanour. The typical dialect that *hijras* and *kotis* share is *Ulti* (literally meaning "opposite") which is said to be the "corrupt" form of Sanskrit. Among *hijras*, the bonding between the community members is in the form of *guru-chela* (master-servant) relationship, which is akin to *koti* kinships such as elder-younger sister, aunt-nephew or mother-daughter. Because the *hijra* community has long been a visible, socially acknowledged alternate sexuality, some effeminate *kotis* used to join this community. With the increasing tolerance towards gender differentiation and sexual behaviour, effeminate *kotis* have started resisting this and desired to remain as biological men with a difference. In Southern India, *hijras* are known as *Ali*[4] *or Avaranis*.

1.5 Are MSM a Homogenous Population?

MSM (men who have sex with other men) are a diverse population in terms of personal sexual identities, age, languages, religion, marital status, sexual behaviours and other socio-economic characteristics. Table 1 describes the diversity of this population using data from the National Behaviour Surveillance Survey (BSS)[5] in Maharashtra[6], Tamil Nadu[7] and Pondicherry[8] APAC: HIV Risk Behaviour Surveillance Survey in Pondicherry, AIDS Prevention and Control Project, Chennai, 2004.

The data show that the sampled population of MSM is young, and has a higher literacy level than female sex workers. A very high percentage of MSM in Tamil Nadu was employed and had an average personal monthly income of Rs. 2312. About one third of the MSM had ever been married (heterosexual). Among the married *aravanis* (*hijras*) in Tamil Nadu, 79% had male spouses, 3% had female spouses and 18% had *aravani* spouses. Studies conducted in Kolkata, Mumbai, Chennai, Lucknow, Delhi, etc. also confirm that MSM are diverse in terms of their socio-economic characteristics.

---

[4] Sherry, Joseph: *Social work practice and men who have sex with men*, Sage Publication, New Delhi, 2005, pp158-159.

[5] NACO: National baseline high risk and bridge population behavioural surveillance survey, Part 2, National AIDS Control Programme, New Delhi, 2002.

[6] AVERT: BSS in Maharashtra, Wave II, Avert Society, Mumbai, 2004.

[7] APAC: HIV Risk Behaviour Surveillance Survey in Tamil Nadu, Wave IX, AIDS Prevention and Control Project, Chennai, 2004.

[8] APAC: HIV Risk Behaviour Surveillance Survey in Pondicherry, AIDS Prevention and Control Project, Chennai, 2004.

Table 1: Selected Socio-Economic Characteristics of MSM

Study	Year	Sample	Sampling Location	Mean Age	Highest Educational Attainment	Marital Status	Average Personal Income (Rs/m)	Percent Employed
National BSS	2002	1387	Delhi Kolkatta Mumbai Chennai Bangalore	28	Secondary (25%)	34 % married	No Info	No Info
Maharashtra BSS	2004	1402	Mumbai Thane Sangli Satara Solapur Aurangabad Nagpur	27	Secondary (34%)	No Info	No Info	No Info
BSS, Tamil Nadu	2004	300 MSM	Chennai Madurai	28	Literate (94%)	27 % married	2312	89
		250 Aravani	Chennai Madurai Salem	30	Literate (96%)	15 % married.	1875	81
BSS, Pondicherry	2004	200	Pondicherry Karakal	24	Literate (93%)	12% married	1581	54

1.6 What do we Know about Male sex Workers (MSWs) and Sex Work by *hijras*?

Since male-to-male sex is not socially acceptable and is stigmatised, many MSM prefer to have sex with anonymous partners, often in public places like parks, bus/train stations, public toilets, movie halls, etc. Almost all studies conducted on MSM in different parts of the country have shown that a commercial transaction takes place between these partners. These commercial transactions may be pre-determined at the beginning of the sexual act, or determined after it. A transaction may also happen after the sexual act in the form of gifts or a taxi fare home, which may not be pre-determined. Further analysis of these partnerships has shown that some MSM consider commercial sex work as a vocation (MSW, *hijra* sex workers), while others consider it as non-vocation (hotel boys, domestic workers, masseurs, film extras, etc.).

In a study among 6,661 MSM in Andhra Pradesh,[9] about 9% reported that they were MSW. Another study from West Bengal[10] of 252 MSM showed a very higher percentage (62) was involved in a commercial transaction. In the Maharashtra BSS, about two-thirds (62%) reported having had sex with at least one male partner in exchange for money. While the data from Andhra Pradesh represent self-identified MSW (vocation), the Maharastra data represent the clients of MSW and the West Bengal data represent MSW and their clients. Another study from Surat in Gujarat shows a much lower percentage of MSM who received money (7%) and who paid money for sex (4%). Taken together, these studies show that the extent of commercial sexual transactions varies from place to place. Even though *hijras* do not publicly acknowledge that their community members are engaged in sex work, the Dai Welfare Society, a *hijra* CBO, claims that about 75% of its members are involved in sex work for their livelihood.

---

[9] Dandona, Lalit et.al: *Sex behaviour of men who have sex with men and risk of HIV in Andhra Pradesh, India*, AIDS, Vol. 19, 2005

[10] Praajak and Manas Bangla: *Report of an assessment of the sexual health needs of MSM in West Bengal*, New Alipore Praajak Development Society, Manas Bangla Network, Kolkata, 2006.

1.7 How does the Law Treat MSM and *hijras*?

Section 377 of the Indian Penal Code, drafted in 1833, is often cited as a hindrance to HIV prevention work among MSM. Under Section 377, whoever voluntarily has "carnal intercourse against the order of nature with man, woman or animal" shall be punished with the establishment of evidence of penetration. In addition, there are other laws that hinder HIV prevention work among MSM and *hijras*:

- Section 292 of the Dramatic Performance Act, 1876 and Customs Act, 1962 can be used to label the printing/importing safer-sex educational materials (books, or electronic document) as obscene, instead of life-saving educational materials.
- Anti-vagrancy laws and local Police Acts, powered by the Public Nuisance Act and Section 268, IPC, can also be applied against MSM in cruising areas on grounds that they are exhibiting "indecent behaviour" and disturbing the "public order".
- The Prevention of Immoral Trafficking Act, 1986, which is usually applicable to women, can also be made applicable to male and *hijra* sex workers.

1.8 Which MSM networks are available for HIV work?

Since the early 1990s, some geographically and ideologically dispersed MSM have organised into groups and collectives. There have been about 94 MSM CBO initiatives in 14 states across the country, of which the highest number was in West Bengal and Maharashtra, followed by Karnataka, New Delhi and Andhra Pradesh. One of the key points to be noted is that networking that initially started in metropolitan areas in the early 1990s is now being seen in smaller urban centres like Baruipur and Bhadrak.

Along with the formation of groups and networks, different publications by and for the MSM community have also appeared, e.g. *Bombay Dost, Pravartak/Naya Pravartak, Gay Scene, The Network, Pratyay, Swikriti Patrika, Aarambh, Darpan, Freedom, Sabang, Sanga Mitra, Sacred Love* and *Time Share*.

2. VULNERABILITY OF MSM AND *HIJRAS* TO HIV AND STIs

2.1 What is the Prevalence of HIV Infection among MSM and *hijras*?

Even though India is classified as a country with a concentrated epidemic (infection contained among certain vulnerable groups), unlike South Africa where the epidemic is generalised, the sentinel surveillance system does not adequately represent FSWs, MSM and IDUs to collect data and substantiate this argument. For instance, there were only 15 sites for MSM, 30 for IDUs and 87 for FSWs out of the total of 750 proposed sites for the 2005 sentinel surveillance round.[11] SACS should make efforts to increase the number of sites for core group surveillance under NACP III to effectively monitor the spread of the virus.

The 2003 sentinel surveillance study[12] of MSM showed an HIV prevalence rate of 29% in Manipur, 27% in Delhi, 13% in Andhra Pradesh, 11% in Karnataka, 9% in Goa, 2% in Bihar and 1% in A & N Island. Another cohort study in Mumbai showed a sero-prevalence rate of 21% among MSM. In Chennai, the HIV prevalence figure from a study of a slum population was estimated at 8% for MSM, of which more than half were married.[13] The 2004 sentinel surveillance report of MSACS on a combined sample of MSM and *hijras* from a consistent site in the Humsafar Trust showed an HIV prevalence of 23.9% in 2000 followed by 23.6% in 2001, 16.8% in 2002 and 18.4% in 2003. In 2004 when the population was divided into MSM and *hijras*, the HIV prevalence rates from the same site were 9.6% and 49.3% respectively. Results from community based studies among MSM in Gujarat show a prevalence of 17% in Ahmadabad followed by 15.6% in Surat and 6.8% in Vadodara. Even though prevalence studies are location specific and are of limited use for generalising at the national level, some of these figures show very disturbing trends that call for immediate focused intervention among MSM.

2.2 What is the Prevalence of STIs among MSM?

Data from studies on reported STI instances, and community-based studies that included laboratory testing for STI, give an indication of the prevalence of STIs among MSM. In the National BSS[14] genital discharge was reported by 16% of MSM, one-fourth reported genital ulcers/sores, and one-third reported genital discharge or ulcers/sores in the last 12 months. 41% reported that they had suffered from at least one of the above symptoms in the previous year, while 22% reported more than one symptom. MSM were more likely than non-MSM to self-report having STIs such as anal warts, gonorrhea and abnormal discharge.[15]

---

[11] NACO, 2004: Annual Sentinel Surveillance for HIV infection in India, Country Report 2003, New Delhi, PP-118

[12] Cf, Population Council, 2005: *Men who have sex with men in India: A desk review*, draft for review of NACP-III Planning Team, New Delhi.

[13] Go, Vivian et al: *High HIV prevalence and risk behaviour in men who have sex with men in Chennai, India*, Epidemiology and Social Sciences, Vol. 35 (3), pp 314-319.

[14] NACO, 2002: National Baseline High risk and bridge population behavioural surveillance survey, Part 2, National AIDS Control Programme, New Delhi,

[15] Go, Vivian et al: *HIV prevalence and risk behaviours in men who have sex with men in Chennai, India*, Epidemiology and Social Science, Vol 35 (3), 2004

Three community based studies in Gujarat looked into the prevalence of STIs and HIV through laboratory diagnosis. Samples (between 300 and 400) were recruited from CBO/NGO led TI projects supported by Ahmadabad MCACS and GSACS, and the study was conducted with technical support from RCSHA.

Table 2: Laboratory Diagnosis of STI among MSM in Gujarat (figures in %)

Place and Date	Tricho-moniasis	Gonorrhea OP	Gonorrhea R	Gonorrhea U	Chlamydial infection	Syphilis RPR	Syphilis TPHA	HIV	LW test
Vadodara, 2004	1.6	4.3	6.5	6.3	3.4	7.2	17.2	6.8	
Surat, 2005	3.7	4.7	5.4	1.7	2	12.4	31.1	15.6	3.3
Ahmadabad*, 2004	1.8	3.3	7.9	4.3	3.6	15.1	36.5	17	12.2

* Includes *hijras* also

(Gonorrhea OP: positive culture from oro-pharyngeal specimen; Gonorrhea R: positive culture from rectal specimen; Gonorrhea U: positive PCR test from urine specimen)

The above study shows that the most prevalent STI among MSM and *hijras* was syphilis, and the prevalence ranged between 3.5% in Ahmadabad and 17.2% in Vadodara. Though the prevalence of syphilis was high (those who are positive for TPHA tests), the lower positivity of syphilis RPR test (positive RPR test at 1:8 dilution and above) indicated a lower prevalence of active syphilis. Gonorrhea was found to be the next most common STI among MSM. While the ano-rectal region was the commonest site for gonococcal infection in all the three study sites, the urethra and the oro-pharynx region was equally infected among samples from Ahmadabad and Vadodara. In Surat, the second commonest site for gonorrhea infection was oro-pharynx, thus showing prevalence of oral STI among MSM. Almost all the gonococcal infections were asymptomatic, as none of the participants had complained of urethral or rectal discharge. Similarly, there was no urethral discharge in any of the participants whose urine specimens were positive for chlamydial infection by the PCR test. The findings from the laboratory diagnosis studies seriously challenge the efficacy of syndromic management of STI under NACP for MSM and *hijras*.

2.3 What is the Level of Awareness about HIV among MSM?

Men in general have access to increased information on transmission routes that has been disseminated across the general population in recent years, but these campaigns have not adequately addressed male-to-male transmission. The National BSS showed that nearly 69% of MSM were aware of two methods of prevention of HIV, while the awareness was almost universal in Tamil Nadu and Pondicherry. In addition, the Maharashtra BSS showed that 75% of MSM were aware of three methods of prevention of HIV. While misconceptions about the transmission routes of HIV were present among 45% of MSM at the national level, the figures were 38% in Tamil Nadu, 33% in Pondicherry and 36% in Maharashtra. Since there are focused interventions among MSM in these States, and since these studies were conducted 3 years after the National BSS, it could be concluded that there is an increase in accurate awareness of HIV transmission routes and a decrease in misconceptions among MSM during this period.

2.4 What are the Risky Sexual Behaviours and Condom Use among MSM and *hijras*?

It is not a person's identity but their behaviour that can put them at risk for HIV/STI transmission. Hence we should first understand what kind of sexual behaviours are risky for MSM. Penetrative sex among men in the form of penile-anal intercourse and penile-oral sex increases the risk of HIV/STI transmission. Sexual behaviour studies have shown that anal sex is one of the most popular sexual behaviours among MSM (77% in West Bengal, 89% in Mumbai and Tane[16]). HIV and STIs are more likely to be transmitted during anal sex than during vaginal sex, because the anus is not naturally lubricated and penetration will lead to small tears and lesions that allow easier HIV transmission. As with heterosexual sex, where the woman is at more risk than the man, the receptive partner is more at risk than the penetrating partner in anal intercourse. But the relative risk to the penetrator is greater in male-male intercourse compared with male-female intercourse, as the probability for tears and lesions in the penis of the penetrating man is higher.

The other form of penetrative sexual activity among MSM is peno-oral sex. Even though there is a myth that oral sex is "disgusting" and is not commonly practiced, behaviour studies of MSM from the intervention sites of Humsafar show that oral sex is another preferred sexual activity among 89% of respondents.[17] The increasing number of MSM who admit to having had peno-oral sex may be indicative of a shift from high risk to low risk sexual behaviour among MSM. The data also show that the prevalence of condom use during oral sex is extremely low, which increases vulnerability to oral STIs. MSM interventions have been more focused on increasing condom use in anal sex and may have neglected the promotion of condom use during oral sex.

Table 3: Penetrative Sex and Condom Use among MSM

Study Year	Sample Size	Anal Sex in Previous Month (%)		Last Time Condom Use in Anal Sex (%)		Oral Sex in Previous Month (%)		Last Time Condom Use in Oral Sex (%)	
		Received	Penetrated	Received	Penetrated	Received	Penetrated	Received	Penetrated
2000	174	53	67	50	58	73	74	30	17
2002	251	62	52	91	89	70	68	36	33
2004	240	53	60	83	72	65	71	20	13
2006	295	68	62	72	72	70	70	21	24

The other aspect that needs to be taken into consideration is the number of partners and the nature of the relationship. The National BSS shows that on an average, MSM had sex with 9 commercial male partners and 5 non-commercial male partners in the month preceding the survey. In the Maharashtra BSS about 62% reported having at least one commercial partner and about 5 anal sex partners. The Tamil Nadu BSS shows a sharp increase in sex with paid partners (59%) and a decrease in sex with regular partners (18%) in 2004 from the figures of 28% and 37% respectively in 2001. Sex with non-regular partners among *aravanis* in Tamil Nadu also rose from 76% in 2003 to 97% in 2004. With a larger number of partners and when the sexual relationship is commercial in nature, the vulnerability to HIV and STIs is greater.

[16] Humsafar, 2005

[17] Behavioural studies conducted by Humsafar Trust in 2000, 2002, 2004, 2006

A high proportion of respondents (83%) in the National BSS were aware that correct and consistent use of condoms could protect people from HIV. However, only 30% reported consistent condom use with non-commercial partners and 13% with commercial partners in the month preceding the survey. In Tamil Nadu, condom use among MSM in the last anal sex with paid partners was 82%, with a regular partner 30%, and with casual partners 79%. Among *aravanis* the figures during paid sex, non-regular sex and casual sex were 81%, 79% and 64% respectively. Condom use among MSM in Tamil Nadu increased in sex with paid partners and casual partners in 2004, but has decreased among *aravanis* in paid sexual encounters, compared to earlier BSS studies. Another study from Surat shows that consistent condom use with male commercial partners was also low as compared to non-commercial partners. Another disturbing finding of this study is that consistent condom usage was lowest (20%) with female partners. (See Figure 4.)

Consistent condom use by MSM with commercial partners, which is much lower compared to brothel-based female sex workers, clients of sex workers and non-brothel-based sex workers, is a major concern which interventions focusing on MSM should address.

Self-perception of risk is a motivator in adopting safer behaviour. Almost one third of MSM interviewed in the National BSS reported that they perceived themselves to be at high or moderate risk of getting HIV. Risk perception was low in Tamil Nadu, with 38% deeming themselves to be at risk in contracting HIV while it was 24% among those MSM who did not use a condom during their last anal sex with non-regular male partner.

Figure 4: Frequency of condom use, Surat, 2005

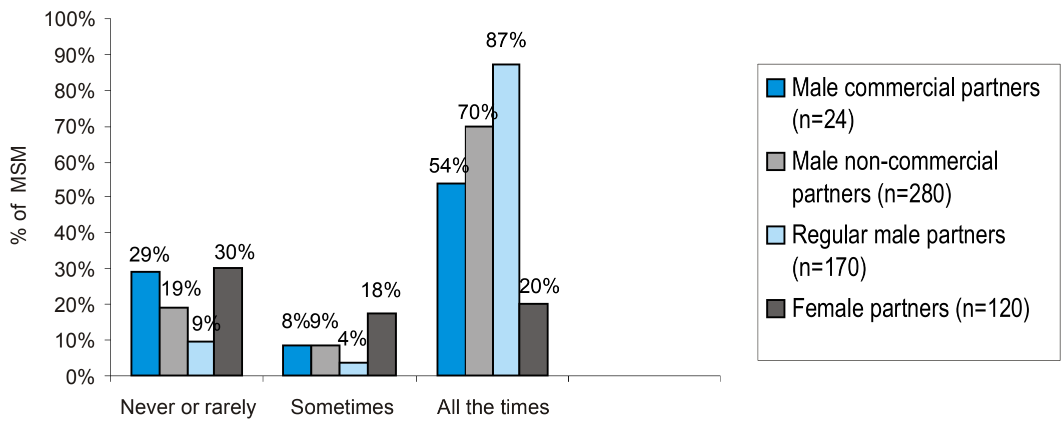

2.5 What is the Linkage between MSM and Injecting Drug Use?

Studies like the National BSS have shown that some MSM are involved in unsafe injecting practices. This increases the vulnerability of cross-transmission of HIV between MSM and the IDU population and the rate of spread of the virus. Intoxicating drug use was reported by nearly 13% of MSM, of which a significant proportion (12%) had also reported injecting addictive drugs without a medical prescription and 41% reported using previously used needles/syringes. Ethnographic data from Manipur show that the partners of MSM include either ex-drug users or practicing drug users. Interestingly, the sample for the sentinel surveillance data from Manipur was recruited from the well-known IDU intervention project of SASO. SASO is also facilitating the formation of the MSM CBO Maruploi Foundation in Manipur. In places where IDU is a practice, the inter-linkage between the two populations should be explored in detail.

2.6 What is the Health Seeking Behaviour of MSM?

The national BSS shows that MSM normally seek treatment for their STI episodes from private healthcare providers (44%), followed by public heath provider (28%), chemist shop (14%), NGO peer educators/ clinics (15%) and home based remedies (11%). Only 20% of MSM sought treatment for STIs from qualified allopathic practioners in Maharashtra, while the figure was as high as 86% in Tamil Nadu.

The main barriers in accessing health services from health care providers by MSM and hijras are as follows:[18]

- Fear of bias or prejudice from the health care providers
- Past negative experiences from health care providers after revealing same-sex behaviour
- Homophobia/biphobia/transphobia of the health care providers
- Refusal to treat or substandard care of persons who revealed their same-sex behaviour to health care providers
- Health care providers trying to “cure” same-sex attracted persons from their homosexuality
- Pathologising of same-sex/bisexual orientation by health care providers
- Low self-esteem among the MSM and *hijras* seeking treatment
- Heterosexual assumptions on medical forms and in providing medical information on sexual and reproductive health
- Gender assumptions on medical forms and not considering alternate gender identities like transgender/transsexuals
- Concerns about breach of confidentiality by the health care providers
- Fear of being “outed” (having one’s sexual orientation revealed against one’s will) to others
- MSM and *hijras* stigmatised as “risk groups” for spreading HIV infection to the general population
- Refusal to treat or lack of knowledge about how to treat transgender persons who request hormone therapy or sex change operations
- Exclusion from health promotion campaigns including STI/HIV public awareness programmes

[18] Charkrapani Venkatesan: Handbook for STI/HIV and sexual health care providers, 2005. www.indianGLBThealth.info

ANNEXURE 12

Excerpt from Infosem's 'Strategic Plan for Scaling Up Interventions for MSM and Transgender Populations in India'

1. Specific Details of Scale-up for some Regions and States

State or region	Who will begin interventions in districts with no coverage?	Who will expand interventions in districts with low coverage?	What consultation process do we suggest for beginning and expanding interventions?	What assistance can current CBOs, NGOs, and networks provide for capacity building
AP, Karnataka, Kerala			▪ Community consultation to be held with full representation to get consensus decisions.	▪ With funding, INFOSEM and NFI can provide capacity building workshops on sexuality/gender; set up process, financial management, accounting, inventory, M&E, audits, documentation.<br>▪ Other CBOs (to be identified) have the skills to provide technical and managerial assistance to new CBOs.
Gujarat	Most existing NGOS and CBOs.	Most existing NGOS and CBOs.	▪ Proactive collaboration with existing NGOs and CBOs<br>▪ Expand networks of existing CBOs	▪ With funding, NFI and INFOSEM
Goa	N/A	Humsafar Goa	Same as Maharashtra (below)	▪ With funding, NFI and INFOSEM
Maharashtra	INFOSEM Network members and Udaan. Priority districts with no current CBOs or interventions include Ahmednagar, Aurngabad, Chandrapur, Dhule, Jalna, Latur, Nanded, Raigad, Ratnagiri, and Solapur.	▪ Jalgaon – Udaan<br>▪ Kolhapur – Samapathik<br>▪ Nagpur – Sarathi<br>▪ Nashik – Samapathik and Udaan<br>▪ Pune – Samapathik, Udaan, and Astitva.	▪ Meetings with SACS and other donors<br>▪ Community representation in recruitment process<br>▪ Community participation in size estimation and sites assessment.	▪ With funding, NFI and INFOSEM
Kashmir	SACS, Humsafar Kashmir.	SACS, Humsafar Kashmir.	Work with organisations already involved in process, share data.	▪ With funding, NFI and INFOSEM
Uttar Pradesh	SACS, NFI, Bharosa Trust (Year 2-3)	SACS, NFI, Bharosa Trust (Year 4-5)	Consultation to include all groups working in UP on MSM and TG issues, to share experiences, and develop helping relationships	▪ With funding, NFI and INFOSEM
East and Northeast	Second priority; based on additional funding.	First priority.	▪ Need state and/or regional consultation to outline who, where, and what to scale up (to avoid overlap, territoriality)<br>▪ Donors need to conduct peer reviews of proposals for expansion/new interventions	▪ With funding, NFI and INFOSEM

2. District-wise Distribution of MSM and TG Targeted Interventions

Colour coding

State/UT	A = High Prevalence	B = Concentrated epidemic	C = Highly vulnerable	D = Vulnerable
District	A (163) = High Prevalence	B (59) = Concentrated epidemic	C (278) = Increased presence of vulnerable populations	D (111) = Low prevalence, low/ unknown vulnerability

Districts by Region: South, Northeast, East, North, West

State/District	Category (A-D)	Urban Centre	Population 2001	MSM-TG size Estimation	NACO Estimate of TI need/ MSM	Number of TIs/ Pop'n covered	Implementing Organisation	What Gaps?
**SOUTH**								
**Andaman & Nicobar Islands: Vul (2 Districts)**			**356,152**		**NONE NEEDED**	**0/0**		
1. Andaman	D					0		
2. Nicobar	C					0		
**Andhra Pradesh[1]: Hi Prev (23 Districts)**			**76,210,007**		**12 Exclusive + 25 Composite**	**3+?/?**		
3. Adilabad	C			486			Shree Sai Mahila Mandali, Swayam Shakthi Society, Alliance NGO	Mapping, Quality STI Care, Condom & Lubes Supply, Care & Support
4. Anantapur	A			4,604			Alliance NGO, Sahacharudu Society	" "
5. Chittoor	A			1,264			Pass(Tirupathi), Sneha Sangam, CRLRSWA (Nagari, Srikalahasthi, Nindra)	" "

State/District	Category (A-D)	Urban Centre	Population 2001	MSM-TG size Estimation	NACO Estimate of TI need/ MSM	Number of TIs/ Popn covered	Implementing Organisation	What Gaps?
7. East Godavari	A	Rajahmundry, Kakinada	4872.62	1,825		1825+	Godavari Mata Welfare Association (CBO-Rajamundry), HLFPPT NGO, Kranthi Rekha Welfare society (CBO-Amalapuram), Aasha Kiranam Welfare Society(CBO-Kakinada)	
8. Guntur	A	Guntur, Narsaraopeta, Tenali and Pidugurall a	4405.52	2,670		2670+	HLFPPT NGO, Sneha Sudha(CBO), Friends Society(CBO)	
9. Hyderabad	A	Hbad		85,000?			MITHRUDU - TI/State Network, ASHA Society - MSM PLHA Network, Raksha Society- MSM PLHA Network, Suraksha Society - State CBO, AMMA - State CBO Associations, Saathi	Mapping, Quality STI Care, Care & Support
10. Karimnagar	A			1,020			Alliance NGO, Shakthi Society	Mapping, Quality STI Care, Condom & Lubes Supply, Care & Support
11. Khammam	A			890			Jeevan Kranthi Welfare Society	
12. Krishna	A	Machilipatnam	4218.41	1,553		1,533+	HLFPPT NGO, SAATHI (CBO-Vijaywada)	
13. Kurnool	A			2,677			Alliance NGO, Suraksha Society - Ind. CBO	
14. Mahbubnagar	C			1,500			Jeevan Jyothi Welfare Society CBO	
15. Medak	A			480			Alliance NGO, MITHRUDU(CBO), Saheli Samanvaya Sangam(CBO-Sangareddy),	

16. Nalgonda	A			1,640			Alliance NGO, Chaitanya Welfare Society (CBO)	
17. Nellore	A			10,000?			Aikyatha Welfare Society(CBO), Duties(NGO), Spandana,	
18. Nizamabd	A			942			Alliance NGO, Anubandham Society	
19. Prakasam	A			10,000?			Navjeevan Welfare Society(CBO- Ongole), Aasha Prakasham, Apthudu, Apthbandav, Friends(TG), Chaitanya	Mapping, Quality STI Care, Condom & Lubes Supply, Care & Support, Technical Support for CBO Development
20. Rangareddy	A			1,253			MITHRUDU(CBO), ASHA Society- MSM PLHA Network, Raksha Society- MSM PLHA Network, Suraksha Society	Mapping, Quality STI Care, Consistent Supply of Condom & Lubes ,Care & Support
21. Srikakulam	A	Palasa, Sklm	2528.49	886		886+	HLFPPT NGO	Mapping, Quality STI Care, Condom & Lubes Supply, Care & Support, CLI
22. Visakhapatnam	A	Vizag, Anakapalle and Paderu	3789.82	1,457		1457+	HLFPPT NGO, Sahara Trust(CBO)	Mapping, Quality STI Care, Condom & Lubes Supply, Care & Support
23. Vizianagaram	A	Vizianagaram and Parvathipuram	2245.1	947		947+	HLFPPT NGO	
24. Warangal	A			3,043			Deeparadhana, Alliance NGO	
25. West Godavari	A	Eluru	3796.14	1,771		1,771	HLFPPT NGO	Mapping, Quality STI Care, Condom & Lubes Supply,Care & Support, CLI
**Karnataka[1]: Hi Prev (27 Districts)**		**246**	**52,850,562**	**11,408**	**12 Exclusive + 21 Composite**	**1+?/ ?**		
26. Bagalkot	A	-	1,652,232	-	1 Composite		KHPT	
27. Bangalore City	A	49	6,523,110	7049		1+?/6255+?	Sangama, Swabhava Trust/Gelaya	

[1] Size estimations for Karnataka taken from Avahan and represent high -risk MSM. There may be more under different definitions.

28. Bangalore Rural	A	9	1,877,416	182	1 Composite	1/1700?	Suraksha	
29. Belgaum	A	20	4,207,246	158	Composite		Birdu (?)	
30. Bellary	A	10	2,025,242	317	CBO+ Composite		Sadhana + Myrada	
31. Bidar	A	6	1,501,374	65				
32. Bijapur	A	6	1,808,863	96	CBO+ Composite		Spoorthi + KHPT	
33. Chamrajnagar	A	4	964,275	268	CBO+ Composite		Belaku + Myrada	
34. Chickmagalur	C	8	1,139,104	133				
35. Chitradurga	A	6	1,510,227	141	1 Composite		Myrada	
36. Davangere	A	6	1,789,693	272	CBO+ Composite		Chetana + Abhaya	
37. Dharwad	A	6	1,603,794	263	CBO+ Composite	2/1000?+?	Snehajyohi + Suraksha	
38. Gadag	A	9	971,955	96	1 Composite		Samraksha	
39. Gulbarga	A	12	3,124,858	165	1 Composite		KHPT	
40. Hassan	A	8	1,721,319	136	CBO+ Composite		Nazar + SVYM	
41. Haveri	A	7	1,437,860	76	1 Composite		Samraksha	
42. Kodagu	A	3	545,322	50				
43. Kolar	A	12	2,523,406	432	1 Composite		Myrada	
44. Koppal	A	4	1,193,496	119	1 Composite		Samraksha	
45. Mandya	A	7	1,761,718	165	1 Composite		KHPT/Gelaya	
46. Mysore	A	7	2,624,991	163	CBO		Gelaya/KHPT	
47. North Kannada	A	11	1,353,299	107				
48. Raichur	A	6	1,648,212	86	1 Composite		Samraksha	
49. Shimoga	C	9	1,639,595	233	1 Composite		KHPT	
50. South Kannada	A	8	1,896,403	272	CBO+ Composite		Aasare + Cardis	
51. Tumkur	A	9	2,579,516	219	CBO+ Composite		Amardeep + Cardis	
52. Udupi	A	4	1,109,494	145	CBO+ Composite		Snehasangama guard +?	
**Kerala: Hi Vul (14 Districts)**			**31,841,374**		**7 Exclusive + 3 Composite**	**2+?/ ?**		
53. Alappuzha	B							
54. Ernakulam	A							
55. Idukki	C							
56. Kannur	C							
57. Kasargod	C							
58. Kollam	C							
59. Kottayam	B							
60. Kozhikode	B						FIRM	
61. Malappuram	C							
62. Palakkad	C							
63. Pathanamthitta	B							

64. Thiruvananthapuram	B						FIRM	
65. Thrissur	B						FIRM	
66. Wayanad	C							
**Lakshwadeep: Vul (1 District)**			**60,650**		**NONE NEEDED**	**0+?/ ?**		
67. Lakshwadeep	C		60,650			0		
**Pondicherry: Concentd (4 Districts)**			**974,345**		**No Exclusive + 1 Composite**	**0+?/ ?**		
68. Karaikal	D			1000?		1/?	Sahodaran	
69. Mahe	B							
70. Pondicherry (City)	A			4000?		1/?	Sahodaran	
71. Yanam	D							
**Tamil Nadu[1]: Hi Prev (30 Districts)**			**62,405,679**	**107,295?**	**10 Exclusive + 20 Composite**	**26+?/10,901?**		
72. Chennai	A					2+?/5000+?	Sahodharan, SWAM, THAA, Acuagul, ICWO	
73. Coimbatore	A	Yes		NMCT: 1822; Other: 13,600	CBO+ Composite	1/2044+	Nesam (NFI), NMCT (TAI)	
74. Cuddalore	A	Yes		4200?	CBO+ Composite	1/?	Pasam	
75. Dharmapuri	A	Yes		RIDO: 130	CBO+ Composite	1/144	Anbalyam, RIDO (TAI)	
76. Dindigul	B	Yes		6250		1/508	Namm (NFI)	
77. Erode	D	Yes		CARE: 827; Other: 6750		2/810+385+?	MSMS (NFI), Erode District Aravanigal Association; CARE (TAI)	
78. Kanchipuram	A	Yes		ARM: 3,588		1+?/1703	Hand, TAA, Suder Foundation; ARM (TAI)	Only ARM is funded.
79. Kanyakumari	A	Nagerkovil		2750?		1/525	Kumari	
80. Karur	A	Yes		2545?		1/?	Wheel	
81. Krishnagiri	D	Yes		3500?		1/900	Krishnagri Aas	
82. Madurai	A	Yes		ISM: 548; Other: 14,250		1/1807+1095	Gokulum (NFI), ISM (TAI)	
83. Nagapattinam	A	Mayavara m		Other: 3680		1/624	Kits&Kins (NFI)	
84. Namakkal	A	Yes		WORD: 177; Other:7800		1/268	Nanban, WORD (TAI)	
85. Nilgiris	D	Yes		Other: 7500		1/?	Rainbow	

[1] Size estimations for TAI (Avahan) represent high -risk MSM. There may be more under different definitions. Other estimates come from the mappi ng exercise conducted by Indian Marketing Research Berew, funded by APAC (figures were provided by Federation for Male Social and Sexual Health Programme).

86. Perambalur	A	Yes		Other: 3500		1/?	Sanghamam	
87. Pudukkottai	A	Yes		3000?		?	Udhayam	Not funded
88. Ramanathapuram	A	Yes		Other: 4400		?	Bright	Not funded
89. Salem	A	Yes		PDI: 1,123		1/900+1490	Vadamalar, PDI (TAI)	Onlhy PDI is funded
90. Sivaganga	C	Yes		Other: 3800		?	Saha	Not funded
91. Thanjavur	A	Kumbakon am		4000?		1/928	Liaas (NFI)	Lube
92. Theni	A	Yes		Arogyaagam: 109		1+?/236+?	Theni District Aravanigal Association, Care, Arogaayam (TAI)	Only Arogaayam is funded.
93. Thoothukudi	C	Yes		6000?		1/831	Love India (NFI)	Lube
94. Tiruchirappalli	A	Yes		11000?		1/942	Snegiytham (NFI)	Lube, no funding for TG CBO
95. Tirunelveli	A	Yes		4500?		1/912	Saral (NFI)	Lube
96. Tiruvallur	B	Yes		CHES: 2819		1/1336	PAA, CHES (TAI)	Only CHES is funded.
97. Tiruvannamalai	A	Yes		Other: 6750		1/?	Aravani Welfare Association, Hope	Not funded
98. Tiruvarur	A	Yes		Other: 3750		1/	Gandhi	Not funded
99. Vellore	C	Yes		GLOW: 2002		1/1649	MAAS, GLOW (TAI)	Only GLOW is funded.
100. Viluppuram	D			Other: 5000		?	VMMK (TNSACS-Supported)	
101. Virudhunagar	C	Yes		Other: 2400		1/?	Priyam	Not funded

State/District	Category (A-D)	Urban Centre?	Population: 2001	MSM-TG size estimation	NACO Estimate of TI needs/MSM	Number of Tis/ Popn covered	Implementing Organisation	What gaps?
**NORTHEAST**								
**Arunachal Pradesh: Vulnerable(16 Districts)**			**1,097,968**		**NONE NEEDED**	**0/0**		
102. Anjaw	C					0		
103. Changlang	C					0		
104. Dibang Valley	D					0		
105. East Kameng	D					0		
106. East Suiang	C					0		
107. Kurung Kumey	D					0		
108. Lohit	C					0		
109. Lower Dibang valley	D					0		
110. Lower Subansiri	D					0		
111. Papum Pare	C					0		
112. Tawang	D					0		
113. Tirap	C					0		
114. Upper Siang	D					0		
115. Upper Subansiri	D					0		
116. West Kameng	C					0		
117. West Siang	C					0		
**Assam (23 Districts)**			**26,655,528**		**6 Exclusive + 8 Composite**	**2+?/?**		
118. Barpeta	C							
119. Bongaigaon	C							
120. Cachas	C							
121. Carrang	D							
122. Dhemaji	C							
123. Dhubri	C							
124. Dibrugarh	D							
125. Goalpara	C							
126. Golaghat	C							
127. Hailakandi	D							
128. Jorhat	C							
129. Kamrup	B							
130. Karbi Anglong	C							

131. Karimganj	D							
132. Kokrajhar	C							
133. Lakhimpur	C							
134. Marigaon	D							
135. Nagaon	D							
136. Nalbari	D							
137. North Cachar Hills	D							
138. Sivasagar	C							
139. Sonitpur	D							
140. Tinsukia	D							
**Manipur[1]: Hi Prev (9 districts)**			**2,166,788**	**2,850?**	**No exclusive + 2 Composite**	**3/1550+?**		**1 TI is BMGF, 1 TI is SACS; 1 TI is unfunded**
141. Bishnupur	A						Aasha	
142. Chandel	A							
143. Churachandpur	A							
144. Imphal	A	Imphal				1000	Aasha / SASO, Maruploi FNDN	
145. Moreh	A							
146. Senapati	A							
147. Tamenglong	A							
148. Thoubal	C						Aasha	
149. Ukhrul	D							
**Meghalaya: Vulnerable (7 Districts)**			**2,318,822**		**1 Exclusive + 1 Compsite**	**0+?/ ?**		
150. East Garo Hills	C					0		
151. East Khasi Hills	C					0		
152. Jaintia Hills	C					0		
153. Ri Bhoi	C					0		
154. South Garo Hills	C					0		
155. West Garo Hills	C					0		
156. West Khasi Hills	C					0		
**Mizoram: Vulnerable (8 Districts)**			**888,573**		**NONE NEEDED**	**0+?/ ?**		
157. Aizawl	A					0		

[1] Size estimations for Manipur taken from Avahan and represent “high risk MSM”. There may be more under different definitions.

158. Champhai	A					0		
159. Kolasib	A					0		
160. Lawngtlai	C					0		
161. Lunglei	D					0		
162. Mamit	C					0		
163. Saiha	A					0		
164. Serchhip	A					0		
**Nagaland[1]: Hi Prev (11 Districts)**			**1,990,036**		**No Exclusive + 1 Composite**	**1/?**		
165. Dimapur	A					300	Guardian Angel	
166. Kohima	A					0		
167. Kiphera	A							
168. Longleng	A							
169. Mokokchung	A					0		
170. Mon	A					0		
171. Peren	A							
172. Phek	D					0		
173. Tuensang	A					0		
174. Wokha	C					0		
175. Zunheboto	A					0		
**Sikkim: Vulnerable (4 Districts)**			**540,851**		**NONE NEEDED**	**0+?/ ?**		
176. East Sikkim	D					0		
177. North Sikkim	D					0		
178. South Sikkim	B					0		
179. West Sikkim	B					0		
**Tripura: Vulnerable (4 Districts)**			**3,199,203**		**1 Exclusive + 1 Composite**	**0+?/ ?**		
180. Dhalai	D					0		
181. North Tripura	B					0		
182. South Tripura	C					0		
183. West Tripura	C					0		

1 Size estimations for Nagaland taken from Avahan and represent "high risk MSM". There may be more under different definitions.

State/District	Category (A-D)	Urban Centre?	Population: 2001	MSM-TG size estimation	NACO Estimate of TI needs/MSM	Number of Tis/ Popn covered	Implementing Organisation	What gaps?
**EAST**								
**Bihar: HI Vul (38 Districts)**			**82,998,509**		**19 Exclusive + 29 Composite**	**6 (SACS-5, UNDP-1)/?**		
184. Araria	C							
185. Arval	C							
186. Aurangabad	D							
187. Banka	C							
188. Begusarai	C							
189. Bhagalpur	D							
190. Bhojpur	C							
191. Buxar	D						PLUS	
192. Darbhanga	C							
193. East Champaran	A							
194. Gaya	C							
195. Gopalganj	C						PLUS	
196. Jamui	C							
197. Jehanabad	C							
198. Kaimur (Bhabua)	B							
199. Katihar	C							
200. Khagaria	A							
201. Kishanganj	A						Pratham	
202. Lakhisarai	C							
203. Madhepura	C							
204. Madhubani	A							
205. Munger	C						Maghad Gramin Seva Sangh	
206. Muzaffarpur	A	Mzffrpr					PLUS	
207. Nalanda	D							
208. Nawada	C							
209. Patna	B	Patna					PLUS, All India Dav Society, Pushpa Bharati Seva Samaj Rachna	
210. Purnia	A							
211. Rohtas	C							
212. Saharsa	C							

213. Samastipur	C						PLUS	
214. Saran	C							
215. Sheikhpura	C							
216. Sheohar	C							
217. Sitamarhi	A							
218. Siwan	D						PLUS	
219. Supaul	C							
220. Vaishali	C						PLUS	
221. West Champaran	A							
**Chhattisgarh: Hi Vul (16 Districts)**			**20,833,803**		**5 Exclusive + 7 Composite**	**0+?/ ?**		
222. Bastar	A					0		
223. Bilaspur	D					0		
224. Dantewada	C					0		
225. Dhamtari	C					0		
226. Durg	D					0		
227. Janjgir-Champa	D					0		
228. Jashpur	C					0		
229. Kanker	D					0		
230. Kawardha	C					0		
231. Korba	C					0		
232. Korea	D					0		
233. Mahasamund	A					0		
234. Raigarh	C					0		
235. Raipur	C					0		
236. Rajnandgaon	D					0		
237. Surguja	C					0		
**Jharkand: Hi Vul (22 Districts)**			**26,945,829**		**6 Exclusive + 10 Composite**	**1 (SACS)/?**		
238. Bokaro	C					0		
239. Chatra	C					0		
240. Deoghar	D					0		
241. Dhanbad	C					0		
242. Dumka	D					0		
243. East Singhbhum	D	Jam				1	Tata Steel	
244. Garhwa	D					0		
245. Giridih	C					0		
246. Godda	C					0		
247. Gumla	D					0		

248. Hazaribag	D					0		
249. Jamtara	D					0		
250. Koderma	D					0		
251. Latehar	D					0		
252. Lohardaga	D					0		
253. Pakur	C					0		
254. Palamu	D					0		
255. Ranchi	C					0		
256. Sahibganj	D					0		
257. Seraikela	D					0		
258. Simdega	D					0		
259. West Singhbhum	C					0		
**Orissa: Hi Vul (30 Districts)**			**36,804,660**	**1000-1200?**	**8 Exclusive + 14 Composite**	**8-10(SACS)+ 1(Oxfam)/230?**		
260. Angul	C							
261. Balangir	C							
262. Baleswar	C							
263. Bargarh	D							
264. Bhadrak	C					100?	Fellowship, Saraswati, Santiseva	
265. Boudh	C							
266. Cuttack	A							
267. Deogarh	C							
268. Dhenkanal	D							
269. Gajapati	D							
270. Ganjam	A							
271. Jagatsinghapur	C							
272. Jajpur	C							
273. Jharsuguda	D							
274. Kalahandi	C			230?		130?	Bhawanis	
275. Kandhamal	D							
276. Kendrapar	C							
277. Kendujhar	C							
278. Khordha	B							
279. Koraput	C							
280. Malkangiri	C							
281. Mayurbhanj	C							
282. Nabarangapur	C							
283. Nayagarh	C							

284. Nuapada	C							
285. Puri	C							
286. Rayagada	C							
287. Sambalpur	A							
288. Subarnapur	C							
289. Sundergarh	C							
**West Bengal: Hi Vul (18 Districts)**			**80,176,197**	**15,000-20,000?**	**18 Exclusive + 25 Composite**	**1(SACS)+6/ 10,000?**		
290. Bankura	C					0		Networking, needs asst
291. Bardhaman	B	Burdwan, Durgapur, Asansol					Swapnil / MANAS Bangla	Footnote[1]
292. Birbhum	C					0		Needs assessment
293. Cooch Behar	C	Cooch Behar					Mitjyu / MANAS Bangla	Needs assessment
294. Darjeeling	C	Darj, Siliguri					Mitjyu / MANAS Bangla	Footnote
295. East Midnapore	C	Haldia				0	MANAS Bangla	Needs assessment
296. Hooghly	A	Chandann agar, Bandel, Serampore					Amitie / MANAS Bangla	Footnote
297. Howrah	C	Howrah					Amitie / MANAS Bangla	Footnote
298. Jalpaiguri	B	Jalpaiguri					Mitjyu / MANAS Bangla	Footnote
299. Kolkata	A	Kolkata					Bandhan, Koshish, MANAS Bangla/ Pratyay, PLUS Kolkata, Kolkata Rishta	Footnote
300. Malda	C					0		Needs assessment
301. Murshidabad	B	Behramp ur					Swikriti / Sangram, MANAS BAngla	Needs assessment
302. Nadia	C	Ranaghat				0	MANAS Bangla	Needs assessment
303. North 24 Parganas	C	Bongaon, Barasat, Dumdum					Swikriti / Prantik / MANAS Bangla	Footnote
304. North Dinajpur	C					0		Networking, needs asst
305. Purulia	C					0		Networking, needs asst
306. South 24 Parganas	C	Baruipur					Astitva / MANAS Bangla	Footnote
307. South Dinajpur	C					0		Networking, needs asst
308. West Midnapore	B	Midnapur, Kharagpur				0	MANAS Bangla	Needs assessment

[1] MOUs, vision, mission of network; policy finalisation; livelihood options; skilled staff; training (including leadership); skills building; GPs; advocacy.

State/District	Category (A-D)	Urban Centre?	Population: 2001	MSM-TG size estimation	NACO Estimate of TI needs/MSM	Number of Tis/ Popn covered	Implementing Organisation	What gaps?
**NORTH**								
**Chandigarh: Vul (1 District)**			**900,635**		**No exclusive + 1 Composite**	**0+?/ ?**		
309. Chandigarh	A		900,635	1000?			Deepshikha Samiti/ Pahal Foundation	
**Delhi: No Category (9 Districts)**			**13,850,507**		**3 Exclusive + 10 Composite**	**3+?/ ?**		
310. Central Delhi	B					3000?	Naz India (NI), Bard	Lube availability
311. East Delhi	B					4500?	NI, SaharaTG	No TG CBO
312. New Delhi	A					3000?	NI	
313. North Delhi	B					2500?	NI, Akansha	
314. North East Delhi	B					1500?	Aradhya	
315. North West Delhi	C					2500?	Aradhya, Love Life Society	
316. South Delhi	C					3000?	NI	
317. South West Delhi	C					2000?	Mitr	
318. West Delhi	C					2000?	Mitr	
**Haryana: Vulnerable (20 Districts)**			**21,144,564**		**5 Exclusive + 6 Composite**	**0+?/ ?**		
319. Ambala	C					0		
320. Bhiwani	C					0		
321. Faridabad	C					0	Pahal Foundation	No TI started
322. Fatehabad	C					0		
323. Gurgaon	C					0	Pahal Foundation	No TI started
324. Hisar	C					0		
325. Jhajjar	A					0		
326. Jind	C					0		
327. Kaithal	C					0		
328. Karnal	C					0		
329. Kurukshetra	C					0		
330. Mahendragarh	C					0		
331. Mewat	C					0		
332. Panchkula	C			1000?		0	Virat Foundation	No TI started
333. Panipat	C					0		
334. Rewari	C					0		

335. Rohtak	C					0		
336. Sirsa	C					0		
337. Sonipat	C					0		
338. Yamunanagar	C					0		
**Himachal Pradesh: Hi Vul (12 Districts)**			**6,077,900**		**1 Exclusive + 2 Composite**	**0+?/ ?**		
339. Bilaspur	D					0		
340. Chamba	C					0		
341. Hamirpur	D					0		
342. Kangra	D					0		
343. Kinnaur	C					0		
344. Kullu	C					0		
345. Lahaul & Spiti	D					0		
346. Mandi	C					0		
347. Shimla	C					0		
348. Sirmaur	D					0		
349. Solan	D					0		
350. Una	C					0		
**Jammu & Kashmir: Vulnerable (15 Districts)**			**10,143,700**		**2 Exclusive + 4 Composite**	**0+?/ ?**		
351. Anantnag	C							
352. Baramulla	D							Generally very
353. Budgam	D	?					Humsafar Kashmir	conservative society.
354. Chilas (Gilgat)	C							
355. Doda	B							NGO/CBO registration is
356. Jammu	C							very time consuming.
357. Kargil	C							
358. Kathua	D							
359. Kupwara	C							
360. Leh	D							
361. Poonch	D							
362. Pulwama	D							
363. Rajauri	C							
364. Srinagar	C	?					Humsafar Kashmir	

365. Udhampur	D							
**Punjab: Hi Vul (17 Districts)**			**24,358,999**		**6 Exclusive + 8 Composite**	**0+?/ ?**		
366. Amritsar	A							
367. Bathinda	C							
368. Faridkot	C							
369. Fatehgarh Sahib	C							
370. Ferozepur	C							
371. Gurdaspur	C							
372. Hoshiarpur	C							
373. Jalandhar	C							
374. Kapurthala	C							
375. Ludhiana	A							
376. Mansa	C							
377. Moga	C							
378. Muktsar	C							
379. Nawanshahr	C							
380. Patiala	C			1000?			Pahal Foundation	No TI started
381. Rupnagar	B							
382. Sangrur	C							
**Rajasthan: Hi Vul (32 Districts)**			**56,507,188**		**13 Exclusive + 19 Composite**		**0+?/ ?**	
383. Ajmer	A			1000?			Pahal Foundation, Manthan	
384. Alwar	B							
385. Banswara	B							
386. Baran	D							
387. Barmer	B							
388. Bharatpur	C							
389. Bhilwara	C							
390. Bikaner	D							
391. Bundi	D							
392. Chittorgarh	D							
393. Churu	D							
394. Dausa	D							
395. Dholpur	C							
396. Durgarpur	D							

397. Ganganagar	A							
398. Hanumangarh	C							
399. Jaipur	B			1000?			Pahal Foundation	No TI started
400. Jaisalmer	D							
401. Jalor	D							
402. Jhalawar	A							
403. Jhunjhunu	D							
404. Jodhpur	D							
405. Karauli	C							
406. Kota	D							
407. Nagaur	D							
408. Pali	D							
409. Rajsamand	C							
410. Sawai Madhopur	D							
411. Sikar	D							
412. Sirohi	B							
413. Tonk	B							
414. Udaipur	C							
**Uttaranchal: Hi Vul (13 Districts)**			**8,489,349**		**2 Exclusive + 3 Composite**	**0+?/ ?**		
415. Almora	C					0		
416. Bageshwar	C					0		
417. Chamoli	C					0		
418. Champawat	C					0		
419. Dehradun	C					0		
420. Haridwar	C					0		
421. Nainital	C					0		
422. Pauri Garhwal	C					0		
423. Pithoragarh	C					0		
424. Rudraprayag	C					0		
425. Tehri Garhwal	C					0		
426. Udham Singh Nagar	C					0		
427. Uttarkashi	C					0		
**Uttar Pradesh: Hi Vul (71 Districts)**			**166,197,921**		**26 Excl + 48 composite**	**0+?/ ?**		
428. Agra	B	Yes				?/1000?	Nai Sebra	Condom and lube

429. Aligarh	C	Yes						
430. Allahabad	C	Yes				?/1000?	Vishvas	Condom and lube
431. Ambedkar Nagar	C							
432. Auraiya	D							
433. Azamgarh	C							
434. Bagpat	C							
435. Bahraich	C							
436. Ballia	C					?/1000?	Nai Kiran	Condom and lube
437. Balrampur	A							
438. Banda	A							
439. Barabanki	C					?/1000?	Mitr	Condom and lube
440. Bareilly	C							
441. Basti	C							
442. Bijnor	C							
443. Budaun	C							
444. Bulandshahr	C							
445. Chandauli	C							
446. Chitrakoot	C							
447. Deoria	C							
448. Etah	A							
449. Etawah	A							
450. Faizabad	C							
451. Farrukhabad	C							
452. Fatehpur	C							
453. Firozabad	C							
454. Gautam Buddha Nagar	C							
455. Ghaziabad	C					?/1000?	Besara	Lubes, condom, STI Tx
456. Ghazipur	B							
457. Gonda	C							
458. Gorakhpur	C							
459. Hamirpur	C							
460. Hardoi	C					?/1000?	Parevertah	Lubes, condom, STI Tx
461. Hathras	D							
462. Jalaun	C							

463. Jaunpur	C					?/1000?	Ham Khayal	Lubes, condom, STI Tx
464. Jhansi	C							
465. Jyotiba Phule Nagar	C							
466. Kannauj	C							
467. Kanpur Dehat	C							
468. Kanpur Nagar	C					?/1000?	Hasrat	Lubes, condom, STI Tx
469. Kaushambi	C							
470. Kheri	C							
471. Kushinagar	C							
472. Lalitpur	C							
473. Latehar	A							
474. Lucknow	C					?/5000?	Bharosa	Lubes, condom, STI Tx
475. Maharajganj	C							
476. Mahoba	C							
477. Mainpuri	C							
478. Mathura	C							
479. Mau	C							
480. Meerut	C							
481. Mirzapur	C							
482. Moradabad	C							
483. Muzaffarnagar	C							
484. Pilibhit	C							
485. Pratapgarh	C							
486. RaeBareli	C							
487. Rampur	C							
488. Saharanpur	C							
489. Sant Kabir Nagar	C							
490. Sant Ravidas Nagar	C							
491. Shahjahanpur	C							
492. Shravasti	C							
493. Siddharthnagar	C							
494. Sitapur	C							
495. Sonbhadra	C							
496. Sultanpur	C							
497. Unnao	C							
498. Varanasi	C					?/1000?	Asha	Lubes, condom, STI Tx

State/District	Category (A-D)	Urban Centre?	Population: 2001	MSM-TG size estimation	NACO Estimate of TI needs/MSM	Number of Tis/ Popn covered	Implementing Organisation	What gaps?
**WEST**								
**Dadra & Nagar Haveli: Vul (1 District)**			**220,490**		**NONE NEEDED**	**0+?/ ?**		
499. Dadra & Nagar Haveli	C		220,490			0		
**Daman & Diu: Vul (2 Districts)**			**158,204**		**NONE NEEDED**	**0+?/ ?**		
500. Daman	C					0		
501. Diu	B					0		
**Goa: Concentrated (2 Districts)**			**1,347,668**		**No exclusive + 1 Composite**	**1+?/ ?**		
502. North Goa	A			7000?/1000?		1/3000?	Humsafar Goa?	Needs upscaling in
503. South Goa	A			total		Total	Humsafar Goa?	general
**Gujarat: Concentrated (25 Districts)**			**50,671,017**		**12 Exclusive + 7 Composite**	**5+?**		
504. Ahmedabad	B	Yes			1 CBO	1+?/2800MSM? + 200 TG?	Chuval Gram Vikas Trust	
505. Amreli	B	Yes						
506. Anand/Nadiyad	B	Yes						
507. Banas Kantha	A	Yes						
508. Bharuch	B	Yes						
509. Bhavnagar	B	Yes			CBO+ Composite		Lakshya Trust + Bhavnagar Bloodbank	NA study in progress
510. Dahod	B	Yes						
511. Gandhinagar	B	Yes						
512. Jamnagar	B	Yes			1 CBO		Lakshya Trust	NA study in progress
513. Junagadh	B	Yes						
514. Kachchh	C	Yes						
515. Kheda	B	Yes						
516. Mahesana	A	Yes			1 CBO	1/2000MSM+TG?	Chuval Gram Vikas Trust	
517. Narmada	B	Yes						
518. Navsari	A	Yes						
519. Panch Mahals	B	Yes						

520. Patan	A	Yes						
521. Porbandar	B	Yes						
522. Rajkot	B	Yes			1 CBO	1/4000MSM?	Lakshya Trust	
523. Sabar Kantha	B	Yes						
524. Surat	A	Yes			1 CBO	1/5000MSM?	Lakshya Trust	
525. Surendranagar	A	Yes						
526. The Dangs	A	Ahwa						
527. Vadodara	B	Yes			1 CBO	1/7000MSM?	Lakshya Trust	
528. Valsad/Vapi	A	Yes					Lakshya Trust	NA study in progress
**Madhya Pradesh: Hi Vul (48 Districts)**			**60,348,023**		**14 Exclusive + 23 Composite**	**0+?/ ?**		
529. Anuppur	D							
530. Ashoknagar	D							
531. Balaghat	D							
532. Barwani	C							
533. Betul	C							
534. Bhind	C							
535. Bhopal	A				1000?		Shringar	
536. Burhanpur	D							
537. Chhatarpur	D							
538. Chhindwara	C							
539. Damoh	D							
540. Datia	D							
541. Dewas	C							
542. Dhar	C							
543. Dindori	D							
544. Guna	D							
545. Gwalior	C							
546. Harda	C							
547. Hoshangabad	C							
548. Indore	A				1000?		Shringar	
549. Jabalpur	A							
550. Jhabua	D							
551. Katni	C							

552. Khandwa	C							
553. Khargone	B							
554. Mandla	D							
555. Mandsaur	A							
556. Morena	C							
557. Narsinghpur	D							
558. Neemuch	B							
559. Panna	D							
560. Raisen	D							
561. Rajgarh	C							
562. Ratlam	C				1000?		Shringar	
563. Rewa	B							
564. Sagar	C							
565. Satna	C							
566. Sehore	C							
567. Seoni	C							
568. Shahdol	C							
569. Shajapur	C							
570. Sheopur	D							
571. Shivpuri	C							
572. Sidhi	D							
573. Tikamgarh	D							
574. Ujjain	A							
575. Umaria	D							
576. Vidisha	D							
**Maharashtra[1]: Hi Prev (35 Districts)**			**96,878,627**		**16 Exclusive + 39 Composite**	**6+?/ ?**		
577. Ahmednagar	A	6 Towns	40,40,642	534	*Samapathik estn*	1/534	Snehalaya (Pathfinder), Udan Savli, Astitva/ Kalaga	Only Snehalaya TI started
578. Akola	A							
579. Amravati	B						Udaan/Humsafar	

[1] Size estimations taken from Avahan and represent "high risk MSM". (Sources are Pathfinder PSA Estimation August 2006; Report of mapping and needs assessment of MSM in Pune by Humsafar & Samapathik, June 2005.) There may be more under different definitions.

580. Aurangabad	A			1000?/?				
581. Beed	A	Beed and Parli	21,61,250	791		1/791	Gramin Vikas Mandal(Pathfinder)	
582. Bhandara	A							
583. Buldhana	B							
584. Chandrapur	A			1000?/?				
585. Dhule	A							
586. Gadchiroli	A							
587. Gondia	B							
588. Hingoli	A							
589. Jalgaon	A	4 Towns	36,82,690	594		1/594	Godavari Foundation (Pathfinder) collaborating with Udaan; Jaagruthi Trust	
590. Jalna	A			500?/?			Udaan, Jagruti/Humsafar	
591. Kolhapur	A	2 Towns	35,23,162	596		1/596	MSPSS (Pathfinder)	
592. Latur	A	Udgir	20,80,285	123		1/123	Grameen Vikas Mandal(Pathfinder)	
593. Mumbai	A			65,000?/4500?		4+?/ ?	Astitva, Dai Welfare Society, Humsaaya, Humsafar Trust, Sakhi Char Chowghi, Udaan	
594. Mumbai Suburban	A							
595. Nagpur	A			1000?/?			Sarathi	Upscaling needed
596. Nanded	A			1000?/1000?	*Sarthi estn*		Udaan-Maya/Astitva	
597. Nandurbar	A	2 Towns	13,11,709	41		1/41	Astitva, Shriram Ahirrao Memorial Trust (Pathfinder)	Map/baseline in progress; Pathfinder project to be started.
598. Nashik		3 Towns	49,93,796	172		1/172	Pravara Medical Trust (Pathfinder), Astitva-Paro	
599. Osmanabad	A						Udan-Rakshak	
600. Parbhani	A	2 Towns	15,27,715	202		1/202	Socio-Economic Development Trust(Pathfinder)	

601. Pune	A	1 Town	72,32,555	3200	*Humsafar & Samapathik estn*	2/3200	Samapathik; Udaan, and Samabhavna (Pathfinder)	Pathfinder project just starting.
602. Raigad	A		22,07,929				HUmraahi Trust; Pathfinder	PSA Baseline Study to be conducted during April-May 2007
603. Ratnagiri	A							
604. Sangli	A			1000?/?			Mooknayak	
605. Satara	A			1000?/?			Mooknayak	
606. Sindhudurg	B							
607. Solapur	A							
608. Thane	A			10,000?/5000?			Jugnu Trust; Astitva	Map/baseline in progress
609. Wardha	A							
610. Washim	B							
611. Yavatmal	A	3 Towns	24,58,271	204		1/204	Grameen Samasya Mukti Trust (Pathfinder)	

This annexure is an excerpt from the Infosem document, “Scaling Up Interventuions for MSM and Transgender Populations in India.”

ANNEXURE 13

Modular Costing Framework for IDU TIs

Note:

- Costs are given as cost heads and some of the cost heads have sub-heads also.
- Budgeting is to be finalised based on needs which have been identified through the baseline; once reflected in a proposal, this may then be discussed by the NGO representative, NGO advisor and the Finance Officer of SACS. In the case of states that have management support agencies for Targeted Interventions (TSU), the budget is to be finalised by that agency in consultation with the NGO and designated officers from SACS.

The following tables are of cost heads common for all categories of interventions.

This costing is worked out based the project size defined in the current costing guidelines, i.e. for 1,000 IDUs.

Table A1 Programme Management Costs

**A1**	**Recurrent C osts**		
	**Cost head**	**Sub-head**	**Base cost**
A1.1	**Honorarium for Project Director of TI**		Rs. 40,000 per annum
	The Project Director is expected to provide overall guidance to the project and also interface between the Governing Board of the NGO and the project . The honorarium is paid for this responsibility. *This honorarium is to be paid in two equal instalments of Rs. 20,000 each. The first instalment is paid mid -year after the mid -year review is completed and the report of mid -year review submitted. The second instalment is paid after annual evaluation is completed a nd the report on evaluation is submitted.*		
A1.2	**Recruitment cost**		Rs. 5,000
	Recruitment cost is paid towards expenses associated with recruitment of staff. This will happen at the beginning of the project and also during the project if new staff are recruited to replace any team members who leave the project. The variation over base cost can be allocated after looking at the possible expenses and the market rates for such items.		
A1.3	**Salary of Project Manager**		Rs. 8,000 per month
A1.4	**Salary of Accountant/Office Support Staff**	2 posts	Rs. 5,000 per month
A1.5	**Travel cost for administrative purpose**		Rs. 800 per month
	Base cost is to be budgeted for interventions which are closer to state head quarters. Progressively higher amounts must be budget ed for interventions which are in other parts of the state, depending on the distance.		
A1.6	**Rent**		Rs. 4,000 per month
	Rent should be budgeted according to the prevailing costs in locations where the Project Office is set up. Thus in bigger cities highe r levels of rent should be budgeted.		
	**Office expenses**		
A1.7		Water and Electricity	Rs. 12,000 per annum
A1.8		Stationery and photocopying	Rs. 9,000 per annum
A1.9		Office maintenance	Rs. 7,200 per annum
A1.10		Phone, fax, postage and courier	Rs. 18,000 per annum
A1.11		Internet	Rs. 6,000 per annum
	The variations in these sub -heads should be budgeted according to the prevailing market rates for these services		
**A2**	**One-Time Costs**		
A2.2	**Office infrastructure**		Rs. 20,000
A2.3	**Computer and peripherals**		Rs. 40,000
A2.4	**Audio-visual and other equipment**		Rs. 20,000
A2.5	**Clinic set-up costs**		Rs. 10,000
	The costs for A2.2, A2.3 and A2.4 are paid at the beginning of the project. After three years, need for replacing or adding infrastructure can be revie wed and the necessary allocation made.		

Table B1 Programme Delivery Costs

B1	Recurrent Costs		
	**Cost head**	**Sub-head**	**Base cost**
B1.1	**Outreach Workers salary**		Rs. 5,000 per month
	The number of ORWs in any project will be based on the estimated number of the HRG to be covered by the TI by the end of the year. The ratio is 1 ORW for every 200 vulnerable persons from IDUs.		
B1.2	**Peer Educator incentives**		Rs. 1,500 per month
	The number of Peer Educators in any project will be based on the estimated number of the HRG to be covered. The ratio is 1 PE for every 40 IDUs as they are a hidden marginalised community. The SACS can use the above as a guideline and may modify the ratio if more ORWs or PEs are needed, based on the distribution of IDUs or other factors.		
B1.3	**Counsellor (full-time) salary**		Rs. 6,500 per month
B1.4	**Peer Counsellor salary**		Rs. 3,500 per month
	One peer counsellor (role model) who is from the target community with a minimum of one years involvement in outreach and befriending of IDUs. The peer counsellor is to assist the professional counsellor in the DIC and field. Two peer counsellors per DIC.		
B1.5	**BCC development**		Rs. 10,000 per annum
B1.6	**Travel costs for programme purposes**		Rs. 500 per month<br>X number of Outreach Workers
	The costing for travel uses the number of outreach workers as a factor, but this amount is for travel for any team member for programme purpose and not only for outreach workers.		
	**Community mobilisation**		
B1.5		Group Discussions / Focus Group Discussions	Rs. 6,000 per annum
B1.6		Community events	Rs. 7,500 per annum
B1.7		Advocacy	Rs. 15,000 per annum
B1.8		Networking	Rs. 15,000 per annum
B1.9	**Training**		Rs. 12,500 per annum from Y2
	For the first year all the trainings will be organised by SACS/PSU and all expenses relating to travel, stay and other training costs will be met by the SACS.		
B1.10	**Training of peers**		Rs. 7,500 per annum
B1.11	**Training of volunteers**		Rs. 5,000 per annum
B1.12	**Exposure visit**		Rs. 15,000 per annum
B1.13	**Monitoring and Evaluation**		Rs. 20,000 per annum
B1.14	**Programme planning for next year**		Rs. 5,000 per annum
B1.16	**PLWA support**		Rs. 40,000 per annum

Table B2 Theme-Specific Essential Costs for IDUs

B2	Recurrent costs	Base cost	Ceiling	Notes
B2 IDU.1	**De-addiction MSJE**	Rs. 2,000 per case	5%-10% of target group	Addiction is a relapse-prone disease. 15 days indoor detoxification at MSJE and other de-addiction centres. The money is for food and medicines. Most IDUs suffer from TB, malnutrition, other skin infections and abscesses. Most centres do not admit them as treatment can be life -threatening and expensive.
B2 IDU.2	**Substitution cum detoxification in the community**	Rs. 7,500 -9,500 per case	10% -20% of target group	To reduce the pool of infection. To bring about BC C, a client is stabilised and detoxified within 6-9 months.
B2 IDU.3	**Abscess management**	Rs. 600 per case	5%-10% of target group	For dressings, antibiotics, etc. This cost will decline drastically as IDUs start safe injecting practices. In the first yea r the budget is based on actual needs and during the following years the budget comes down significantly.
B2 IDU.4	**Abscess prevention**	Rs. 730 per IDU per annum	All those in the needle exchange programme. 100% of the HRG.	Alcohol/cotton swipes/water pouches/betadine
B2 IDU.5	**Needles and syringes**	Rs. 750 per IDU per annum	60% of the target group	
B2 IDU.6	**STI medicine**	Rs. 125 per case	% of HRG as reflected in the baseline/ sentinel surveillance	The partners of IDUs would also need to have access to sy ndromic management of STIs. It is recommended that once the number of HRG needing STI services is decided based on baseline/surveillance , an equal number (or a minimum of half that number) of partners is included in the costing for STI drugs.
B2 IDU.7	**Services of doctor**	Rs. 6,000 per month	Part- time doctor	For primary health care, syndromic management of STIs and OST dosage.
B2 IDU.8	**Services of Nurse**	Rs. 5,000 per month	Full-time	For abscess management, delivery and monitoring OST services.
B2 IDU.9	**Drop-In Centre**	Rs. 50,000	Serves as a hub for continuum of services	

Costing

- 1 syringe and 2 needles per client every 2 days to ensure that every injecting episode is safe and there is no sharing among different members of the network.
- Divide users accessing clean needles, abscess management, oral substitution, and referral to de-addiction. Rationale for this is 60-80% coverage to reverse trends of epidemic. The mix should have a minimum of 60% on NSEP.
- Oral substitution and de-addiction to MSJE and other centres should constitute a further 20% of participants so as to ensure at least 80% coverage of IDUs.
- On an average costing for oral substitution (2.2 - 4.4 mg per day for rest of India and 6mg for north-east) for 6-9 months per client @ Rs. 14.70 for 2 mg. The SACS/TSU will work with the TI partner to decide OST costing and dosage.

ANNEXURE 14

Staffing and Running a Drop-In Centre (DIC)

Staff Roles and Responsibilities

Project Coordinator
■ Supervision of field and clinic activities on regular basis<br>■ Overseeing MIS<br>■ Development of capacity building and sustainability strategy<br>■ Capacity building of staff and organisation<br>■ Helping to develop organisational policies and plans<br>■ Development and monitoring of weekly work plan as per the performance indicators for Outreach Workers and counsellors<br>■ Arrangement of weekly and monthly meetings to identify shortfalls and to evolve corrective measures/plan of action<br>■ Facilitating advocacy meetings and focus group discussions in the field<br>■ Continuous analysis of the project activities as to costs incurred to ensure cost-effective implementation<br>■ Liaison with funding agency<br>■ Clinic and field visit at least thrice weekly<br>■ Meeting with governing body<br>■ Monitoring and Evaluation

Counsellor
■ Development of BCC strategy<br>■ Development of sub-group-specific IEC<br>■ Patient management, ensure partner notification, ensure follow-up of recurrent cases and one-to-one counselling of STI cases<br>■ Pre- and post-test counselling<br>■ Family counselling<br>■ Community counselling programme<br>■ Counselling patients with high risk behaviours<br>■ Meet with community and staff<br>■ Facilitate the process of capacity building of ORWs, including pre- and post-training assessment<br>■ Monitoring the weekly work plan of ORWs<br>■ Coordination in creating linkages/networking<br>■ Helping Project Coordinator in creating appropriate strategies for advocacy environment<br>■ Facilitation of advocacy meeting, focus group discussion and awareness campaigns<br>■ Field visit on days when not needed in clinic<br>■ Maintenance of registers<br>■ Recording feedback from the community<br>■ Condom promotion, demonstration and distribution, including social marketing<br>■ Assist Project Coordinator in building effective field team

Doctor
■ Treatment (General, STI, HRB, Opportunistic Infection)<br>■ Taking of exposure history from the patient<br>■ Advice for investigation and referral<br>■ Motivating the patient regarding follow-up, partner notification

Outreach Worker (ORW)
■ Lead the Field Team<br>■ Field visit, awareness generation and field counselling<br>■ Development of work plan<br>■ Organising advocacy meetings<br>■ Facilitating networking among partner NGOs, horizontal linkages<br>■ Organizing AV Programme, influencers meeting, quiz contest and talking doll, camp, etc.<br>■ Conducting group sessions<br>■ Development of list of target area<br>■ Rapport building<br>■ Listing and meeting with local private practitioners of the target area<br>■ Identification of peer volunteers, stakeholders, condom outlets<br>■ Facilitating the process of capacity building of peer volunteers<br>■ Monitoring and supervision of peer volunteers<br>■ Meeting with the peer volunteers once a week<br>■ Responsible for weekly report writing, record keeping, MIS<br>■ Maintaining the stocks (medicine, condoms, IEC materials)<br>■ Recording feedback from the community

Peer volunteers
■ Rapport building<br>■ NSEP/safer injecting practices<br>■ Dissemination of message, information about programme services<br>■ Distribution of IEC materials<br>■ Condom distribution, including social marketing<br>■ Motivating IDUs towards STI treatment and safer sex practice<br>■ Referral services to VCTC, DOTS and other health care services

Accountant
■ Field visit once weekly<br>■ Disbursement of salaries<br>■ Meeting with governing body<br>■ Preparation of appointment letters for new staff in consultation with general secretary and Project Coordinator<br>■ Maintaining indent file, requisition slip, order file, quotation file, chalan, cash book, lager book, voucher file, rent and service charges file, office operating cost file, communication (telephone/ T.A., etc.), bank transaction, recording daily flow chart<br>■ Preparation of financial reports

Office Assistant
■ Assisting the Project Coordinator in coordinating field and clinical activities<br>■ Organising and scheduling meetings, preparing minutes and ensuring that the quarterly plan of action and budget is adhered to<br>■ Assisting the Project Coordinator in maintaining MIS so that continuous monitoring of field activities is possible<br>■ Aiding the team members in developing BCC materials, conducting street shows, audio-visual programmes, etc.<br>■ Checking/verifying money receipt book and physical stock of condoms, IEC materials, drugs

Considerations in Starting a DIC

- DIC needs to be located where the drug users are or within easy reach of them
- Explain to the community what the DIC is all about, why it is needed and how they will benefit
- Address concerns of the community about
  - their children being harassed by drug users
  - their children watching drug users
  - their cars and homes being vandalized
  - drug use equipments such as pipes, needles, syringes being found near their doorsteps
- Involve the community in the various activities of the centre
  - World AIDS Day observance
  - International day against illicit drugs and trafficking
  - Use community festivals to create awareness
  - Keep the community informed of what is happening

DICs should have a friendly and learning environment that encourages IDUs to relax. It is important to ensure a high level of receptivity before one undergoes an intervention. DICs should be user-friendly and sensitive to the community and should incorporate feedback from clients.

How do we respond to people who come to the DIC?

General Principles

- Make every effort to help the client feel valued and comfortable
- Take consent for testing and medication and emphasise confidentiality
- Listen to the whole story
- Observe the client's physical and emotional condition and jointly agree to an appropriate response
- Attend to needs identified, e.g. nutrition, wound management, etc.
- Identify follow-up action, e.g. referral to hospitals, social support, etc.

Assess client's health risks

- Take the medical history and make a provisional diagnosis (by doctor)
- Enquire about type of drug and mode and patterns of use
- Take history of exposure to contaminated blood and other risk behaviours
- Assess the level of knowledge related to diseases like TB, STIs, HIV/AIDS and hepatitis B and C

Assess client's social well-being

- Find out about any mental health problems
- Enquire about family history
- Note down current residence
- Find out about legal status
- Ask about sexual behaviour and practices
- Make note of education, skills, jobs, etc.

What to do when things go wrong?

Needle stick injury

Wash the injury with soap and water, allow it to bleed and do not pressurise the injured area. Seek advice immediately from the local AIDS control staff for PEP.

Overdose

- Check to see if the person is able to open the eyes or speak to you. Shake and call out the name for a response. Wear protective gloves as a precaution
- Check pulse and breathing

- Call ambulance service if the person is unconscious
- Give mouth-to-mouth resuscitation if breathing has stopped (make sure that there is no skin contact, use CPR mouthpiece if available)
- Try to keep the person alert and awake
- Put the person in the recovery position on the floor
- Clean the mouth if there is vomit
- Never leave someone alone who has overdosed

Seizures

- Ensure the individual is in a recovery position
- Use a hard object such as a spoon between the person's teeth to prevent the person from biting the tongue
- Refer to a doctor for advice

Violence

- In case of violence make sure the management is informed
- Staff who have a good relationship with the client concerned should try to intervene verbally
- Failing this the person must be told that the police will be called
- Those that are not involved must be moved out of the area
- The police may be called or the centre may be temporarily shut down in an emergency
- Make sure that clear procedures in dealing with violence exists

Drug selling and using

- Anyone who is seen selling or using drugs should be made aware of the cardinal rule that prohibits such activity
- Selling drugs puts client, staff and centre at risk of ill repute and danger
- Any action taken must be mindful of existing laws - know the law clearly

Networking for access to additional needed resources

- Ensure a good working relationship with health care institutions such as public hospitals, and with welfare agencies, legal aid bodies and the police
- Ensure that there is good understanding with treatment centres for detoxification and rehabilitation as well as with other DICs
- While using the media as a tool to generate resources, confidentiality must be maintained
- Religious institutions, hotels and public service organisations are a source of additional support (often in-kind)
- Recognise that other services like detox centres or night shelters are needed to provide optimal care: make sure that you have friends among other NGOs
- Be sure that your donors and other well-wishers are kept up to date with developments at your DIC

Challenges of a Drop-In Centre

- To visibly change and improve the conditions of the drug using scene by decreasing violence and crime as well as improve the physical appearance of the drug users
- To ensure that there is a beneficial relationship to the local community that surrounds the DIC
- To supervise medication regimes that require strict adherence and compliance in close coordination with dispensing authorities
- To be consistent in service provision
- To be able to generate sufficient resources to maintain services
- To ensure that a system for compliance with antiretroviral treatment (ART) is in place and that this treatment is made available to street-based drug users
- Flexible working hours that suit the needs of the client
- Policies that are helpful for staff, clients and organisation

ANNEXURE 15

Universal Precautions and Post-Exposure Prophylaxis

Universal Precautions
**Promote a safe work environment**
■ Implement, monitor and evaluate use of universal precautions<br>■ Develop procedures for reporting and treating occupational exposure to HIV infection<br>■ Attain and maintain appropriate staffing levels<br>■ Provide protective equipment and materials
**Education in infection prevention**
■ Make all staff aware of established infection control policies<br>■ Provide ongoing training to build skills in safe handling of equipment and materials<br>■ Supervise and evaluate practices to remedy deficiencies
**Handling of equipment and materials**
■ Assess condition of protective equipment<br>■ Safely dispose of waste materials<br>■ Make available appropriate cleaning and disinfecting agents<br>■ Decontaminate instruments and equipment<br>■ Monitor skin integrity
**Handling and disposal of sharps/disposal containers**
■ Use syringe or needle once only<br>■ Avoid recapping, bending, or breaking needles<br>■ Use puncture-proof container for disposal<br>■ Clearly label container —"SHARPS"<br>■ Never overfill or reuse sharps containers<br>■ Dispose of sharps according to local protocol
**Hand hygiene**
■ Soap and water hand-washing using friction under running water for at least 15 seconds<br>■ Using alcohol-based hand rubs (or antimicrobial soap) and water for routine decontamination
**Personal protective equipment**
■ Gloves—correct size<br>■ Aprons—as a waterproof barrier<br>■ Eyewear—to protect against accidental splash<br>■ Footwear—rubber boots or clean leather shoes<br>■ Safe work practices to reduce occupational risks<br>■ Assess high risk situations and areas<br>■ Develop safety standards and protocols<br>■ Institute measures to reduce occupational stress<br>■ Orient new staff to protocols<br>■ Provide ongoing staff education and supervision<br>■ Develop protocols for post-exposure prophylaxis (PEP)

Post-Exposure Prophylaxis (PEP)	
**Treatment of exposure site**	
Skin	Wash skin with soap and water
Eyes	Rinse eyes immediately with eye wash fluid
Oral exposure	Spit out immediately; rinse mouth immediately several times

- PCR (polymerase chain reaction) testing for HIV is recommended where available
- Inform supervisor of type of exposure and the actions taken
- Assure confidentiality, support and referral for treatment
- Short-course of ARV drugs is recommended to reduce the likelihood of infection
- Document the incident

Administration of Post-Exposure Prophylaxis (PEP)

- Ideally, initiate PEP treatment immediately after exposure and within one hour and not more than 72 hours, if possible. Consider PEP > 72 hours if there was a high risk exposure.
- Duration of PEP: one month
- Regimens: Dual drug therapy is recommended (e.g. ZDU plus 3TC)
- PEP in pregnant women:
  - PEP should be provided if clinically indicated
  - Pregnant women should not receive EFV (efavirenz), tenofovir or the combination of d4T + ddI
  - Preferred PIs in pregnancy are nelfinavir and saquinavir

ANNEXURE 16

Developing a BCC Strategy and IEC Materials for IDUs

BCC Strategy

An effective BCC strategy needs to be developed to guide achievement of intermediate and longer-term outcomes. Examples of BCC objectives are:

- Increased demand for information about HIV and AIDS (IDUs will ask for information about HIV and AIDS)
- Increased knowledge about HIV and AIDS (IDUs will have correct knowledge of modes of transmission of HIV and AIDS.)
- Increased self-risk assessment (IDUs will say that if they do not use disposable syringes or use condoms they feel at increased risk of contracting HIV)
- Increased demand for information on STIs (IDUs will ask for more information on STIs)
- Increased demand for services (IDUs will demand VCTC services)

Observable changes in behaviour, as specified in the behaviour change objectives, are a **final programme outcome**. Such changes are generally preceded by **intermediate changes**. Such changes include:

- **Knowledge change:** an increase in knowledge among targeted IDUs of modes of transmission
- **Attitude change:** an increase in perception of personal risk; also a change in attitude of the authorities toward promoting condoms and towards safe injecting practices
- **Environmental change:** a decrease in harassment of IDUs by police or an increase in acceptance of messages about condom use and safe injecting practices on hoardings, television, etc.

Although some of these changes are not directly related to behaviour change, they can function as necessary environmental antecedents or as shifts that reflect an increasingly supportive environment.

Issues to be addressed through IEC materials

IEC aims to provide accurate information to increase knowledge, modify attitudes and in turn change behaviours to decrease HIV risks.

Modes of delivery

- Mass media
- Targeted information campaigns
- Drug user networks and peers

Special groups to be targeted

- Prisoners
- Ethnic minorities
- Women IDUs
- Sex Workers

Content of materials

- Reduce number of sharing partners
- Cleaning techniques of injecting equipment
- Safer injecting techniques
- Condom use and safer sex
- Reducing indiscriminate sharing of injecting equipment
- Reducing the number of sharing partners and sharing occasions
- Risks of different drug preparation techniques
- Risks of different drug distribution techniques (e.g. front-loading, back-loading)
- Risks of sharing drug injecting paraphernalia (e.g. filters, cookers, water)
- Needle and syringe cleaning/sterilisation techniques
- Access to sterile needles and syringes
- Safe disposal of contaminated injecting equipment
- Alternatives to drug injecting
- Drug treatment services available
- Overdose prevention and management
- Hepatitis B and C prevention
- Abscess and vein care
- Contact details for health, welfare, and other services

ANNEXURE 17

Application Form for Accreditation to Run OST Services

PROPOSAL FORMAT

Please use formats provided – add extra photocopies if necessary.

The proposal must include all the following sections in the order listed:

1. Cover Page

- Name(s) of the implementing organisation(s)
- Title of the project
- Location of the project: specific geographical location of the DIC and its approximate distance (in kms) from the nearest targeted intervention for IDUs, drug detoxification center, Needle Syringe Exchange Programme (NSEP), medical facility for emergency care, VCTC centre, centre for referral for overdose management and co-morbid psychiatric disorders
- Details of the identified IDU community
- Amount of funding requested

2. Proposal Summary (maximum 1 page)

This section provides the key information about the OST intervention. It should be clear and short, but it should contain information on the following:

- Key lessons learnt from past two years' work among the IDU community
- A brief history of the DIC and analysis of the client turnover at the DIC and the TI (if applicable) in the past two years, with specific data on injecting drug users enrolled in the DIC and at the TI
- Description of procurement and supply chain management systems to be established by the applicant organisation for minimising the abuse potential of buprenorphine
- Objectives
- Activities
- Inputs, i.e. staff and requested budget (detailed training plans for the core team of medical and paramedical staff for quality clinical services)
- Expected outputs

3. Baseline Assessment and Follow-up Assessment Plans of OST Clients (maximum 2 pages)

The information for this section should be drawn from the baseline assessment (by the applicant organisation). The information for the baseline assessment of the OST can be gathered through focus group discussions, key informant studies and the records of the DIC and the TI outreach staff.

Baseline and follow-up assessment[1] questionnaires should include:

- Drug use history, medical history, psychosocial assessment, monitoring for side effects
- Proportion of IDU clients (denominator – number of clients in the DIC and the TI)
- Average daily visits of the IDUs to the DIC in the past 6 months (for DIC clients only)

[1] Follow-up done periodically at predefined intervals (recommendation is initially twice-weekly follow-ups for at least two weeks, followed by fortnightly and subsequently monthly follow-ups).

- Reported injecting behaviour practices (individual/group) by IDUs
- Reported frequency of injected drug use in the past 6 months by IDUs and the sexual partners (both male and female)
- Type of services accessed by the drug user/IDUs and their sexual partners
- Health care seeking behaviour for HIV and drug related issues (e.g. management of hepatitis C, drug overdose, testing, abscess or HIV, access to ARVs, etc.)

4. Constraints

Constraints (human, financial resources, issues of capacity, etc.) faced by the applicant organisation in the past two years and the strategy adopted to overcome these.

5. Description of the Project Area

This must be provided in terms of:

- Involvement of injecting drug users community in the design/implementation of the project
- Availability of target audience and sites for conducting outreach intervention
- Mapping of sites of injecting drug users and their approximate distance from the DIC
- Distance and feasibility for providing BCC intervention and health care services
- Participation/involvement of secondary stakeholders and the potential of initiating peer education

6. Implementation of OST Programme

- **Goals and objectives of the DOT intervention for OST**
- **Project implementation:** activities, time frame, staff requirement and work plan
- **Planned coverage**: What is your planned coverage?[2] What are the plans for community outreach for recruiting patients in treatment and facilitating retention? What outreach and follow-up strategies will be used to achieve a reasonable retention rate for clients in the OST programme?
- **Specific inclusion/exclusion criteria:** Issues related to induction in buprenorphine and increasing the dosage, duration of treatment as well as tapering of buprenorphine are clearly defined.
- **Delivery of OST services:** Who is the proposed person for screening clients to start OST (a trained medical doctor is essential)? Who follows up the client? Who administers the OST drug? What are the proposed mechanisms to ensure that the client does not divert the OST drug for injecting? How is counselling and delivery of other services ensured?
- **Detailed staffing plan** for OST[3] (including details of training received by the staff, back-up coverage for absence or leave of the medical doctor/core team)

---

[2] A maximum planned coverage of 200 clients is required for any government college, hospital or NGO which has a Drop-In Centre (DIC) to be eligible for applying for accreditation. Please specify how many clients would be drawn from the nearest TI (on IDU) and how many clients would be drawn from the DIC.

[3] Staffing should include one full-time doctor / part-time (thrice weekly) who has been trained in OST, and a full time nurse, full-time counsellor, 3 security staff/office boys to manage the drugs

- **Record maintenance:** What is the system devised for record maintenance? (Special mention should be made of records for starting a client on OST drug, follow up and monitoring of client during follow up, stock registers.)

7. Plans to provide other critical HIV and drug services[4]:

- Drug treatment services (detoxification centres, psycho-social interventions)
- Management of abscess, TB, STI, AIDS (ARV available)
- Referral network of medical specialists
- VCTC, routine clinical chemistry
- Overdose management
- Management of co-morbid psychiatric disorders
- Behaviour change and harm reduction counselling, condom promotion
- Relapse prevention and family counselling

8. Systems Established

Description of drug procurement, supply and safe-keeping (the system description should provide information on what checks and balances have been put in place to prevent diversion of drugs for illicit use).

9. Availability of Space and Basic Infrastructure[5]

Description of size of the DIC and the plan to establish the OST programme.

10. Monitoring and Evaluation Plan

Duration at which the clients would be followed up on, including the record-keeping system, dispensing units, stock registers, case records.

11. Detailed Budget, Inputs and Human Resources

12. Attachments (if any)

---

[4] Contact details of each service and the approximate distance from the DIC to be specified in the proposal

[5] NACO guidelines specify a minimum area of 8 feet by 8 feet for counselling.

ANNEXURE 18

Checklist for Scoring Proposals to Run OST Services

The proposed format for developing a checklist to provide accreditation to a particular agency for running OST services is as follows. In order to enable the scoring for each of the factor blocks the breakdown of factors is provided below:

1. **Reflection of the understanding of the findings of the baseline assessment**

— Out of 5	Understanding of socio-demographic profile of the IDU community.
— Out of 5	Understanding of the injecting drug use including prevalence, types of drugs used in the target area and the health care seeking behaviour of IDUs and their sexual partners.
— Out of 5	Understanding of the injecting drug user community's knowledge, attitude and practices in relation to HIV/AIDS prevention.
— Out of 5	Proposed OST intervention design based on the needs of the injecting drug users.
**— Out of 20**	**Total points for factor block 1**

2. **Clarity in drawing up goals, objectives and activities including output and outcome indicators**

— Out of 5	Developing goals, objectives, outputs and outcomes based on the baseline assessment.
— Out of 5	Clearly stated measurable goals, objectives, output and outcome indicators.
— Out of 5	Linkages between goals, objectives, outputs and outcome indicators.
**— Out of 15**	**Total for factor block 2**

3. **Demonstrate understanding of the comprehensive package of services for IDUs**

— Out of 5	Has the proposal listed the agencies providing HIV and drug prevention, care and treatment services and other schemes?
— Out of 5	Does the proposal indicate the approaches that would be adopted for involving the IDU community in the design and implementation of the OST programme?
— Out of 5	Does the proposal clearly articulate the mechanism for establishing referral and follow-up with TIs for IDUs, VCTC, ARV centres, PPTCT programmes and drug detoxification centres?
**— Out of 15**	**Total for factor block 3**

4. **Demonstrated understanding of setting up an OST programme**

— Out of 5	Does the proposal clearly articulate the specific inclusion, exclusion criterion for clients on OST inline with the national guidelines?
— Out of 10	Is the staff available to deliver OST services as required in the NACO operational guideline? (Check specifically if it has been clearly mentioned in the proposal: who is responsible for starting to administer OST to a client? Who is responsible for administering OST on a daily basis? What are the procedures followed to ensure that the client does not divert the OST drug for injecting?)
— Out of 5	Has the staff received adequate training to run OST service? (Focus on those delivering OST service, especially doctor and personnel responsible for administering drugs.)
— Out of 5	Is the detailed staffing plan adequate to provide back-up coverage for the absence of leave of the medical doctor/core?
— Out of 5	What is the proposed system of record maintenance (for screening and follow-up of clients, dispensing of OST drug, other interventions carried out)?
— Out of 10	Does the proposal clearly indicate the systems to be established for drug procurement, supply and safe-keeping to prevent diversion of drugs for illicit purposes?
**— Out of 40**	**Total for factor block 4**

5. **Monitoring and Evaluation Plan**

— Out of 5	Does the proposal indicate the duration of baseline, midline and follow-up assessment of clients on OST?
— Out of 5	Are the monitoring mechanisms (record keeping system for dispensing units, stock registers and case records, field-based information system including documentation, deliverables, benchmarks, etc.) clearly listed in proposal along with timeline?
**—Out of 10**	**Total for Factor block 5**

ANNEXURE 19

Quality Assurance Protocol

TI Components	Quality Control	Quality Assessment	Quality Improvement
**Clinical Services**	▪ Checklist for doctors<br>▪ Checklist for counsellor<br>▪ Check list for clinical infrastructure and environment<br>▪ Monitoring visit<br>▪ Surprise visit	▪ Patient feedback<br>▪ Registers and reports<br>▪ (quantitative and qualitative)<br>▪ Quarterly evaluation by external evaluator	▪ To address the gap identified through quality assessment<br>▪ Initial planning will be changed considering situation to provide quality services<br>▪ Discussion regarding reports with staff and community
**BCC Activities**	▪ Ensure community's involvement in developing IEC resources and also ensuring quality of IEC material<br>▪ Evaluation of resources<br>▪ Checklist for ORW and counsellors to ensure effective BCC<br>▪ Checklist for conducting group meeting<br>▪ Assess knowledge, attitude and behaviour practice (KABP)	▪ Reports on field interaction<br>▪ Group meeting for needs assessment<br>▪ Reports of the process on documentation<br>▪ Interaction<br>▪ Quarterly evaluation (both participatory and non-participatory method)	▪ Reports findings will be shared in meeting<br>▪ Modification according to the feedback through effective one-to-one and group discussion
**Counselling**	▪ Imply protocol for counsellor<br>▪ Environment of counselling set-up<br>▪ Assess KABP	▪ Assessing record and procurement procedure	▪ Changes implemented according to feedback and reports
**Condom Promotion**	▪ Staff capacity regarding social marketing<br>▪ Method of using condoms<br>▪ Introduced multiple brands of condom<br>▪ Fixing up of target for condom distribution<br>▪ Maintaining the quality of condoms<br>▪ Ensure easy accessibility<br>▪ Ensuring community participation for developing effective social marketing plan<br>▪ Assess KABP	▪ Condom demo by community members<br>▪ Clients' feedback on quality, accessibility and affordability<br>▪ Monitoring Report (qualitative and quantitative)	▪ Initial planning will be changed considering situation to provide quality services
**Procurement/ Stocking**	▪ Inventory list<br>▪ Identification of suppliers on the basis of goodwill, quality and availability of materials, supply of materials within time<br>▪ Physical verification of stock at 15-day intervals by office support staff, quarterly by PC and annually by auditor from SACS	▪ To address the gap identification through quality assessment<br>▪ Assessing record and procurement procedure	▪ Effective steps will be taken as early as possible to bridge the gap

TI Components	Quality Control	Quality Assessment	Quality Improvement
**NSEP**	▪ Monitoring visit for checking expiry dates of needle and syringes<br>▪ Ensuring stoppage of recycling of used needles	▪ Quarterly report	▪ Bridging gaps for gaps in the services
**Substitution**	▪ Ensuring quality provision of buprenorphine sublingual<br>▪ Appropriate counselling<br>▪ Quality abscess management process	▪ Quarterly report	▪ Bridging gaps for gaps in the services
**Drop-in Centre**	▪ Maintenance of minimum standard guidelines for setting up the DICs	▪ Tracking on DIC update<br>▪ Feedback from HRG on accessibility and standard of DIC	▪ Setting up Community DIC Monitoring Committee<br>▪ Upgrade of DIC services as per the needs of the HRG
**Advocacy**	▪ Development of strategic advocacy plan	▪ Periodic review of proportions of HRG members regularly accessing the services<br>▪ Tracking of Harassment cases	▪ Formation of "Rapid Response Team" to address the issue<br>▪ Formation of District-specific Advocacy Committee<br>▪ Advocacy skills development among the NGO's staff and HRG

ANNEXURE 20

Indicators Scoring Sheet

Indicators	Score (1 = lowest, 5 = highest)
**1. Performance of the project implemented by the NGOs as measured through standard indicators pro vided by SACS/PSU**	
▪ Information on STI/HIV/AIDS	
▪ Linkages with VCTC and ART services	
▪ STI treatment	
▪ Linkages developed for other perceived needs	
▪ Condoms	
**2. Ability to create an enabling environment in and around the community**	
▪ Level and trends of violence in the project site (type and nature of harassment, incidence of violence and perpetrators of violence)	
▪ Transactional ability of the community members with clients/local goons/police/madams on relevant issues	
▪ Free movement on the part of the community members in and across the project site as well as in the geographical area	
▪ Local policy makers are aware of the intervention and/or supportive of the project	
▪ Level of networking with other organisations including various government departments	
**3. Key communities' overall presence in the project**	
▪ Level of trust observed between the project and the community members	
▪ Level of networks developed and functional both within and across the project as well as in the larger communities in geograph ical region (preferably at the District level)	
▪ Visibility of the core group within the project purview	
▪ Perceived needs of the community are identified and attempts are being made to address those	
▪ Number of community members functioning as peers/staff in the programme and/or playing role in different committees	
**4. Interest and the attitude of the implementing NGOs and their staff members for building community ownership**	
▪ Board and executive members' overall perception about the HRG	
▪ Job responsibilities of different categories of staff members emphasise capacity building of HRG	
▪ Community friendly monitoring system in place where community members are playing crucial role	
▪ Attitude of project staff as perceived/assessed by the community members	
▪ Efforts to build capacity of the HRG and attempt to delegate responsibility to them	

Note: Each of the 5 sub-indicators can score up to 5 marks, making the maximum sub-total for each main indicator 25 marks. In order to qualify for the transition, NGO must score 60 or above with a minimum of 15 in each indicator.

ANNEXURE 21

Site Selection Scoring Sheet

Comparative Matrix to Determine Feasibility based on Selected C riteria											
Sex work site	Size (No. of sex workers)	Sex work volume (no. of sex workers X no. of dients)	Cohesive-ness among the sex workers	Stability of sex work at the site	Mobility of sex workers	Relative indepen-dence of sex workers	Incidence of violence	History of collective resistance showed by the community against injustice, discrimination, etc.	Feasibility of reaching clients in the area	Existence of interventions run by NGO in the area	Total

National AIDS Control Organization
Ministry of Health & Family Welfare
Government of India
