# Supplementary figures and images for: Effect of Physical Violence on Sexually Transmitted Infections and Treatment Seeking Behaviour among Female Sex Workers in Thane District, Maharashtra, India
Source: PLoS One. 2016 Mar 2;11(3):e0150347. doi: 10.1371/journal.pone.0150347 (PMC4774990; doi:10.1371/journal.pone.0150347)

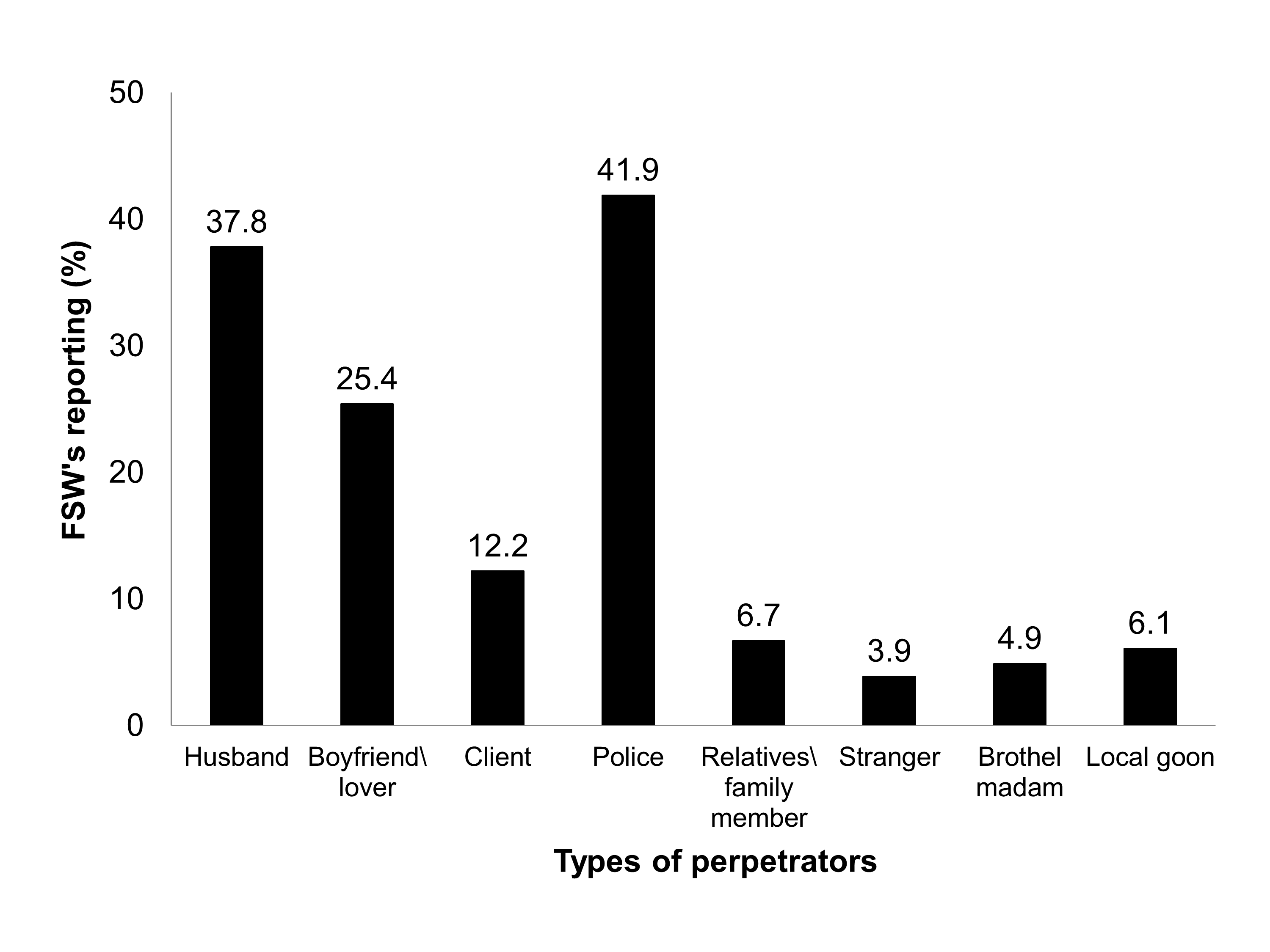

Supplement: S1 Fig — FSWs reported physical violence was perpetrated by various perpetrators in Thane district. The key perpetrators of physical violence were regular partners (husband or boy-friend or lover), occasional or regular clients including the stranger, local goons (local criminal or violent person in the community), police, and others in Thane district. (TIF) [file pone.0150347.s002.tif]
