# Supplementary material for: Effect of Physical Violence on Sexually Transmitted Infections and Treatment Seeking Behaviour among Female Sex Workers in Thane District, Maharashtra, India
Source: PLoS One. 2016 Mar 2;11(3):e0150347. doi: 10.1371/journal.pone.0150347 (PMC4774990; doi:10.1371/journal.pone.0150347)
Supplement: S1 Table — (DOCX) [file pone.0150347.s003.docx]

| **Table S2.** Results from multivariate logistic regression analysis showing association between selected demographic and sexual behaviour related characteristics with outcomes on STI and treatment seeking | | | | | | | | |
| --- | --- | --- | --- | --- | --- | --- | --- | --- |
| **Background and sexual behaviour variables** | **Reported any STI symptom in last 12 months** | |  | **Reported multiple STI symptoms in last 12 months** | |  | **Sought treatment for the last STI symptom** | |
|  | **Adjusted OR (95% CI)** | **p-value** |  | **Adjusted OR (95% CI)** | **p-value** |  | **Adjusted OR (95% CI)** | **p-value** |
| **Experienced any physical violence in last 12 months** |  |  |  |  |  |  |  |  |
| No | Ref |  |  | Ref |  |  | Ref |  |
| Yes | 1.848 (1.248-2.736) | 0.002 |  | 1.353 (0.911-2.009) | 0.134 |  | 1.440 (0.978-2.120) | 0.064 |
| **Current age#** | 1.016 (0.985-1.048) | 0.319 |  | 1.008 (0.976-1.042) | 0.626 |  | 0.998 (0.967-1.031) | 0.921 |
| **Marital status** |  |  |  |  |  |  |  |  |
| Never married | Ref |  |  | Ref |  |  | Ref |  |
| Ever married | 1.337 (0.756-2.364) | 0.318 |  | 0.972 (0.540-1.749) | 0.924 |  | 1.260 (0.697-2.278) | 0.445 |
| **Literacy status** |  |  |  |  |  |  |  |  |
| Non-literate | Ref |  |  | Ref |  |  | Ref |  |
| Literate | 1.480 (1.064-2.060) | 0.020 |  | 1.564 (1.107-2.209) | 0.011 |  | 1.383 (0.984-1.942) | 0.062 |
| **Residence** |  |  |  |  |  |  |  |  |
| Localite | Ref |  |  | Ref |  |  | Ref |  |
| Non-localite | 2.453 (1.498-4.015) | 0.000 |  | 1.569 (0.927-2.655) | 0.094 |  | 1.705 (1.017-2.858) | 0.043 |
| **Any co-resident** |  |  |  |  |  |  |  |  |
| No | Ref |  |  | Ref |  |  | Ref |  |
| Yes | 0.950 (0.611-1.478) | 0.820 |  | 1.265 (0.795-2.011) | 0.321 |  | 1.062 (0.680-1.660) | 0.791 |
| **Any source of income other than sex work** |  |  |  |  |  |  |  |  |
| No | Ref |  |  | Ref |  |  | Ref |  |
| Yes | 0.713 (0.509-1.001) | 0.050 |  | 0.722 (0.507-1.028) | 0.070 |  | 0.550 (0.388-0.780) | 0.001 |
| **Any savings** |  |  |  |  |  |  |  |  |
| No | Ref |  |  | Ref |  |  | Ref |  |
| Yes | 0.532 (0.249-1.138) | 0.104 |  | 0.491 (0.202-1.193) | 0.116 |  | 0.655 (0.296-1.449) | 0.296 |
| **Consume alcohol** |  |  |  |  |  |  |  |  |
| No | Ref |  |  | Ref |  |  | Ref |  |
| Yes | 1.129 (0.797-1.600) | 0.494 |  | 0.680 (0.473-0.978) | 0.038 |  | 0.905 (0.635-1.290) | 0.580 |
| **Age at first sex** |  |  |  |  |  |  |  |  |
| <15 years | Ref |  |  | Ref |  |  | Ref |  |
| 15 or more years | 1.019 (0.962-1.078) | 0.523 |  | 1.050 (0.989-1.115) | 0.109 |  | 0.996 (0.939-1.056) | 0.894 |
| **Duration in sex work** |  |  |  |  |  |  |  |  |
| <3 years | Ref |  |  | Ref |  |  | Ref |  |
| 3 or more years | 0.996 (0.962-1.031) | 0.836 |  | 0.997 (0.962-1.033) | 0.874 |  | 1.032 (0.996-1.069) | 0.081 |
| **Place of solicitation** |  |  |  |  |  |  |  |  |
| Non-public | Ref |  |  | Ref |  |  | Ref |  |
| Public places | 1.153 (0.775-1.715) | 0.482 |  | 1.443 (0.961-2.166) | 0.077 |  | 0.845 (0.562-1.272) | 0.420 |
| **Weekly client volume** |  |  |  |  |  |  |  |  |
| <10 clients | Ref |  |  | Ref |  |  | Ref |  |
| 10 or more clients | 0.952 (0.590-1.537) | 0.841 |  | 1.197 (0.725-1.976) | 0.481 |  | 1.151 (0.709-1.871) | 0.569 |
| **Exposure to programme** |  |  |  |  |  |  |  |  |
| No | Ref |  |  | Ref |  |  | Ref |  |
| Yes | 1.338 (0.782-2.289) | 0.288 |  | 2.738 (1.482-5.060) | 0.001 |  | 1.196 (0.682-2.100) | 0.532 |
| **Consistent condom use with regular partners** |  |  |  |  |  |  |  |  |
| No | Ref |  |  | Ref |  |  | Ref |  |
| Yes | 1.316 (0.927-1.867) | 0.124 |  | 1.189 (0.823-1.718) | 0.357 |  | 1.041 (0.727-1.490) | 0.828 |
| **Consistent condom use with regular clients** |  |  |  |  |  |  |  |  |
| No | Ref |  |  | Ref |  |  | Ref |  |
| Yes | 0.643 (0.429-0.965) | 0.033 |  | 0.829 (0.547-1.255) | 0.375 |  | 1.055 (0.697-1.597) | 0.799 |
| Ref: Reference category; #included as a continuous variable in the model | |  |  |  |  |  |  |  |
